# Acknowledgment to Reviewers of the *International Journal of Molecular Sciences* in 2020

**DOI:** 10.3390/ijms22031123

**Published:** 2021-01-23

**Authors:** International Journal of Molecular Sciences Editorial Office

**Affiliations:** MDPI AG, St. Alban-Anlage 66, 4052 Basel, Switzerland

Peer review is the driving force of journal development, and reviewers are gatekeepers who ensure that the *International Journal of Molecular Sciences* maintains its standards for the high quality of its published papers. Thanks to the cooperation of our reviewers, in 2020, the median time to first decision was 15 days and the median time to publication was 33 days. The editors would like to express their sincere gratitude to the following reviewers for their precious time and dedication, regardless of whether the papers were finally published: Aaron, Jean E.Liu, WenxianAaseth, JanLiu, XiaoboAbad, CatalinaLiu, XiaojieAbadía, JavierLiu, XiaoyingAbalde-Cela, SaraLiu, YajunAbashev, George G.Liu, YangAbate, GiuliaLiu, Yi-ChangAbba, Mohammed L.Liu, YingjunAbballe, AnnalisaLiu, YonggangAbbate, LoredanaLiu, YongliangAbbina, SrinivasLiu, YuAbd El Hakim, YasminaLiu, Yu-ChangAbdallah, AliLiu, Yu-PengAbdallah, Mohamed F. Liu, YutaoAbdelalim, Essam M.Liu, ZhaohuiAbdelhafiz, Ahmed HLiu, ZhiAbdelkarim, HazemLiu, ZhipengAbdellatif, AhmedLiu, ZhixiaAbdelmohsen, Usama RamadanLiu, ZhongweiAbdul-Muneer, P. M.Liu, ZiqingAbe, MasahiroLiutkevičienė, RasaAbe, TatsuyaLivanos, PantelisAbedi Ardekani, BehnoushLiverani, LilianaAbedi, TayebehLivingston, DavidAbend, MichaelLiWang, PatriciaAberg, ChristofferLiwinienko, GrzegorzAbete Goñi, ItziarLizard, GérardAbeykoon, JithmaLizcano, FernandoAbia, Akebe Luther KingLizcano, Jose M.Abicht, AngelaLleo, AnaAbinaya, ManivannanLlopis-González, AgustínAbkhalimov, Еvgeny V.Llorens Martin, MariaAbo-Ismail, MohammedLlorens, EugenioAbondio, PaoloLlorens, JordiAboubakar, FrankLlorente, IreneAboudehen, KaramLloret, AnaAbraham, DietmarLluna, Amparo GameroAbraham, NaderLo Giudice, AntoninoAbrahams, Jan PieterLo Giudice, RobertoAbramyan, JohnLo Muzio, LorenzoÁbrányi-Balogh, PéterLo Piero, Angela RobertaAbubakr, Neamat HassanLo Sardo, FedericaAbu-Reidah, Ibrahim M.Lo, Chunmin C.Abu-Romman, SaeidLo, Kai-YinAbutarboush, RaniaLo, SzechengAccardi, LuisaLobanova, Ekaterina S.Accardo, AngeloLobo, Glenn P.Acedo Nunez, Maria Del PilarLocascio, AntonellaAcharya, ArpanLocatelli, GiuseppeAcharya, GhanashyamLochhead, Jeffrey J.Achim, CristinaLochmanová, GabrielaAchiron, AsafLochner, MartinAcosta-Jurado, SebastiánLockshin, Michael D.Acuña-Castroviejo, DaríoLodge, Katharine M.Adachi, HiroakiLodi, AlessiaAdachi, NaokiLodyga-Chruscinska, ElżbietaAdachi, ShunsukeLoebel, ClaudiaÁdám, Attila L.Loeffler, IvonneAdam, GerhardLoessner, DanielaAdamakis, Ioannis-DimosthenisLogan, DerekAdamcakova-Dodd, AndreaLoginov, DmitriyAdamczyk, AgataLognay, Georges C.Adamek, JakubLohan, SilkeAdamopoulos, PanagiotisLohmann, VolkerAdams, Bryn L.Lohning, AnnaAdams, JamesLohr, ChristianAdams, VolkerLoid, JosefAdamska, AgnieszkaLoiselle, Denis S.Adawy, AlaaLoizzo, Monica RosaAdegbola, RaphaelLojkić, IvanaAdekoya, Timothy O.Lojkova, LeaAdell, TeresaLojou, ElisabethÅdén, JörgenLok, Chun NamAdey, DeborahLoka, DimitraAdhikari, ManishLokensgard, James RAdhikari, NeetaLokman, MarkAdhikary, AmitavaLokshin, AnnaAdil, Syed FarooqLombardi, AssuntaAdina, Turcu-StiolicaLombardi, FrancescaAdkar-Purushothama, Charith RajLombardi, GiuseppeAdney, DanielleLombardi, RaffaellaAdolf-Bryfogle, JaredLombardi, Vincent C.Adrover, AlessandraLombardo, Giovanni EnricoAdt, IsabelleLomovatskaya, LidiaAdur, MalavikaLonardo, AmedeoAdvani, VivekLonardo, EnzaAfanassieff, MarielleLončar, Mirela BausAfantitis, AntreasLondei, PaolaAfolayan, Adeleye J.London, Sarah E.Afonso, Clélia NevesLonetti, AnnalisaAgarwal, GauravLong, Chiau MingAgarwal, PoonamLong, YichengAgatemor, ChristianLong, YuchenAgeitos, Jose ManuelLongo, AntonioAggarwal, Bharat B.Longo, ValentinaAggeli, Ioanna-KaterinaLongone, PatriziaAggrey, Samuel E.Longy, MichelAgić, DejanLönnberg, HarriAgirre, JonLopes, Alberto Jorge OliveiraAgnelli, LucaLopes, GraçaAgnieszka, GizakLópez Lluch, GuillermoAgostino, MarkLópez Malo, DanielAgoulnik, AlexanderLópez Martínez, JuanAgrawal, Sandeep K.Lopez Martinez, Maria JoséAguennouz, M.López Nicolás, Jose ManuelAguilera-Aguirre, LeopoldoLópez Sesé, Ana IsabelAguirre, AitorLopez, César A.Agusa, TetsuroLópez, CristinaAgustí-Brisach, CarlosLopez, Jose CristobalAhmad, AamirLopez, Jose JavierAhmad, AliLópez, Luis CarlosAhmad, AsrarLopez, SophieAhmad, FahimLópez-Camarillo, CésarAhmad, IbrarLopez-Canul, MarthaAhmad, ShakilLopez-Castejon, GloriaAhmed, Aisha SiddiqahLópez-Cebral, RitaAhmed, Atif AliLopez-Escamez, Jose A.Ahmed, ChulbulLopez-Ferber, MiguelAhmed, HafizLópez-Gallego, FernandoAhmed, MukhtarLópez-Guerrero, José AntonioAhmed, NuzhatLópez-Hernández, Luz BereniceAhmed, Shafiq ULópez-Hernández, YamiléAhmed, ZubairLópez-Huertas, María RosaAhn, Chi BumLópez-López, DanielAhn, Dong-KukLópez-López, ElixabetAhn, Eun YoungLópez-López, ManuelAhsan, Md. ArifulLópez-Marqués, Rosa LauraAiba, TakeshiLópez-Pintor Muñoz, Rosa MaríaAiba, YuichiroLópez-Ruiz, ElenaAidinis, VassilisŁopieńska-Biernat, ElżbietaAiello, FrancescaLoppi, StefanoAimi, JunkoLoppi, SteffanoAine, MattiasŁopusiewicz, ŁukaszAirenne, KariLora, JorgeAires, Inês DinisLorberboum-Galski, HayaAisa, Maria CristinaLordan, RonanAissat, AbdelLordkipanidzé, MarieAitken, Karen S.Lorenz, AlexanderAjduk, AnnaLorenz, KristinaAjjan, RamziLorenz, RonnyAkahoshi, MitsuteruLorenz, SusanneAkam, Elizabeth CLorenzo González, ÓscarAkhgari, AmirLorenzo Sanchez, OscarAkhtar, YasminLorenzo, SempereAkimov, Sergey A.Loreti, ElenaAkitsu, TakashiroLorite, PedroAkiyama, MasashiLosada-Perez, PatriciaAkk, GustavLoschenov, Victor B.Akopian, ArmenLoskog, AngelicaAkula, ShawLossius, AndreasAl Bari, Md. AbdullahLosurdo, GiuseppeAl Rihani, SweilemŁotocka, BarbaraAlaimo, AlessandroLotti, Lavinia VittoriaAlain, TommyLouis, SandrineAlam, MobashwerLoura, Luís M.S.Alam, PerwezLourenço, TiagoAlam, ShafiulLouveau, AntoineAlamillo, Josefa MuñozLovat, FrancescaAlansary, DaliaLovato, LauraAl-Aubaidy, HayderLovatt, MatthewAlavi, SamanLovisa, SaraAlavi, Seyed EbrahimLovisolo, ClaudioAlbano, EmanueleLöw, PéterAlbarracín, Sonia L.Lowe Jr., William L.Albeck, JohnLowe, GillianAlbericio, FernandoLowe, MartinAlbert, MareikeLowery, JonathanAlbertolle, MatthewLowin, TorstenAlbrecht, RandyLozano, R. M.Albrechtsen, Nicolai Jacob WewerLozano-Elena, FidelAlcalde, MireiaLozano-Lorca, MacarenaAlcántara-Ortigoza, Miguel AngelLu, AilingAlcaro, StefanoLu, Chih-HaoAlcolea, VeronicaLu, Chi-YuAlcover, AndresLu, Chung-kuangAldabe, RafaelLu, Da-WenAldámiz-Echevarría, LuisLu, HongAlder, JanetLu, Hong S.Aldieri, ElisabettaLu, JingAldred, NicholasLu, Kuo-ChengAlduina, RosaLu, LinchaoAl-Dujaili, EmadLu, LixiaAlecsandru, Diana M.Lu, Ming-WeiAleffi, MicheleLu, Pei-LuenAlejandro, SantiagoLu, QuanlongAleksandrovych, VeronikaLu, SizhaoAlekseev, AlexeyLu, WeiAleksova, AnetaLu, Wei-YuAlessenko, Alice V.Lu, XiaoAlessio, NicolaLu, YangAleti, GajenderLu, YangfanAleu, JosefinaLu, YongkeAlewine, ChristineLu, YouAlexander, DorotheaLu, YubingAlexander, Francis Borgio J.Lu, YujiaoAlexander, H. DenisLu, ZhanpingAlexander, MathewLualdi, MartaAlexander, RebeccaLubawy, JanAlexandrescu, SandaLubecka-Pietruszewska, KAlexandrov, Oleg S.Lubell, William D.Alexov, EmilLuca, AndreiAlfano, DanielaLuca, GiovanniAlfano, MassimoLuca, MariaAlfarouk, Khalid OmerLuca, RossellaAl-Fatimi, MohamedLucaciu, Constantin MihaiAlfei, SilvanaLucantoni, FedericoAlfieri, Maria LauraLucarelli, EnricoAlfirevic, AnaLucas, Heather R.Al-Ghadban, SaraLucas, Jose AntonioAl-Haj-Zen, AymanLucchese, AlessandraAlhakamy, NabilLucchetti, DonatellaAlhenc-Gélas, FrancoisLucena, CarlosAl-Hilal, Taslim AhmedLucenti, ElenaAli, AkhtarLucero, DiegoAli, AliLuchetti, AndreaAli, Hayssam M.Luchetti, Michele M.Ali, TahirLuchian, TudorAliaño González, María JoséLuchinat, ClaudioAlicja, Macko-PodgorniLuchini, ClaudioAliev, GjumrakchLucic, VladanAliki, XanthopoulouLucioli, SimonaAlina, WiszniewskaLuck, RudyAlique, MatildeLuckman, SimonAlizargar, JavadLuddi, AliceAljabali, Alaa A ALuddy, Kimberly A.Alkalaeva, ElenaLudidi, NdikoAllan, AlisonLudlow, Andrew T.Allegra, MarioLudwig, AndreasAllegrucci, CinziaLudwig-Müller, JuttaAllen, Bruce G.Ludwikõw, AgnieszkaAllen-Petersen, BrittanyLue, Ko-HuangAllijn, Iris ELuebbert, ChristianAllikmets, Rando L.Luengo, YurenaAllison, LizabethLuethi, DinoAllison, SimonLugnier, ClaireAllison, W. TedŁugowska, AgnieszkaAllmang, ChristineLui, KathyAlloza, IraideLukacher, Aron E.Allu, Prasanna K.R.Lukas, JanAlmahli, HadiaŁukasiuk, KatarzynaAlmansa, María SoledadLukaszewski, Adam J.Almasan, AlexŁukomska, AgnieszkaAlmasri, MaherŁukowski, AdrianAlmeida, Luciana OliveiraLukyanov, Konstantin A.Almeida, Maria RosárioLuna, BelénAlmeida, PedroLund, Elizabeth K.Almeida, Vitor HugoLundborg, MagnusAlméras, TancredeLuneau, DominiqueAlmerico, Anna MariaLung, Shiu CheungAl-Mrabeh, AhmadLungaro, LisaAl-Mubarak, Bashayer R.Lunov, OlegAl-Mutairi, NawafLunt, PeterAloman, CosticaLuo, Chi-WenAlonge, SalvatoreLuo, LinAlonso, Juan CarlosLuo, MinkuiAlonso, M. Beatriz DuránLuo, RayAlonso, SergioLuo, WeiboAlpaugh, KatherineLuongo, LivioAlQuraishi, MohammedLupien, AndréanneAlsaab, HashemLupini, AntonioAlsadeq, AmeeraLupu, StelianAlshareef, AbdulraheemLuque, FranciscoAlsina, BertaLurz, EberhardAltamirano-Bustamante, Myriam M.Lusa, MerjaAltamura, GennaroLushington, GerryAltmann, BrigitteLustgarten, MichaelAltmann, FriedrichLustig, ArthurAltmeppen, HermannLustig, YanivAltomonte, JenniferŁuszczki, JarogniewAltucci, LuciaLüthje, SabineÁlvarez Martín, JavierLutter, ErikaAlvarez, Ana I.Lüttge, Ulrich E.Alvarez, ClaraLutz, Pierre-EricAlvarez, GuzmánLuuk, HendrikAlvarez, Juan B.Luvisi, AndreaAlvarez, MartinaLuzio, AnaÁlvarez-González, Carlos AlfonsoLuznik, ZalaÁlvarez-Mercado, Ana IsabelLymperopoulos, AnastasiosAlvarez-Pizarro, Juan CarlosLyu, Ping-ChiangÁlvarez-Sánchez, NuriaLyubchenko, YuriÁlvaro, TomásLyukmanova, Ekaterina N.Alves E Silva, Thiago LuizM’kacher, RadhiaAlves Lopes, João PedroMa, GuojiaAlves, C. HenriqueMa, HongAlves, CelsoMa, JiyanAlves, JoanaMa, LishaAlves, MarcoMa, PeisongAlves, NunoMa, QianliAlves, SandraMa, Suk LingAlves, ViniciusMa, WanshuAlyamani, MohammadMa, WeiAlyass, AkramMa, XiaochaoAlzubaidi, LaithMa, XiaochiAmabilino, DavidMa, YiyiAmadio, MarialauraMaak, SteffenAmadio, SusannaMaass, KendraAmador-Noguez, DanielMaaß, SandraAmantini, ConsueloMabbott, NeilAmaradhi, RadhikaMabbott, SamuelAmaral, AlexandraMaccallini, CristinaAmaral, Ana ClaudiaMaccari, AlbertoAmaral, OlgaMaccarinelli, FedericaAmaral, RonaldoMacchi, SamnthaAmarelli, CristianoMacDonald, Clinton C.Amarilla, Alberto A.Macedo, AntóniaAmaro, FranciscoMacek Jilkova, ZuzanaAmasheh, SalahMacek Jílková, ZuzanaAmata, EmanueleMacEwan, DavidAmato, AntonellaMach, MojmirAmato, FeliceMachado, RaulAmbrose, ChrisMachairiotis, NikolaosAmbrosini, GraziaMachelart, ArnaudAmélie, SaunierMachetti, FabrizioAmeln, Anne Klotzsche-vonMachon, OndrejAmeloot, MarcelMachuca-Gayet, IrmaAmenabar, MaximilianoMachuca-Portillo, GuillermoAmendola, RobertoMacías, RamónAmengual, JaumeMaciej, StrzemskiAmes, James BMaciejewska, BarbaraAmiel, CatherineMaciejewski, RyszardAmiel, EyalMacín, Stella MarisAmillis, SotirisMacirella, RacheleAmils, RicardoMaciver, BryceAmin, KawaMacIver, M. BruceAmin, Muhammad JunaidMackiewicz, AndrzejAmin, Nirav H.Mackiewicz, DorotaAmin, RajeshMacNeill, AmyAmini, HajarMaçôas, ErmelindaAminin, DmitryMacori, GuerrinoAMIRAL, JeanMacRaild, ChristopherAmiri, AchourMączka, WandaAmirkhani, MasoumeMadak-Erdogan, ZeynepAmit, MoranMadala, SatishAmital, HowardMadaro, LucaAmiya, EisukeMadeddu, PaoloAmler, EvzenMadeddu, RobertoAmmendola, SerenaMadeira, Maria H.Amoaku, Winfried M.Madej, DawidAmodeo, PietroMadej, M. GregorAmores, MarcoMaden-Wilkinson, ThomasAmorim, ChristianiMádi, AndrásAmoroso, RosaMadigan, James P.Amoutzias, GrigorisMadka, VenkateshwarAmpofo, EmmanuelMadkour, AichaAmrani, AbdelazizMädler, KathrinAmuzescu, BogdanMadonna, StefaniaAn, Seong Soo A.Madonov, PavelAn, SeungMadrid, EvaAn, Tae KyuMadsen, Claus KroghAn, WonsukMadueño, RafaelAn, YuMadurantakam, Parthasarathy A.Ana María, Rojo LópezMadureira, Patrícia A.Anamthathmakula, PrashanthMaduro, MorrisAnand, SwatiMaeda, AkikoAnandhan, AnnaduraiMaeda, HiroshiAnastasi, Giuseppe PioMaeda, KazuhiroAnastasio, NoelleMaeda, MegumiAnathy, VikasMaeda, YuichiAnbarasu, KumaraswamyMaehre, HanneAncel, JulienMaekawa, RyoAnchordoquy, TomMaekawa, ShunAncuceanu, RobertMaenaka, KatsumiAnderegg, UlfMaestri, ElenaAndersen, Ethan J.Maestú Unturbe, CeferinoAndersen, Thomas LevinMaffei, MargheritaAnderson, CharlesMaftah, AbderrahmanAnderson, Greg M.Magdinier, FrederiqueAnderssion, LeifMaggert, KeithAnderton, RyanMaggi, DavideAndia, IsabelMaggo, SimranAndicsov ´a Eckstein, AnitaMagherini, FrancescaAndl, ThomasMaghuly, FatemehAndón, Fernando TorresMagierowski, MarcinAndrás, IbolyaMagi-Galluzzi, CristinaAndre, HelderMagin, Thomas M.Andreadou, IoannaMagini, AlessandroAndreasson, ErikMagistrato, AlessandraAndrée, BirgitMaglio, CristinaAndreev, Dmitriy NikolaevichMaglio, MelaniaAndreev, Dmitry EMaglio, OrnellaAndreev, KonstantinMagnaghi, ValerioAndré-Grégoire, GwennanMagnaldo, ThierryAndreoli, RobertaMagnani, CorradoAndres-Mach, MartaMagnani, FrancescaAndressen, KjetilMagnani, MauroAndreu, VicenteMagnano Di San Lio, GaetanoAndreuzzi, EvaMagne, DavidAndrew, TedderMagnus, MarcinAndrews, Allison M.Magnusson, Karl-EricAndrews, Rachel NMagrassi, LorenzoAndric, SilvanaMaguire, DonoghAndriessen, AnnekeMahalingaiah, PrathapAndrieux, GeoffroyMahalingam, RamamurthyAndrukhov, OlehMahato, NeelimaAndrzej, NowickiMahavadi, SunilaAneja, RituMahéo, KarineAneskievich, Brian J.Maher, PamelaAnestopoulos, IoannisMahmood, IftekharAnfinogenova, Yana J.Mahmood, KhalidAng, Kok SiongMai, Hans-JörgAng, ZhiweiMaia, CláudioAngastiniotis, MichaelMaidaniuc, AndreeaAngelastro, JamesMaier, CameliaAngeles-Shim, Rosalyn B.Maier, PatrickAngeletti, MauroMaillard, Jean-YvesAngelici, Maria CristinaMaimets, ToivoAngelico, GiuseppeMainardi, MarcoAngelini, FrancescoMainen, Zachary F.Angelino, DonatoMaioli, MargheritaAngelis, Karel J.Maioli, SilviaAngelova, Assia L.Maione, Angela SerenaAngelucci, CristianaMaione, FrancescoAngelucci, EmanueleMaiorano, DomenicoAnger, MartinMaiorano, EugenioAngioni, Maria MaddalenaMaiorino, MatildeAngius, FabrizioMaitarad, PhornphimonAnglada-Huguet, MartaMaitra, RadhashreeAniello, FrancescoMaity, SankarAnis, EmanMaity, ShuvadeepAnisimova, IrinaMaiuolo, JessicaAnita, BoratkóMaixent, Jean MichelAnitas, EugenMaj, MalgorzataAnna, Chlebowska-ŚmigielMajd, NazaninAnna, Lange-ConsiglioMájek, PavelAnnaratone, LauraMajello, BarbaraAnnese, VitoMajer, AnnaAnnesley, SarahMajera, DusanaAnnibale, PaoloMajerova, TatanaAnnibalini, GiosuèMajeský, ĽubošAnraku, MakotoMajewska, AnnaAnsani, LuciaMajewski, LukaszAnsorena, EduardoMaji, TanmoyAntal, DianaMajima, Hideyuki J.Antal, LászlóMajkowska-Skrobek, GrażynaAnticoli, SimonaMajor, IanAntinozzi, CristinaMajor, Ian T.Antipova, VeronicaMajtan, JurajAntognelli, CinziaMajumdar, RajtilakAntoine, Marie-HélèneMajumder, MousumiAnton, LennikovMajumder, PoulamiAntonela Antoniu, SabinaMajumder, PrithaAntonelli, AlessandroMajzoub, AhmadAntonello E Spinelli, Antonello EMak, IvanAntonelou, Marianna H.Mak, Lung-YiAntonio Malfa, GiuseppeMakabe, KokiAntonio, CrovaceMakarem, NourAntonioli, LucaMakarevich, PavelAntonkiewicz, JacekMakarevitch, IrinaAntonogeorgos, GeorgeMakarewicz, WojciechAntony, JaneMakarova, KaterinaAntony, Marie LueMaki, KevinAntosiewicz, Anti-oxidationJędrzejMaki, MasatoshiAntunes, HenedinaMakino, AkikoAnver, ShajahanMakino, HiroshiAnwar, Muhammad AyazMakishima, MakotoAnyfantakis, Dimitrios I.Makiyama, TakeruAoe, TomohikoMaksimovic, IvanaAoki, KosukeMaksymiak, Magdalena MartinkaAoyama, KojiMaksymiuk, Andrew W.Apel, ChristianMakunin, AlexApetrei, ConstantinMakunin, Alexey I.Apicella, AntonioMakvandi, PooyanApostolopoulos, VassoMalacarne, GiuliaApostolova, ElenaMalafa, MokengeAppavu, RajagopalMalafoglia, ValentinaAppels, RudiMalaguarnera, LuciaAprile, AlessioMalaguarnera, MicheleAprile, Francesco A.Malandrakis, EmmanouilAprotosoaie, Ana ClaraMalandrino, AndreaApte, UdayanMalanga, GerardArai, ToshiroMalara, AlessandroAraki, ManabuMalarkannan, SubramaniamAraki, ToshiyukiMalashicheva, AnnaAran, GemmaMalaspina, PatriziaAranda, Carlos J.Malavasi, VeronicaAránega, AmeliaMalcher, AgnieszkaArango Duque, GuillermoMalchik, FyodorAraniciu, CătălinMaldonado-Contreras, AnaArano, YasushiMalemud, Charles J.Arasanz Esteban, HugoMaléth, JózsefArata, ToshiakiMaletínská, LenkaAratani, YasuakiMalewski, TadeuszAraújo, Inês M.Malfa, Giuseppe AntonioAraujo, RicardoMalfatti, Dr. EdoardoArcher, EdwardMalfatti, EdoardoArchibong, AnthonyMalfeito Ferreira, ManuelArcidiacono, BiagioMalhão, FernandaArciello, AngelaMali, VishalArczewska, KatarzynaMalik, Anna R.Ardalan, MaryamMalik, TaimurArdévol, AnnaMalik-Gajewska, MagdalenaArdi-Pastores, VeronicaMalinge, Jean-MarcArechederra, MaríaMalinowska, BarbaraArena, AlessandroMalkemper, PascalArenas, JesúsMalladi, AnishArenas, MiguelMallamace, DomenicoArend, Rebecca C.Mallela, Shamroop KumarArevalo, Juan CarlosMalli, RolandArgentati, ChiaraMalliavin, ThérèseArgentiero, AntonellaMallipeddi, Prema LathaArgento, SergioMallipeddi, SrikrishnanArgueta, Donovan A.Mallis, PanagiotisAriani, AlaricoMallo, NataliaArias Mediano, José LuisMálnási-Csizmadia, AndrásArias-Santiago, SalvadorMaloberti, AlessandroAricò, EleonoraMalouf, CamilleArif, AbulMalpeli, GiorgioArif, TasleemMaltecca, FrancescaArino, JoaquinMalva, João O.Arinstein, ArkadiMaly, PavelArioli, StefaniaMalý, PetrArisaka, YoshinoriMalyarenko, Timofey V.Arita, MasanoriMałysz - Cymborska, IzabelaAriyasu, DaisukeMan, SimonaAriyoshi, WataruManakhov, AntonAriza, Adolfo J.Manaskova-Postlerova, PavlaArizza, VincenzoManavalan, BalachandranArkhipov, Vladimir I.Manca, GabrieleArmato, UbaldoManca, Maria LetiziaArmellini, EliaManchia, MirkoArmeni, TatianaMancinelli, RominaArmiento, Angela RitaMancini, AnnamariaArmstead, William M.Mancini, MaicolArmstrong, HeatherMancini, Stéphane J. C.Arnao, Marino BañónManco, LicínioArnesano, FabioMancuso, FrancescaArnhold, JuergenMancuso, SalvatriceArnhold, JürgenManda, GinaArnoult, DamienMandal, AmritlalArock, MichelMandal, Mihir KumarAroni, SoniaMandal, MrinmayArora, GunjanMandalà, MaurizioArosio, PaoloMändar, ReetArranz, AmaiaMandard, StéphaneArrebola, Francisco A.Mandava, Nagabhushanam BhushanArrigoni, ChiaraMandelli, DavideArroo, RandolphMandoki, MiraArruda-Carvalho, MaitheMandrioli, RobertoArshad, NajlaMandyam, ChitraArtalejo, Antonio RManenti, AntonioArteel, Gavin E.Manera, MaurizioArtero, RubenManfredi, Kirk P.Artés-Hernández, FranciscoMang, Stefania MirelaArtín-Aragón, Sagrario MMangalagiu, IonelArtuch, RafaMangani, StefanoArtur, Mariana Aline SilvaMangano, KatiaArtyszak, ArkadiuszMangaonkar, AbhishekAruanno, A.Mangiagalli, MarcoArumäe, UrmasMangino, GiorgioAryal, RishiMango, LucioAryal, Uma KMangogna, AlessandroAryan, LailaMani, GovindasamyAsada, KenMani, SridharAsada, MasahitoManiam, SubashaniAsadipooya, KamyarManickavelu, AlaguAsano, AtsushiMankin, AlexanderAsano, NaokiManley, Sharon J.Asaro, FiorettaManna, IdaAsatryan, LianaMannell, HannaAscoli Marchetti, AndreaMannello, FerdinandoAseervatham, JayaMänner, JörgAsfor, AminMannik, JaanAsgharian, HosseinaliMannini, BenedettaAshida, HitoshiMansier, OlivierAshihara, EishiMansky, Kim C.Ashley, JonMansour, SaharAshley, ZoeMånsson, AlfAshour, Mohamed LotfyManstein, DietmarAshour, Mohamed-BassemMansuri, ShahidAshpole, Nicole M.Mantadakis, ElpisAshraf, Ghulam MdMantareva, Vanya N.Ashton, MelanieManti, SaraAsimaki, AngelikiManto, MarioAsimakopoulos, AnastasiaMantovani, AlbertoAsimakopoulos, ByronMantripragada, Venkata P.Askes, SvenManukhina, EugeniaAskjaer, PeterManukjan, GeorgiAslam, MuhammadManville, RianAspenström, PontusManzo-Merino, JoaquinAsquith, Christopher R. M.Manzotti, GloriaAssaf, Jean-ClaudeMao, XiaoboAssenza, GiovanniMapelli, LisaAssirelli, ElisaMaple, PeterAstolfi, AndreaMaqoud, FatimaAstolfi, MariaMaraldi, TulliaAsturiano, Juan F.Maramai, SamueleAszodi, AttilaMarampon, FrancescoAtabai, KamranMaran, UkoAtawia, Reem T.Maranesi, MargheritaAthinarayanan, ShaminieMarascio, NadiaAthyros, VasiliosMarasco, DanielaAtkin, Stephen L.Marasek-Ciolakowska, AgnieszkaAtlante, AnnaMarassi, FrancescaAtlasz, TamasMarazziti, DanielaAtobe, MasakazuMarć, Małgorzata AnnaAtochin, DmitriyMarcel, VirginieAtrei, AndreaMarcelli, Augusto ClaudioAtsushi, MatsumuraMarcenaro, EmanuelaAttanasio, ChiaraMarchand, ChristopheAtteritano, MarcoMarchand, Patrice A.Attia, Mohamed F. Marchese, AdrianoAttila, FehérMarchese, CinziaAttili, Anna RitaMarchese, EnricoAttri, PankajMarchese, MariaAtwal, Paldeep SMarchesi, IsabellaAubert, EmmanuelMarchetti, FabioAubert, GregoryMarchetti, PhilippeAuburger, GeorgMarchetti, SandrineAuclair, KarineMarchiani, SaraAudet-Walsh, ÉtienneMarchini, AntonioAudi, SaidMarchini, CristinaAuguet, TeresaMarchiò, CaterinaAugustin, HellmutMarcilla, AntonioAunapuu, MarinaMarcinkowska, EwaAung, Meiji SoeMarco, FranciscoAureli, MassimoMarco-Contelles, JoséAurrand-Lions, MichelMarco-Contelles, Jose LuisAushev, VasilyMarcon, CarolineAussenac, ThierryMarconi, GiovanniAustin, RachelMarconi, Guya DilettaAutelli, RiccardoMarcos, Jose F.Autry, JosephMarcu, LoredanaAvalle, LidiaMarcus, WiederAvdeeva, VarvaraMarcuzzo, StefaniaAvelange-Macherel, Marie-HélèneMaréchal, Jean-DidierAvidor-Reiss, TomerMarečková, KláraAvignone, ElenaMarei, HanyÁvila, JulioMarek, KieliszekAvila, MatiasMarek-Trzonkowska, NataliaÁvila-Flores, AntoniaMarengo, BarbaraAviles, Francesc XavierMareri, LaviniaAvin, VijayMaresca, VittoriaAvino, PasqualeMareschi, KatiaAvramova, ViktoriyaMaret, WolfgangAwasthi, MayankaMaretzky, ThorstenAwasthi, PraveenMargaglione, MaurizioAwatade, Nikhil TMargaritis, LukasAwate, SanketMargassery, Lekha MenonAwayda, MouhamedMargheritis, EleonoraAyabe, TatsuhiroMargiotta, AzzurraAyayee, Paul AkwetteyMargulies, BarryAyaz, AhmetMarí, MontserratAydar, EbruMaria Angela, AngelaAylett, Christopher H.S.Maria Carmela, Bonaccorsi Di PattiAyuso, José MaríaMaria Irene, BelliniAzad, Obyedul KalamMaria Musarella, CarmeloAzad, TahaMaría Rosaura, Leis TrabazoAzam, Aa HaerumanMariadason, JohnAzarin, Kirill VitalievichMarian, BrigitteAzarov, Jan E.Mariano, CaratozzoloAzer, Samy A.Mariconda, AnnaluisaAzevedo, AndreiaMariggiò, Maria AddolorataAzevedo, HerlanderMarijuán, PedroAzevedo, JorgeMarín, ClaraAzevedo, Maria ManuelMarin, Jose J.G.Azimzadeh, OmidMarin, LuminitaAzuma, ChiekoMarín, MacarenaAzuma, KazuoMarincs, FerencAzushima, KengoMarinelli, EnricoBaaße, AnnemarieMarini, FrancescaBaba, HirokoMarini, MarinaBaba, TsukasaMarino Gammazza, AntonellaBabes, AlexandruMarino, AngelaBabichev, SergiiMarino, FrancaBabickova, JankaMarino, JosephBabii, OlegMarino, TizianaBabina, MagdaMariot, VirginieBabu, AnishMariotto, ElenaBabu, DineshMariscalco, GiovanniBaburamani, Ana A.Mariuzza, Roy A.Baccarani, UmbertoMarkey, Michael P.Bacchus-Montabonel, Marie-ChristineMarkiewicz, AleksandraBacci, Maria LauraMarkkanen, EnniBacci, StefanoMarkopoulos, AnastasiosBacete, LauraMarkopoulos, Georgios S.Bacher, UlrikeMarkopoulou, OlgaBachrach, GiladMarkova, SvetlanaBachy, Christine MMarkova-Car, Elitza P.Baci, DenisaMarkowicz-Piasecka, MagdalenaBacker, Ronald A.Markowitz, JosephBacolla, AlbinoMarks, HendrikBacso, ZsoltMarks, Jeremy D.Baczkó, IstvánMarkus, Waldeck-WeiermairBadadani, MallikarjunMarkwardt, FritzBadaeva, EkaterinaMarlicz, WojciechBadawi, MichaelMarminon, ChristelleBadawy, AbdullaMármol, InesBadea, NicoletaMaron, Steven B.Badea, Tudor ConstantinMarongiu, MaraBade-Doeding, ChristinaMaroni, PaolaBadhe, RavindraMarote, AnaBadía, RogerMarotte, HubertBadino, PaolaMarques, IsabelBado, IgorMarques, João GamaBadowski, MichaelMarques, NatáliaBadran, AhmedMarques, TaniaBadrising, UmeshMarquez, EdgarBae, Hae-RahnMarra, AlbertoBae, In HwaMarracci, SilviaBae, SangsuMarrano, NicolaBaek, Jeong-HwaMarrocchi, AssuntaBaek, Jong-SuepMarrone, GiuseppeBaer, Patrick C.Marrone, OresteBaeza, AlejandroMarsano, René MassimilianoBagal-Kestwal, Dipali R.Marsat, GaryBagchi, DebasisMarschall, Hanns-UlrichBaghbaderani, Behnam AhmadianMarschall, ManfredBaglivo, IlariaMarsolais, FredericBaglole, CarolynMarston, DeniseBagniewska-Zadworna, AgnieszkaMarston, Steven B.Bagnoli, PaolaMartău, Adrian GheorgheBagyinszky, EvaMartel, BernardBahal, RamanMartel, PauloBahls, MartinMartell, KevinBahn, AndrewMartella, DanieleBai, RenrenMartella, ElisaBai, ShiMartella, GiuseppinaBaietti, Maria FrancescaMartelli, Alberto MariaBaig, Mohammad HassanMartelli, FabioBailey, Adam L.Martens, GerardBailey, Charles GMartens, JürgenBailey, ErnestMartens, StefanBailly, ChristianMarteyn, AntoineBaino, FrancescoMartí, JoaquínBairaktari, EleniMarti, MatteoBaizabal, José ManuelMartí, SergioBaj, AndreinaMartial, SoniaBaj, JacekMartikainen, RiikkaBajaj, JeevishaMartin Galan, AidaBajaj, RuchikaMartin Miguel, Casco RoblesBajan, SarahMartin, Andrew V.Bajda, TomaszMartín, CarmenBajek, AnnaMartin, DanielBajgain, PrabinMartin, Elizabeth M.Bajguz, AndrzejMartin, FranckBajinskis, AinarsMartin, KeithBajpai, VivekMartin, KrssakBajusz, DávidMartín, MargaritaBajusz, IzabellaMartin, MariaBajwa, AmandeepMartin, Olivier C.Bak, AndrzejMartin, PatriciaBakaeva, ZandaMartin, RuthBakarčić, DankoMartin, SallyBaker, BradleyMartin, Tammy M.Baker, Brent A.Martin, TiphaineBakht, MartinMartina, MatteoBakhti, MostafaMartín-Antonio, BeatrizBakillah, AhmedMartín-Aragón, SagrarioBakker, HansMartín-Cano, Francisco EduardoBakloushinskaya, IrinaMartín-de-Llano, José JavierBakos, JanMartinelli, ChiaraBalabanova, LarissaMartinelli, GiovanniBalacescu, OvidiuMartinez Alonso, MartaBalaguer, PatrickMartinez Calejman, CamilaBalaj, LeonoraMartínez De Pablos, RocíoBalakirev, Evgeniy S.Martínez Iglesias, OlaiaBalalaeva, Irina V.Martinez, AgustinBalan, PrabhuMartínez, AlbertoBalan, SreeKumarMartínez, Carlos M.Balandin, Alexander A.Martinez, ClaudiaBalao, FranciscoMartínez, ConstantinoBalaphas, AlexandreMartínez, IsidoroBalaraman, VelmuruganMartinez, Jose CBalas, MihaelaMartinez, Luis A.Balashova, NataliyaMartinez, ManuelBalázs, VargaMartínez, MartaBalbach, MelanieMartínez, Miguel A.Balcar, VladimirMartínez-Abarca, FranciscoBalcerczyk, AnetaMartínez-Arnau, Francisco MiguelBaldanzi, GianlucaMartínez-Banaclocha, Marcos ArturoBaldassarre, Maria ElisabettaMartínez-Cifuentes, MaximilianoBaldi, ElisabettaMartinez-de-Tejada, GuillermoBaldini, EnkeMartínez-Espinosa, Rosa MaríaBaldwin, Albert S.Martínez-Esteso, María JoséBalestra, DarioMartínez-Galiano, Juan MiguelBalestrini, Raffaella MariaMartínez-García, ManuelBalestrini, SimonaMartínez-Gómez, PedroBalkan, Wayne W.Martinez-Guryn, KristinaBalla, JozsefMartinez-Hervas, SergioBalland, EglantineMartinez-Martos, Jose ManuelBallaz, Santiago J.Martinez-Moreno, CarlosBallegeer, MarliesMartinez-Navarrete, GemaBallester, Ana-rosaMartinez-Pastor, FelipeBallini, AndreaMartínez-Rodríguez, AlejandroBallouz, SaraMartínez-Rodríguez, SergioBalmer, LoisMartínez-Salas, EncarnaciónBalne, Praveen KumarMartínez-Sánchez, NoeliaBaloni, PriyankaMartinez-Useros, JavierBalsano, ClaraMartín-Hidalgo, DavidBalsinde, JesusMartini, ClaudiaBaltriukiene, DaivaMartinka, MichalBamforth, SimonMartínková, LudmilaBan, BhupalMartin-Lopez, EduardoBan, YusukeMartín-Molina, AlbertoBanach, ArturMartín-Montalvo, AlejandroBanach, MateuszMartín-Nieto, JoséBanadyga, LoganMartino, Piera AnnaBanaś, AntoniMartinotti, SimonaBanaszak, SzymonMartín-Peláez, SandraBandapalli, Obul ReddyMartín-Rodríguez, Alberto J.Bandopadhyay, RinaMartins Lima, AugustoBanegas Luna, Antonio JesúsMartins, Antonio HenriqueBanerjee, AbhinandanMartins, FilipaBanerjee, AnweshaMartins, IanBanerjee, AparajitaMartins, José A.Banerjee, Priya R.Martins, Teresa VazBanerji, VershaMartín-Sánchez, EsperanzaBanfi, GiuseppeMartín-Satué, MireiaBank, IlanMartinuzzi, AndreaBanke, Tue G.Martín-Valero, María JesúsBankova, VassyaMartín-Vasallo, PabloBanks, WilliamMarto, Carlos MiguelBanning, AntjeMartorana, AlessandroBanno, MasahiroMartu, Maria AlexandraBanoub, Joseph H.Martz, FrancoiseBansal, AdityaMaruani, AntoineBansal, Kuldeep K.Marumo, TakeshiBanta, LoisMarunaka, YoshinoriBanti, Christina N.Maruyama, IchiroBao, Jun-XiangMaruyama, KeiBao, YongpingMarwitz, SebastianBaptista, Pedro VianaMarx, FlorentineBär, ChristianMarx, Gerald R. Bär, SéverineMarx, Joannes J. M.Barabasz, WieslawMarycz, KrzysztofBarabutis, NektariosMarzano, FlavianaBarajas-Martínez, H., Marzec, MichalBarak, BoazMarzocco, StefaniaBarakat, Tahsin StefanMarzoli, FrancescaBarakate, AbdellahMasahiro, SatoBaralle, MarcoMasaki, TakayukiBaran, JaroslawMasana, LuisBaran, WojciechMasarone, DanieleBarancik, MiroslavMasatake, KanaiBaranova, EkaterinaMascarenhas, MariolaBaranowska-Bosiacka, IrenaMascellino, Maria TeresaBarash, DannyMasciarelli, SilviaBarashkov, Nikolay A.Mascini, MacelloBarazzuol, LaraMaserti, Bianca ElenaBarbagallo, DavideMás-Estellés, J.Barbalho, Sandra MariaMashiguchi, KiyoshiBarbanti-Bròdano, GiovanniMasi, AnnalisaBarbara, BreznikMasli, SharmilaBarbas, João PedroMaslow, Joel N.Barbati, CristianaMason, NigelBarbault, FlorentMasonbrink, RickBarbe, Mary F.Masquida, BenoitBarberis, ElettraMassa, AnnamariaBarberis, MassimoMassa, DanieleBarbero, FrancescaMassa, ValentinaBarbier, JulienMassad, Salwa G.Barbieri, AntonioMassai, RossanoBarbieri, MichelangelaMassardier, ValérieBarbieri, Silvia S.Massaro, MarikaBarbonetti, ArcangeloMassimiliano, CordaroBarbosa, Flávia LeãoMassimini, MarcellaBarca, AmilcareMassimino Cocuzza, GiuseppeBarcaccia, GianniMassing, UlrichBarcellini, WilmaMassotte, DominiqueBarcia, CarlosMassoud, SalahBardají, EduardMasternak, JoannaBarding, Gregory A.Masters, Colin L.Bardova, KristinaMastinu, AndreaBardyn, ManonMastrangelo, Anna M.Bareja, AkshayMastrangelo, StefanoBari, EliaMastroleo, FeliceBarichello, TatianaMastronuzzi, AngelaBarik, SailenMasuda, KiyoshiBarillari, GiovanniMasuda, MariBaritugo, Kei AnneMasuda, TakaakiBarkan, DanielMasuda, TatsuruBarker, TylerMasui, KentaBarki-Harrington, LizaMasullo, MariorosarioBarkleit, AstridMasutani, MitsukoBarlier, AnneMasuyer, GeoffreyBarlocco, DanielaMatacchione, GiuliaBarlow, JacquelineMatafome, PauloBarlow, Linda A.Matarredona, Esperanza R.Barna, BalazsMatarrese, PaolaBarna, JánosMaté, BélenBarnabei, AgneseMatei, AlinaBarnard, MollieMatei, CristianBarnes, JarrodMatencio, AdriánBaron, ByronMatera, CarloBaroncini, DamianoMateus, Maria L.Barone, InesMathew, ShibinBar-Or, DavidMathias, ClintonBarosi, GiovanniMatiadis, DimitriosBarragán, IsabelMatiadis, DimitrisBarral, DuarteMatias, Pedro M.Barras, FrédéricMatikonda, SiddharthBarreca, DavideMatilla, Angel J.Barreiro Iglesias, AntónMatilla, MiguelBarrero, María JoséMatlawska-Wasowska, KseniaBarro, FranciscoMatoba, KeiichiroBarroso, MadalenaMatoba, ShogoBarro-Soria, ReneMatos, ManuelaBarrott, JaredMatosiuk, DariuszBarta, CsengeleMatoušek, JaroslavBartáková, VendulaMatraszek-Gawron, RenataBarteková, MonikaMATRICARDI, PietroBartel, SabineMatrone, CarmelaBartels, DorotheaMatschke, JohannBartelt, AlexanderMatsubara, KazukiBarth, BrianMatsubara, KeiichiBartholomeeusen, Dr. KoenMatsubara, ShigekiBartnik, EwaMatsuda, HisashiBartok, EvaMatsuda, KoichiBartoli, ManuelaMatsuda, TomokiBartollino, SilviaMatsuda, YudaiBartolome, FernandoMatsugaki, AiraBarton, SamanthaMatsui, AkihiroBartosik, Aneta AgnieszkaMatsui, HidenoriBartosz, GrzegorzMatsumoto, KenBartoszek, AgnieszkaMatsumoto, TakayukiBartoszewski, RafalMatsumoto, YukihiroBartova, EvaMatsumura, KiyoshiBartuzi, DamianMatsumura, ShigeruBarutcu, RasimMatsumura, TsuyoshiBarvik, IvanMatsunami, MasatoshiBarwiolek, MagdalenaMatsuo, KoichiBarygin, Oleg I.Matsuo, MuneakiBarzilay, JoshuaMatsuo, TakuyaBar-Ziv, RazMatsuo, TatsuhitoBar-Zvi, DudyMatsuo, YasumitsuBaś, BogusławMatsuoka, DaisukeBasak, IndranilMatsuoka, IsaoBasalay, MarynaMatsushita, TakuBasar, MuratMatsuura, BunzoBasetti, MadhuMatsuya, YusukeBashkin, James K.Matsuzaki, ShinyaBashtrykov, PavelMatsuzaki, ToshiyukiBashur, ChristopherMatta, CsabaBasile, TeodoraMattapally, SaiduluBasit, AbdulMatte, AlessandroBaslam, MarouaneMattei, CésarBasnet, Ram ManoharMattei, FabrizioBass, AndrewMatter, KarlBaßler, JochenMatteucci, ClaudiaBassolé, Imaël Henri NestorMatthew, BeneschBastmeyer, MartinMatthiesen, RuneBasu, ReetobrataMattii, LetiziaBasu, RupsaMattila, SariBasu, SupratimMattioli, Anna VittoriaBaswan, SudhirMattioli-Belmonte, MonicaBatchu, RameshMattison, Chris P.Bateman, Joesph MatthewMattoo, AutarBates, John T.Mattoscio, DomenicoBathula, Chandra SekharMattsson, Mats-OlofBatish, MonaMatucci-Cerinic, MarcoBatool, MariaMatulić, MajaBatorova, AngelikaMaturana, Andrés DanielBatta, KiranMaturana, Carola J.Battaglia, EricMatusik, PawełBattaglia, RosaliaMatwijczuk, ArkadiuszBattista, SabrinaMátyás, GáborBattistelli, CeciliaMatynia, AnnaBattistelli, MichelaMatysik, SilkeBattistini, ChiaraMaue, Robert A.Battula, NarendraMaugeri, GraziaBaucum, Anthony J.Mauhin, WladimirBaud, StephanieMaurer, BarbaraBaudier, JacquesMaurer, JochenBaudoin-Dehoux, CécileMauri, EmanueleBauer, JessicaMauriello, AlessandroBaugh, J. A.Mauriello, EmiliaBaulin, Vladimir AMaurizio, MartiniBaulina, NataliaMauro, Adolfo GabrieleBaumann, MarcusMauro, AnnunziataBaumann, UlrichMaury, StéphaneBaumgarner, Bradley L.Maurya, ShailendraBaumgart, Simon J.Maver, TinaBaumgarten, MartinMaves, LisaBaumgarten, ThomasMavridis, KonstantinosBaumgartner, UlrichMavrogeni, SophieBaumstark-Khan, ChristaMavrogonatou, EleniBauser-Heaton, HollyMavropoulos, AthanasiosBautista, RocíoMawhinney, ThomasBawadekar, MandarMaxim, AurelBawage, SwapnilMaxwell, Jessie R.Bax, Bridget E.May, Kerrie L.Baxter, Victoria K.Mayan, Maria D.Bay, Denice C.Maybeck, VanessaBay, PeterMayer, RupertBayer, ThomasMayer, ThomasBayer-Skoff, Sarah M.Mayerhofer, ArturBaykov, AlexanderMaynard, JenniferBayona-Bafaluy, María PilarMayr, Johannes A.Bazán, EulaliaMazan, SylvieBazan-Socha, StanislawaMazerska, ZofiaBazhanova, Elena DMazierski, PawełBazhenov, ViacheslavMazilu, LauraBazihizina, NadiaMazin, PavelBazil, JasonMazor, OhadBazrafshan, ZahraMazur, Antonina JoannaBazwinsky-Wutschke, IvonneMazur-Bialy, AgnieszkaBazylińska, UrszulaMazur-Marzec, HannaBear, ChristineMazurov, Dmitriy VBeard, MatthewMazzanti, MicheleBeasley, ShannonMazzeo, GiuseppeBeaumelle, BrunoMazzeo, Maria FiorellaBeauvais, Wendy A.Mazzoni, MariaBeaven, Anne W.Mazzucotelli, ElisabettaBeavis, PaulMazzuoli-Weber, GemmaBebelman, Maarten P.Mbagwu, Smart IkechukwuBeberok, ArturMc Auley, Mark T.Becciolini, AndreaMcArdle, StephanieBecerra, ManuelMcBride, Devin W.Beck, BenjaminMcCann, Liza J. Beck, Hans ChristianMcCann, Matthew R.Beck, Michael W.McCarthy, JohnBeckel, J. M.McCarthy, MichaelBecker, Daniel P.McCartney, FionaBecker, SusanneMccarty, Nael A.Becker, ThomasMcClung, C. RobertsonBecker, YvonneMcCluskey, LynnetteBecker-Pauly, ChristophMcCombe, PamelaBecq, FredricMcCorvy, John D.Becucci, LuciaMccown, PhillipBedi, DeepaMcCoy, AnnetteBedia, CarmenMcCoy, Sara S.Bedini, EmilianoMcCubbin, AndrewBedini, GianniMcDaneld, Tara GBednarek, RadoslawMcDaneld, Tara G.Bednarski, Patrick J.McDermott, SuzanneBednarz-Knoll, NataliaMcDevitt, ChristopherBedon, FrankMcDonald, DeniseBedoyan, Jirair KMcDonnell, AlisonBeenakker, Jan-WillemMcdonnell, ThomasBeesley, JonathanMcEwan, IainBeetz, ChristianMcGee-Lawrence, MeghanBegcy, KevinMcGill, JodiBegemann, MatthiasMcGill, Mitchell R.Beger, RichardMcgilvray, Kirk CBeghini, AlessandroMcGregor, NeilBeguelin, WendyMcGrew, MikeBehera, SmrutisanjitaMcIntosh, RobertBehling, FelixMcKay, BrianBehr, MarcMcKay, TinaBehrens, JürgenMcKenna, Joseph F.Behrens, Sven-ErikMcKenzie, Brienne A.Behringer, ErikMcKeown-Longo, Paula J.Behzadi, PayamMcKillop, A. M.Beilby, MaryMcKinstry-Wu, AndrewBeirowski, BogdanMcLaren, James E.Beissert, TimMcLaurin, JoAnneBejarano, IgnacioMcLean, GaryBekeschus, SanderMcManus, KirkBekiari, VlasoulaMcMillin, MatthewBekri, SoumeyaMcMurray, Cynthia T.Belayew, AlexandraMcNally, Martin A.Belda-Palazón, BorjaMcPeek, AshleyBelgiovine, CristinaMcPhillie, MartinBelin De Chantemèle, Eric J.McQuade, RachelBelizario, José ErnestoMcRobb, Lucinda SBelizna, CristinaMeade, AidanBell, JohnMeana Paneda, Rubenbell, StephenMeana, ClaraBellan, MattiaMears, JasonBellanti, FrancescoMechoulam, RaphaelBellardita, CarmeloMechrez, GuyBellavia, DanieleMechulam, YvesBellei, BarbaraMęczyńska-Wielgosz, SylwiaBellelli, RobertoMeder, LydiaBellemin-Laponnaz, StéphaneMediero, AránzazuBellien, JeremyMedina, DavidBellier, Jean PierreMedina, DiegoBellini, Maria IreneMedina, MilagrosBello, Nicholas T.Medina, Reinhold J.Bello, XabierMedina-Kauwe, Lali K.Bello-Espinosa, Luis E.Medina-Ramírez, IlianaBellomo, FrancescoMedina-Rodríguez, Eva M.Bellucci, StefanoMediouni, SoniaBelluti, SilviaMedová, MichaelaBeloborodova, Natalia V.Medronho, Bruno Filipe FigueirasBelosludtsev, KonstantinMedvecz, MártaBeloukas, ApostolosMedvedev, Ilya NikolaevichBeltowski, JerzyMeegan, Jamie E.Bemporad, FrancescoMeerson, AriBenabdellah, KarimMegaw, RolyBenada, JanMège, René-MarcBenamar, KhalidMehaffy, CarolinaBenarafa, CharafMehariya, SanjeetBenarba, BachirMehdi, EbrahimiBenavente, Claudia AMehla, JogenderBenavides-Mendoza, AdalbertoMehlmann, LisaBenbrook, Doris MangiaracinaMehlotra, Rajeev K.Bencharit, SompopMehrabadi, MohammadBencivenga, StefanoMehrazma, BanafshehBencsik, PeterMehrpour, MaryamBendahhou, SaïdMehta, Gaurav A.Ben-David, YaacovMehta, GautamBende, AttilaMehta, GunjanBender, KevinMéhul, BrunoBendokas, VidmantasMei, Kuo-ChingBenedetti, ElisabettaMei, ShenglinBenedetti, FrancescaMei, Ya-FangBenedetti, MicheleMei, YangBenedetti, RosariaMeier, CarolaBenedetto, AlexandreMeierhofer, DavidBenelli, RobertoMeijer, Harold J GBenetti, ElisaMeijers, BjörnBenfaremo, DevisMeijers, WouterBenfeito, Sofia EsterMeijles, Daniel N.Benfenati, EmilioMeimberg, HaraldBenhelli, HoudaMeimoun, PatriceBenistant, ChristineMeinander, AnnikaBenítez Jiménez, José JesúsMeinhardt, MatthiasBennet, AndrewMeiri, HamutalBennett, Lea D.Meisel, Hans-JörgBennevault-Celton, VéroniqueMejuto, JuanBennewitz, Margaret F.Meksem, KhalidBenoit, ChassaingMelas, MarilenaBenoit, MatthiasMelcher, KarstenBenowitz, Kyle MMelchor, JuanBenrick, AnnaMeldolesi, JacopoBentel, Jacqueline M.Mele, AndreaBentley, T. WilliamMeleady, PaulaBenussi, AlbertoMeléndez-Hevia, EnriqueBenveniste, HeleneMelgar, SilviaBenyhe, SandorMeli, VijaykumarBenz, RolandMellett, MarkBen-Ze’ev, AvriMellidou, IfigeneiaBenzoni, PatriziaMelnik, DanielaBerardesca, EnzoMelo, Bernardete F.Berardi, Anna ConcettaMelo, Sonia A.Berardi, EmanueleMeloni, BrunoBerardo, ClarissaMeloto, Carolina BeraldoBerchtold, Martin W.Melrose, JamesBeretta, Giovanni L.Melve, Guro KristinBerezhnaya, ElenaMenasché, PhilippeBerezovski, Maxim V.Mencarelli, MonicaBerezovsky, IgorMendes, Alexandrina FerreiraBerg, Matthew D.Mendes, Livia P.Bergamo, AlbertaMendes, Marta V.Bergandi, LoredanaMéndez, AnaBerg-Beckhoff, GabrieleMéndez-Barbero, NereaBergdahl, AndreasMendonça, MarceloBergen, ArthurMenegazzi, MartaBerger, BeatriceMeneses, CarlosBerger, SlavaMeneses, Maria JoãoBerger, WolfgangMenezes, José C. J. M. D. S. Bergero, RobertaMenezes, Miriam-RoseBergholz, TeresaMenezo, YvesBergmann, CorneliaMeng, DongBergougnoux, VéroniqueMeng, HuanxinBergquist, RobertMeng, JinhongBeringue, VincentMeng, QinghangBeristain, AlexanderMeng, QingshiBerka, KarelMengel, DavidBerkefeld, AndreasMenin, ChiaraBerlanda, NicolaMenke, AndréBerlec, AlešMenon, Manoj B.Berlin, K. DarrellMensah-Nyagam, GuyBermudez, MarcelMenziani, Maria CristinaBernacchia, GiovanniMeola, GiovanniBernal, VicenteMeraviglia, VivianaBernal-Lopez, M. RosaMercati, FrancescoBernard, AmélieMercedes, GimenoBernard, EmilienMerches, KatjaBernard, Marek K.Merdes, AndreasBernardes, NunoMerfort, IrmgardBernardi, SimonaMergeay, MaxBernardini, AndreaMergler, StefanBernardino, Raquel L.Merhi, AhmadBernardo, AntoniettaMerida, IsabelBernasconi, PiaMerigo, FlaviaBernatchez, Jean A.Merino, BeatrizBernatík, OndřejMerino, Jaime M.Bernaudin, Jean-FrançoisMerlot, ElodieBerndt, Michael C.Meroni, MaricaBernhardt, AnneMerrick, DanielBerni, RobertoMertens, DanielBernitsas, EvanthiaMertlíková-Kaiserová, HelenaBernocchi, GraziellaMertz, Janet E.Bernstein, Hans-GertMerz, Alexey J.Bersano, AnnaMesaros, Clementina A.Bertacchini, JessikaMesaros, EugenBertea, CinziaMeshram, ChetanBerthelot, LaurelineMešić, AleksandarBertho, Jean-MarcMeškys, RolandasBertho, NicolasMessina, SamanthaBertin, SamuelMessori, LuigiBertok, SaraMestan, KarenBertoldi, MariaritaMester, Patrick-JulianBertoli, AlessandroMesuraca, MariaBertorelli, RosaliaMetwally, MayadaBesalú, EmiliMetzger, Dennis W.Besharat, Zein MersiniMetzger, JochenBessa, JoseMetzger, Monika L.Besse, LenkaMetzler, RebeccaBesser, MartinMeurer, Steffen KlausBessette, BarbaraMeurette, OlivierBessmertnykh-Lemeune, AllaMevissen, TychoBesson, ThierryMeyer Zu Heringdorf, DagmarBesteiro, SébastienMeyer, MichelBettache, NadirMeyer, Ralph GBettotti, PaoloMeyer-Ficca, Mirella L.Betzel, ChristianMező, GáborBevilacqua, ArturoMezzasoma, LetiziaBeyeh, Ngong KodiahMezzomo Collares, FabrícioBeyer, Eric C.Mia, SobujBeyersdorf, FriedhelmMiao, LinglingBeznosková, PetraMiazek, ArkadiuszBEZU, LucilliaMicale, NicolaBharadwaj, VivekMiceli, AlessandroBharaj, PreetiMiceli, VitaleBharath, Leena P.Michael, AdrianBhardwaj, GouravMichaelis, MartinBhardwaj, RajeshMichaelson, DanielBhat, JaydeepMichaeu, OlivierBhat, KamakotiMichal, KohoutBhat, NehaMichalak, MarcinBhat, OwaisMichalke, BernhardBhati, Kaushal KumarMichalopoulos, EfstathiosBhatia, DivyaMichard, ErwanBhatia, Harsharan S.Michel, Jean-baptisteBhattacharjee, Apurba KrishnaMichel, Martin ChristianBhattacharjee, MrinalMichel, MaximilianBhattacharjee, SonaliMichel, PiotrBhattacharya, AbhisekMichel, SebastianBhattacharya, AnindyaMichelacci, Yara M.Bhattacharya, KaushikMicheletti, GabrieleBhattacharya, SupriyoMicheletti, RudiBhattacharyya, PiyaliMichelhaugh, Sharon KayBhattacharyya, SanchariMichelle, PalmieriBhattacharyya, TapanMichels, AlexanderBhattarai, AjayaMichelucci, AlessandroBhattarai, GehendraMichelucci, AntonioBhattarai, Kashi RajMichetti, FabrizioBhattarai, KrishnaMichiels, ChrisBhatti, Fazal-ur-RehmanMichinaga, ShotaroBhawal, Ujjal KumarMichl, PatrickBhetwal, Bhupal P.Micillo, RaffaellaBhuckory, ShashiMicucci, MatteoBhullar, RajMiddei, SilviaBhusal, RamMiękus, NataliaBhusal, Siddhi JeewanMielańczyk, AnnaBi, Andy ChengfengMielcarek, MichalBi, MingjunMiele, Adriana EricaBi, XinMielgo-Ayuso, JuanBiagioni, AlessioMierek-Adamska, AgnieszkaBiagioni, Audrey FranceschiMierzwiński, JózefBialecka, MonikaMifsud, KarenBiałek, AgnieszkaMigdalis, IliasBialonska, DobroslawaMiggiano, RiccardoBiałowąs, JacekMigita, SatoshiBiamonti, GiuseppeMiglinas, MariusBianchi, AntonioMigliore, AlbertoBianchi, FabrizioMigliori, EdoardoBianchi, FrancescaMignatti, PaoloBianchi, VittorioMiguel, CeliaBianco, AndreaMiguel, Maria Da Graça CostaBianco, FedericoMíguez, Jesús M.Bianco, PieroMihály, BorosBianco, SimonaMihály, JudithBiancolella, MichelaMihara, KeichiroBiancolillo, AlessandraMihola, OndrejBiasucci, GiacomoMikaelyan, Arsen S.Bichet, Daniel G.Mikaia, AnzorBidault, GuillaumeMikala, GaborBieber, MichaelMikami, KojiBiedermann, ThomasMikami, YoshinoriBiehl, ChristophMikecz, KatalinBielas, RafałMikelis, ConstantinosBielas, WiesławMiki, HiroakiBielczyk-Maczyńska, EwaMiko, EditBielfeldt, StephanMikołajczak, KrzysztofBienkiewicz, Ewa AnnaMikstacka, RenataBienvenut, WillyMikula, MarioBierle, CraigMikulski, DawidBiernasiuk, AnnaMikuš, PeterBiersack, BernhardMilanese, MarcoBijlsma, Maarten F.Milano, GiuseppinaBikbova, GuzelMilano, MarianneBilancio, AntonioMilano, SerenaBilbao, DanielMilanova, AneliyaBilewicz, AleksanderMilardi, DaniloBilger, AndreaMilatz, SusanneBilic-Ćurčić, InesMilavetz, BarryBilińska, Zofia T.Milc, JustynaBillard, Jean MarieMilcarek, ChristineBilliald, PhilippeMilczarek, MałgorzataBillingsley, Hayley E.Milczarski, PawelBillmyre, BlakeMildner, MichaelBilska-Kos, AnnaMilella, LuigiBilska-Wilkosz, AnnaMilenkovic, DraganBin, Bum-HoMilenkovic, Vladimir M.Binhi, Vladimir N.Miles, Wayne OwenBink, Diewertje IMilescu, LorinBinley, JamesMiletín, MiroslavBiondi, AntonioMiletta, MariaBiondi, StefaniaMilhano Santos, FátimaBiondo, Luana AmorimMilhinhos, AnaBiosca, Elena G.Militello, GiuseppeBiressi, StefanoMiljković, Miloš D.Birgegård, GunnarMillar, AnnBirket, SusanMillard, Andrew D.Biroccio, AnnamariaMillecamps, MagaliBiron, David GeorgesMiller, CharlesBironaité, DaivaMiller, DennisBiscetti, FedericoMiller, ElżbietaBischof, JoachimMiller, IngridBischof, SandraMiller, JustinBiscotti, Maria AssuntaMiller, KarlBishehsari, FarazMiller, KevinBishnupuri, Kumar S.Miller, Mark W.Bissonnette, Élyse Y.Miller, RachelBissonnette, NathalieMiller, Robert H.Biswas, ArijitMiller, Stephen C.Biswas, RoopaMiller, VandanaBis-Wencel, HannaMiller, W. ToddBitonti, MariaMiller, William B.Bitra, ArunaMillet, ÓscarBitto, AlessandroMillon, LaurenceBiundo, FabrizioMills, KenBIVONA, GiuliaMilosavljević, AleksandarBjelland, SveinMiloso, MariarosariaBjelobaba, IvanaMilovanovic, DragomirBjork, BryanMilroy, Jeffrey J.Bjorklund, RobertMimura, JunseiBlaby-Haas, Crysten E.Mimura, KazuyaBlack, James R.M.Min, Kyung-JinBlackburn, GavinMin, Kyung-SanBlagojevic Zagorac, GordanaMinafra, LuigiBlaheta, RomanMinami, MasaakiBlalock, WilliamMinami, YosukeBlamires, SeanMinamisawa, SusumuBlanc, EtienneMinarovits, JanosBlanch, Richard J.Mindt, ThomasBlanco, EduardoMine, AkiraBlanton, CynthiaMine, YuichiBlanvillain, RobertMineev, KonstantinBlasco Benito, SandraMineo, PlacidoBlasi, FrancescaMiners, ScottBlau, JennyMinervini, Crescenzio FrancescoBlazevic, IvicaMingorance, JesúsBlázquez, ManuelMinguet, Eugenio G.Bleibtreu, AlexandreMinh, David D. L.Bleilevens, ChristianMinh, TruongngocBlenda, AnnaMinic, ZeljkaBlesson, Chellakkan SelvanesanMinic, ZoranBlitek, AgnieszkaMiniero, Daniela ValeriaBloch, SylwiaMiniewicz, AndrzejBloch, WilhelmMinini, MirkoBlock, StephanMino, MasanobuBloise, NoraMinocha, Subhash C.Blomhoff, Heidi K.Minorics, RenátaBlomme, EricMintz, Cyrus DavidBlomme, JonasMinucci, SergioBlonska, MarzennaMinutolo, AntonellaBloom, KerryMio, CatiaBloor, IanMiosge, NicolaiBlossey, RalfMiotto, BenoitBlum, Jason L.Miquerol, LucileBlume, CorneliaMir, HinaBlumenschein, TharinMIR, MOHAMMED A.Blumer-Schuette, Sara E.Mir, Tahir MaqboolBlunk, TorstenMiragliotta, VincenzoBlunt, WarrenMiragoli, MicheleBłyszczuk, PrzemysławMiralles, Manuel G.Board, MaryMiranda, AdelaideBoarescu, Paul-MihaiMiranda, EnriqueBoase, NathanMiranda, HugoBoateng, JoshuaMiranda, João M.Boaventura, PaulaMircioiu, ConstantinBobasov, EvgeniiMiretti, SilviaBobay, Louis-MarieMirkhani, Seyyed AlirezaBobe, RegisMiro, JordiBobich, EdwardMironov, AlexanderBobiński, MarcinMirossay, LadislavBobis-Wozowicz, SylwiaMirshahi, MassoudBobrovskaya, LarisaMirzayans, RazmikBoccaccio, CarlaMirzoev, Timur M.Boccalatte, FrancescoMisasi, RobertaBoccardo, FrancescoMisawa, ErikoBoccella, SerenaMisciagna, GiovanniBocchinfuso, GianfrancoMiserey-Lenkei, StéphanieBock, FlorianMishima, HiroyukiBock, NathalieMishima, MasakiBocos, CarlosMishin, Alexander S.Boczkowska, MajaMishra, AbheepsaBodelón-González, GustavoMishra, Ajay KumarBodine, SueMishra, AwdheshBodo, EnricoMishra, MeerambikaBoeckel, Jes-NielsMishra, Nagendra N.Boege, FritzMishra, PriyankaBoehm, StefanMishra, RosalinBoehme, Karen A.Mishra, Santosh K.Boehr, David D.Mishto, MicheleBoekema, BoukeMisicka, AleksandraBoerma, MarjanMisiolek, MaciejBoeuf, HélèneMiskiewicz, PiotrBoffano, PaoloMiskulin, MajaBogacka, IwonaMistrik, MartinBogado Pascottini, OsvaldoMistriotis, PanagiotisBogdan, MariaMisu, TatsuroBogdanov, Alexey M.Misztalewska-Turkowicz, IwonaBoger, HeatherMita, KojiBogers, Willy M J MMitchell, Cassie S.Boggaram, VijayMitchell, Michael J.Bøgwald, JarlMitchell, MurrayBohannon, Richard W.Mithoefer, AxelBohl, DelphineMitjans Arnal, MontserratBöhrnsen, FlorianMitkin, Nikita A.Boia, RaquelMitola, StefaniaBoichuk, SergeiMitoma, HiroshiBoisivon, Helene RobertMitra, JoyBoissan, MathieuMitrofanis, JohnBoissinot, MarjorieMitsuhashi, WataruBojarczuk, KamilMitsui, ToshiakiBojarska, JoannaMitsuru, OkuwakiBojarski, PiotrMitsuya, ShiroBojková, BiankaMitsuyama, SusumuBol, KalijnMittal, SandeepBoland, C. RichardMittnacht, SibylleBoland, SebastianMitura, KatarzynaBolanowski, MarekMiura, NatsukoBölcskei, KataMiura, TakashiBoldison, JoanneMiyado, KenjiBoldizsár, ImreMiyagaki, TomomitsuBoldogh, IstvanMiyahara, TairaBoldrini, LucaMiyake, MasateruBollag, WendyMiyamoto, KojiBollong, MichaelMiyamoto, YoheiBollyky, PaulMiyashita, ShuheiBolognin, SilviaMiyata, YoshihikoBombard, SophieMiyazaki, MotoyasuBombardi, CristianoMiyazaki, TeruoBömer, NilsMiyazaki, TetsuroBommagani, ShobanbabuMiyazaki, ToshikiBommareddy, PraveenMiyoshi, DaisukeBommireddy, RamireddyMizia-Stec, KatarzynaBonacchi, MassimoMizobata, TomohiroBonacina, FabriziaMizuguchi, MarikoBonam, Srinivasa ReddyMizui, ToshiyukiBonanno, SilviaMizuno, HideakiBonar, EmiliaMizuno, TomohiroBonato, MatteoMizuo, KeisukeBoncela, JoannaMizzotti, ChiaraBond, Heather M.Mkrtchian, SourenBond, Simon T.Mladěnka, PřemyslBondar, Natalia PMladenov, EmilBondarev, Stanislav A.Mladenova, IrenaBonduelle, ColinMlaga, Kodjovi D.Bondy, Stephen C.Mlera, LuwanikaBonen, LindaMlinarić, SelmaBonetta, DarioMlostoń, GrzegorzBonfiglio, RitaMlynarczyk, DariuszBongers, KaleMo, ChenglinBonggi, LeeMo, FeiBongiorno, DafneMo, YiqunBonifacio, Maria A.Mo, YoungjunBonifacio, Maria AddolorataMoar, WilliamBönig, HalvardMoawad, AdelBonin, SerenaMobbili, GiovannaBonnelye, EdithMobed-Miremadi, MaryamBonnet, CrystelMochida, SumikoBonnet, PascalMochizuki, KazufumiBonnet, Pierre-AntoineModa, FabioBono, FedericaModahl, CassandraBonomi, FrancescoModenutti, CarlosBonomi, MarcoModesti, AlessandraBonomini, FrancescaModrowski, DominiqueBonora, ElenaMogi, MasakiBonora, MassimoMogielnicki, AndrzejBonvicini, CristianMöglich, AndreasBoomer, Jonathan S.Moha Ou Maati, HamidBoonen, MarielleMohamed, Azza H.Booth, BrianMohamed, HebaBooth, Kevin T.Mohamed, Junaith S.Boothby, ThomasMohamed, Salah A.Boppana, Nithin B.Mohamed, TamerBoquete, TeresaMohamed, Tarek MagdyBorbély, JánosMohammad, AliyuBorbone, NicolaMohammed, AltafBorcan, FlorinMohammed, YousufBorchert, GlenMohan, Ganesh Babu MalliBorde, ValérieMohanta, Tapan KumarBordes, María Dolores LlobatMohanty, SmitaBordes, PatriciaMohanty, VakulBordignon, VilceuMohd Hanif, ZulfakarBordin, LucianaMöhlmann, TorstenBorgel, DelphineMohr, SusanneBorger, VerenaMohsen, AttayebBorges, KarinMohsin, SadiaBorges, PedroMoin, MazaharBorghaei, Ruth C.Moir, Anne J.GBorghese, RobertoMoiseev, Roman V.Borghi, MonicaMoiseyev, GennadiyBorgnia, Mario J.Mojzych, MariuszBorgo, ChristianMok, TzeBorgonovo, GigliolaMokra, DanielaBorin, DmitryMokry, JaroslavBoris, PejinMoktefi, AnissaBorkowska, Edyta MartaMold, Matthew J.Borkowska, PaulinaMoldogazieva, N.T.Borlee, BradMolenaar, Remco J.Borlongan, Cesario VenturinaMolesini, BarbaraBorm, PaulMolgó, JordiBornancin, FrédéricMolina, Cristina E.Börner, RichardMolina-Cerrillo, JavierBornhorst, MiriamMolinari, SergioBoros, LaszloMolinari, SusannaBorota, AnaMolinier, JeanBoroviak, KatharinaMoll, GuidoBorovikov, Yurii S.Møller, Lisbeth BirkBorrelli, EnricoMøller, Thor ChristianBorschevsky, AnastasiaMolmenti, Ernesto P.Borse, VikrantMolnár, ElekBorsetti, AlessandraMołoń, MateuszBortey-Sam, NestaMoloney, Gerard M.Bortner, Carl D. Moltzau, Lise RománBortolotto, TissianaMolyneux, KarenBortoluzzi, CristianoMomekov, GregoriBorzok, MaegenMomeny, MajidBosca, LisardoMonaco, GiovanniBoschert, VerenaMonaghan, DanielBoscolo, ElisaMonari, AntonioBose, DebojitMonasky, MichelleBosnar, Maja HerakMoncharmont, BrunoBosnjak, BerislavMonchatre-Leroy, ElodieBosse, Jens B.Mondal, GoutamBossi, GianlucaMonfrini, MariannaBosso, AntonellaMong, Mei-ChinBostan, HamedMonget, PhilippeBostelmann, RichardMongin, Alexander A.Bostjancic, EmanuelaMonickaraj, FinnyBotelho, JoãoMonjarás Feria, JuliaBotella, Luisa MaríaMonje, Paula V.Boto, Roberto A.Monné, MagnusBott, AlexMonokrousos, NikolaosBotta, LuigiMonroe, Glen R.Botta, MauraMonroy, FernandoBottani, EmanuelaMonsalve, MariaBottari, GiovanniMonsarrat, PaulBotti, GerardoMontag, DirkBotticelli, AndreaMontag, JudithBotton, AlessandroMontagne, KevinBottone, Maria GraziaMontague, SamanthaBotturi, AndreaMontalvo-Ortiz, Janitza L.Botubol-Ares, José ManuelMontalvo-Rodríguez, RafaelBoublikova, LudmilaMontanari, ElitaBouchal, JanMontanaro, LorenzoBouché, MarinaMontarolo, FrancescaBoucheix, ClaudeMonte, IsabelBoucher, DaveMontecucco, FabrizioBouckaert, JulieMonteil, ChristelleBoue-Grabot, EricMonteiro, FilipaBougea, AnastasiaMonteiro, MarioBou-Gharios, GeorgeMonteiro, SílviaBoukens, Bastiaan J.Monteleone, IvanBoukpessi, TchilaloMontemiglio, LindaBoulaiz, HouriaMontemurro, CinziaBouley, RichardMontemurro, NicolaBoulling, ArnaudMontenegro, LuciaBouranis, Dimitris L.Montero, OlimpioBourbousse, ClaraMontero-Melendez, TrinidadBourdon, EmmanuelMontes Resano, MartaBourgault, SteveMontes, RaquelBourgeois, DominiqueMontesano, DomenicoBourgine, Paul EmileMontesano, VincenzoBourguet, WilliamMontesarchio, DanielaBourland, FredMontesinos, LauraBourneuf, EmmanuelleMonti, BarbaraBouropoulos, NikolaosMontiel-Duarte, CristinaBourras, SalimMontiel-Smith, SaraBourret, Robert B.Montoliu-Gaya, LaiaBouschet, TristanMontoro, MiguelBousquet, GuilhemMontrucchio, GiuseppeBousset, LucMonville, ChristelleBoutilier, KimMonza, LauraBouyer, DanielMoo, Eng KuanBové, JordiMoody, SuzyBowden, BruceMooers, BlaineBowen, Mark E.Mookerjee, ShonaBowerman, MelissaMoolhuijzen, Paula M.Bowers, Doria FMoon, ChangjongBowling, SarahMoon, Il SooBoyanova, LyudmilaMoon, Jong-SeokBoyd, Robert S.Moon, MinhoBoyer, Justin G.Moon, Seung MyungBoyles, Matthew S. P.Moon, Sung-KwonBoysen, ReinhardMoonen, Carolyn G.J.Bozdag, MuratMoore, CatrinBozic, JoskoMoore, DavidBozso, ZoltanMoore, Geromy GeorgeBozzi, FabioMoore, Matthew D.Bozzo, Gale G.Moore, StuartBrabet, PhilippeMoore, Thomas FBracco, EnricoMoore-Carrasco, RodrigoBrachner, AndreasMoorehead, RogerBracht, ThiloMoosmann, BerndBracken, AdrianMorabito, AlessandroBrader, GünterMorabito, AntoninoBradford, BarryMoraczewska, JohannaBradford, KentMoradian-Oldak, JanetBradfute, StevenMorais, MauricioBradley, Elizabeth W.Morais, VictorBradshaw, NicholasMorales Suárez-Varela, María M.Bradshaw, PatrickMorales, JulioBradshaw, Tracey D.Morales, PaulaBraendle, ChristianMorales, SerafinBrainard, MichaelMorales, Víctor AbadBraliou, GeorgiaMorales-González, José AntonioBrambilla, VittoriaMoral-García, José EnriqueBrancato, VirginiaMora-Montes, HectorBranch, Matthew JamesMoran, JoseBranchini, AlessioMoran, OscarBrand, KorbinianMorán, PalomaBrand, MarcusMorandi, LucaBrandao, BrunaMorante, Juan José HernándezBrandenburg, KlausMoravcikova, JanaBrandenburg, Lars-OveMordalski, StefanBrandli, AliceMoreira, Ana CarolinaBrandt, RolandMoreira, André FerreiraBrandusoiu, Ionut BogdanMoreira, Daniel CarneiroBranković, IvanMoreira, Irina S.Branlant, ChristianeMorel, BertrandBrantley, William A.Morel, Jean-LucBrantly, MarkMorelli, EugenioBratengeier, CorneliaMorelli, Maria BeatriceBrault, JeffreyMorello, SilvanaBraun, Andrew P.Moreno Amador, María LourdesBraun, Frank K.Moreno, AdrianBraun, RalfMoreno, JordiBraun, Rudolf K.Moreno-Gonzalez, InesBraun, WernerMoreno-Loshuertos, RaquelBräuniger, ThomasMoreno-Piraján, Juan CarlosBravatà, ValentinaMoreno-Villanueva, MariaBravo, JeronimoMoretto, AlessandroBravo, LidiaMorffy, NicholasBravo, Susana B.Morgan, Ethan L.Brazzolotto, XavierMorgan, PhilipBréchard, SabrinaMorgan, Rhianna K.Brecker, LotharMorgante, GiuseppeBreckwoldt, MichaelMori, KiyoshiBrehm, MartinMori, MasakiBrehme, MarcMori, ShigekiBreitbart, HaimMoricoli, DiegoBrejnrod, AskerMoriguchi, TakayaBremer, JeroenMorikawa, SatoruBrender, JeffreyMorikawa, ToshioBrenišin, MarekMorikawa, YukoBrennan, Anthony B.Morimoto, KinjiroBrennan, Todd V.Morimoto, TatsuyaBrenner, Annette K.Morimoto, YasuoBrenner, Gary J.Morimura, ShigeruBrenner, RolfMorini, LucaBrents, Lisa K.Morini, MartinaBresciani, CarlaMorino, KatsutaroBresciani, ElenaMorison, IanBresson-Bepoldin, LaurenceMorita, KentaBrestič, MariánMorita, ManabuBrestoff, Jonathan R.Morita, MitsuhiroBrett, ThomasMorita, NaokiBreunig, JoshuaMorita, ShigetoBreuzard, GillesMoritake, TakashiBrevini, Tiziana A. L.Moritz, MichałBrew-Appiah, RhodaMoriya, MakotoBrewer, Matthew G.Moriyama, ShtjnsukeBrezovský, JanMoriyama, YasukoBriana, Despina D.Morland, CecilieBriand, LoicMorleo, ManuelaBridges, ChristyMorohashi, KengoBrieño-Enriquez, MiguelMoros, MaríaBrieva, LluísMorouço, PedroBriganti, MatteoMorozov, Oleg G.Brill, Richard W.Morozov, Vitaly A.Brindisi, MatteoMorozov, Vladimir N.Brindley, DavidMorozova-Roche, LudmillaBrindley, MelindaMorra, MarcoBrini, EmilianoMorreau, HansBrini, MarisaMorrice, NicolaBrink, PeterMorrione, AndreaBrinkmann, VolkerMorris, AndrewBrito, Luis A.Morris, Jill K.Brito, NélidaMorris, KeithBritt, Rodney D.Morris, Paul FrancisBrittan, MairiMorrison, DouglasBritz, Margaret L.Morroni, FabianaBriz, OscarMorrow, John P.Brizuela-Madrid, LeyreMorsczeck, ChristianBroberg, MartinMorselli, MarcoBrock, JamesMortara, LorenzoBrodaczewska, Klaudia K.Morten, Karl J.Brodehl, AndreasMortensen, Luke J.Broderick, PatriciaMortensen, Martin S.Brodlie, MalcolmMosbah, Ismail BenBroeckling, CoreyMosca, LucianaBroer, StefanMoschini, RobertaBrogden, GrahamMoschou, PanagiotisBrogi, SimoneMoschovi, MariaBroglie, MartinaMoscucci, FedericaBrogna, ClaudiaMosgöller, WilhelmBrommer, BenediktMoshiri, AlaBrone, BertMoshiri, ArfaBrooks, BrianMosienko, ValentinaBrooks, Craig R.Moskalev, Alexey A.Brooks, Tracy A.Moškon, MihaBrorsson, Ann-ChristinMosqueira, DiogoBrosche, MikaelMosqueira, MatiasBrosens, JanMoss, Anthony G.Brotto, MarcoMoss, DonBroude, Eugenia V.Moss, WalterBrouwers, Jos F.Mossel, EricBrown, Alan B.Mota, ManuelBrown, AngelaMotakis, EfthymiosBrown, Christopher LyonMotlík, JanBrown, DennisMotloch, Lukas JBrown, Janet E.Motloch, Lukas J.Brown, LauraMotohashi, KenBrown, NelsonMotose, HiroyasuBrown, PatrickMotta, IreneBrown, Robert J.Mottes, MonicaBrown, Ronald B.Mou, HaiweiBrownstone, NicholasMou, YongchaoBroza, YoavMoudgil, KamalBrozek-Pluska, BeataMoujir, Laila M.Brözel, VolkerMoukhles, HakimaBrozmanová, MarianaMoulin, AlexandreBrozovic, AnamariaMourão, Carlos FernandoBrozyna, AnnaMousa, Shaaban A.Brucoli, MatteoMousa, ShakerBruder Do Nascimento, ThiagoMousley, CarlBruemmer, Jason E.Moustaïd-Moussa, NaïmaBrugarolas, PedroMoutinho, MiguelBrügger, BrittaMouzaki, AthanasiaBrugna, MyriamMouzeyar, SaïdBruinsma, BoteMoy, JamieBrukman, NicolasMoyes, DavidBrule, Sybille Van DenMozgova, IvaBrulíková, LucieMozos, IoanaBrullo, ChiaraMracek, TomasBrumos, JavierMravec, JozefBrun, PaolaMróz, WaldemarBrundage, Cord M.Msaouel, PavlosBruneel, ArnaudMu, Hai-JinBrunello, GiuliaMu, PingBrunetti, CeciliaMuccilli, VeraBrunetti, GiacominaMuceniece, RutaBrunetti, OronzoMucignat, CarlaBrunkhorst, RobertMück-Lichtenfeld, ChristianBrunner, SusanneMucsi, ZoltánBrünnert, DanielaMudgil, PoonamBruno, AndreinaMueller, Wolf C.Bruno, AnnalisaMueller-Roeber, BerndBruno, EleonoraMuff, RomanBruns, Danielle R.Mugisho, OdunayoBruschini, LucaMühle, ChristianeBruserud, ØysteinMühlfeld, ChristianBrusslan, JudyMuhovski, YordanBrust-Mascher, IngridMu-Hsin, ChangBruzzese, DarioMuhvic Urek, MirandaBruzzone, SantinaMujtaba, ShirazBrvar, MiranMukai, TomoyukiBryantsev, AntonMukherjee, AmitavaBrycki, BogumilMukherjee, DwaipayanBryk, PawełMukherjee, NabanitaBryniarski, KrzysztofMukherjee, RajibBryszewska, MariaMukherjee, SarbajitBrzeska, JoannaMukherjee, SomnathBrzeziński, KrzysztofMukherjee, Sudipbrzoska, malgorzata michalinaMukhtar, KarolinaBrzozowski, TomaszMukwaya, AnthonyBubu, OmonighoMulcahy, BenBuccarelli, MariachiaraMulder, CallistaBuccheri, Maria-AntoniettaMulet, Jose M.Bucci, CeciliaMulherkar, ShalakaBucciantini, MonicaMulholland, DavidBuchanan, Daniel D.Muller Kratz, JadelBuchberger, AlexanderMüller, Christoph W.Buchet, ReneMüller, Hans-Arno J.Buchheim, JudithMuller, LaurentBüchler, TomášMuller, MandyBuchman, VladimirMuller, MarcBuckel, WolfgangMuller, MiryamBucki, RobertMüller, Thomas ErnstBuckingham, LelaMüller-Deubert, SigridBuck-Koehntop, Bethany A.Müller-Vahl, Kirsten R.Buckley, Shannon M.Mulligan, Vikram K.Bucolo, ClaudioMullin, James M.Budimirovic, DejanMulthoff, GabrieleBudinsky, Robert A.Mulvey, MatthewBudzak, SimonMulvihill, ErinBuechler, ChristaMunafo, JohnBueno, ClarissaMunarin, FabiolaBuentello-Volante, BeatrizMunaron, LucaBufalieri, FrancescaMuniraj, NethajiBuffart, Tineke E.Muniswamy, MadeshBuga, Ana-MariaMunk, CarstenBugarin, AlejandroMuñoz, AlfonsoBugla-Płoskońska, GabrielaMuñoz, Francisco JoséBugno, RyszardMuñoz, VictorBuha, AleksandraMuñoz-Castañeda, Juan R.Buijs, GondaMuñoz-Chápuli, RamónBujacz, AnnaMuñoz-Descalzo, SilviaBujacz, GrzegorzMuñoz-Félix, José M.Bujak-Gizycka, BeataMuñoz-Torrero, DiegoBujdáková, HelenaMunro, DavidBujdoso, GezaMuntané, GerardBujko, MateuszMuntane, JordiBüki, AndrásMuntean, AlexandrinaBukowska, BożenaMunye, Mustafa M.Bukowska, JoannaMünzer, PatrickBukowski, MichalMupo, AnnalisaBulatov, EmilMurab, SumitBulatovic, MajaMuraca, MaurizioBulboacă, Adriana ElenaMurach, KevinBulc, MichałMurai, KojiBulfamante, Antonio MarioMuraille, EricBulgakov, VictorMurakami, KoheiBulla Jr, LeeMurakami, YasufumiBulla, RobertaMurakoshi, NobuyukiBulli, PeterMurale, Dhiraj P.Bullon, PedroMuraleva, Natalia A.Bulte, JeffMuranaka, AtsuyaBultynck, GeertMurani, EduardBunaciu, Rodica PMuranov, KonstantinBundschuh, Ralph A.Murase, DaikiBunghez, RalucaMurase, SachikoBunik, VictoriaMurashita, KojiBunkin, Nikolay F.Murata, HitoshiBunoiu, Octavian MădălinMurata, JunBunse, LukasMurata, SoichiroBuntov, EvgenyMurata, TakayukiBuonanno, ManuelaMuratani, MasafumiBurchakov, Denis I.Muratori, MonicaBurdet, NicolasMuratov, Eugene NBurdukiewicz, MichałMuravnik, Lyudmila E.Burek, MalgorzataMurawska-Ciałowicz, EugeniaBurga, Laura N.Murciano-Calles, JavierBurger, Michael CMurdica, ValentinaBurgess, David R.Muresan, Mihai StefanBurgess, JohnMurias, MarekBurghardt, KyleMurín, RadoslavBurghardt, RobertMurkovic, MichaelBurgstaller, JoergMuroya, SusumuBurkard, NatalieMuroya, YoshikazuBurke, Donald H.Murphy, AndrewBurke, RichardMurphy, Ciara M.Burke, Susan J.Murphy, DenisBurkel, EberhardMurphy, EainBurkhead, JasonMurphy, RonanBurlacu, AlexandrinaMurphy-Ullrich, Joanne E.Burleigh, Thomas D.Murray Stewart, TracyBurnier, JuliaMuruganandan, ShanmugamBurns, DavidMusacchia, FrancescoBurns, KatherineMusalgaonkar, SharmishthaBurpo, FredMusani, VesnaBurtey, StéphaneMusazzi, LauraBurton, Zachary F.Musch, MarkBus, Vincent G.M.Muschiol, JanBusardò, Francesco PaoloMuschol, MartinBusby, Ryan R.Musil, Linda S.Busch, MaikeMusilek, KamilBusch, MartinMusk, Gabrielle C.Bušek, PetrMußbacher, MarionBusetto, Gian MariaMusso, NataleBushakra, Jill MMusson, David S.Businaro, RitaMustachio, LisaBuskbjerg, Cecilie D. R.Mustafa, GhazalaBussalleu, EvaMusti, Anna MariaBusse, MandyMusulen, EvaBüsselberg, DietrichMusumeci, GiuseppeBussu, FrancescoMuszbek, LászlóBustamante, AlejandroMuszyńska, EwaBusto, RebecaMuszyński, SiemowitBustos, Diego MartinMuthamilarasan, MehanathanBustos, FranciscoMuthu Karuppan, Mohan KumarBustos-Martínez, JaimeMuthukumar, Alagar R.Bustuchina Vlaicu, MihaelaMuthukumar, KannanButkauskas, DaliusMuthuramu, IlayarajaButler, Georgina SMuthusamy, NagendranButler-browne, GillianMutis, TunaButnariu, MonicaMutoh, TatsuroButrón, AnaMutsaers, Steven E.Butu, AlinaMutti, LucianoByadgi, OmkarMuyldermans, SergeByadgi, Omkar VijayMuz, BarbaraBychkov, AndreyMuziol, Tadeusz MikolajBychkov, Maxim L.Muzykantov, Vladimir R.Byeon, HaewonMylonas, IoannisByler, Kendall G.Mylonis, IliasByrd, Alicia K.Mymryk, JoeByrd-Leotis, LaurenMyrianthopoulos, VassiliosByrne, Frances L.Mysiukiewicz, OlgaByun, JonghoeMysliveček, JaromírByun, KyungheeMyśliwiec, HannaByun, SangwonMysliwiec, TamiBzducha-Wróbel, AnnaMytych, JenniferCaaveiro, JoseMyung, HeejoonCaba, OctavioNa, DokyunCaballero-Garcia, BeatrizNa, Dong GyuCabaro, SerenaNa, Dong HeeCabiscol, ElisaNa, SungsooCabot, GabrielNabissi, MassimoCabral, Benedito J.C.Naccarato, MarcelloCabré, Juan J.Nacry, PhilippeCabrele, ChiaraNadai, MatteoCabrera, AdoracionNadakuduti, Satya SwathiCacabelos, RamónNadeau, Jay L.Cacciola, Nunzio AntonioNadeem, MuhammadCacciotti, IlariaNademanee, KoonlaweeCacciotto, CarlaNaderi, NassimCacho Teixeira, MiguelNadiminty, NagalakshmiCachón-Gonzalez, María BegoñaNaegeli, HanspeterCadario, FrancescoNaftalin, RichardCadefau Surroca, Joan AureliNag, Okhil K.Cadelis, Melissa M.Nagahage, IsuraCaenazzo, LucianaNagai, NoriakiCaetano-Anolles, DerekNagajyothi, Jyothi FCafasso, DonataNagano, SeiichiCafiero, MauricioNagaoka, IsaoCaglayan, MelikeNagaraj, Ram H.Cahill, MichaelNagaraj, ViniCahoon, A. BruceNagarajan, BalajiCahova, MonikaNagarajan, PrabakaranCai, BishuangNagarajan, Uma M.Cai, DeminNagasaka, KazunoriCai, HaiyuanNagasawa, KazueCai, HoujianNagashima, YukihiroCai, JasonNagata, EiichiroCai, JieruNagata, NaotoCai, JiyangNagata, ShinjiCai, KaiNagata, TakashiCaiati, CarloNagatoishi, SatoruCaiazza, MartinaNagatomo, YujiCaiazzo, ElisabettaNaghibalhossaini, FakhraddinCailhier, Jean FrançoisNagy, BálintCailliau, KatiaNagy, BélaCairo, MontserratNagy, CorinaCairrão, ElisaNagy, ElodCaissard, Jean-ClaudeNagy, Endre V.CAKIR, BirsenNagy, IstvanCakouros, DimitriosNagy, TamásCakstina, IneseNahalkova, JarmilaCalabrese, GianpieroNahi, HarethCalabrese, VittorioNair, Sunila G.Calabria, ElisaNaito, TateakiCalado, ÂngeloNaito, ToshioCalado, Cecília R.C.Najar, AmerCalamai, MartinoNajdenski, HristoCalandra, PietroNajera, FranciscoCalarco, AnnaNaka, HiroakiCalcagno, SimoneNakabayashi, KazumiCalcott, Mark J.Nakagawa, HiroshiCalderini, OrnellaNakagawa, NaokiCalderone, VitoNakagawa, TakuroCaldo, Kristian MarkNakagawa, TetsuyaCaldovic, LjubicaNakagomi, TakayukiCalendar, RichardNakahama, Ken-ichiCaleo, MatteoNakahara, TsutomuCalero Valdayo, Patricia MariaNakahata, ShingoCali, JamesNakajima, AkiraCaligiuri, IsabellaNakamaru-Ogiso, EikoCaligo, Maria AdelaideNakamichi, NorihitoCalina, DanielaNakamura, KazufumiÇalışkan, GürselNakamura, KeigoCalistri, AriannaNakamura, KoheiCalistri, DanieleNakamura, KyoheiCalixto, Cristiane P.Nakamura, MasanaoCallaini, GiulianoNakamura, MasatoCallaway, Todd R.Nakamura, MotokiCallebaut, IsabelleNakamura, NobuhiroCalligari, PaoloNakamura, NorikoCalogeropoulou, TheodoraNakamura, Shin-ichiCaltabiano, RosarioNakamura, TomokiCalvani, RiccardoNakamura, YokiCalvaruso, MarcoNakamura, YoshiyukiCalvert, JaneNakano, MichiharuCalvi, BrianNakano, ShuichiCalvo, Ana CristinaNakano, TakanariCalvo-Lobo, CésarNakano, TamakiCalzada, María J.Nakano, ToshiakiCamacho, M. EncarnaciónNakao, ReikoCamaioni, AntonellaNakase, HiroshiCamara, JohannaNakase, IkuhikoCambra Bort, Josep M.Nakashima, KazuoCamejo, GermánNakata, MiyukiCameli, PaoloNakatani, YoshihikoCameron, DonaldNakatsu, KanjiCameselle-Teijeiro, Josè ManuelNakatsuka, AkiraCametti, CesareNakatsukasa, KunioCamins, AntoniNakayama, HironaoCammisotto, VittoriaNakayama, HokutoCampa, ClaudineNakayama, JunCampagnolo, LuisaNakayama, MasafumiCampana, DavideNakayama, MasayoshiCampanella, AlessandroNakayama, NaomiCampanella, MichelangeloNakayasu, ErnestoCampbell, BradleyNakazawa, MitsuruCampbell, ColinNakchbandi, InaamCampbell, Corey L.Nakhjavani, MaryamCampbell, MichaelNale, JanetCampbell, MorayNalli, CeciliaCampbell, Susan L.Nalvarte, IvanCamper, SallyNam, Jin-WuCamphausen, Kevin A.Nam, Ki HyunCampiche, RemoNam, Kung-WooCampiglia, PietroNam, Seung YunCampilho, AnaNam, Young-JaeCampo, GianlucaNamasivayam, VigneshwaranCampodoni, ElisabettaNambara, EijiCampolo, FedericaNamgaladze, DmitryCamporesi, Enrico M.Namihira, MasakazuCampos, Camila D. MadeiraNanba, EijiCampos, CatarinaNand, AshveenCampos, Joana F.Nanda, SatyabrataCampos-Parra, Alma DeliaNanda, Sitansu SekharCampos-Toimil, ManuelNandi, DeipanjanCamproux, Anne-ClaudeNandi, ShyamCampus, GuglielmoNandi, SomenCampuzano, AltheaNanduri, RavikanthCampuzano, OscarNanno, MasanobuCañadas, OlgaNanoff, ChristianCanal, Clinton E.Nanou, AfroditiCanals, IsaacNaoe, YoshinoriCanas, JoseNaoumkina, MarinaCañas, Rafael A.Naponelli, ValeriaCancemi, PatriziaNappi, LuciaCancian, MauroNaqash, Abdul RafehCandéias, SergeNaranjo, TomásCandela, Diaz-CanestroNarayan, EdwardCandela, HéctorNarayan, MaheshCândido Carvalho, KátiaNarayanan, GaneshCândido, ElizabeteNardecchia, StefaniaCanela, NúriaNardelli, CarmelaCanepari, MonicaNardi, FrancescoCanfield, ScottNardi, James B.Cangiano, BiagioNardi, SerenellaCanosa, StefanoNardini, MarcoCantaluppi, VincenzoNarendra, DerekCantamessa, SimoneNarita, ShintaroCantley, Melissa D.Narra, Hema PrasadCañueto, JavierNaruishi, KojiCanut, HerveNarute, PurushottamCao, GuodongNarváez, ManuelCao, Hieu X.Narwani, Tarun JaiRajCao, JianNascimento, Weslania VivianeCao, TongyuNash, KevinCao, XianNashimoto, YujiCao, XuNashine, SonaliCao, ZheNasi, MilenaCápal, PetrNasim, Muhammad JawadCaparello, ChiaraNasim, TalatCaparrotta, StefaniaNasim, ZeeshanCapasso, AnnaNassa, GiovanniCapdevila, Daiana ANassar, Zeyad D.Capel, CarmenNässel, Dick R.Capel, JuanNasser, Mohd WasimCapela E Silva, FernandoNassini, RominaCapell, TeresaNassir, FatihaCapilla-González, VivianNassisi, MarcoCapillo, GioeleNastasa, CristinaCaplan, AllanNastasi, ClaudiaCaponi, SilviaNasti, Tahseen HCaponnetto, AngelaNastoupil, Loretta JCapozzi, AnnaNat, Irina RoxanaCappadone, ConcettinaNatale, DavidCapparè, PaoloNatale, GianfrancoCappelletti, GraziellaNatarajan, PurushothamanCappellini, FrancescaNatarajan, SivaramanCappello, FrancescoNatarajan, ViswanathanCappello, ValentinaNatarelli, LuciaCappoen, DavieNatesan, ShanmugasundaramCappuccilli, MariaNath, AudreyCapraro, JessicaNath, NiharikaCaprini, ElisabettaNath, ShubhankarCapuano, AlessandraNathanailides, CosmasCapuano, AlessandroNathanson, LubovCaputa, MichałNatile, GiovanniCaputo, IvanaNatoli, SilviaCaraballo, RémiNaujok, OrtwinCaraballo-Rodríguez, Andrés MauricioNauli, SuryaCaraci, FilippoNault, RanceCarafa, VincenzoNaumov, Anton V.Caraglia, MicheleNaumovski, NenadCarapelli, AntonioNaumowicz, MonikaCarare, Roxana OctaviaNava, SaraCarata, ElisabettaNavarra, MicheleCarazo, AlejandroNavarrete-Vazquez, GabrielCarberry, PatrickNavarro, ElisaCarbone, CarmineNavarro, EstanisCarbone, FabrizioNavarro, RodrigoCarboni, LuciaNavarro-Guillén, CarmenCardinale, JensNavarro-López, Juan D.Cardona, FernandoNavas, AlfonsoCardone, Maria FrancescaNaveed Yousaf, MuhammadCardoso, Bruno AntónioNaves, ThomasCardoso, Isabel SantosNaviglio, SamueleCardoso, TainaNavizet, IsabelleCardozo, Licy YanesNavone, StefaniaCarella, Angelo MNawade, BhagwatCarella, PhilipNawaz, FahimCarestia, MariachiaraNawirska-Olszańska, AgnieszkaCaretta, AntonioNawrot, BarbaraCaretto, SofiaNawrot, RobertCariboni, AnnaNayak, Manasa K.Cariello, MaricaNayak, NiharCarlin, DanielleNaylor, KariCarlon, MarianneNazar, Ross N.Carlos, Sao-JoseNazare, MarcCarlotto, SilviaNazarov, AlexeyCarlyle, BeckyNazaruk, EwaCarmieli, RaananNazimek, KatarzynaCarmona Mejías, RitaNazio, FrancescaCarmona, F. DavidNazzaro, GianlucaCarmona, JUNdika, JosephCarmosino, MonicaNeagu, AdrianCarnero, AmancioNechepurenko, Ivan V.Carnevale Neto, FaustoNeda, IonCarol, PierreNeelakantan, PrasannaCaroleo, Maria CristinaNeelam, SrujanaCaroppo, EttoreNeerman-Arbez, MargueriteCarotenuto, MarcoNees, MatthiasCarotti, SimoneNeffe, Axel T.Carpenter, GuyNefzger, ChristianCarpi, SaraNeginskaya, Maria A.Carr, ChristopherNegrea, PetruCarrasco, ElisaNehela, YasserCarrasco-López, CésarNeidlinger-Wilke, CorneliaCarrella, SabrinaNeira, José L.Carreras, JoaquimNekhai, SergeiCarrier, PaulNelson, Amanda M.Carrieri, AntonioNelson, AndrewCarriero, Maria VincenzaNelson, Dorothy ACarrillo, JenifferNelson, Fred R.T.Carrión, MarNelson, RandyCarro, EvaNemec, IvanCarroll, ChadNemery, BenoitCarroll, James A.Nemes, KatalinCarroll, Lara S.Nemeș, Roxana MariaCarroll, ThomasNémeth, András GyörgyCarrouel, FlorenceNemeth, Zoltan HCarruthers, Nicholas J.Nemirovskaya, Tatiana L.Cartee, Gregory D.Nemoto, KiyomitsuCarter, WayneNemoto, WataruCarulli, LuciaNenna, AntonioCarullo, GabrieleNenoi, MitsuruCaruntu, ConstantinNenov, Miroslav N.Caruso, CalogeroNeo, Ana G.Caruso, CarlaNeophytou, Christiana M.Caruso, CristianoNepal, Mahesh RajCaruso, GerardoNeri, GiovanniCaruso, GiuseppeNeri, MatteoCaruso, SalvatoreNeri, SimonaCarvajal, KarlaNesan, DinushanCarvajal, MicaelaNesbitt, Warwick S.Carvalho De Oliveira, JaquelineNesmelov, Yuri E.Carvalho, Danilo OliveiraNestares Pleguezuelo, Maria TeresaCarvalho, EdmundNesterov-Mueller, AlexanderCarvalho, Lina A.S.Nestor, CaseyCarvalho, LuísaNestrasil, IgorCarvalho, Marta S.Nethi, Susheel KumarCarver, John A.Nettersheim, DanielCasadei, NicolasNeubauer, DamianCasadei, RaffaellaNeubert, ReinhardCasadio, RitaNeufeld, HowardCasado-Díaz, AntonioNeufeldt, Christopher JohnCasais, RosaNeufurth, MeikCasalino, GiuseppeNeugebauer, DorotaCasalou, CristinaNeuhaus, JochenCasals, GregoriNeuhauss, StephanCasamassimi, AmeliaNeuhuber, Winfried L.Casanova, LlanosNeumann, BrentCasanova, NancyNeumann, DonnaCasanova-Sáez, RubénNeumann, JakeCasao Gascón, AdrianaNeunaber, ClaudiaCasari, AliceNeupane, MaheshCasarini, LivioNeupane, Yub RajCasas, Ana M.Neureiter, DanielCasas, FrançoisNevárez-Moorillón, Guadalupe VirginiaCascella, RaffaellaNeves, JoãoCascini, Giuseppe LucioNeves, VeraCascioferro, StellaNevi, LorenzoCaseiro, Ana RitaNevoral, JanCaseiro, ArmandoNewmire, DanielCasellini, CarolinaNewsome, Timothy P.Caser, MatteoNewton, JasonCaseys, CelineNewton, RobertCasini, GiovanniNeyroud, DariaCasiraghi, AntonellaNezhat, Farr RezaCaspari, ThomasNg, Ho LeungCassano, DomenicoNg, Hooi HooiCassiman, DavidNg, Keng WooiCassisi, Jeffrey E.Ng, Pei YingCassone, GiuseppeNg, RayCastagna, MichelaNg, Tzi BunCastaing, BertrandNg, Wai-LungCastaldi, AlessandraNgezahayo, AnacletCastaldo, PasqualinaNguyen, Chi HuuCastaldo, RossanaNguyen, COL ChiCastanheira, Elisabete M.S.Nguyen, DuyCastan-Laurell, IsabelleNguyen, Khoi TanCastaño, Justo P.Nguyen, Linh T.BCastel, HélèneNguyen, Long TheCastellano, MarNguyen, Minh ThoCastellano, María DominguezNguyen, QuynhCastellano, OrlandoNguyen, Thanh HungCastellanos, AinaraNguyen, TrieuCastiglioni, BiancaNgwa, Che JuliusCastillejo, Maria AngelesNianiou-Obeidat, IriniCastillo, FranciscoNicaise, CharlesCastillo, SilviaNiccoli, TeresaCastillo-Gonzalez, Claudia MarcelaNiclos Gutiérrez, JuanCastle, AlanNicolas, CyrilCastoldi, AngelaNicolas, EmmanuelleCastren, EeroNicolás, Francisco J.Castriconi, RobertaNicolas, GaëlCastro, AnaNicolazzo, JosephCastro, AndreiaNicoletti, FerdinandoCastro, Fidel OvidioNicoletti, VincenzoCastro, Pedro HumbertoNicolì, FrancescaCastro, Tarsila G.Nicolini, GabriellaCastro-Giner, FrancescNicoll, Amanda J.Castro-Marrero, JesusNicoll, RachelCastro-Torres, Rubén DaríoNiculae, DanaCastroverde, Christian Danve M.Niculescu, LoredanCasu, GavinoNiczyporyk, Jowita SamantaCasuso-Pérez, RafaelNie, YuCatalani, ElisabettaNiederberger, EllenCataldi, AmeliaNiederseer, DavidCatalin, BogdanNiederwieser, DietgerCatanzaro, GiuseppinaNiehrs, ChristofCatarzi, DanielaNielsen, Claus H.Cate, Hugo TenNielsen, MortenCatez, FrédéricNielsen, Thorbjørn TerndrupCato, Andrew C. B.Nielsen, Vance GirardCatravas, JohnNiemann, CarstenCatros, SylvainNiemczyk, MariuszCattaneo, FabioNieminen, Taina T.Catto, MarcoNiemirowicz-Laskowska, KatarzynaCauffiez, ChristelleNieoczym, DorotaCaufield, J. HarryNiesor, Eric J.Caulfield, Thomas R.Nieuwenhuizen, Niels J.Cauli, OmarNievergelt, Adrian PascalCausse, MathildeNifantiev, NikolayCavalcanti, João Henrique F.Nifli, Artemissia-PhoebeCavalieri, FrancescaNighot, PrashantCavalieri, VincenzoNijnik, AnastasiaCavalletto, LuisaNikishin, DenisCavalli, LoredanaNikitin, Dmitri V.Cavalu, SimonaNikitovic, DraganaCavanaugh, JaneNikodemova, MariaCavazzoni, AndreaNikolaev, AntonCavé, ChristianNikolaidis, NikolasCazzola, RobertaNikolaivits, EfstratiosCebova, MartinaNikolakaki, EleniCeccherini, Maria TeresaNikolakakis, IoannisCecchi, IreneNikolakopoulou, Angeliki M.Cecconi, CiroNikolay, RainerCecconi, SandraNikolic, DejanCeci, AndreaNikolich, MikeljonCecile, VignalNikoloudakis, NikolaosCegolon, LucaNikolova, IrinaCejkova, JitkaNikonenko, VictorCekanaviciute, EgleNilsen-Hamilton, MaritCelakovska, JarmilaNilsson, MariaCeletti, AngelaNina, BrutchCeliński, KonradNinni, SandroCelsi, FulvioNirala, Niraj K.Cembrowska-Lech, DanutaNishi, MayumiCenac, NicolasNishi, RyotaroČėnas, NarimantasNishibori, MasahiroCenci, GiovanniNishida, EmiCenciarelli, CarloNishida, HarutoCencioni, ChiaraNishida, KenseiCendron, LauraNishida, ShoCengiz, Ibrahim FatihNishida, ShozoCenter, Rob J.Nishiguchi, MasamichiCerchiara, TeresaNishihara, HidenoriCerchione, ClaudioNishihara, RyoCerdán, María-EsperanzaNishikawa, KiisaCereda, CristinaNishikawa, MasakiCereda, MatteoNishikawa, YujiCeresa, BrianNishimura, TakeshiCereseto, AnnaNishiyama, AkikoČerná, MarieNishiyama, AkiraCernat, AndreeaNishiyama, YoshitakaCerón-Carrasco, José PedroNishiyama, YusukeCerrito, Maria GraziaNishizawa, YokoCeruti, StefaniaNishizuka, MakotoCervelli, ManuelaNislow, CoreyCervelli, TizianaNisoli, EnzoCervera, AnaNissen, LorenzoCervetto, ChiaraNissen, Peter HCesaro, AnnabelleNistri, AndreaCescutti, PaolaNitti, Maria PaolaCestmir, AltanerNitulescu, George MihaiCeyzériat, KellyNitulescu, GeorgianaCha, Hee-JaeNitulescu, MihaiCha, Hwa JunNiu, Dau-MingCha, Hyuk-JinNiu, FangChaanine, Antoine H.Niu, HengyaoChaban, InnaNiu, TianhuaChaber, RadosławNixon, Daniel W.Chabot, BenoitNiyazov, Dmitriy M.Chacko, Jenu VargheseNjoku, Dolores B.Chadwick, Amy E.Nobile, StefanoChae, HeeyoungNobili, StefaniaChae, Sung-sukNobis, MaxChae, YounbyoungNóbrega, ClévioChagraoui, AbdeslamNóbrega-Pereira, SandrinaChai, Han-haNobs, Samuel PhilipChai, HongyanNocentini, AlessioChai, RenjieNocentini, GiuseppeChai, Toby C.Nociari, MarceloChaitanya, SaurabhNofer, Jerzy-RochChaki, MouniraNogales Gadea, GiselaChaklader, ReazNogales, AitorChakrabarti, SubhadeepNogales-Gadea, GiselaChakraborty, ArijitNoghero, AlessioChakraborty, IndranilNoguchi, Constance TomChakraborty, RajaNoguchi, SatoruChakraborty, SayanNoguchi, TakuyaChakravorty, DavidNogueira, Paulo JorgeChalard, PierreNoguera, Nélida InésChałas, RenataNogues, IsabelChałasiński, GrzegorzNohara, KazunariChalla, DilipNohata, NijiroChallagundla, KishoreNohe, AnjaChallet, EtienneNolan, JohnChalovich, Joseph M.Nolan, TaniaChami, BelalNollert, Matthias U.Chami, MouniaNoll-Hussong, MichaelChamuleau, Robert A.F.M.Nolte, IngoChamulitrat, WaleeNomura, SeitaroChan, Ben C. L.Nonaka, TaichiroChan, Edward D.Nonami, AtsushiChan, K.L.Nonell, SantiChan, Koon-HoNonomura, Ken-ichiChan, William K.Noonepalle, SatishChan, Yau KeiNoorani, ImranChan, Yin-ChingNoordam, RaymondChand, Kirat K.Norcic, GregorChand, VaibhavNordén, RickardChandrasekar, IndraNoren Hooten, NicoleChandrasekaran, VidyaNorgren, LarsChandrasekharan, BinduNoriega, Fernando G.Chang, Audrey N.Norinder, UlfChang, Cheng-ChangNoritaka, YamaguchiChang, Chia-cheNorman, KennethChang, Chia-JungNorman, TrevorChang, Chien-ChungNormark, BenjaminChang, Ching-JinNorton, Kerri-AnnChang, Chuang-RungNorton, MichaelChang, Hai-ChouNorval, MaryChang, Hao-HuengNosch, Daniela SonjaChang, Hao-XunNosoudi, NasimChang, HsiNotaras, Michael J.Chang, Hui-YunNotarbartolo, MonicaChang, HyunNotario-Pérez, FernandoChang, Ing-FengNotarstefano, ValentinaChang, Jer-MingNottegar, AlessiaChang, Jia-FengNoubissi, Felicite KamdemChang, Jui-ChengNoureddine, AchrafChang, Ke-VinNouvian, RegisChang, Kuo-HsuanNovak Kujundžić, RenataChang, Li-ChingNovak, IonaChang, Ling-ChuNovak, SanjaChang, Long-SenNovick, DanielaChang, Nai-JenNovick, Richard P.Chang, Shu-ChunNovikov, Alexander S.Chang, Shun-FuNovikov, Arthur I.Chang, Te-ShengNovinec, MarkoChang, Tzu-ChingNovío, SilviaChang, Wei ChiaoNovo, LuísChang, Wei-MinNovoplansky, ArielChang, Won HyukNovo-Veleiro, IgnacioChang, Yu-ChanNowacka, MariaChang, Yu-ChaoNowaczyk, AlicjaChang, Yun SilNowak, AdrianaChang, Yu-WeiNowak, Romana A.Chao, CeciliaNowicka, GrażynaChao, Chia-TerNowicki, MarcinChao, Chien-MingNowicki, MichałChao, K.S. CliffordNowicki, RomanChao, Yin XiaNowotny, MarcinChapoval, Svetlana P.Nozaki, MasamiChappell, GraceNozumi, MotohiroChappell, JohnNtougias, SpyridonChappell, KeithNuber, SilkeChappell, Mark C.Nuc, KatarzynaChapple, SarahNuc, PrzemysławChapuis, JulienNucci, NathanielCharles Jacob, Harrys KishoreNucera, EleonoraCharles Richard, John LalithNudler, EvgenyCharles, PhilipNuesch, JurgCharlton, AmandaNumakawa, TadahiroCharlton, ClivelNunes, FernandoCharriaut-Marlangue, ChristianeNunes, Francis Morais FrancoChase, Christine D.Nunez De Cáceres González, Francisco FedericoChastre, ÉricNunziata, AngelinaChatain, NicolasNunzio, VicarioChatrikhi, RakeshNussbaumer, ThomasChatterjea, DevananiNuţǎ, Diana CameliaChatterjee, AnushilaNuzziello, NicolettaChatterjee, IshitaNuzzo, DomenicoChatterjee, MadhumitaNwaiwu, OgueriChatterjee, NimratNwosu, Zeribe ChikeChatterjee, SampurnaNylander, KarinChatterjee, Som S.Nypelo, TiinaChatterjee, SubrotoNyström, AlexanderChaturvedi, PalakNzau Matondo, BillyChatzaki, EkateriniO’Callaghan, DavidChaudhari, KiranO’Connell, ThomasChaudhri, VirendraO’Connor, ChristineChauhan, BhareshO’Day, DantonChauhan, HarshO’Dell, William BradChauhan, NeerajO’Halloran, KennethChaurasiya, ShyambabuO’Keeffe-Ahern, JonathanChauveau, LiseO’Leary, Brendan M.Chavan, HemantkumarO’Leary, SeánChavarro Fierro, Martha CarolinaO’Leary, SeonadhChaveroux, CedricO’Neal, HollisChaves, ElenaO’Neal, Matthew E.Chaves, Susana RodriguesO’Reilly, Kally C.Chaves-Martinez, Felipe JavierO’Reilly, StevenChavez, FranciscoO’Rourke, JamieChawengsaksophak, KallayaneeO’Sullivan, Jeremy A.Checa, AntonioOakhill, JonChecchetto, VanessaObana, AkiraChekanov, KonstantinObara, YutaroChelli, RiccardoObata, YasukoChemello, LilianaObeng, SamuelChemtob, SylvainOberemok, Volodymyr V.Chen, Alan A.Obermüller, NicholasChen, Chao-JungObeso, AnaChen, CharlesObialo, Chamberlain I.Chen, ChenObinata, DaisukeChen, Chien-ChinObrador, ElenaChen, Chi-LongObri, ArnaudChen, Chin-ChuanObuchowski, MichalChen, Chiung-MeiObukhov, Alexander G.Chen, Chun-ChiOchatt, SergioChen, Chung-MingOchayon, David E.Chen, Chun-HanOchi, ShinichiroChen, DanicaOchiai, DaigoChen, Der-YuanOchoa, TheresaChen, Fure-ChyiOchoa-Reparaz, JavierChen, GangOchs, Raymond S.Chen, GuijieOczkowicz, MariaChen, HansenOdagi, MinamiChen, HaoOddi, SergioChen, HaotongOdenthal, MargareteChen, HaoyuanOdierna, GaetanoChen, HuOdintsova, Tatyiana I.Chen, HuiOechslin, FrankChen, Hui-ChenOfir, RivkaChen, Jhy-DerOgai, KazuhiroChen, JiOgami, KoichiChen, JieliOgasawara, ToruChen, JinbiaoOgata, YoshiyukiChen, Jin-ShuenOgawa, AkikoChen, JunpingOgawa, HidehikoChen, Jyh-PingOgawa, YouichiChen, KaihongOgbureke, Kalu UEChen, Kuan-FuOger, Philippe M.Chen, Kuan-WeiOgino, ShujiChen, Kuo-HuOgneva, IrinaChen, LianminOgnibene, MarziaChen, Li-HanOgórek, RafałChen, LinÖgren, Sven O.Chen, LinyiOgura, TakehikoChen, LisongOh, ByungchulChen, LixianOh, JunChen, Mei-JouOh, Jun-SeokChen, Miao-HsuehOh, Man-hoChen, MingOh, Min KyuChen, Mu-KuanOh, SeikwanChen, Pei-WenOh, Soo YoungChen, QiOh, YohanChen, QianOhashi, ManabuChen, QunOhashi, RiukoChen, Sean Chun ChangOhko, KentaroChen, Sheng-HungOhkubo, HirotsuguChen, Shih-ChiehOhlmann, Andreas V.Chen, Shih-ChuOhlsson, Sophie M.Chen, Shih-KuoOhmido, NobukoChen, Shin-ChehOhmiya, YoshhiroChen, ShukunOhneda, KinukoChen, Shuo-BinOhnishi, ShihoChen, ShuyangOhno, JunChen, Si-ChongOho, TakahikoChen, Suet NeeÖhrfelt, AnnikaChen, SuzieOhta, YukoChen, Szu-TahOhtsuka, AkiraChen, Tai-HengOhtsuka, MasayukiChen, Tzong-YuehOhtsuki, GenChen, VincentOhtsuki, TakashiChen, WeiOhya, SusumuChen, Wei-JungOhya, YoshikazuChen, WeiqinOikawa, TsunekazuChen, Wei-ShengOiso, NaokiChen, Wei-YuOjha, RaniChen, WenchunOka, YujiChen, Wen-YingOkada, HiroshiChen, XiOkada, MotohiroChen, XiaoyanOkada, TomoyoChen, XingchengOkae, HiroakiChen, XingqiOkamoto, CurtisChen, XiongwenOkamoto, MamoruChen, XuOkamoto, MasamiChen, Yao-tsengOkamoto, TakayukiChen, Yi-ChunOkamura, YuChen, Yih-FungOkazaki, SatoshiChen, Yih-YuanOkazaki, YozoChen, YiliangOkazawa, AtsushiChen, Yi-WenOkba, Nisreen M.A.Chen, YuOkegawa, TakatsuguChen, Yuan-ChuanOkita, Thomas W.Chen, Yuan-TsungOklešťková, JanaChen, Yu-ChihOklinski, Michal Krystian EgelundChen, Yung-ChihOkochi, MinaChen, Yun-JuOkoń, SylwiaChen, ZhonghuaOkuda, NagakoChen, ZhongzhiOkuda-Ashitaka, EmikoChénais, BenoitOkumura, AkihisaCheng, Chien-YuOkumura, MasakiCheng, ChunmingOkumura, NobuakiCheng, Fong-YuOkumura, NobuoCheng, HailiOláh, JuliannaCheng, HaiziOlar, RodicaCheng, HenriqueOlczyk, KrystynaCheng, Heung-ChinOlczyk, PawełCheng, Hong ShengOldfield, EricCheng, Hung-ChiOleksiewicz, UrszulaCheng, Jya-WeiOliva, FrancescoCheng, Ming-HueiOliva, María ÁngelaCheng, PeilinOliveira, Ana M.M.Cheng, Shih-PingOliveira, Mariana Bleker DeCheng, Wei-chiehOliveira, Mariana BragaCheng, XiangOliveira, Paula A.Cheng, XiaodongOliveira, Rui S.Cheng, XinlaiOlivier, Jean LucCheng, Yi-ShengOliviera, PedroCheng, Yu-JungOlkkonen, Vesa M.Cheon, Yong-PilÖllinger, RobertCheong, HeesunOlmo, FranciscoChereddy, KiranOlnes, Matthew J.Cherkaoui Malki, MustaphaOlova, NellyChern, Chi-LiangOlsen, Christopher M.Chern, Ming-KaiOlson, MatthewChernikov, OlegOlson-Manning, CarrieChernoff, YuryOlsson, MartinChernova, TatianaOlszewska, MartaCherry, JonathanOlszewska, Monika ACheruzel, LionelOltolina, FrancescaChesney, EdwardOltra, ElisaChester, Adrian HOmar, Ahmad A.Cheung, Ming-YanOmori, YasufumiCheungpasitporn, WisitOmote, HiroshiChevalier, ChristopheOmura, JunichiChevalier, FrançoisOndřej, VladanChevillard, ChristopheOnel, BuketChevret, EdithOnimaru, HiroshiChhabra, GaganOnkokesung, NawapornChhabra, YashOno, MasanoriChi, Celestine N.Ono, NoriakiChi, GeraldOno, TakashiChi, Yu-JenOno, YusukeChiang, Chi-LingOnomoto, KojiChiang, Hsiu-MeiOnori, PaoloChiang, Hung-LungOnukwufor, John O.Chiang, Meng-TsanOnyango, IsaacChiang, Ming-KoOnyeagucha, Benjamin ChidiChiang, NaijungOorschot, DorothyChiang, Tung-chinOpaliński, ŁukaszChiang, WeichungOpiela, JolantaChiang, Yi-TingOpländer, ChristianChiang, Yu-ChungOpocher, EnricoChiang-Ni, ChuanOppert, BrendaChiapella, JorgeOralová, VeronikaChiara, María-DoloresOramas-Royo, SandraChiaraluce, RobertaOrban, JohnChiarella, EmanuelaÖrdög, AttilaChiavaroli, AnnalisaOrecchioni, MarcoChibalin, Alexander V.Orefice, Nicola SalvatoreChieffi, PaoloOrekhov, Alexander N.Chien, ChianshiuOrellana, EstebanChien, JeremyOrelle, CédricChiesa, GiuliaOren, YoramChiesa, MattiaOrenes-Piñero, EstebanChifiriuc, Mariana CarmenOrengo, DorcasChifiruc, Mariana CarmenOresnik, IvanChigbu, De Gaulle IOrf, IsabelChighizola, Cecilia BOrian, LauraChim, Shek ManOrii, Kenji E.Chimenti, IsottaOrive, Alejandro GonzalezChimienti, Guglielmina AlessandraOrkusz, AgnieszkaChin, Kok YongOrlandi, AugustoChin, Young-WonOrlandi, Viviana TeresaChina, ArnabOrlandini, MaurizioChinchilla Salcedo, NuriaOrlando, GiustinoChino, MarcoOrloff, MarlanaChintala, SreenivasuluOrłowski, MarekChintalapudi, SumanaOrnoy, AsherChioccarelli, TeresaOrosco, PabloChiocchetti, AnnalisaOrrú, ChristinaChiou, Chun-TangOrsini, JoeChiou, Shih-HwaOrso, EvelynChiriacò, Maria SerenaOrsolic, NadaChiricozzi, ElenaOrsucci, DanieleChisari, EmanueleOrtan, AlinaChitale, ShalakaOrtega, Angel L.Chitarra, WalterOrtega, EduardoChitraju, ChandramohanOrtega, Francisco JoséChittori, SagarOrtega, Miguel AngelChiu, Ching-FengOrtega-Gutierrez, SilviaChiu, Hua-ShengOrtega-Villaizan, Maria Del MarChiu, JoannaOrtín-Martínez, ArturoChiu, Ka Fung PeterOrtiz, J. BryceChiu, Ming-JangOrtiz, JorgeChiu, Norman H. L.Ortiz, Maria J.Chiu, Tsai-HsinOrtiz-Masiá, DoloresChiurazzi, MaurizioOrtolani, FulviaChivero, ErnestOrtolano, SaidaChiyoda, TatsuyukiOrton, Thomas J.Chizhov, Alexander O.Orusa, RiccardoChizzolini, CarloOrynbayeva, ZulfiyaChladek, GrzegorzOrzan, FrancescaChlichlia, KaterinaOrzechowski, ArkadiuszChmielarz, PiotrOrzelska-Górka, JolantaChmielewska, JoannaOsborn, MarkChmielewski, MichałOsborn, Stephanie L.Chmyrov, AndriyOsella, SilvioChng, Shu SinOsellame, Laura D.Cho, Hee JunOshi, MasanoriCho, Jae YoulOshima, ShunjiCho, JaehoonOshima, YusukeCho, JaehyungOsipova, ZinaidaCho, Je-YoelOsmanagic-Myers, SelmaCho, Jung SookOsório, DanielCho, KinsangOsorio, Elvia JanethCho, Kwang-JinOsornio, ÁlvaroCho, Nicole A.Ostaszewski, RyszardCho, SangyunOster, HenrikCho, Tae-JuOster, MichaelCho, Won KyongOstersetzer-Biran, OrenChockalingam, Sriram P.Ostos Marcos, Francisco JoséChoe, Eun SangOstrowski, DanielaChohnan, ShigeruOstrowski, MaciejChoi, Cheol YongOstróżka-Cieślik, AnetaChoi, Dong-KugOstuni, MarianoChoi, HojungOswald, StefanChoi, Hong-ilOsyczka, Anna MariaChoi, HosoonOszako, TomaszChoi, Hyun JinOtani, MasahiroChoi, InhoOteri, GiacomoChoi, Jae-HongOtero-Vinas, MartaChoi, Jeong JuneOthumpangat, SreekumarChoi, Jeong-HwaOtręba, MichałChoi, Pui WahOtsuguro, Ken-ichiChoi, SangchunOtsuka, IsaoChoi, Se-YoungOtsuka, YuzuruChoi, Soo-KyoungOtsuki, TakemiChoi, Tae GyuOttmann, ChristianChoi, Won HyungOttolenghi, SaraChoi, Young MinOtulak-Kozieł, KatarzynaCholeva, LukasÖtvös, KrisztinaCholkar, KishoreOtzen, DanielChong, Cher-RinOu, Hsiu-ChungChong, Gun OhOu, ShujunChong, Irene Yu-ShingOuchi, TakehitoChoo, Hyo-jungOugham, HelenChorianopoulou, Styliani N.Ouro, AlbertoChorostecki, UcielOuweland, Jody M. W. Van DenChoshi, TominariOuyang, XinhaoChou, Chih-HungOvádi, JuditChou, Feng-ChengOvecka, MiroslavChou, Tsung-HanOvergard, Aina-CathrineChoudhary, SanjeevOvsepian, Saak VChoudhuri, AvikOvsyannikov, A. S.Choudhuri, SubhadipOwczarczyk-Saczonek, AgnieszkaChoudhury, Swarup RoyOwen, CarolynChow, JamesOwen, RobertChow, Simon Kwoon-HoOwen, ShawnChow, Tahsin J.Oxford, JuliaChowdhury, FarhanOyarzabal, AlfonsoChowdhury, IndrajitOyoshi, TakanoriChowdhury, NityanandaOza, JavinChowdhury, RatulOzaki, IwataChowdhury, SabihaÖzdinler, P. HandeChoy, Francis Y.M.Ozeki, YasuhiroChrist, TorstenOzga, AndrewChristensen, Jørn BOzretić, PetarChristensen, Lars P.Pabbidi, Reddy MChristenson, Lane K.Pabla, NavjotChristians, ElisabethPabst, Andreas MaxChristie, GrahamPacal, LukasChristodoulou, Maria-IoannaPacella, ElenaChristoffers, MichaelPacelli, ConsigliaChristopoulos, PetrosPachulska-Wieczorek, KatarzynaChristou, Georgios A.Paci, MaurizioChristov, Nikolai K.Pacia, Marta Z.Chronakis, NikosPacifici, LucianoChroni, AngelikiPacifico, Francesco M.Chrousos, George P.Pacifico, SeverinaChrysanthopoulou, AkriviPaciolla, CostantinoChtarbanova-Rudloff, StanislavaPacios, Luis F.Chu, ChenggenPackiam, Vignesh T.Chu, Dinh ToiPaço, AnaChu, I-MingPacquelet, AnneChu, Shi-JyePacurari, MaricicaChu, Sin TakPaczesny, JanChu, XiangpingPadan, EtanaChuah, Seng-KeePadanilam, BabuChuang, Hung-YiPadera, TimothyChuang, Ming-LungPadhi, Bhaja KrushnaChuang, Wan-LongPadhi, SoumeshChubanov, VladimirPadial-Molina, MiguelChubarev, Vladimir N.Padro, TeresaChueh, Pin JuPaduch, RomanChulanov, VladimirPadula, Matthew P.Chul-sung, HuhPădureanu, VladChun, Kyung-SooPaduszynska, MalgorzataChung, Bo YoungPae, Eung-KwonChung, Byung MinPafili, KalliopiChung, Chi-LiPagán, IsraelChung, Ching-HuPagani, StefaniaChung, GeehoonPagano, AlessandraChung, HakjaePagano, BrunoChung, HsiaohangPagano, CinziaChung, Hwan-SuckPagano, DuilioChung, Jay H.Pagano, LucaChung, JayongPagano, MarioChung, Ki WhaPagano, StefanoChung, KwiMiPage, Richard C.Chung, Man-KyoPageau, KarineChung, Tae NyoungPagella, PierfrancescoChung-Davidson, Yu-WenPagenstecher, AxelChungying, TsaiPagliai, Fernando A.Chwalek, KarolinaPagliarani, ChiaraChwalibóg, AndréPagliardini, LucaChwil, MirosławaPagnotta, Mario A.Chworos, ArkadiuszPagonopoulou, OlgaCiaccio, MarcelloPagotto, SaraCiacka, KatarzynaPagter, MajkenCiafrè, Silvia AnnaPahwa, RomaCiancetta, AntonellaPaidi, Santosh KumarCianci, AntonioPaik, TaejongCianciolo, GiuseppePaine, AnantaCianflone, EleonoraPaino, FrancescaCiani, MaurizioPais, Maria SaloméCiarmiello, LoredanaPais, PedroCiavarella, CarmenPajak, BeataCicala, CarlaPajares, MartaCicalini, IlariaPakdel, FarzadCiccarelli, MichelePaknahad, AliCicchini, CarlaPal, DebjaniCiccodicola, AlfredoPal, KrishnenduCiccone, LidiaPál, MagdaCichowska-Kopczyńska, IwonaPal, RumpaCiciliot, StefanoPal, SangitaCiclitira, PaulPalacios Calvo, JoseCicone, FrancescoPalacios, Isabel M.Cid, Victor JPalacios, JavierCid-Arregui, AngelPalacios, José-ManuelCielecka-Piontek, JudytaPaladini, Maria SerenaCiemerych-Litwinienko, AnnaPaladino, SimonaCiereszko, IwonaPalage, MarianaCiesielska, AnnaPalani, ChitraCiesielski, WojciechPalaniappan, BalasubramanianCifelli, PierangeloPalanisamy, ArulselvanCifone, Maria GraziaPałasz, ArturÇiftçi, Halil İbrahimPalazzo, BarbaraCifuentes, DanielPalazzo, EnzaCigana, CristinaPalese, Luigi LeonardoCignarella, AndreaPalestini, PaolaCil, OnurPalicka, VladimirCilurzo, FelisaPalini, SimoneCimmino, GiovanniPaliogiannis, PanagiotisCimpean, Anca-MariaPali-Schöll, IsabellaCîmpean, AnişoaraPalko-Labuz, AnnaCinelli, PatriziaPall, MartinCiobica, AlinPallag, AnnamáriaCiofi-Baffoni, SimonePallardó, Federico V.Cione, ErikaPallàs Lliberia, MercèCiornea, Elena TodirascuPalle, KomaraiahCipak Gasparovic, AnaPallotta, IsabellaCipak, LubosPallottini, ValentinaCipolloni, LuigiPalm, Gottfried J.Ciranna, LuciaPalma, Angelina S.Cirillo, GiuseppePalma, JoséCiriza, JesusPalma, Paulo JorgeCirmi, SantaPalmer, AlanCiszewski, Wojciech MichałPalmer, MichaelCitelli, MartaPalmer, Nathan A.Civiero, LauraPalmer, Timothy MartinCiz, MilanPalmerini, MariaClanchy, FelixPalmieri, ErikaCłapa, TomaszPalmieri, FerdinandoClaridge, Jolyon KPalmieri, GiuseppeClarimon, JordiPalmirotta, RaffaeleClark, Barbara JPalmisano, AlidaClark, GeorginaPalmour, RobertaClark, Natalie MPalomba, LetiziaClarke, AnthonyPalomo-Ríos, ElenaClarke, ChristopherPalou, MarionaClarke, JonathanPalsamy, PeriyasamyClarke, Lane L.Palsdottir, VilborgClarke, PennyPaluch, Andrew S.Claro, CarmenPalumbo, DomenicoClassen, Carl FriedrichPalumbo, GiuseppeClement, AlbrechtPalumbo, Vincenzo DavideClement, CristinaPaluri, RaviClement, JoachimPalus, KatarzynaClément, NathaliePaluszczak, JarosławClifton, NicholasPaluzzi, Jean-Paul V.Clifton, PeterPamarthy, SahithiCliment Salarich, MontserratPamir, NathalieClodt, Juliana IsabelPampaloni, FrancescoClose, DanPan, Chun-HsuCluzeau, ThomasPan, HaihuaCobbs, CharlesPan, Hung-ChuanCobley, JamesPan, JiayiCoccaro, NicolettaPan, JingCocco, StefaniaPan, MingguiCocovi-Solberg, David J.Pan, Ming-KaiCoelho, Antonio Victor CamposPan, Tai-longCoelho, RicardoPan, Tung-MingCoelhoso, IsabelPan, XiaoyueCofelice, MartinaPan, YangangCogato, AlessiaPan, Ying-xianCognasse, FabricePan, YoulianCohen, GuyPanara, FrancescoCohen, Justus B.Panasiewicz, GrzegorzCohen, Michael S.Panatala, RadhakrishnanCoiffard, Laurence J MPanchapakesan, BalajiCojocaru, FlorinaPanda, Amaresh C.Coker, Olabisi OluwabukolaPanda, KaushikColangelo, DonatoPanda, SantoshColanzi, AntoninoPandey, Dhananjay K.Colasante, GaiaPandey, PankajColasanti, RobertoPandey, SantoshColcombet, JeanPandiyan, ThangarasuCole, David K.Pandolfi, LauraCole, Kathryn E.Pandolfini, TizianaCole, MarshaPandya, Anshul A.Coleman Jr, Leon GarlandPanegyres, Peter K.Coleman, CynthiaPanek, JaroslawColeman, Jonathan A.Paneni, FrancescoColeman, MichaelPaneru, BamColeman, Mitchell C.Panga, JaipalColeman, Nichola J.Pangestu, MulyotoColeman, PaulPangrazzi, LucaColetta, MassimoPaniagua, CandelasColitti, MonicaPankonien, InesColl, MonicaPankotai, TiborColla, EmanuelaPankratova, StanislavaCollart-Dutilleul, Pierre-YvesPannaccione, AnnaColleluori, GeorgiaPanos, Anthony L.Collina, SimonaPanos, Georgios D.Collins, NicholasPanov, KonstantinCollongues, NicolasPanova-Noeva, MarinaCollura, AdaPant, BishweshwarColnaghi, LucaPantaleo, AntonellaCologna, StephaniePantea Stoian, AncaColombatto, PieroPanteris, EmmanuelColombeau, LudovicPantham, PriyadarshiniColonna, GiovanniPanuzzo, CristinaColuccia, MauroPanwar, PriyankaColussi, SilviaPanyutin, Igor G.Comai, GiorgiaPanzarasa, GuidoCombaret, LydiePanzer, SimonCombarnous, YvesPao, Ping-ChiehCombet, ChristophePaolella, GaetanaComelli, ElenaPaolini, MorenoComez, LuciaPaolo Vincenzo, PedoneComincini, SergioPapaccio, FedericaComizzoli, PierrePapaccio, GianpaoloCommault, Audrey S.Papadaki, CharaComoletti, DavidePapadimitriou, Dimitra N.Conan, FrancoisePapadopoulos, DimitriosConceição, NatérciaPapageorgiou, AnastassiosConciatori, FabianaPapageorgiou, Anastassios C.Conde, CarlosPapageorgiou, George Z.Conde, JavierPapagerakis, PétrosConfaloni, Anna MariaPapaioannou, Vasilios EConfavreux, CyrillePapalois, VassiliosConigliaro, AlicePapamitsou, TheodoraConiglio, Salvatore J.Papanas, NikolaosConnell, GregoryPapanikolaou, EleniConnell, Terry D.Paparella, Ashleigh S.Connor, MarkPapenbrock, JuttaConsolati, GiovanniPapi, PieroConsole, LaraPapini, AlessioConsonni, Francesca MariaPaplinska, MagdalenaConstantinescu-Aruxandei, DianaPaplińska-Goryca, MagdalenaConstantinos, PapagiannitsisPapoutsoglou, PanagiotisConstantin-Teodosiu, DumitruPapp, AndrásContaldo, NicolettaPapp, BélaConte, ClaudiaPapp, IstvánConte, IvanPappas, Maria L.Conte, MariarosariaPappone, CarloConteduca, VincenzaPapp-Wallace, Krisztina M.Conti, GiovanniPaquette, BenoitConti, StefaniaPar, GabriellaConti, ValeriaParajuli, NirmalaContino, MarialessandraParajuli, RajendraContreras, OsvaldoParanjape, AnuragContreras, Rubén G.Parashar, DeepakContursi, AnnalisaParaskeva, EfrosyniConvertini, PaoloParaskevas, George P.Conzuelo, FelipeParchem, Ronald J.Cook, LeahPardal, RicardoCooke, MeganPardini, BarbaraCoombs, MelaniePardo, FatimaCoon, Steven L. Parekh, AnantCooper, Elizabeth AParent, RomainCooper, KerryParente, PaolaCopolovici, Dana MariaParenti, IlariaCoppedè, FabioParida, SheetalCoppola, VincenzoParikesit, Arli AdityaCoquerel, DavidParimon, TanyalakCoras, RoxanaParinandi, NarasimhamCorazzari, MarcoParis, DeboraCorbet, CyrilParis, FrancoisCorbo, VincenzoParisi, KathyCorcoran, JacobPark, Ah-MeeCorcoran, JenniferPark, Byung-WookCordani, MarcoPark, Chan HoCordani, NicolettaPark, DaehoCordano, ChristianPark, Edwards A.Cordaro, MarikaPark, EujinCordaro, MassimoPARK, Eun JeongCordeddu, VivianaPark, Eun-MiCordero-Llana, ÓscarPark, GyungsoonCordes, NilsPark, Han-ACormio, AntonellaPark, Hyun-WooCornelius, DenisePark, Jae-BongCornillet, MartinPark, JeewoongCorrado, ChiaraPark, Ji-WonCorrado, GiandomenicoPark, JohnCorrado, GiovanniPark, Jong KookCorre, SébastienPark, Jong-SeokCorreia Coelho, Ana CláudiaPark, Joo-CheolCorreia, Sónia C.Park, JoongkyuCorreia-de-Sá, PauloPark, Jun-BeomCorridore, DenisePark, JunseongCorsalini, MassimoPark, JunsooCorsaro, AlessandroPark, Junyoung O.Corsaro, Maria MichelaPark, Kwang HwanCorso, GiovanniPark, Ky YoungCortay, HélènePark, KyeongsoonCortes, Andres J.Park, Kyung-soonCortés, Kamila CaraballoPark, Kyu-SangCortes, NataliePark, Myoung-HwanCorthay, AlexandrePark, Phil JuneCorti, ChiaraPark, Phun BumCorti, OlgaPark, Sang WonCortis, PierluigiPark, Sang-WookCorvis, YohannPark, See-HyoungCos, PaulPark, Shin-HyungCosenza, MariaPark, Sung-JoonCoseri, SergiuPark, WansuCosín-Roger, JesúsPark, Won MinCossu, DavidePark, Won SoonCosta, ClotildePark, Yong-DooCosta, JuliaParkar, Shanthi G.Costa, MarcelloParker, Daniel M.Costa, RaquelParker, EdwardCosta, RobertoParker, LewanCosta, RuiParker, Steven L.Costa, Vera MarisaParkkila, SeppoCosta, VivianaParkkita, SeppoCostantini, ClaudioPark-min, Kyung HyunCostantini, EricaParks, RuthCostantino, ClaudioParks, William C.Costa-Pinto, Ana RitaParl, FritzCostarelli, LeopoldoParlatescu, IoaninaCostea, Daniela ElenaParnell, Laura K.S.Costello, EithneParnetti, LucillaCostoya, Jose A.Paronetto, Maria PaolaCote, PatriceParrales, AlejandroCotella, DiegoParri, RheinCotoras, DarkoParrington, JohnCotter, Thomas G.Parrish II, Richard H.Cottini, FrancescaParrizas, MarcelinaCouce, María D.Parry, GeraintCouce, María LuzParsons, Ryan G.Couch, YvonnePartyka, AgnieszkaCoudert, Amélie EParus, AnnaCougnoux, AntonyPârvu, MarcelCoulouarn, CédricParzonko, AndrzejCoulson-Thomas, Vivien JanePasantes, JuanCoumans, Joëlle V. F.Pasca, SergiuCoureux, Pierre DamienPascal, LauraCourt, Karem A.Pascal, Steven M.Courtois, GillesPaschalis, Eleftherios I.Cousins, FionaPasciu, ValeriaCoussons, PeterPascual, Carmen María GarcíaCoutant, FrédéricPascual, María BelénCoutinho, PaulaPascut, DevisCoutinho, Paulo José GomesPasetto, AnnaCoutinho-Silva, RobsonPashov, AnastasCouttas, Timothy A.Pasin, FabioCouture, RéjeanPasini, LuigiCouvineau, AlainPasquariello, RolandoCovarrubias, AlejandraPasquet, Jean-MaxCovo, ShayPasquinelli, GianandreaCowman, MaryPassali, Giulio CesareCox, MichaelPassos, MarisaCox, Richard Aras (Aaron)Pasternak, AlexanderCozza, GiorgioPasternak, TarasCozzani, EmanuelePasternak, Taras P.Craig, MorganPastey, ManojCram, Erin JPastor, WilliamCravero, Maria CarlaPastor-Anglada, MarçalCrawford, Bryan D.Pastore, AnalisaCrawford, Emily D.Pastores, Gregory M.Crawford, LisaPastorino, PaoloCreasy, CarethaPastuch-Gawolek, GabrielaCrepaldi, TizianaPasupuleti, VisweswaraCrescenzi, AnnaPatarrão, RitaCrescenzi, CarloPatching, Simon GCrescenzi, MarcoPátek, MiroslavCrescioli, ClaraPatel, BhargavCrespan, EmmanuelePatel, DevenCrespo, FranciscoPatel, HardikkumarCrespo, VicentePatel, NibeditaCrestani, MaurizioPatel, NilkanthCretoiu, DragosPatel, NrupaliCrevenna, AlvaroPatel, RakeshCrevillén, PedroPatenge, NadjaCribbs, AdamPateras, Ioannis S.Crichton, Robert R.Paternoster, SilvanoCridge, AndrewPathak, BhuvanCrisan, LuminitaPathak, DhrubaCrisp, PeterPathipati, PraneetiCrispi, StefaniaPatik, JoradanCrisponi, GuidoPatil, DeepakCrisponi, LauraPatil, Mahesh D. Cristescu, RodicaPatil, Swati J.Cristofani, RiccardoPatila, MichaelaCristofaro, Raimondo DePatini, RomeoCroce’, Lory SaveriaPaton, ChadCrocetti, LetiziaPaton, Madison C. B.Crona, DanielPatra, AmiyaCrosas, BernatPatrauchan, Marianna A.Crosatti, CristinaPatrick Lajoie, Patrick LajoieCroset, MartinePatrignani, PaolaCross, MichaelPatrinos, GeorgeCrotti, TaniaPatrizi, AnnalisaCrozatier-Borde, MichelePatrizi, BarbaraCrucho, CarinaPatro-Małysza, JolantaCrucitta, StefaniaPatrone, CesareCrupi, Mathieu J.F.Patruno, CataldoCrusats, JoaquimPatrussi, LauraCrus-Topete, DianaPatsali, PetrosCruz, CarlaPattammattel, AjithCruz, Homero Reyes De LaPatten, Shunmoogum A.Cruz, Juan C.Patterson, JenniferCruz, RodrigoPatterson, StevenCruz, Rui M.S.Patti, AlessandroCruz-Lopez, OlgaPaudel, Keshav RajCsabafi, KrisztinaPaul, AshimCsámpai, AntalPaul, MatthewCsepany, TundePaul, ParameswariCsernok, ElenaPaul, SudipCsík, GabriellaPaul, YaswenCsiszar, AgnesPaulo, AntónioCsősz, ÉvaPaulusma, Coen C.Cuajungco, MathPavanello, ChiaraCubellis, MariaPavani, Krishna ChaitanyaCubero, Francisco JavierPavankumar, TheethaCubo, LeticiaPavlidis, EfstathiosCucchiarini, MagaliPavlik, EdwardCucinotta, FrancisPavlik, Edward J.Cuenda, AnaPavlin, MaticCuesta, CandelaPavlínková, GabrielaCui, HaitaoPavlos, Nathan JohnCui, QingbinPavlov, TengisCui, RongfengPavlov, Youri ICui, TiantianPavlovic, NebojsaCui, YanxiangPavo, NoemiCui, YePavšič, MihaCui, ZhouqiPawar, Shrikant D.Cuif, Jean-PierrePawar, SnehalataCukrowska, BoženaPaweł’kowicz, Magdalena ECullen, PaulPawelec, GrahamCumbo, CosimoPawlak, AleksandraCumming, Mathew HoaniPawlak, DariuszCummings, Brian S.Pawlak, KrystynaCummins, TheodorePawlak, PiotrCunha, Rodrigo A.Pawlik, AndrzejCunnane, StephenPawlik, AnnaCunningham, AmyPawlikowska-Pawlęga, BożenaCunningham, Madeleine W.Pawlikowski, MarekCunningham, Margaret RosePawlowski, KatharinaCuppoletti, JohnPawłowski, TomaszCurci, Pasquale LucaPayne, AnnetteCurcic, MarijanaPazdro, RobertCurcio, AlbertoPeacock, ChristopherCurran, ChristinePearce, NeilCurreli, SabrinaPearce, StephenCurrò, MonicaPearl, Christopher A.Curtale, GraziellaPearring, JillianCurti, AntonioPec, MartinCurtin, James F.Pečenková, TamaraCurtis, AnnePeche, VivekCurtis, Brian R.PECHEUR, EveCurtis, MarionPecho-Vrieseling, ElineCusick, Kathleen D.Pecina-Slaus, NivesCustodio, CatarinaPeck, AmmonCutler, Christopher WPecori, RiccardoCutler, Mary LouPecqueur, ClaireCutone, AntimoPecyna, PaulinaCuypers, BartPedemonte, NicolettaCvetkovska, EmilijaPedersen, HenrikCycoń, MariuszPedeux, RémyCyganek, LukasPeditto, MatteoCymbaluk, AnetaPedone, E. M.Cytlak, UrszulaPedrazzini, EmanuelaCzaja-Bulsa, GrażynaPedron, SaraCzajkowski, RobertPedrosa, PedroCzakó, MártaPedroza-Garcia, Jose-AntonioCzapik, AgnieszkaPeelman, FrankCzaplewski, CezaryPeeples, MarkCzarna, MalgorzataPeery, RhiannonCzarnecka, AnnaPeeters-Scholte, CachaCzechowski, Piotr OskarPeeva, VioletaCzeiter, EndrePegolo, EnricoCzerwinska, MonikaPeifer, ChristianCzerwiński, MarcinPeigneur, SteveCzigany, ZoltanPeinado, María ÁngelesCzogalla, AleksanderPeinelt, ChristineCzuczwar, StanisławPeirats-Llobet, MartaCzystowska-Kuzmicz, MalgorzataPeiró, SandraCzyz, AnnaPeitsidis, PanagiotisCzyż, JarosławPeitzsch, ClaudiaCzyż, KatarzynaPeixoto, CristinaCzyz, MalgorzataPejchal, JaroslavCzyżewski, KrzysztofPejin, BorisCzyżnikowska, ŻanetaPekkanen-Mattila, MariD’ Acunto, MarioPěknicová, JanaD’Acquarica, IlariaPekrun, ArnulfD’Acquisto, FulvioPeláez-García, AlbertoD’Agostino, CarminePelagalli, AlessandraD’Agostino, MassimoPellegrini, MarikaD’Agostino, PaulPellerino, AlessiaD’Agostino, Vito GiuseppePelletier, EmilienD’Aguanno, SimonaPelliccioni, PaoloD’Alessandra, YuriPeluffo, HugoD’alessio, AlessioPeluso, Marco E. M.D’Alò, FrancescoPeña Quintana, LuisD’Ambrosi, NadiaPeña, CristinaD’Amelia, VincenzoPena, Maria MarjoretteD’Amelio, RaffaelePeñafiel, RafaelD’Amico, RamonaPeña-Llopis, SamuelD’Amora, UgoPenchev, HristoD’anca, MariannaPendin, DianaD’Angelo, ArmandoPendyala, Gurudutt ND’Angelo, LiviaPenezic, AnaD’Angelo, MichelePeng, Chung-KanD’Anneo, AntonellaPeng, CongyueD’arcy, PádraigPeng, HanD’Ardes, DamianoPeng, HuiD’Argenio, ValeriaPeng, I-ChenD’Autreaux, BenoitPeng, XianjunD’Costa, VanessaPeng, Xianlu LauraD’Elios, Mario MilcoPeng, XuD’Haese, PatrickPeng, Yi-JenD’Onofrio, GraziaPeng, Ying-JieD’Orazi, ValerioPeng, ZeD’Orsi, BeatricePeng, ZhaoD’Souza, Rochelle C. J.Penke, BotondD’Ursi, Anna MariaPennerman, KaylaDa Costa, Rui M. GilPennicooke, BrentonDa Lage, Jean-LucPenning, LouisDa Silva E Silva, DanielPennisi, Elena MariaDa Silva, Anabela BernardesPenno, MeganDa Vià, MatteoPenson, PeterDabbou, SihemPentikäinen, UllaDabdoub, AlainPentzold, StefanDabiri, YasaminPenzo, MariannaDabrowski, FilipPepe, JessicaDabrowski, Michal J. Pepeu, GiancarloDacarro, GiacomoPerálvarez-Marín, AlexDacheux, Jean-LouisPeran, IvanaDachineni, RakeshPerdas, EwelinaDachs, GabiPerdigão, NelsonDaci, ArmondPerdomo, GermanDada, LauraPerdomo, JoseDadachova, EkaterinaPerego, CarlaDadashi-Silab, SajjadPEREIRA LEMOS, JuliaDagmara, MalinaPereira, Cidália AlmeidaDagur, Raghubendra S.Pereira, ClaudiaDahan, AlbertPereira, Cláudia Maria F.Dahiya, RajivPereira, DavidDahl, Jan-UlrikPereira, FilipeDai, Chia-YenPereira, FlorbelaDai, JiangkunPereira, LeonelDai, WeiPereira, LeonorDaimi, HouriaPereira, Maria De LourdesDajas-Bailador, FedericoPereira, Nelson A. M.Dal Ben, MatteoPereira, PatriciaDal Corso, AlbertoPereira, SofiaDal Prá, IlariaPereira, SóniaDalal, VijitPerepechaeva, Maria L.Dalbeni, AndreaPerera, Chamini J.Dale, Erica A.Perera, LalithDalhaimer, PaulPerera, OmaththageDali-Youcef, NassimPérez De La Vega, MarcelinoDall’Olio, FabioPérez Puyana, Víctor ManuelDalla Pozza, ElisaPérez Tur, JordiDalla, EmilianoPerez, GuillermoDallinger, ReinhardPerez, Juan J.Dama, MuraliPérez, Ruth G.Damalanka, VishnuPérez, SalvadorDamaziak, KrzysztofPerez, SergeDamberger, FredPérez-Badia, RosaDamerval, CatherinePérez-Caballero, LauraDamia, GiovannaPérez-García, SeleneDamiani, DanielaPerez-Hernandez, ConcepciónDamiani, GiovanniPerez-Mendez, OscarDamiano, FabrizioPérez-Pascual, DavidDamiano, SaraPerez-Pe, RosauraDamkier, HellePérez-Ros, PilarDandawate, PrasadPérez-Sala, DoloresDandekar, ThomasPérez-Sánchez, CarlosDane, FennyPerez-Siles, GonzaloDaniel, YosephPerez-Stable, CarlosDaniela, AilincaiPérez-Torras, SandraDaniele, AuroraPérez-Vázquez, VictorianoDaniellou, RichardPergament, EugeneDanielsen, MichaelPeriasamy, RameshDanilenko, MichaelPerin, CeciliaDanilenko, VNPeriyasamy, Arun PrakashDaniluk, UrszulaPeriyasamy, PalsamyDanisa, Olumide A.Perkeybile, Allison M.Danisovic, LubosPerkin, Lindsey C.Danková, ZuzanaPerkins, AndrewDannaoui, EricPerkovic, IvanaDansette, Patrick M.Perkovic, Matea NikolacDantuma, NicoPerl, AndrasDanyluk, JeanPerlikowski, DawidDar, Mohd SaleemPerlin, Michael H.Dar, Wasim A.Perluigi, MarziaDaras, GerasimosPermyakov, EugeneDardis, AndreaPernisová, MarkétaDario, BrunettiPero, RaffaelaDark, MichaelPeronnet, FrederiqueDaroszewski, JacekPerouansky, MishaDarras, AnastasiosPerreault, JonathanDarreh-Shori, TaherPerreault, Marie-ClaudeDarst, Seth A.Perrella, GiorgioDartt, DarlenePerricone, UgoDarvell, BrianPerrier, Jean-FrancoisDas, AbhirupPerrino, EnricoDas, AditiPerron, HervéDas, ArabindaPerrone, Anna MyriamDas, Arup KumarPerrone, GiancarloDas, JoydipPerrone, Marco AlfonsoDas, Kumuda C.Perrone, SerafinaDas, RupaliPerrucci, Gianluca LorenzoDas, SabyasachiPerry, RachelDas, SubhaPerše, MartinaDas, SudipPershina, O. V.Das, ViswanathPersico, MarcoDasgupta, KasturiPersson, JennyDasgupta, PiyaliPerucca Orfei, CarlottaDash, BirajaPerucka, IrenaDash, ChandravanuPerut, FrancescaDash, Ranjeet PrasadPeruzzi, FrancescaDastsooz, HassanPeruzzo, RobertaDaszkowska-Golec, AgataPes, Giovanni MarioDatki, ZsoltPeschke, KatharinaDatta, Anita N.Pešić, MilicaDatta, ArpitaPeška, VratislavDatta, KausikPest, Michael AndrewDatta, PoulamiPestell, RichardDatta, RupsaPestov, AlexanderDatta, SandipanPestov, DimitriDaubon, ThomasPeták, IstvánDaudé, DavidPetejová, NadeždaDaugrois, Jean-HeinrichPéter, BeatrixDave, SandeepPeter, FranciscDaveluy, StevenPeter, KarlheinzDaverey, AmitaPeters, DonnaDavid, LaurePeters, JudithDavid, RobertPeters, MaireDavidova, IrenePetersen, OleDavidson, ColinPeterson, LarrynDavidson, CristinPetersson, KristofferDavie’re, Jean-MichelPethő, ZoltánDavies, KevinPetit, Patrice X.Davies, NigelPetitjean, AnneDavis, Erica E.Petra I., LorenzoDavis, Keith R.Petrache, IrinaDavis, Mellar P.Petralia, Maria CristinaDavis, Michael E.Petreaca, Ruben CDavis, Randall L.Petrie-Hanson, LoraDavis, Richard P.Petrikaite, VilmaDavoodi, PooyaPetrillo, AngelicaDavtyan, AramPetrillo, SaraDavuluri, GangaraoPetrinovic, Marija-MagdalenaDawood, Mahmoud A.O.Petrof, Basil J.Dawra, RajinderPetropoulos, Spyridon ADay, Andrew S.Petrosillo, GiuseppeDay, DavidPetrosyan, ArmenDay, Regina M.Petrov, Alexey M.Dayal, SanjanaPetrov, AntonDaza-Navarro, PaulaPetrova, MarinaDe Almeida, Rodrigo CoutinhoPetrovic, ZeljkaDe Almodóvar, José Mariano RuizPetrut, BogdanDe Amicis, FrancescaPetrzik, KarelDe Angelis, AntonellaPetta, IoannaDe Araujo, ElvinPetti, CarloalbertoDe Aza, Piedad N.Pettignano, AlbertoDe Baaij, JeroenPetukhov, Dmitriy I.De Barrios, OriolPeulen, OlivierDe Bellis, LuigiPeverali, FiorenzoDe Benedetti, Pier G.Peviani, MarcoDe Benedetto, AnnaPey, Angel L. De Bernardi Schneider, AdrianoPeyton, David H.De Biase, DanielaPezzella, AlessandroDe Bock, KatrienPezzilli, RaffaeleDe Breyne, SylvainPezzuto, AldoDe Carlis, LucianoPfannschmidt, ThomasDe Caro, VivianaPfeffer, Lawrence M.De Carvalho, Carla C. C. R.Pfeffer, PeterDe Chiara, FrancescoPfeffer, UlrichDe Chiara, LetiziaPfeil, AlexanderDe Cock, DiederikPfister, BarbaraDe Falco, ElenaPham, Anh TungDe Falco, ValentinaPham, ThaoDe Felice, FrancescaPhan, Thanh KhaDe Francesco, Ernestina MariannaPhan, Thi Tuong VyDe Francesco, FrancescoPhang, JamesDe Francesco, RaffaelePhilipp, ManfredDe Francisco, SeylaPhilips, ThomasDe Geest, BartPhillips, Kevin J.De Graaf, CoenPhillips, TycelDe Guia, Roldan M.Philpott, MichaelDe Jesus Pereira Godinho Velada, IsabelPi, JiangDe Jong, StevenPiacenza, ElenaDe Jonge, HugoPiaggio, GiuliaDe Julián Ortiz, JesusPiane, MariaDe Koning, LeannePiasecka, AgnieszkaDe Kruif, JohnPiasecka, MałgorzataDe Kruijff, Robin M.Piątczak, EwelinaDe La Fuente, HortensiaPiatek, JacekDe La Lastra, José Manuel PérezPiatkevich, Kiryl D.De La Luz Garcia-Hernandez, MariaPiattelli, AdrianoDe La Puente, PilarPiazza, FrancescoDe La Torre, Jose GarciaPiazza, Gary A.De La Vega, José Manuel GarcíaPiazzon, M. CarlaDe Las Heras Montero, Javier AdolfoPicado, CésarDe Lellis, LauraPicard, DidierDe Lorenzo, Mariana SPicca, Rosaria AnnaDe Luca, AnastasiaPiccinin, ElenaDe Luca, GiuseppinaPiccionello, Antonio PalumboDe Luca, PaolaPiccirillo, RosannaDe Luna-Bertos, ElviraPiccoli, MarcoDe Marchi, TommasoPiccolo, MarialuisaDe Martin, RainerPichler, VerenaDe Martinis, MassimoPicimbon, Jean-FrançoisDe Martino, MarcoPickering, Andrew M.De Matteis, RitaPickering, RaeleneDe Matteis, ValeriaPico Alfonso, Antonio M.De Meyts, PierrePicollet-D’hahan, NathalieDe Mieri, MariaPicozza, MarioDe Miglio, Maria RosariaPidot, Sacha J.De Moor, CorneliaPidoux, GuillaumeDe Munain, Adolfo LópezPiechota, AleksandraDe Nisco, Nicole J.Piechowicz, LidiaDe Nova, Pedro J.G.Pieczonka, Adam M.De Oliveira Carvalho, Carla RobertaPiedras, PedroDe Oliveira, CláudioPiegat, AgnieszkaDe Paepe, BoelPiekiełko-Witkowska, AgnieszkaDe Paiva, Cintia S.Piemonte, FiorellaDe Palma, RaffaelePierce, David M.De Paola, DomenicoPierce, DwightDe Paolis, AngeloPierdomenico, Johnny DiDe Pasquale, ValeriaPiergentili, RobertoDe Pastena, MatteoPieroni, MarcoDe Pauw, EdwinPierozan, PaulaDe Pinto, MariaPierpaoli, MattiaDe Rasmo, DomenicoPierreeiche, OlivierDe Re, VallìPierri, Ciro LeonardoDe Reijke, T.M.Pieters, Alejandro J.De Rienzo, AssuntaPieters, RolandDe Robertis, MariangelaPietras, AlexanderDe Rocquigny, HuguesPietropaolo, AdrianaDe Rooij, DirkPietruszewska, WiolettaDe Rosa, Maria CristinaPietrzak, BogusławaDe Rosa, VivianaPievani, Alicede Rosales, Rafael T.M. Pignataro, GiuseppeDe Silva, FranklynPignatti, PatriziaDe Simone, AngelaPignone, DomenicoDe Smaele, EnricoPigossi, Suzane CristinaDe Sousa-Coelho, Ana LuísaPiiper, AlbrechtDe Spiegelaere, WardPijnappel, W.W.M. PimDe Stefano, RosaPikija, SlavenDe Summa, SimonaPiknova, BarboraDe Teresa, Jose-MariaPikula, KonstantinDe Tullio, Mario C.Pikuła, MichałDe Vicente, Juan CarlosPilarsky, ChristianDe Vita, AlessandroPilato, FabioDe Vita, DanielaPileczki, ValentinaDe Vito, DanilaPiliponsky, Adrian M.De Vos, JohnPillai Raju, RaghavanDe Vries, CarliePillozzi, SerenaDe Vries, TeunPilmane, MāraDe Waard, MichelPilon, MarinusDe Waard, VivianPilones, Karsten AndersonDe Zinis, Luca Oscar RedaelliPilot, GuillaumeDe Zotti, MartaPilot, MalgorzataDe, PradipPilu, RobertoDe, TanimaPiluso, GiulioDealy, Caroline N.Piluzza, GiannellaDean, MichaelPilvankar, MinuDean, Olivia MPimentel, MadalenaDeans, RebeccaPimerzin, AlekseyDeb, SubrataPin, FabrizioDe-Bandt, Jean-PascalPiña Capó, Mª NievesDebatin, Klaus-MichaelPina, AnaDebela, Ahmed M.Pina, JoãoDębowski, DawidPinalli, RobertaDębska, BernardetaPiña-Oviedo, SergioDe-Chiara, LorettaPinar, AnitaDecorti, GiulianaPinart, ElisabethDecuypere, Jean-PaulPina-Vaz, CidaliaDed, LukasPindyurin, Alexey V.Dedhar, ShoukatPineau, PascalDedkova, Elena N.Pineault, NicolasDeelman, L.E.Pineda, Jose RamonDeep, GaganPinelli, MicheleDeepak, JanakiPiñero, ManuelDeftereos, GeorgiosPingault, LiseDegani, GadPingtao, DingDeganutti, GiuseppePinho, DianaDegola, FrancescaPini, TaylorDeGregorio, DaniloPinna, GrazianoDei, SilviaPinnapireddy, Shashank ReddyDeineko, Elena VictorovnaPinniger, GavinDeive, FranciscoPino-Yanes, MariaDeja, StanislawPinter, MatthiasDejeu, JérômePintilie, Grigore D.Dekker, MarloesPinto Da Silva, LuísDel Angel, Rosa MaríaPinto, DeesyDel Boccio, PieroPinto, GloriaDel Fattore, AndreaPinto, MafaldaDel Fresno Sánchez, CarlosPinto, SaraDel Gaudio, CostantinoPinto-Medel, MaríaDel Giacco, LucaPinzaru, IuliaDel Giudice, RitaPiorecky, MarekDel Hoyo Martínez, CarmenPiotrowska, EwaDel Prete, AnnalisaPiotrowska, KatarzynaDel Río Celestino, MercedesPiozzi, AntonellaDe-la-Casa-Almeida, MaríaPippenger, BenjaminDelannoy, PhilippePiqueras, LauraDeLaurier, AprilPires, Bruno R. B.Delebarre, ChristophePirkmajer, SergejDeleo, FrancescoPiro, Rosario M.Delfino, Domenico V.Pirola, LucianoDelgado Palacio, SusanaPirola, YuriDelgado, Daniel RicardoPirooznia, MehdiDelgado, MaríaPirozzi, Christopher J.Delgado-Esteban, MariaPirtoli, LuigiDelgado-Ruiz, RafaelPisani, AntonioDelhomme, NicolasPisani, Didier F.Delhommel, FlorentPisanu, ClaudiaDelicado, Esmerilda G.Pisanu, Maria ElenaDelisle, Jean-SébastienPiscopo, MarinaDelitala, AlessandroPiscopo, PaolaDeliyanti, DevyPisetsky, David S.Dell’Era, PatriziaPissas, GeorgiosDell’olmo, ElianaPistis, MarcoDella Bartola, MichelePistocchi, Anna SilviaDella Porta, GiovannaPistone, AlessandroDella Ragione, FulvioPita, SebastianDella Torre, EmanuelPitsillides, AndrewDella Torre, SaraPitt, JamesDellaGreca, MarinaPittalà, ValeriaDelle Monache, SimonaPittaluga, AnnaDellepiane, SergioPittman, KarinDellero, YounèsPitto, LetiziaDellis, OlivierPitucha, MonikaDello Ioio, RaffaelePitule, PavelDelmaghani, SedighehPiu, SahaDelorme, VincentPiunova, VictoriaDel-Rio Celestino, MercedesPiva, RobertaDeLuca, LuigiPiva, TerrenceDemartini, ChiaraPivac, NelaDembowski, JillPivarcsi, AndorDembska, Anna RenataPivoriunas, AugustasDeme, PragneyaPiwowarczyk, KatarzynaDemey, HaryPizzanelli, SilviaDemir, FatihPizzolanti, GiuseppeDeMirci, HasanPłachno, BartoszDemonbreun, AlexisPlacido, AntonioDempski, RobertPlacido, JerssonDemyanenko, Svetlana A.Placzek, William J.Demyanets, SvitlanaPlakht, YgalDemyda-Peyrás, SebastiánPlanas, MartaDen Blaauwen, TannekePlaneta, DiegoDenegri, BernardPlano, Laura DeDengler, FranziskaPlant, LeighDenham, MarkPlant, Timothy D.Deniaud, AurélienPlante, IsabelleDenina, MarcoPlastina, PierluigiDennington, SimonPlatania, ChiaraDenovan-Wright, Eileen M.Plate, LarsDensham, RuthPlatella, ChiaraDenti, Michela A.Platt, Donna M.Deore, Prashant S.Platta, HaraldDepalma, Ralph G.Pławski, AndrzejDepeint, FlorePlayford, Martin P.DePinho, Ronald A.Płaza, GrażynaDepping, ReinhardPlaza-Zabala, AinhoaDeptuła, MilenaPŁażek, AgnieszkaDerer, StefaniePlazinski, WojciechDergunov, SergeyPlazzer, John-PaulDeriu, Marco AgostinoPlech, TomaszDerkach, Kira V.Plecita, LydieDerkinderen, PascalPlenis, AlinaDerler, IsabellaPletser, VladimirDerwich, KatarzynaPletzer, DanielDesai, Amar P.Plíhal, OndřejDesai, AmritaPlíhalová, LucieDesai, ChandniPloetz, EvelynDesai, MoreshwarPloner, ChristianDesai, Umesh R.Plonka, PrzemyslawDesaubry, LaurentPlotka, MagdalenaDesbois, Andrew P.Plotkin, Lilian I.Descamps, GéraldinePlotnikov, Egor Y.Deschanels, XavierPłuciennik, ElżbietaDesforges, MarcPluta, RyszardDeshayes, FrédériquePluth, Janice M.Deshpande, ShayuPniewski, TomaszDesiderio, VincenzoPobbati, AjaybabuDeslouches, BerthonyPobiega, KatarzynaDesnos, ThierryPocha, ChristineDespotovic, InesPocheć, EwaDesriac, FloriePochet, RolandDesRochers, AnniePochwat, BartłomiejDessein, AlainPochynyuk, OlehDesselberger, UlrichPodar, KlausDettin, MonicaPoddelsky, AndreyDettmer, UlfPodnar, SimonDeuis, JenniferPodobnik, MarjetkaDeutsch, AlexanderPodust, Larissa M.Deutsch, MelaniePoeggeler, Burkhard H.Devarajan, PriyadharshiniPogacnik, LeaDevesa, JesúsPoggi, AlessandroDevillers, JamesPoggio, PaoloDevine, Patrick J.Poglitsch, MarkoDevireddy, Amith R.Pogocki, DariuszDevroye, CélinePogorielov, MaksymDevy, JeromePohjanvirta, RaimoDey, KamolPohjoismäki, Jaakko L. O.Dey, MadhusudanPohl, Carolina H.Dey, NandiniPohl, EhmkeDey, PriyankarPohl, Sebastian Ӧther-GeeDeyev, Igor E.Pohlkamp, TheresaDeyev, Sergey M.Poinsot, VerenaDhakal, DipeshPoirot, MarcDhakar, Nilesh KumarPojo, MartaDhakshinamoorthy, AmarajothiPóka, RóbertDhanasekaran, MuraliPoku, Rosemary A.Dhanasiri, AnushaPolacek, NorbertDhankher, Om ParkashPolak, AnnaDhasmana, AnupamPolak, Marta EwaDhindsa, SandeepPolakowski, Nicholas J.Dhooge, Ingeborg Johanna MariaPolańczyk, AndrzejDhungel, BijayPolano, MaurizioDi Agostino, SilviaPolanski, WitoldDi Bernardo, GiovanniPolari, LauriDi Bussolo, ValeriaPoldermans, DonDi Cesare Mannelli, LorenzoPolge, CécileDi Donato, MarziaPoli, AlessandroDi Fazio, PietroPoli, GiuliaDi Filippo, Ester SaraPolicar, ClotildeDi Filippo, MarziaPolidoros, AlexiosDi Fiore, AnnaPolimeno, LorenzoDi Franco, SimonePolina, IuliiaDi Gennaro, FrancescoPolini, BeatriceDi Giaimo, RossellaPolishchuk, PavelDi Giannatale, AngelaPolishchuk, RomanDi Gioia, Maria LuisaPolissidis, AlexiaDi Giovanni, GiuseppePolit, JustynaDi Giulio, CamilioPolitano, LuisaDi Liegro, ItaliaPolizzi, AlessandroDi Lorenzo, FlavianaPolkowska-Kowalczyk, LidiaDi Lorenzo, FrancescoPollastro, StefaniaDi Maro, AntimoPollok, Karen E.Di Martino, OrsolaPolly, PatsieDi Matteo, AdelePolticelli, FabioDi Matteo, SabinaPoltronieri, PalmiroDi Mauro, MariaPoluektova, LarisaDi Meo, IvanoPolverino De Laureto, PatriziaDi Mise, AnnaritaPolyakov, NikolayDi Nardo, FabioPomastowski, PawełDi Natale, ConcettaPombo, AnaDi Nisio, AndreaPomerantz, OriDi Paola, RosannaPommerrenig, BenjaminDi Pasqua, Laura GiuseppinaPompella, AlfonsoDi Pietro, LorenaPompili, ElenaDi Pietro, PaolaPoncet, DelphineDi Pizio, AntonellaPonnu, JathishDi Polo, AdrianaPonraj, PrabakaranDi Pompo, GemmaPons, MiquelDi Resta, ChiaraPontarotti, PierreDi Sansebastiano, Gian-PietroPonte, NunoDi Serio, FrancescoPontejo, Sergio MDi Sotto, AntonellaPontes, BrunoDi Tucci, ChiaraPontremoli, CarlottaDi Virgilio, FranciscoPoole, Angela Z.Di Zazzo, ErikaPoole, Jill A.Diaconeasa, ZoritaPoole, LeslieDiaferia, CarloPoór, PéterDiamantopoulou, PanagiotaPop, Laura AncuţaDias, Albino AlvesPop, LaurentiuDias, Daniel A.Pop, Oana LeliaDias-Ferreira, JoãoPopa, LacramioaraDiaz Hernandez, MiguelPopa, MarcelDíaz, EsperanzaPopa, Mircea IoanDiaz, Eva C.Pope, ChadDiaz, FranciscaPopeijus, Herman E.Diaz, MarvinPopelka, HanaDíaz-Encarnación, MontserratPopescu, Mihaela R.Diaz-Garcia, LuisPopgeorgiev, NikolayDiaz-Munoz, Manuel DPopolo, AdaDiaz-Rodriguez, PatriciaPopova, Alexandra A.DiBella, CalogeroPopova, AntoanetaDiCarlo, Andrea L.Popović Đorđević, JelenaDiccianni, MitchellPopp, HenningDickinson, Bryan C.Poppinga, SimonDickson, Benjamin D.Poradowski, DominikDidiášová, MiroslavaPorcheri, CristinaDidier Germain, ArnaudPorchia, MarinaDidier, ChristinePordea, AncaDidion, Sean P.Porokhovnik, LevDiederichs, SvenPorollo, AlekseyDiego, La MendolaPorras, IgnacioDiehl, LindaPorrelli, DavideDiehnelt, Chris W.Porres, JesusDiekwisch, Thomas G.H.Porrini, Esteban L.Diene, SeydinaPorro, ChiaraDienes, BeatrixPorta, ChiaraDiepold, AndreasPorta, HelenaDietrich, AlexanderPortelli, MarcoDietrich, OlafPorteu, FrançoiseDiez-Escudero, AnnaPortillo-Lara, RobertoDigiacomo, MariaPoschenrieder, CharlotteDignon, GregoryPosey, Avery D.Diharce, JulienPospíšilová, ŠárkaDijk, AaltPossidente, BernardDijk, FrederikePossik, EliteDijkhuizen, LubbertPostler, Thomas S.Dijkstra, Bauke W.Potaczek, Daniel PDika, EmiPotaniec, BartłomiejDikalov, SergeyPotekhin, AlexeyDikalova, AnnaPotenza, NicolettaDileep, VishnuPotes, YaizaDilley, RodneyPotiron, VincentDillman, AdlerPotokar, MajaDillman, Adler R.Potrykus, KatarzynaDIMA, Stefan-OvidiuPotter, HuntingtonDimaki, MariaPotter, JuliaDimarogona, MariaPotter, Pamela EDiMattia, GabrielPoudel, BarunDimberg, AnnaPoudel, Sher BahadurDimienescu, Oana GabrielaPouliot, RoxaneDimitriadis, StavrosPourbaghi Masouleh, MiladDimitrios, FessasPourcet, BenoîtDimitroff, Charles J.Pourié, GrégoryDimitroulas, TheodorosPourquier, PhilippeDimitrov, EugenePourrezaei, KambizDimova, TanyaPovelones, MeganDinca, ValentinaPowers, EvanDinel, Anne-LaurePowrózek, TomaszDinescu, SorinaPoyet, Jean-LucDing, LinPoynter, Sarah J.Ding, Ling-WenPozharskiy, EdvinDing, QingbaoPoznański, JarosławDing, YapingPozzi, CeciliaDing, ZhaojunPozzi, NicolaDingley, Andrew J.Pozzo, Eleonora DaDinh, Dzung H.Pozzobon, MichelaDinh, Quynh NhuPrabhu, Antony HeroldDinis, MárioPrabhu, LakshmiDinkova, Tzvetanka D.Prada, IlariaDinos, GeorgiosPradhan, DineshDion, Patrick A.Pradhan, SriharsaDir, JanPrado-Gotor, RafaelDiretto, GianfrancoPradotto, Luca GuglielmoDirsch, VerenaPrakapenka, AlesiaDisanza, AndreaPrakash, NilimaDisney, Matthew D.Prakash, SatyaDissanayaka, WarunaPramanik, ArindamDissel, StephanePramanik, AvijitDistasi, CarlaPramod, SreepriyaDite, TobyPranckeviciene, ErinijaDitengou, FranckPranno, NicolaDitrich, TomášPrasad, AnkushDivashuk, MikhailPrasad, Asuri N.Divella, RosaPrasad, ChitraDiVerdi, JosephPrasad, KasavajhalaDivi, Rao L.Prasai, AneshDivoký, VladimírPrassas, IonassisDixit, MuditPrassl, RuthDixit, NaveenPrat, MariaDixon, BrianPratesi, AlessandroDizdaroglu, MiralPratsinis, HarrisDjavaheri-Mergny, MojganPrattichizzo, FrancescoDjerada, ZoubirPrchal, Josef T.Dkhar, Hedwin KitdorlangPreca, Bogdan TiberiusDmitriev, Alexey A.Predescu, DanDo, Duy NgocPrehn, Jochen H. M.Do, Minh TruongPrehna, GerdDo, Sun HeePrêle, Cecilia M.Dobens, Leonard L.Preller, MatthiasDoboszewska, UrszulaPremilovac, DinoDobrev, IvoPrerostova, Mgr. SylvaDobrikova, AneliaPressyanov, DobromirDobrowolska, GrażynaPrestel, MatthiasDobrowolski, PiotrPrestori, FrancescaDobruch, JakubPrestwich, ErinDobrzyńska, MalgorzataPrevette, Lisa E.Docampo, RobertoPrévost, MichelDocea, Anca OanaPreziosa, PaoloDoci, ColleenPreziosi, DanieleDocimo, TeresaPriault, MurielDocoslis, ArisPribyl, JanDoda, Sai ReddyPrice, JeffDodd, Rebecca D.Price, PatriciaDoddapattar, PrakashPriel, AviDodig, DejanPriestman, David A.Doglio, AlainPrieto, ElenaDogra, PranayPrieto, Rafael MariaDoi, KentPrigge, MatthiasDoi, KentaroPrimack, William A.Dokudovskaya, SvetlanaPrinsi, BhaktiDoles, JasonPrintza, NikoletaDoležal, KarelPriola, EmanueleDolezel, JaroslavPrior, Timothy J.Dolga, AmaliaPritchett, JamesDolganov, Pavel VladimirovichPritzker, KennethDolgikh, Elena A.Privette Vinnedge, LisaDolinsky, Vernon W.Procházková, JiřinaDołowy, KrzysztofProchownik, Edward V.Dołowy, MałgorzataProdromos, Chadwick C.Domanskyi, AndriiProescholdt, MartinDomaradzki, PiotrProestos, CharalamposDombrovsky, AvivProestou, Dina A.Dombrowski, YvonneProia, RichardDomenech, MaribellaProietti, SilviaDomenici, Maria RosariaProkop, Jeremy WDomercq, MaríaPrömel, SimoneDomingo, ConchaPronin, Alexander V.Domingo-Calap, PilarProsperi, EnnioDomínguez Díaz, LauraProsperi, JeniferDomínguez Medina, EduardoProszynski, TomekDomínguez Rebolledo, Álvaro EfrénProtasi, FelicianoDomínguez-Álvarez, EnriqueProtasoni, MarinaDomínguez-Vías, GermánProtogerou, Athanase D.Domoki, FerencProtonotarios, AlexandrosDomonkos, IldikóProukakis, ChristosDomozych, David S.Proulx, StevenDon, AnthonyProvot, SylvainDonadel, GiuliaPrudovsky, IgorDonaldson, Logan W.F.Pruess, Birgit M.Donat, CorneliusPrugovečki, BiserkaDonati Zeppa, SabrinaPruneda, Jonathan N.Donati, BenedettaPrystupa, AndrzejDonati, GiacomoPrzekora, AgataDonato, LuigiPrzepiera-Będzak, HannaDonato, Rosario FrancescoPrzybył, JarosławDong, FengPrzybył, Jarosław L.Dong, FengpingPrzybyło, MałgorzataDong, L. (Lemeng)Przygodzki, TomaszDong, MingxinPshezhetsky, AlexeyDong, ShenPsilodimitrakopoulos, SotirisDong, YanPszczółkowska, AgnieszkaDongiovanni, PaolaPuca, AnnibaleDonnay, IsabellePucci, MarziaDonnelly, Callum GeorgePucciarelli, SandraDonnelly, DeirdrePuchades, María JesúsDonnini, SandraPuchalka, RadoslawDonohue, MauraPuchkova, Ludmila V.Donzelli, ElisabettaPuente-Massaguer, EduardDonze-Reiner, TeresaPuerta, ElenaDoody, GinaPuertas, Maria CarmenDoppler, StefaniePuglia, GiuseppeDöppner, Thorsten R.Puglisi, FabioDores, Michael R.Puigdomènech, PereDoretto, PaoloPuiggalí, JordiDorfman, RuslanPUILLANDRE, NicolasDöring, YvonnePuła, BartoszDormán, GyörgyPula, GiordanoDorn, AlexanderPulaski, LukaszDoroftei, BogdanPulavarti, Surya VSRKDorotea, Muck-SelerPulford, KarenDorozhkin, Sergey V.Pulgar, VictorDorożyński, PrzemysławPulicharla, RamaDorsky, RichardPulido, Olga MDos Anjos Pires, MariaPulido, RafaelDos Santos, João Miguel MarquesPulit-Prociak, JolantaDos Santos, Nuno RodriguesPullen, NicholasDos Santos, WagnerPulliero, AlessandraDOSOKY, NOURAPunetha, AnkitaDoss, MohanPungerčar, JožeDosset, MegaliePupilli, FulvioDou, QingpingPuppala, NaveenDou, XiaoguangPuri, SonamDoud, MichaelPurpura, ValeriaDoulamis, IliasPurrucker, Jan ChristophDouros, AntoniosPusch, StefanDoustkhah Heragh, EsmaeilPüschel, GerhardDoveston, RichardPushchina, EvgeniyaDovizio, MelaniaPussinen, PirkkoDovnik, AndrazPusztahelyi, TündeDoxakis, E.Putnik, PredragDoyle, SiamsaPuvvula, Pavan KumarDozois, CharlesPuzer, LucianoDrabczyk, AnnaPycia, KarolinaDracatos, Peter MichaelPyo, Jung-SooDraganova, MilenaPytel, DariuszDragavon, Joseph M.Pyun, Jae-CheolDragomir, MihneaPyzik, MichalDragoni, SilviaQadri, SyedDragos, HorvathQasim, MuhammadDrake, Penelope M.Qasim, Syed Saad BDrakos, EliasQi, JunpengDramsi, ShaynoorQi, Yanfei (Jacob)Dreher, Mark L.Qian, JinDreier, RitaQiao, PengfeiDreschers, StephanQimron, UdiDrexler, BertholdQin, QianDrey, MichaelQin, TingtingDrgan, ViktorQin, Xian-YangDridi, SamiQin, ZhaohaiDrissi-Habti, MonssefQin, ZhenpengDrochioiu, GabiQin, ZhihuiDrosten, MatthiasQiu, BinDrozd, MarcinQiu, YongjianDrozdov, AnatoliyQiu, ZhenDroździk, MarekQu, NingDrulis-Fajdasz, DominikaQu, PengDrummer, HeidiQuadri, MarikaDruzbicki, KacperQuadri, Syed A.Drużyńska, BeataQuadros, EdwardDrzazga, MichałQuagliariello, VincenzoDrzazga, ZofiaQuaglino, DanielaDrzymała-Czyż, SławomiraQuaglio, DeborahDu, QianQuaini, FedericoDu, XiaojunQuang Le, BachDu, ZhifengQuarck, RozennDuan, JialeiQuartu, MarinaDuan, ZhijunQuattrocelli, MattiaDuarte, AnaQuax, PaulDuarte, Filipe V.Quek, CameliaDuarte, JoaoQueralt, EthelDuarte, Maria CristinaQuerleu, DenisDubash, Taronish DorabQuéro, AnthonyDubbelboer, IlseQuesada Pérez, VíctorDubey, RaminQuesnel-Vallières, MathieuDubiel, WolfgangQuigley, RaymondDubin, GrzegorzQuinn, John P.Dubinin, Mikhail V.Quinn, Peter M.J.Dubińska-Magiera, MagdaQuintana, JoséDuBois, RobertQuintana, MercedesDubreucq, BertrandQuintanilla, Luis GDubrez, LaurenceQuintas, AlexandreDubrova, YuriQuintas, Luis Eduardo M.Dubrovina, AlexandraQuinzii, Catarina MariaDuchi, SerenaQuiroga, CeciliaDucreux, SylvieRabanal Ruiz, YoanaDucza, EszterRabiee, HesamoddinDuda, KatarzynaRabuazzo, Agata MariaDudas, JozsefRaccuia, Salvatore AntoninoDudás, ZoltánRachmawati, DessyDuddy, WilliamRachon, DominikDudka, JaroslawRacioppi, MarcoDudok, JeroenRacz, AttilaDuechler, MarkusRacz, IldikoDuennwald, Martin LRadaram, BhaskerDufossé, LaurentRadchenko, Eugene V.Dugar, Rohit P.Radchuk, VolodymyrDugé De Bernonville, ThomasRadcliff, Fiona JaneDuguez, StephanieRademaker, GillesDuhan, VikasRadic, MarcoDuitman, JanWillemRadić, TomislavDulermo, RémiRadichev, IlianDulskas, AudriusRadisavljevic, ZivDuma, LuminitaRádis-Baptista, GandhiDumas, PhilippeRadko, LidiaDumaz, NicolasRadosavljević, TatjanaDumez, SylvainRadošević-Stašić, BiserkaDumitru, GabrielaRadosinska, JanaDumnicka, PaulinaRadu, Beatrice MihaelaDunaeva, Marina V.Radu, FierascuDunbar, Gary L.Radzikowska, UrszulaDungan, CoryRae, ColinDunham, Rex A.Rafael, DianaDunić, Jasenka AntunovićRafi, JunaidDunlap, ChristopherRafiad, IslamDunlop, ElaineRafiq, KaziDunlop, KatharineRagavan, MukundanDunphy, Michael J.Ragazzi, EugenioDünser, Kai AlexanderRageul, JulieDuplaa, CecileRaggi, CarlaDuployez, NicolasRaghunathan, VijayDupraz, SebastianRagino, Yuliya I.Durand, Béatrice C.Ragnini-Wilson, AntonellaDurand, NishaRagno, RinoDuran-Ortiz, SilvanaRagusa, AndreaDuranti, MarcelloRagusa, Maria AntoniettaDuranton, ChristopheRagusa, Michael J.Durazzo, MarilenaRahat, MilkiDurbeej, MadeleineRahbar, AfsarDurgo, KsenijaRahbari, Nuh N.Durham, Benjamin H.Rahikainen, MoonaDurian, GuidoRahimi, FaridDuriancik, DavidRahman, Ashiqur XDuric, VanjaRahman, M. AzizurDurocher, FrancineRahman, Md. AtikurDurrant, Lindy G.Rahman, MilladurDürst, MatthiasRahman, Mohammad MijanurDursun, SerdarRahman, SafikurDuryee, Michael J.Rahmani, FarzinDusi, VeronicaRahnavard, GholamaliDüsterhöft, StefanRai, KrishanDustin, LynnRai, NavneetDutartre, PatrickRai, SumitDuterque-Coquillaud, MartineRai, TatemitsuDutot, MélodyRai, VikrantDutt, Arun K.Rai, VineetaDutta, ArijitRaikar, Sunil S.Dutta, RajeshRaikes, Adam C.Dutta, SanjuctaRaimondo, AnnunziataDutto, MorenoRaimondo, StefaniaDuvigneau, J. CatharinaRaina, DeepakDuvnjak, TomislavRaina, KomalDuwaerts, CarolineRainger, George EdwardDüzgünes, NejatRajagopalan, AnugrahaDvorak, CherylRajakumar, NagalingamDvorakova, Magdalena ChottovaRajalingam, KrishnarajDvorakova-Hortova, KaterinaRajamohan, ArunDworkin, SebastianRajas, FabienneDyakin, Victor V.Rajasekharan, Satish KumarDyda, FredRajendran, Jagathesh ChandraDye, Danielle E.Rajendran, VazhaikkurichiDykhuizen, EmilyRajesh, RamachandranDymarska, MonikaRajkowska, KatarzynaDymińska, LucynaRajnarayanan, RajendramDynarowicz-Latka, PatrycjaRajpurohit, SurendraDyson, Paul J.Rajput, SandeepDzantiev, BorisRaju, HariharanDzhimak, StepanRak, JanuszDzidic, AlenRakic, JelenaDziedzic, ArkadiuszRakonczay, ZoltánDzięgiel, PiotrRakotondrafara, AurelieDziegielewska, BarbaraRakus, DariuszDziendzikowska, KatarzynaRalle, MartinaDzietko, MarkRalli, MassimoDziewięcka, EwaRamachandran, SrinivasanDzimitrowicz, AnnaRamadas, AmuthaDziurka, KingaRamadori, GiulianoDzobo, KevinRamalho Venâncio, Anabela SantoEamens, Andrew L.Ramalho, Maria JoãoEar, JasonRamalho-Santos, JoãoEarl, JulieRamalingam, NagendranEarle, Kenneth A.Ramalingam, NaveenEaswaran, HariharanRamamurthy, NarayanEaton, DouglasRaman, LennartEaton, PeterRamani, KomalEbeed, HebaRamani, KritikaEberhardt, Mirjam JeanetteRamani, VijayEberlein, BernadetteRamaraj, PanduranganEbersviller, SethRamasamy, RanjanEbert, Timothy ARamasubramanian, Anand AnandEbihara, LisaRamaswami, ManiEble, JohannesRamazzotti, MatteoEbrahimi, AzizRambow, FlorianEccles, DavidRamchandani, DivyaEchchannaoui, HakimRamegowda, VenkategowdaEchevarria, MiriamRamesh, AramandlaEchigoya, YusukeRamesh, Sunita A.Ecke, Thorsten HolgerRamezanpour, MahnazEckert, Alexander W.Ramić, SnježanaEckert, AlexanerRamil, CarloEckl, KatjaRamírez Sánchez, ManuelEckschlager, TomasRamírez, ManuelEconomou, AnastassiosRamírez, MartínEda, ShigetoshiRamírez, SaraEdamakanti, Chandrakanth RRamírez-Gonzalez, Ricardo H.Edeas, MarvinRamírez-López, María TeresaEdelman, Irina S.Ramírez-Parra, ElenaEder, Iris E.Ramirez-Peña, EsmeraldaEdin, Nina JeppesenRamos Cabrer, PedroEdiriweera, Meran KeshawaRamos Morales, FranciscoEdman, MariaRamos, CarmenEdmé, SergeRamos, CatarinaEdskes, Herman K.Ramos, SoniaEdwards, Adam B.Ramos-Garcia, PabloEferl, RobertRampazzo, ElenaEftestøl, EinarRampias, TheodorosEgan, TerranceRampon, ChristineEgawa, KiyoshiRamsköld, DanielEgbert, Jeremy R.Ran, SophiaEgea Gutiérrez-Cortines, MarcosRanawade, AyushEgea, GustavoRanc, VáclavEgea, IsabelRancan, FiorenzaEgerszegi, IstvánRand, MatthewEgger, BorisRandall, Stephen K.Eggers, Christian HRandall-Demllo, SarronEgorova, KseniaRandazzo, BasilioEhlen, J. ChristopherRandow, FelixEhler, ElisabethRanganathan, NatarajanEhrich, MarionRanganathan, ParvathiEichmeier, AlesRanganathan, SrivathsanEichstaedt, ChristinaRangappa, Raju BheemanahalliEikmans, MichaelRangasamy, SampathEilertsen, Karl-ErikRangel-Barajas, ClaudiaEiring, AnnaRangnekar, AbhijitEisendle, KlausRanieri, DaniloEisenstat, David D.Ranieri, FedericoEisinger-Mathason, Tzipora S.KarinRanieri, MariannaEiz-Vesper, BrittaRanjan, KishuEkberg, JennyRanjit, SumanEkdahl, Kristina N.Rao, C.V.Ekker, MarcRao, FengEkVitorin, JoseRao, Ponugoti VasanthaEl Kasmi, FaridRao, VidhyaEl Mourabit, HaquimaRapado-González, ÓscarEl Shamieh, SaidRapak, AndrzejElango, JeevithanRapala-Kozik, MariaElansary, Hosam O.Raphael, ItayElaswad, AhmedRapino, CinziaElder, RhoderickRapp, AlexanderEleftheriadis, TheodorosRapposelli, SimonaElemans, J.A.A.W.Rapson, Trevor D.El-Esawi, Mohamed A.Rascio, AgataElewa, Yaser H.A.Rashad, Adel AhmedElezkurtaj, SeferRashdan, NabilElfalleh, WalidRashid, Mohammad HarunElferink, CornelisRašin, Mladen RokoElhai, MurielRaskina, OlgaElia, MaurizioRasmussen, SorenEliaz, IsaacRasoulianboroujeni, MortezaElida Nora, FerriRastelli, GiulioEligini, SoniaRastenytė, DaivaElis, SebastienRastrelli, LucaElisashvili, VladimirRat, PatriceEliseev, RomanRatajewski, MarcinEljaafari, AssiaRateb, MostafaEl-Jawhari, JehanRatera, ImmaElkabets, MosheRathbone, Alex J.Elleder, DanielRather, GulamEllert-Miklaszewska, AleksandraRathinavelu, AppuEllery, Stacey J.Rathmacher, John A.Elliott, EvanRatiu, AndreeaEllis, CameronRato, Luís Pedro FerreiraEllis, Jessica M.Rätsep, MatthewEllison, MichaelRattan, RamandeepEllwood, Simon R.Rattray, ZahraElMallah, Mai K.Rau, IleanaElmarakby, AhmedRaucci, Maria GraziaEl-Mashtoly, Samir F.Raucci, UmbertoElmouki, IliasRauceo, Jason M.Elnitski, LauraRauch, Bernhard H.Elsea, Sarah H.Rauch, FrankEl-Seedi, HeshamRauch, JensElshabrawy, HatemRaudino, AntonioElshafie, Hazem S.Raudsepp, TerjeEl-Sharkawy, IslamRauova, LubicaElson, AriRauvala, HeikkiElviri, LisaRaval, ManmeetElyaman, WassimRavanel, StephaneEmami, PayamRavanidis, StylianosEmous, M.Ravegnini, GloriaEmri, EszterRavera, EnricoEmrich, Fabian C.Ravera, MauroEndepols, HeikeRavi, AnuradhaEndo, MakotoRavi, Rama S.Endo, MitsuharuRavin, Nikolai V.Endo, MizukiRavingerová, TáňaEndre Károly, KristófRavnik-Glavač, MetkaEndres, KristinaRavuri, SudheerEne, RazvanRawat, ManmeetEng, LoRawla, PrashanthEngedal, NikolaiRay, SamriddhaEngel, KathrinRay, SutapaEngel, StanislavRaya, AngelEngeland, Christine E.Rayalam, SrujanaEngelberg, DavidRaychaudhuri, PradipEngelberth, JurgenRayes, JulieEngelhardt, Paul FriedrichRaynaud, CécileEngelmann, IlkaRayport, StephenEngelmann, PeterRažná, KatarínaEngler, AnnaRazvan-Cosmin, PetcaEnguita, Francisco J.Razzaque, SamsadEnjeti, AnoopRazzuoli, ElisabettaEnnequin, GaelRead, David AlanEnomoto, AtsushiReader, Jocelyn C.Enomoto, HirayukiReader, Karen L.Entelis, NinaReagan, John LEnyedi, AgnesReale, MarcellaEom, Gwang HyeonReali, EvaEpand, RichardReam, WaltEppard, ElisabethRebbeck, RobynEpping, Eric A.Rébé, CédricEppler, ElisabethRebelo, SandraEramo, StefanoRebmann, VeraEraña, HasierRebuli, MeghanErastova, ValentinaRebuzzini, PaolaErben, PhilippRecalde, SergioErdélyi, MiklósRecasens, AriadnaErdmann, KatiRechkunova, N. I.Erdődi, FerencReddi, Amit R.Erenpreisa, JekaterinaReddy, Pullagurala Venkata LaxmaEri, S. SrivatsanReddy, SakamuriEric Kast, RichardReddy, Sudhir PuttyEric, TillmanRedell, JohnErickson, Michelle A.Redina, OlgaErickson, TimothyRedmer, TorbenEriksen, Erik F.Redolfi, MichaëlErjavec, Gordana NedicRedondo, MaximinoErland, LaurenRedowicz, Maria JolantaErmakov, ArtemReed, May J.Ermolaeva, SvetlanaReed, William R.Ernsberger, PaulReers, StefanErnst Seemann, StefanReeve, VivienneErnst, MargotReeves, Stephen R.Erogbogbo, FolarinRegal, JeanErsilia, AlexaRegazzo, DanielaErskine, LyndaRegiec, AndrzejErwin, William MarkReglödi, DóraEsakimuthu, ShankarRégnier-Vigouroux, AnneEscalante, RicardoReguła, JulitaEscalona, Hermes E.Regunath, HariharanEscargueil, AlexandreRehfeld, Jens F.Escobedo Monge, Marlene FabiolaRehman, Atteeq UrEscors, DavidRehman, MichaelEscoté, XavierRehrig, ErinEscribese, María M.Reichenbach, AlexEscriu, FernandoReichert, David E.Eskelinen, Eeva-LiisaReid, BrianEskiw, Christopher H.Reid, Christopher A.Eslami, HosseinReif, ShimonEspada, JesúsRein, TheoEspadas Villanueva, IsabelReinach, Peter S.Espen, LucaReinartz, SilkeEspinós, CarmenReiner, AgnesEspinosa-Diez, CristinaReinhard, JacquelineEspinosa-Garcia, ClaudiaReinhardt, ChristophEspinoza-Fonseca, Lennane MichelReinke, HansEspona-Noguera, AlbertReis, HenningEsposito, Emilio XavierReis, SaraEsposito, FrancescoReiser, JakobEsposito, GabriellaReiser, Peter J.Esposito, Maria TeresaReiss, Allison BEsposito, RobertaReiter, OferEsposito, SergioRelat, JoanaEssa, MusthafaRelic, BiserkaEsser, CharlotteReliga, PiotrEsser, Michael J.Rembiałkowska, EwaEssner, JeffRembold, ChristopherEsteban, VanesaRemelli, MaurizioEstévez, FranciscoRemigante, AlessiaEstévez, JorgeRemuzzi, AndreaEstévez, Laura GarcíaRen, JunEstrada-Gutierrez, GuadalupeRen, PingEsworthy, Robert StevenRen, YuanEtich, JuliaRen, ZhenhuaEtienne-Manneville, SandrineRenaud, StephenEtsushi, KurodaRenault, NicolasEttayapuram Ramaprasad, Azhagiya SingamRenčiuk, DanielEudald, CasalsRengaraj, DeivendranEufemi, MargheritaRengasamy, KannanEugène, MEGNASSANRengo, SandroEugenin, EliseoRenshaw, DerekEuler, GerhildRenu, SankarEun, Jung WooRenukuntla, JwalaEuverink, Gert-JanRenzi, SamueleÉva, MikóRenzini, AlessandraEvangelisti, CamillaRenzoni, AdrianaEvangelisti, ClaudioRequena, JesusEvangelisti, EdouardRequicha, JoaoEvangelopoulos, MichaelResch, UlrikeEvani, Shankar JaikishanRescifina, AntonioEvans, Christopher H.Resentini, FrancescaEvans, Dolores Gareth R.Reshkin, StephanEvans, Iwan RobertReshkin, Stephan JoelEveillard, MarionRestivo, Francesco MariaEven-Hernandez, PascaleRetière, ChristelleEvert, MatthiasRettinger, EvaEvinger, MarianRety, StephaneEwans, MatthewRetzer, KatarzynaEwing, AdamReuter, KlausExbrayat, Jean-MarieReuter, SebastianEyers, PatrickRevel-Vilk, ShoshanaEysel-Gosepath, KatrinReverter, DavidEysteinsson, ThorRevuelta, JuliaEzquer, IgnacioRévy, PatrickEzura, YoichiRewitz, Kim F.Ezzeddine, ZeinabReychler, HervéFabbian, FabioReyes Parada, MiguelFabbrini, Maria SerenaReyes-del Valle, JorgeFabbrocini, GabriellaReyes-Garcés, NathalyFabi, João PauloReyes-López, Felipe E.Fabian, ZsoltReyes-Ortega, FelisaFabiani, RobertoReynisson, JóhannesFabiano, EduardoReynoird, NicolasFabio, MunariReynolds, C. PatrickFabiszewska, AgataReynolds, Clare M.Fabrizio, Federico PioReynolds, PaulFacchiano, AngeloŘezáčová, MartinaFacchin, FedericaRezania, ShahabaldinFacchinetti, RobertaRežen, TadejaFacchini, GaetanoRezk, Bashir M.Faccio, GretaReznikov, Leah R.Faciotti, FedericaRhaleb, Nour-EddineFacoetti, AngelicaRhein, MathiasFadeeva, IrinaRibaldone, DavideFafilek, BohumilRibaldone, Davide GiuseppeFagerholm, Susanna C.Ribaudo, GiovanniFaggioli, FrancescoRibeiro, ArturFaggioni, MichelaRibeiro, Artur Jorge Araújo MagalhãesFagioli, FrancaRibeiro, César Augusto JoãoFagoonee, SharmilaRibeiro, IsabelFahlbusch, Fabian B.Ribeiro, Viviana P.Fahnestock, MargaretRibeiro-Barros, Ana I.Failla, Cristina M.Ribera, JordiFailla, OsvaldoRibero, SimoneFaille, ChristineRicagno, StefanoFairless, RichardRicardo, SaraFairweather, StephenRiccardi, ClaudiaFais, AntonellaRiccardo, Zenezini ChiozziFaivre-Sarrailh, CatherineRicci, ClaudiaFajardo, Val AndrewRicci, FedericoFakhouri, Walid D.Ricci, GiuliaFakhrullin, Rawil F.Ricciardelli, CarmelaFalcieri, ElisabettaRicciardi, LoredanaFalcone, ItaliaRicciardi, Maria RosariaFaleiro, Maria LeonorRiccio, AntonellaFalguières, ThomasRiccio, GennaroFali, TinhinaneRiccio, PaoloFalistocco, EgiziaRicci-Vitiani, LuciaFalkinham III, Joseph O.Richard, BodnarFallini, ClaudiaRichard, MelissaFalls, ZackaryRichard, SabineFalsini, BenedettoRichard, SteetFambrini, MassimilianoRichards, Dylan J.Famulski, JakubRichards, Joanne S.Fan, Guo-ChangRichardson, LaurenFan, Hueng-ChuenRichardson, Sandra R.Fan, JianglinRichardson, Simon C. W.Fan, LushengRiches-Suman, KirstenFan, WenchunRichter, KatharinaFan, ZhengRichter, KatrinFan, ZhichaoRichter, Manuel JonasFandrey, JoachimRichter, PeterFane, MitchellRichter, ShacharFanelli, ValentinaRichter, SusanFang, ChengRichter, WitoFang, Evandro F.Richtera, LukasFang, JenniferRichter-Dennerlein, RicardaFang, JiyuRiddiford, Lynn M.Fang, JunnanRideout, Elizabeth J.Fang, QingmingRidola, LorenzoFang, RunchenRieder, Leila E.Fang, SungSoonRiedl, RainerFang, XingRiese, Matthew J.Fang, YiRietdorf, KatjaFanning, SarannaRiffo-Vasquez, YaniraFanourakis, GalinosRigalli, Juan PabloFantacuzzi, MarialuigiaRiganti, ChiaraFantini, MassimoRigas, StamatisFantini, SebastianRighetti, LauraFaouzi, MalikaRighi, AlbertoFarabegoli, FulviaRighi, MarcoFaraji, FarhoudRigo, RiccardoFaralli, Jennifer A.Rigual, VictoriaFaraoni, IsabellaRihan, HailFarcasanu, Ileana C.Rijavec, MatijaFarè, SilviaRikkerink, ErikFareed, AmberRilcheva, Violeta S.Fareed, JawedRim, John HoonFaria De Oliveira, VirusMichelliRim, Kyung TaekFaridi, PouyaRimoldi, IsabellaFariña, José B.Rimondi, ErikaFarina, NicholasRimondini, LiaFarinha, CarlosRimpelova, SilvieFaris, RobertRimpelová, SilvieFarjon, JonathanRinaldi, FabioFarkas, Attila E.Rincon, Melvin Y.Farkas, LaszloRingli, ChristophFarkaš, VladimírRinschen, Markus M.Faron-Górecka, AgataRiobo, NataliaFarrar, Michael A.Rios, FranciscoFasinu, PiusRios-Covian, DavidFasler, ElizavetaRíos-Santos, Jose VicenteFasolato, CristinaRipperger, Jürgen A.Fass, DeborahRis, LaurenceFast, Loren D.Risk, Janet M.Fatkhudinov, TimurRiska, PaulFaussone-Pellegrini, Maria SimonettaRisner, MichaelFaustino, RandolphRitchie, HelenaFaux, DavidRitsma, LailaFavaloro, EmmanuelRitter, AndreasFavara, GiulianaRiuzzi, FrancescaFavery, BrunoRiva, AntonellaFavi, EvaldoRiva, FedericaFavia, MariaRivera, ErnestoFavier, Anne-LaureRivera, Hugo VaraFavre, ClaudioRivera, Víctor M.Fay, FabienneRivera-Monroy, Zuly JennyFazeli, AlirezaRivero, FranciscoFazi, FrancescoRivinius, RasmusFazio, FrancescoRižner, Tea LanišnikFeás, XesúsRizos, DimitriosFebbraio, FerdinandoRizvanov, AlbertFede, CaterinaRizvi, Syed A. A.Fedele, FrancescoRizzi, ChiaraFedele, MonicaRizzi, LauraFederico, AntonioRizzi, ManuelaFedorova, Olga S.Rizzi, MenicoFedorova, Olga V.Rizzo, AlessandroFedotova, Julia O.Rizzo, FabianaFeghali-Bostwick, CarolRizzo, PaolaFeher, GergelyRo, Hyeon-SuFeher, TamasRo, HyunjuFehon, RichardRo, Seung-HyunFehr, AnthonyRoana, JaniraFehse, BorisRoato, IlariaFeinn, Richard S.Robakowski, PiotrFelder-Schmittbuhl, Marie-PauleRobaszkiewicz, AgnieszkaFeldman, SteveRobben, JohanFeldo, MarcinRobert, PhilippeFelicetti, TommasoRoberto, SilvanaFeliciano, JoanaRoberto, VâniaFélix, CarinaRoberts, DavidFélix, LuisRoberts, TaraFelley-Bosco, EmanuelaRobertson, Cayleih E.Felmerer, GuntherRobin, Arif Hasan KhanFelten, MatthiasRobin, Jérôme D.Fendt, MarkusRobinson, Anne SkajaFeng, ChaohuiRobinson, DouglasFeng, Hua-JunRobinson, KarlFeng, HuapengRobinson, SeriFeng, LijuanRobles, VanesaFeng, YongjunRobles-Marhuenda, A.Fenini, GabrieleRoby, KatherineFenton, TimRoca, JoaquimFeo, FrancescoRocca, Carlo DellaFeofanov, Alexey V.Rocca, RobertaFercher, ChristianRoccaro, AldoFeregrino-Pérez, Ana A.Rocha, JavierFerens-Sieczkowska, MirosławaRochais, ChristopheFerji, KhalidRocha-Martin, JavierFernandes Fagundes, NathaliaRocha-Rodrigues, SílviaFernandes, AnaRoche, BenjaminFernandes, HugoRoche, Marc De LaFernandes, Maria HelenaRoche, PhilippeFernandes, Maria José Da SilvaRocher, AsuncionFernandes, MónicaRochfort, KeithFernandes, TiagoRoda, AldoFernandez Fairén, MarianoRodamilans, BernardoFernandez, Agustin F.Rodat-Despoix, LiseFernandez, DianaRodella, Luigi F.Fernández, IgnacioRodemer, WilliamFernández, José A.Roderburg, ChristophFernández, LucíaRodet, FranckFernández, ManuelRodgers, JeanFernandez, MercedesRodilla, VerónicaFernandez, PaulaRodin, Andrei S.Fernandez, Sandra V.Rodolfo, MonicaFernández, Vanesa MartínRodrigo, GuillermoFernández-Álvarez, AlfonsoRodrigo, IsmaelFernandez-Botran, RafaelRodrigo-Muñoz, José MFernandez-Funez, PedroRodriguero, MarcelaFernández-García, MartaRodrigues, António S.Fernández-Gutiérrez, MarRodrigues, Célia F.Fernández-Mateos, M. PilarRodrigues, FranciscaFernandez-Pozo, NoeRodrigues, JoanaFernández-Rodríguez, JavierRodrigues, Márcia T.Fernandez-Rojo, Manuel A.Rodrigues, OlivierFernández-Ruiz, JavierRodrigues, Pedro MiguelFernández-Sánchez, LauraRodrigues, TeresaFernández-Tomé, SamuelRodríguez González, ÁlvaroFernández-Velasco, MaríaRodriguez Lozano, Francisco JavierFernandez-Vizarra, ErikaRodriguez Sosa, Jose RFernando, BinoshaRodriguez Valle, ManuelFernando, M. RohanRodríguez Villanueva, JavierFerramosca, AlessandraRodriguez, AnalizFerrando, RiccardoRodriguez, CristianFerrante, AntonellaRodríguez, IsabelFerrante, AntonioRodríguez, Matías MorínFerrante, TonyRodriguez, PatriciaFerrara, FelicettoRodríguez, Ricardo ReyesFerrara, NicolaRodríguez, Víctor ManuelFerrara, Nicole C.Rodriguez-Cueto, CarmenFerrari, FedericoRodriguez-Gil, JorgeFerrari, PaolaRodríguez-Jerez, José JuanFerrario-Méry, SylvieRodríguez-López, LorenaFerraroni, MartaRodriguez-Lorenzo, LauraFerrarotti, FrancescoRodríguez-Lozano, Francisco JavierFerraz, Marcia De Almeida Monteiro MeloRodríguez-Pallares, JannetteFerraz, RicardoRodríguez-Perálvarez, ManuelFerreira, HelenaRodríguez-Sanz, DavidFerreira, JorgeRodriguez-Sinovas, AntonioFerreira, Leonardo M.R.Rodriguez-Vargas, Jose M.Ferreira, Manuel M.Rodriguez-Vita, JuanFerreira, NelsonRodríguez-Yoldi, María JesúsFerreira, Paula M.Rodriquez, ManuelaFerreira, SamiraRodziewicz-Motowidło, SylwiaFerreira-Cerca, SebastienRoe, Richard MichaelFerreira-Dias, Graça M.Roedel, FranzFerreon, Allan Chris M.Roelen, BernardFerreon, JosephineRoex, GilsFerrer, GerardoRogalska, JustynaFerreras, José MiguelRogatzki, MatthewFerreri, CarlaRoger, LaurélineFerrer-Miralles, NeusRogers, HilaryFerrer-Montiel, AntonioRogina, AnamarijaFerrer-Perez, CarmenRogobete, Alexandru FlorinFerretta, AnnaRogozea, LilianaFerri, FlaminiaRogozhin, EugeneFerrigo, DavideRoh, SanghoFerriz-Martínez, Roberto AugustoRohacs, TiborFerro, AlbertRohan, Fernando M.Ferro, DomenicoRohde, John R.Ferro, MatteoRohila, JaiFerro, RiccardoRohila, Jai S.Ferroni, LorenzoRoizenblatt, MarinaFerruz-Capapey, NoeliaRojano-Delgado, Antonia MariaFessner, Wolf-DieterRojo Marcos, Francisco JavierFetissov, Sergueï O.Rojo, Maria AngelesFettke, JörgRokavec, MatjazFiandra, LuisaRokosh, Donald G.Fiani, Maria LuisaRokstad, Anne MariFiaschi, TaniaRolbiecki, LeszekFields, Peter D.Roldo, MartaFierabracci, AlessandraRollenhagen, ChristianeFiering, Steve N.Roller, Richard J.Fiering, Steven N.Rom, SlavaFieseler, LarsRoman, ShaunFigaj, DonataRomanelli, AlessandraFigiel, AdamRomanello, VaninaFigueiredo, AndreiaRomanenko, Svetlana A.Figueiredo, ConstançaRomano, AlessandraFijak, MonikaRomano, Giovanni LucaFik-Jaskółka, Marta A.Romano, LorenzoFilaci, GilbertoRomano, M CarmenFiladi, RiccardoRomano, MarcoFilarowski, AleksanderRomano, NiclaFilek, MariaRomanov, Georgy A.Filella, XavierRomanov, Roman A.Filgueira, CarlyRomański, JarosławFilho, AlvesRomão, LuísaFiliou, MichaelaRoma-Rodrigues, CatarinaFilipcik, PeterRomero Morales, CarlosFilipek, AgnieszkaRomero, Fernando M.Filipek, AnnaRomero, IreneFilipič, BratkoRomero, JulianFilipkowski, RobertRomero, Marta R.Filipova, IneseRomero-Lorca, AliciaFilipovic, MilosRomero-Reyes, MarcelaFilippelli, AmeliaRonca, RobertoFilippou, Panagiota S.Roncada, PaolaFilippov, AndreiRoncevic, TomislavFilippov, Mikhail A.Ronchetti, SimonaFilip-Psurska, BeataRoncucci, LucaFiliz, ErtugrulRondanino, ChristineFillmore, HelenRonellenfitsch, Michael W.Filová, EvaRong, Chan KuanFimognari, CarmelaRongvaux, AnthonyFinelli, CarmineRoni, MonzurulFineschi, VittorioRoninson, IgorFinet, CédricRonnberg, ElinFinetti-Sialer, Mariella MatildeRonquist, GunnarFingerhut, RalphRonzan, MarilenaFini, Mehdi ARonzitti, GiuseppeFink, DieterRoquis, DavidFink, GeorgeRosa, Arianna CarolinaFinkelstein, DavidRosa, Bruce A.Finlay-Schultz, JessicaRosa, IreneFinlayson, Courtney AnneRosado, CarlosFintini, DaniloRosado, JuanFiocchetti, MarcoRosa-Garrido, ManuelFionda, CinziaRosales, RubenFioramonti, XavierRosas-Hernandez, HectorFiore, MicheleRosati, JessicaFiorelli, SusannaRosato, AntonioFiorentini, GiammariaRoscoe, ThomasFiorentino, GabriellaRose, Christopher F.Fiorenza, RobertoRoseanu Constantinescu, AncaFiori, EnricoRoseanu, AncaFiori, MarianaRose-John, StefanFiorillo, LucaRoselli, MariannaFiorio Pla, AlessandraRosen, Michael J.Firdessa, RebumaRosendale, Andrew J.Firestein, BonnieRosenfeld, MarkFisar, ZdenekRosengren, JohanFischer, Dagmar-ChristianeRosenkranz, Jeremy AmielFischer, JörgRosenthal, CynthiaFischer, LukášRosenthal, RitaFischer-Fodor, EvaRosenzweig, Steven A.Fischer-Smith, TracyRoshan, AmitFiscon, GiuliaRoshanghias, AliFisher, Jonathan S.Rosic, GvozdenFisher, ScottRosicka-Kaczmarek, JustynaFisher-Wellman, KelseyRösl, FrankFishman, PninaRoss, IanFisslthaler, BeateRoss, MatthewFiszer, AgnieszkaRos-Santaella, José LuisFitches, ElaineRossbach, OliverFitton, HelenRossetti, CarloFitz, Nicholas F.Rossi, AndreaFiume, GiuseppeRossi, BarbaraFladung, MatthiasRossi, FilomenaFlak, Jonathan NRossi, FrancescaFlannery, JohnRossi, GiulioFleites, Laura A.Rossi, MichelaFletcher, AnneRossi, MiriamFletcher, Madeleine M.Rossi, Noreen F.Fletcher, Nicola F.Rossi, PaoloFletcher, SusanRossi, PellegrinoFlick, MatthewRossignol, JulienFliefel, Riham M.Rosso, ChiaraFliegel, LarryRosspeintner, ArnulfFlieger, JolantaRossy, JérémieFliegert, RalfRoszak, PawelFlinn, BarryRoszczenko-Jasinska, PaulaFlint, MikeRoterman, IrenaFlint, SteveRoth, MichaelFlorán, BenjamínRothbauer, MarioFlorczyk, StephanieRothenberg, RichardFlorea, AdrianRothhammer, VeitFlorea, Ana-MariaRotili, DanteFlorescu, MonicaRotoli, Bianca MariaFlores-Félix, J.D.Rotolo, CaterinaFlores-Guerrero, Jose L.Rotondo, John CahrlesFlores-López, María L.Rouault-Pierre, KevinFloria, MarianaRoudier, EmilieFlorian, Maria CarolinaRoue, GaelFloris, MatteoRouge, PierreFlors, VictorRougeot, JulienFlottmann, FabianRouhiainen, AriFluri, FelixRouillé, YvesFodor, FerencRouleau, EtienneFoecking, MelanieRouleau, MatthieuFogolari, FedericoRousar, TomasFojta, MiroslavRousselle, PatriciaFojtová, MiloslavaRousset, FrancisFoka, PelagiaRouthu, Nanda KishoreFokkema, M. Rebecca HeinerRoutray, PratyushFol, MarekRoux, Stanley J.Foley, Ann C.Rovatsos, MichailFoley, ChrisRovere, MauroFolgosa, FilipeRoversi, PietroFolini, MarcoRovida, ElisabettaFologea, DanielRovina, DavideFonkem, EkokobeRowe, Glenn C.Fonseca, Bruno Miguel Reis DaRowley, Paul AndrewFontana, Barbara D.Roy, AnuradhaFontana, FedericoRoy, DipankarFontana, PaoloRoy, IpsitaFontanesi, FlaviaRoy, ParthaFontenla, Jose AngelRoy, Saktimayee MitraFoong, JaimeRoy, SoumenForbes, LouisaRoy, Swapan KumarForbes-Hernández, Tamara Y.Roy, UrmiForbester, JessicaRoychowdhury, RajibFord, NicoleRoyo, FelixForero-Castro, MaribelRóżalska, BarbaraForet, FrantišekRóżalska, SylwiaForini, Francesca S.Rozalski, MarcinForlino, AntonellaRozhon, WilfriedForma, EwaRozov, Serge M.Formanowicz, DorotaRóżycki, BartoszFormela, KrzysztofRozza, ArianeFormoso-Rafferty, NoraRuan, KeFornai, FrancescoRuaro, BarbaraFornai, MatteoRubasinghege, GayanFornstedt, TorgnyRubattu, SperanzaForster, JamesonRubin, Robert L.Forstova, JitkaRubina, KseniyaForsyth, Christopher B.Rubinowska, KatarzynaForsyth, NicholasRubio, MyriamFort, Patrice E.Rubio-Aliaga, IsabelFort, PhilippeRubio-Cabetas, M.J.Forte, Iris MariaRubis, BlazejForte, NicolaRubis, PawelForte, Stefano ForteRubtsov, NikolayFortis, Sotirios P.Rucci, NadiaFortunato, OrazioRuch, RandallFoster, JohnRucinski, MarcinFoster, TimRucker, Robert B.Foster, Toshana LauriaRuden, DouglasFoster, Warren G.Rudnicka, WiesławaFotouhi, OmidRudnóy, SzabolcsFouladkhah, AliyarRudolf, EmilFoulke-Abel, Jennifer D.Rudolph, UweFoulquier, SébastienRudra, ArnabFountain, Samuel J.Rudroff, ThorstenFountoulaki, EleniRudzińska, MagdalenaFournier, AntoineRueda, BoFournier, Marcia V.Ruegg, JoelleFournier, Neil M.Ruegg, UrsFowkes, RobRuest, L. BrunoFowler, MarcieRuffinatti, Federico AFox, JulieRuffinatti, Federico AlessandroFox, Keith R.Ruffner, Melanie A.Foyer, Christine HRuffolo, GabrieleFracanzani, AnnaRufini, AlessandroFrachisse, Jean-MarieRuggieri, VitalbaFradet, VincentRugolo, MichelaFradin, ChantalRuhanya, VurayaiFradin, DelphineRühl, MartinFraga-Corral, MariaRuivo, RaquelFragai, MarcoRuiz De Mier, M. Victoria PendónFragliasso, ValentinaRuiz Laza, RocíoFraguas-Sánchez, Ana IsabelRuiz Ojeda, Francisco JavierFraichard, StéphaneRuiz Téllez, TrinidadFraineau, SylvainRuiz, FacundoFraizer, GailRuiz, MarioFrampton, GarrettRuiz, MatthieuFrancesca, LunardiRuiz-Rodríguez, Juan C.Franceschelli, SaraRuiz-Ruano, Francisco J.Francesco, ScavelloRuiz-Valdivieso, VictorFrancesco, TrapassoRuiz-Velasco, VictorFrancis, Ashwanth ChristopherRummel, AndreasFrancis, Brian R.Rumyantseva, NatalyaFrancis, HaneRund, DeborahFrancis-Boyle, OliviaRuppert, ElisabethFrancisco, VeraRurek, MichalFranco, IreneRusak, MalgorzataFranco, LuisRusanova, IrynaFranco, Marina SantiagoRusciano, DarioFranco, OmarRusciano, Maria RosariaFranco, RodrigoRusinek, DagmaraFranco-Barraza, JanuszRusmini, PaolaFrancs-Small, Catherine Colas DesRusnati, MarcoFrandes, MirelaRuss, BrendanFrank, LuizaRussell, RodFrank, SašaRussell, ScottFranke, JakobRussi, SabinoFrank-Kamenetskaya, Olga V.Russo, AlessandroFranko, AndrasRusso, AnnapinaFranks, Jennifer M.Russo, ChiaraFranzè, AnnamariaRusso, DanielaFranzese, MonicaRusso, DomenicoFranzini, AncaRusso, EthanFranzyk, HenrikRusso, Francesco PaoloFrasca, AngelisaRusso, GiuliaFrasca, LoredanaRusso, IsabellaFraser, MichaelRusso, MariaFrassanito, Maria AntoniaRusso, MassimoFrasson, IlariaRusso, Matteo A.Frati, AlessandroRusso, NinoFratzl-Zelman, NadjaRussu, WadeFrazer, AlanRusu, Laura CristinaFrazer, J. KimbleRusu, Mugurel ConstantinFrech, MoritzRusu, TeodorFrederick Charles, NuciforaRuszkowska-Ciastek, BarbaraFredholm, MereteRuthstein, SharonFredua-Agyeman, RudolphRutigliano, GraziaFreedman, BenjaminRutkowski, Joseph M.Freedman, JonathanRutters, FemkeFreiberger, Sandra NicoleRutzler, MichaelFreichel, MarcRuvo, MenottiFreitag, JulienRuysschaert, Jean-MarieFreitas De Freitas, LucasRůzǐčka, KamilFreitas, RenataRuzza, PaoloFreitas, Vanessa MoraisRuzzene, MariaFrenkel, AmitRyabchikova, Elena I.Frenkiel, SaulRyadnov, MaxFreude, KristineRybak, Leonard P.Freudlsperger, ChristianRybka, Jakub DaliborFreytag, SaskiaRybka, KrystynaFrezza, ClaudioRybtsov, StanislavFrezzato, FedericaRychahou, PiotrFrias Casas, MarioRychkov, DenisFrick, ManfredRychkov, GrigoriFricker, Lloyd D.Rychlik, BlazejFried, MiklosRymowicz, WaldemarFrieden, MaudRyota, TamuraFrieri, GiuseppeRyszawy, DamianFries, FabianRyszkowska, JoannaFrietze, SethRytelewski, MateuszFrigeri, AntonioRyu, ChangseonFriis-Hansen, LennartRyu, JiyoonFrisch, Steven M.Ryu, Jung SuFrischauf, IreneRyu, Kwon-YulFrisso, GiuliaRyu, SangjinFritsche, RaphaelaRzemieniec, JoannaFrittitta, LuciaRzepecka-Stojko, AnnaFroehlich, LeopoldRzepkowska, MalgorzataFroeyen, MatheusRzewuska, MagdalenaFroeyen, MathyRzońca, Sylwia OlimpiaFrohme, MarcusRzymowska, JolantaFrolíková, MichaelaRzymski, PiotrFrosini, MariaSá, RosáliaFrossi, BarbaraSaad, Nizar Y.Fruchon, SéverineSaakes, MichelFrugé, AndrewSaavedra, Juan M.Fruhbeck, GemaSaba, Nakhle S.Fry, Stephen C.Sabater, BartolomeFu, JianmingSabatino, DavidFu, LianwuSabatino, LeoFu, XingSabhachandani, PoojaFu, XueweiSabina, SaverioFu, YingSabnis, HimaleeFu, ZhengSabogal-Guáqueta, Angélica MaríaFu, ZhongjieSabol, MajaFuchs, DietmarŠabovič, MišoFuchs, OtaSaccà, Sergio ClaudioFucile, SergioSaccenti, EdoardoFuellen, GeorgSacco, PasqualeFuentes, EduardoSacco, RodolfoFuggi, AmodioSaccone, SalvatoreFujihara, YoshitakaSaccone, ValentinaFujihara, YukoSachar, MadhavFujii, HideakiSacher, EdwardFujii, HidekiSachio, TsuchidaFujii, HiroakiSachithanandham, JaiprasathFujii, KentaroSachkova, MariaFujii, MasayukiSacitharan, Pradeep KumarFujii, ShoSack, UlrichFujii, YoshiharuSaclier, MarielleFujii, YukiSaczewski, JarosławFujimori, KoSaczonek, Agnieszka OwczarczykFujimoto, AyatakaSadaghianloo, NirvanaFujinaga, KohSadasivam, MohanrajFujisawa, YasuhiroSaddala, Madhu SudhanaFujita, KaoriSader, RobertFujita, KazutoshiSadhukhan, AyanFujita, KojiSadiku, PranveraFujita, MasakiSadler, AnthonyFujita, MikakoSadovsky, YoelFujita, RyosukeSadri-Ardekani, HoomanFujita, YukiSadykov, Marat R.Fujiwara, KeigiSaeki, KumikoFujiwara, SatoruSáez, Guillermo T.Fujiwara, TomoyaSafa, Ahmad R.Fujiwara, ToshinobuSafo, Martin K.Fujiwara, YasuhiroSafronova, TatianaFukada, So-ichiroSagheddu, ClaudiaFukagawa, TatsuoSagner, AndreasFukai, TohruSagnou, MarinaFukuchi, SatoshiSah, NirnathFukuda, AisakuSaha, DipongkorFukuda, MitsuruSaha, NirmalyaFukuda, NoboruSaha, RanajayFukuda, TakeshiSahab, SareenaFukudome, AkihitoSaharan, LalitaFukuhara, AtsunoriSaheki, TakeyoriFukuhara, YasuyukiSahiner, NurettinFukunaga, KohjiSahni, Sanjeev K.Fukunaga, NaotoSahoo, PabitraFukunaga, RyuyaSahu, DebashishFukunaga, TsukasaSahu, Ravi PFukuoka, HidenoriSaielli, GiacomoFukushima, AtsushiSaif, SadiaFukushima, HiroshiSaika-Voivod, IvanFukushima, KentaroSaini, Ramesh KumarFukushima, TsuyoshiSaini, ShikhaFukuta, ShiroSaint-Dizier, MarieFukuyama, TomokiSaint-Pol, JulienFulci, ValerioSainz, Rosa MFulciniti, FrancoSaito, IzumuFulford, TomSaito, KanFulle, StefaniaSaito, MarikoFuller, BryanSaito, RyokoFuller, MariaSaito, YoshiroFumagalli, FrancescaSaito, YukihiroFumagalli, LauraSaitoh, AkiyoshiFumarola, ClaudiaSaitoh, IsseiFunakoshi, KengoSaitoh, MasaoFunato, NorikoSaiz-Fernández, IñigoFunato, YosukeSaiz-Sanchez, DanielFunel, NiccolaSajesh, Babu V.Funk, RichardSajeva, MaurizioFurdui, BiancaSak, Jarosław J.Füredi, AndrásSakabe, MasahideFurfaro, Anna LisaSakač, NikolaFurtula, BorisSakai, AtsushiFurue, MasutakaSakai, HidekiFuruta, KazuyukiSakai, HiromiFuruta, SaoriSakai, HiromichiFushida, SachioSakai, HiroyasuFüssel, SusanneSakakibara, IoriFuster, Jose JavierSakakibara, KeikoFutakuchi, MitsuruSakamoto, AkioFutoma-Kołoch, BożenaSakamoto, KenjiFutrega, KathrynSakamuri, Siva Sankara Vara PrasadFutter, ClareSakamuro, DaitokuFuu-Jen, TsaiSakane, FumioGaag, Kristiaan J. Van DerSakata, JunkiGabandé-Rodríguez, EnriqueSakata, NaoakiGabbia, DanielaSakellari, DimitraGabbiani, GiulioSaki, MortezaGabdulkhakov, AzatSaki, NajmaldinGaber, TimoSakina, Mhaouty-KodjaGabernet, GiselaSako, KaoriGabler, ChristophSakr, SoulaimanGaborit, BénédicteSaksena, NitinGabrielli, BrianSaksena, SeemaGabrielson, Nathan P.Sakthivel, KogularasuGaceb, AbderahimSakurai, HidetoshiGacesa, RankoSakurai, KenichiGachomo, EmmaSakurai, YuGachon, FredericSala, GessicaGackowski, DanielSala, Lucia LaGaczynska, MariaSaladi, Srinivas VinodGad, AhmedSalagierski, MaciejGadad, ShrikanthSaláková, MartinaGadaleta, AgataSalamanca, EisnerGadeau, Alain-PierreSalamon, SylwiaGadella, Theodorus WJSalánki, KatalinGaetani, LorenzoSalas, Joaquín J.Gafencu, AncaSalas-Huetos, AlbertGaffet, EricSalazar, IvanGaffney, ChristopherSalazar, Juan J.Gaforio, JoséSaldarelli, PasqualeGafurov, MaratSaleem, AyeshaGagat, MaciejSaleem, FoziaGaglia, Marta MariaSaleh, Fatima A.Gagliano, CaterinaSaleh, MonaGagliano, NicolettaSalehi, S AlbertGagliardi, Paolo ArmandoSalehi, SarahGaglio, DanielaSalehzadeh-Yazdi, AliGagniuc, PaulSalem, AbdelhakimGago-Fuentes, RaquelSalem, VictoriaGai, ZhiboSalemi, MicheleGaid, MariamSaler, MarcoGaida, MatthiasSalerno, Alessandro GonzalezGaiddon, ChristianSales, EsterGaipl, Udo S.Sales-Gil, RaquelGaitonde, VishwanathSalgar, ShashikumarGajavelli, ShyamSalifoglou, AthanasiosGajdosik, Martina SrajerSalim, VonnyGajdzis, PawelSalimi, ZahraGajger, Ivana TlakSalinska, ElzbietaGajos-Michniewicz, AnnaSallam, AhmadGalan, JacobSalmeri, MarioGalan, MariaSalmhofer, HermannGalasso, GiovanniSalmina, AllaGalateanu, BiancaSalmon, MorganGalatou, EleftheriaSalomaa, SiskoGalazios, GeorgiosSalomon, JohannaGalderisi, UmbertoSalonurmi, TuireGaldiero, MarilenaSalpini, RominaGaleffi, PatriziaSalsi, ValentinaGales, LuísSalta, EvgeniaGali, HimabinduSaltarella, IlariaGaliba, GaborSalton, FrancescoGalina, GenchevaSalucci, SaraGalindo, AntonioSaluk-Bijak, JoannaGalindo, Fernando ShintateSalunke, SmitaGalindo-Villegas, JorgeSalvador F, AliñoGalkin, Alexander S.Salvadori, NicolaGalkina, Svetlana I.Salvarani, Nicolo’Gall, JosephSalvati, EricaGáll, TamásSalvato, FernandaGáll, ZsoltSalvatore, FrancescoGallagher, Jennifer E. G.Salvatore, LisaGallagher, RenataSalvatore, Maria MichelaGallagher, TravisSalvi, MauroGallavardin, ThibaultSalvi, SilviaGallego, Juan CarlosSalzer, IsabellaGallego, Óscar S.Sam, RaminGalleni, MorenoSamaja, MicheleGallet, Paul FrançoisSamal, SangramGalli, AlvaroSamal, SnehaGalliciotti, GiovannaSamanta, Pralok KumarGallo Cordova, AlvaroSambandam, VaishnaviGallo, DanielaŠamec, DunjaGallo, Maria PiaSamhan-Arias, AlejandroGallo, SalvatoreSamiec, MarcinGallo, SimonaSamigullin, Tahir H.Gallorini, MarialuciaSamir, ParimalGalpert, DeborahSammar, MareiGaluska, Sebastian P.Sammarco, GiuseppeGaluska, Sebastian PeterSamochowiec, JerzyGalvagni, FedericoSampaolo, SimoneGalvão, Adelino M.Sampath, ChethanGalvao, IzabelaSampath, KarunaGama, AdelinaSamson, Abraham O.Gama, SofiaSamsonov, SergeyGama, VivianSamsudin, Asep MuhamadGamal El-Din, Tamer M.Samuel, Samson MathewsGamaleev, VladislavSamuels, A. LaceyGaman, AmeliaSamuels, Jon DeanGaman, Amelia MariaSamulski, Richard JudeGaman, Mihnea-AlexandruSan Bautista, AlbertoGambardella, AlessandroSanabria, HugoGambardella, JessicaSanak, MarekGambari, LauraSanche, LeonGamberi, TaniaSanchez Galan, JavierGamboa-deBuen, AliciaSanchez Muñoz, RaulGamella Carballo, MariaSanchez Vargas, Luis AlbertoGamian, AndrzejSanchez, AnaGamper, ArminSanchez, GoarGan, RenyouSánchez, Juan AntonioGan, SamuelSánchez, RobersyGanaie, SafderSánchez, TeresaGanapathy, VadivelSánchez-De-Diego, CristinaGanassi, SoniaSanchez-Infantes, DavidGanau, MarioSancho, JaimeGanazzoli, FabioSancho-Vaello, EneaGandellini, PaoloSandbichler, AdolfGandhirajan, Rajesh KumarSandeep, DaveGandía, MónicaSanders, YanGanesan, A.Sandjo, Louis PergaudGanesan, MuraliSandlers, YanaGanesh Rai, BantukalluSandomenico, AnnamariaGangadaran, PrakashSandovici, IonelGanini, CarloSandrock, IngaGanji, RakeshSands, Jeff MGannett, Peter M.Sandsmark, DanielleGannon, MaureenSan-Félix, Ana RosaGanopoulous, IoannisSanfilippo, CristinaGanser, ChristianSangadala, SreedharaGantz, StephanieSangiao-Alvarellos, SusanaGanz, JuliaSangineto, MorisGao, GarySangiovanni, EnricoGao, JieSangireddy, Sasikiran ReddyGao, ShiqiangSanguedolce, FrancescaGao, SongSanguigni, ValerioGao, XiaolongSanij, ElaineGaponenko, VadimSanjairaj, VijayavenkataramanGaraigorta, UrtziSanjeev, ChawlaGaranto, AlejandroSanjeewa, Kalu Kapuge AsankaGaranto, AlexSankararaman, SenthilkumarGarau, AlessandraSankarasubramanian, VishwanathGaravelli, LiviaSanmartí, RaimónGarbar, ChristianSanna, FabrizioGarcia Alonso, Maria AlejandraSansberro, Pedro AGarcia De Frutos, PabloSansone, AnnaGarcia Perez, SamuelSantabarbara, StefanoGarcía Souto, DanielSantacroce, LuigiGarcia, Andres SanzSantamaria, DavidGarcia, AntonioSantamaría, RafaelGarcía, ConcepciónSantamaria, RitaGarcia, CyrielleSantamaría-Martinez, AlbertGarcía, GabrielaSantambrogio, PaoloGarcia, Jose MartinSantangelo, CarmelaGarcía, María-LuzSantarelli, AndreaGarcia, MichelleSantarriaga, StephanieGarcía, MontserratSanthakumar, Abishek BommannanGarcia, Natalia MoraSanthanam, AbiramiGarcia, SamuelSanthoshkumar, PutturGarcia, VictorSanti, Chiara DeGarcía-Álvarez, YolandaSanti, ClaudioGarcía-Aranda, MarilinaSanti, LucaGarcía-Barrado, María JoséSanti, MelissaGarcia-Borron, Jose CarlosSanti, SimonettaGarcia-Caballero, AgustinSantiago, James PatrickGarcia-Deibe, AnaSantino, AngeloGarcía-Díaz Barriga, GerardoSantocki, MichalGarcía-Domínguez, MarioSantoni-Rugiu, EricGarcia-Estañ, Luis PerezSantoro, NicolaGarcia-Fruitos, ElenaSantoro, RosariaGarcia-Gallego, SandraSantos De Almeida, TâniaGarcía-García, AndrésSantos López, Jorge ArturoGarcía-García, Rosa MaríaSantos Vizcaíno, EdortaGarcia-Garrido, Jose ManuelSantos, Bruno A C MGarcia-Gil, MercedesSantos, CarlaGarcia-Giralt, NataliaSantos, Carla S.Garcia-Gonzales, CarlosSantos, HugoGarcía-López, RodrigoSantos, JerranGarcia-Molina, AntoniSantos, LuisGarcia-Orduña, PilarSantos, MarianaGarcía-Otín, Ángel LuisSantos, PauloGarcia-Redondo, Ana BelenSantos, RosárioGarcia-Sanchez, AsuncionSantos-Coquillat, AnaGarcía-Sánchez, SusanaSantos-Juanes, JorgeGarcía-Sancho, JavierSantos-Ruiz, LeonorGarcia-Sherman, MelissaSantovito, GianfrancoGarcía-Silva, SusanaSantucci, AnnalisaGarcia-Sosa, AlfonsoSanz Luque, EmanuelGarcía-Villalón, Angel LuisSanz-González, Silvia M.Gard, PaulSanzo, AntonioGardiner, Amy SSaotome, MasaoGardiner, JasonSapkota, KiranGardner, Andrew F.Saponara, SimonaGardón, Juan CarlosSaponaro, ConcettaGareri, PietroSapone, AnnaGarg, KoyalSapozhnikov, AlexanderGargiulo, ValentinaSara, FranceschelliGarí, EloiSara, ZalbaGarikipati, VenkataSarafidis, Charalampos S.Garinis, GeorgeSarapultsev, AlexeyGarino, CristianoSaravanan, Prathab BalajiGarita-Hernandez, MarcelaSarbini, ShahrulGarmanchuk, L. V.Sarde, Claude-OlivierGarn, HolgerSardeli, ChrysanthiGarncarek, ZbigniewSardinha Francisco, CarolinaGarofalo, CeciliaSardu, CelestinoGarozzo, DomenicoSaretzki, GabrieleGarre, VictorianoSarhan, DhifafGarrett, ScottŠarić, AnkicaGarriga, PereSarkadi, BalázsGarrity, DeborahSarkar, AbhijitGartel, AndreiSarkar, AmritaGarvalov, Boyan K.Sarkar, AurijitGarweg, Justus G.Sarkar, DevanandGarzon, SimoneSarkar, DhrubaGarzon-Muvdi, TomasSarkar, SibajiGas, PiotrSarkar, SusobhanGąsecka, AleksandraSarkes, Deborah A.Gash, Don MSárközy, MartaGashti, Mazeyar ParvinzadehSarma, PranjalGasic, KsenijaSarma, SauravGąsior-Perczak, DanutaSarmati, LoredanaGasiūnas, GiedriusSarmento-Ribeiro, Ana BelaGaspar, KrisztianSarmiento Soto, ManuelGaspar, Maria ManuelaSarna, MichalGáspári, ZoltánSarna, TadeuszGasparri, FabioSarnataro, DanielaGasparri, RobertoSarniguet, AlainGasparski, AlexanderSarnow, PeterGasser, Susan MSarnowski, TomaszGasset, MariaSarti, Alba ClaraGassman, NatalieSartini, DavideGa̧stoł, MaciejSasaki, HirokiGasull, XavierSasaki, KotaroGaszner, BalázsSasase, TomohikoGates, ColinSasca, DanielGatti, AntoniettaSase, SunetraGatto, FrancescaSassi, MohamedGatzka, MartinaSasso, Ferdinando CarloGaubert, AlexandraSastre, LeandroGauda, EstelleSastre-Santos, AngelaGaudin, RobertSatake, AkikoGaudio, AgostinoSathyanesan, AaronGautam, MukeshSatish, LakkakulaGautam, PrsonSatitsuksanoa, PattrapornGautheron, JérémieSato, AkinoriGauthier, AdrienSato, EiichiGauthier, BenoitSato, FuyukiGauthier, ThierrySato, HiromiGautier, MathieuSato, KeisakuGavalas, AnthonySato, Ken-ichiGavara, LaurentSato, ShuzoGavira, JoséSato, TakashiGaviraghi, MarcoSato, TakuichiGavriilaki, EleniSatoh, AkiraGawel, DamianSatoh, KatsuyaGawel, KingaSatoh, TadashiGawlik, AnetaSatou, AkiraGawlik-Dziki, UrszulaSattar, SampurnaGay, FrancescaSattler, SusanneGay-Quéheillard, JeromeSattler, WolfgangGaze, David C.Satué, KatiuskaGazerani, ParisaSatuito, Cyril Glenn P.Gazouli, MariaSaturnino, CarmelaGazzin, SilviaSaúde, LeonorGdaniec, ZofiaSauder, ChristianGe, XiaodongSauer, DanielGeary, SeanSauer, Pieter JjGebert, JohannesSaule, SimonGedara, PradeepSaulle, IrmaGee, Christine ESaurav, KumarGeest, Ferdy VanSaussez, SvenGeetha, ThangiahSauzay, ChloéGęgotek, AgnieszkaSavaşan, SüreyyaGehlert, SebastianSavatin, Daniel-ValentinGehring, ChristophSavchenko, Tatyana V.Geisberger, RolandSavilahti, HarriGelissen, IngridSavoi, StefaniaGenco, Caroline AttardoSavoia, PaolaGendaszewska-Darmach, EdytaSavopoulos, ChristosGendek-Kubiak, HannaSavu, DianaGenders, AmandaSaw, Constance L.L.Geng, XiaokunSawada, KazuhikoGennari, AlessandraSawada, KojiGennari, LuigiSawamura, Kentaro-ichiGennery, AndrewSawosz, EwaGenova, TullioSawynok, JanaGenovese, CarloSaxena, SugandhaGenovese, TizianaSayar, HamidGent, MartinSayner, Sarah L.Gentile, AlessandraSazonova, Margarita A.Gentile, CarlaScacco, SalvatoreGentile, LucaScaglione, Kenneth MattGentile, PietroScaini, GiselliGentile, VittorioScala, AngelaGentilin, EricaScalise, MariafrancescaGentle, Ian E.Scalschi, LoredanaGentry, Matthew S.Scandurra, RobertoGentzsch, MartinaScarafoni, AlessioGeoerger, BirgitScarcia, PasqualeGeorge, AnneScarinci, FabioGeorgescu, AdrianaScarpa, ElenaGeorgiev, Georgi As.Scarpellini, EmidioGeorgieva, MilenaScarpellino, GiorgiaGeorgieva, RadostinaScarselli, MarcoGeorgii, ElisabethScatena, RobertoGeppert, MarkSchaaf, Gerben J.Gera, RuchiSchachner, AdenaGeraci, FabianaSchade, DennisGeraghty, PatrickSchaefer, MatthiasGerasimaitė, RūtaSchaefer, MichaelGerber, AndréSchäfer, KatrinGerbino, AndreaSchäfer, ReinholdGerbst, Alexey G.Schaffer, AriGerding, HeinrichSchaft, NielsGerdol, MarcoSchaftingen, Emile VanGeremia, SilvanoSchaiquevich, PaulaGereñu, GorkaSchallner, NilsGergs, UlrichSchänzer, AnneGerisch, GüntherScharf, BirgitGerli, MattiaSchaub, Günter A.Gerlich, WolframScheau, CristianGerling, MarcoSchedel, MichaelaGerloff, DennisScheel-Krüger, JørgenGermain, LucieScheffel, JörgGermain, PierreScheiner, SteveGerman, Nadezhda A.Schellino, RobertaGermanidis, GeorgiosSchemmer, PeterGermano, GiovanniSchempp, Christoph M.Germini, DiegoSchepisi, GiuseppeGerriets, ValerieScherbak, NikolaiGervois, PascalScherer, GüntherGescher, AndreasSchernthaner, JohannGesi, CamillaScherthan, HarryGeso, MoshiScherz-Shouval, RuthGessani, SandraScheuer, TillGessler, FlorianScheuring, DavidGessner, GuidoSchiaffino, StefanoGeta, David A.Schiattarella, AntonioGetz, GodfreySchibler, UeliGhadieh, Hilda E.Schicho, RudolfGhai, VikasSchicker, KlausGhaisas, ShivaniSchierwagen, RobertGhaleh, BijanSchiewe, MitchelGhandhi, ShanazSchiff, Elena R.Ghandour, RashedSchiffer, LinaGhantous, YasmineSchiffmann, RaphaelGhasemi, RasoulSchikov, AlexanderGhatak, ArindamSchildberg, Frank A.Ghavami, SaeidSchildgen, OliverGhayor, ChafikSchillaci, DomenicoGhelli Luserna Di Rorá, AndreaSchilling, Arndt FriedrichGherasim, CarmenSchindl, RainerGhesquiere, BartSchindler, Charles W.Ghezzi, DanieleSchipper, KerstinGhiani, CristinaSchippers, AngelaGhiasi, Seyed MojtabaSchirhagl, RomanaGhidoni, RiccardoSchirmer, EricGhigna, ClaudiaSchito, Anna MariaGhigo, EricSchjenken, John E.Ghilli, MatteoSchlachetzki, JohannesGhinet, AlinaSchlaepfer, DavidGhiran, Ionita C.Schlaepfer, Isabel RGhiroldi, AndreaSchlegel, JürgenGhislat, GhitaSchlegel, PatrickGhobadloo, Shahrokh MohammadzadehSchlein, ChristianGholami, FatemehSchlicker, EberhardGhomi, MahmoudSchlitt, HansGhonaim, HassanSchlothauer, TilmanGhosh, ArnabSchlunk, FriederGhosh, RajeshwarySchmalhausen, E.V.Ghosh, SantoshSchmid, RafaelGhosh, SomiranjanSchmid, RalfGhosh, SoumitaSchmidt, BorisGhosh, SubhajitSchmidt, EdGhosh, SumitSchmidt, Jonas DamgårdGhoshal, KalpanaSchmidt, MarcusGiacobini, Ezio G.Schmidt, ThomasGiacomazza, DanielaSchmidt-Bleek, KatharinaGiacomelli, LucaSchmidtke, GunterGiacomello, EmilianaSchmidts, MiriamGiacomini, ElisaSchmiedl, AndreasGiacomo, Viviana DiSchmitt, EmmanuelleGiagulli, CinziaSchmitt, Nicole CGiallauria, FrancescoSchmittgen, ThomasGiamberardino, Maria AdeleSchmitt-Ulms, GeroldGiamogante, FlaviaSchmitz, IngoGiampieri, EnricoSchmitz, MatthiasGianesello, LisaSchmitz, ThomasGianfredi, VincenzaSchnabelrauch, MatthiasGiangrande, AngelaSchneider, AnjaGianluca, CarnevaleSchneider, BarbaraGiannaccare, GiuseppeSchneider, BohdanGiannakouros, ThomasSchneider, Elena K.Giannattasio, MicheleSchneider, MartinGiannetto, ClaudiaSchneider, RobertGiannis, AthanassiosSchneider, SimonGiannoni, ElisaSchneider-Maunoury, SylvieGiannoni, FedericoSchnieke, Angelika E.Giannoutsou, EleniSchoenaers, SébastjenGianoncelli, LetiziaSchoepp-Cothenet, BarbaraGiansanti, LuisaSchohn, HervéGianturco, LuigiScholey, JamesGiardina, PaolaScholz, Carsten C.Giarola, ValentinoScholze, AlexandraGibbs, Bernhard F.Schomburg, LutzGibellini, LaraSchouten, AlexanderGibney, NoelSchrader, AndreaGibson, Angela L. F.Schrama, DavidGibson, Susan I.Schramm, AlexanderGiebultowicz, Jadwiga M.Schreck, Karisa C.Giełdoń, ArturSchreiber, MartinGieras, AnnaSchrezenmeir, JuergenGierszewska, MagdalenaSchröder, AgnesGigli-Bisceglia, NoraSchröder, ChristianGiglio, PierreSchröder, Henrik DaaGigliotti, Casimiro LucaSchroeder, AnnaGijón-Nogueron, GabrielSchroeder, HilkeGil Fernández, VanessaSchroer, SibylleGil, Jose FernandoSchröpfer, SusanGil, JustynaSchrum, AdamGil, KrzysztofSchubert, AndreaGil, MinchanSchubert, Frank RichardGil, RobertSchubert, MarioGil, RosarioSchuchardt, MirjamGilbert, CarolineSchuck, Robert N.Gilbo, NicholasSchueler, JuliaGiles, WayneSchulte, ChristianGiliopoulos, DimitriosSchulte, MarvinGill, HarsharnSchultz, David JGilli, FrancescaSchultz, Kris Ann P.Gilloteaux, JacquesSchulz, BurkhardGil-Mohapel, Joana M.Schulz, FlorianGilot, DavidSchulz, HerbertGils, Janine VanSchulze, MargitGimpel, MatthiasSchulze-Tanzil, GundulaGinaldi, LiaSchulze-Tanzil, Gundula GesineGindraux, FlorelleSchumacher, JuliaGinebra, María PauSchumann, CananGiner-Tarrida, LuísSchunke, Kathryn JaquesGinsberg, StephenSchuren, FrankGinzburg, Yelena ZSchuss, PatrickGiordano, ElenaSchutt, AmyGiordano, EmanueleSchutte, StaceyGiordano, FlavioSchwaab, JulianaGiordano, MariaSchwalbe, Ruth A.Giordano, RaffaeleSchwaminger, SebastianGiordano, SalvatoreSchwan, StefanGiorgetti, AlejandroSchwan, WilliamGiorgi, MauroSchwans, Jason P.Giorgi, Ugo DeSchwartz, Lawrence M.Giorgianni, FrancescoSchwarz, NicoleGiorgini, ElisabettaSchwarz, PatrickGiovacchini, GiampieroSchwarz, Steven M.Giovannelli, PiaSchwarzbacherová, VieraGiovannetti, ElisaSchweitzer-Stenner, ReinhardGiovannini, AnnalisaSchweizer, FrankGiovannini, CatiaSchweizer, MichaelGiovannoni, RobertoSchwind, SebastianGiovannucci, David R.Sciacca, Michele Francesco MariaGiovarelli, MatteoScialla, StefaniaGiovarelli, MirellaSciamanna, GiuseppeGiovino, AntonioSciascia, SavinoGiraldo, PilarŚcibisz, IwonaGirao, HenriqueScicali, RobertoGirault, AlbanScichilone, NicolaGiri, BanabihariScieglinska, DorotaGiri, HemantScilabra, Simone D.Girolami, MarcoScioli, Maria GiovannaGiron-Gonzalez, Maria D.Sciortino, AliceGiroud, MaudeSciortino, Maria TeresaGisbert-Garzarán, MiguelScioscia, CrescenzioGish, RobertScipioni, AnitaGisriel, ChristopherSciubba, FabioGisselbrecht, ChristianSciumè, GiuseppeGiubellino, AlessioScornik, Fabiana SGiudice, Linda C.Scorziello, AntonellaGiudice, ValentinaScott, EmmaGiudici, FabiolaScott, IainGiuffrè, MauroScotti, MarcusGiuffrida, Maria LauraScotti, NunziaGiuffrida, PaoloScotto D’Abusco, AnnaGiuffrida, RosarioScozzafava, AndreaGiugliani, RobertoScrano, LauraGiuliani, Anna LisaScribano, DanielaGiulianini, Piero GiulioScrima, MarioGiuliano, Michela M.Scuderi, CaterinaGiurg, MirosławScuderi, SorayaGiuseppina, BasiniScudiero, RosariaGiusti, IlariaScully, ErinGjorgjieva, MonikaScuoppo, ClaudioGkaliagkousi, EugeniaScuruchi, MicheleGkika, DimitraScuteri, AriannaGłąbska, DominikaScuto, MariaGladish, Daniel K.Scutt, CharlieGladkikh, IrinaSczepanski, JonathanGladysheva, Inna P.Seaby, EleanorGładysz, AnnaSeale, Andre P.Glaser, BrunoSeale, Lucia A.Glass, DonaldSebastian, AimyGlass, JonSebastián, DavidGlatt, SebastianSebastianelli, ArcangeloGlavač, DamjanSebastiani, FedericoGlazar, IrenaSebastiani, MarcoGlazińska, PaulinaSecci, DanielaGlazunova, Olga A.Secondo, AgneseGlazyrin, YurySeco-Rovira, VicenteGliszczynska, AnnaSedda, GiuliaGlossmann, HartmutSedic, MirelaGlover, Anthony R.Sedlačík, MichalGłowacka, KatarzynaSedlar, KarelGlubb, DylanSedlářová, MichaelaGlubb, Dylan M.See Hoe, Louise E.Gluck, JessicaSeeliger, ClaudineGlukhov, AlexeySeene, TeetGlukhova, Olga E.Seers, ChristineGlushakov, AlexanderSefat, FarshidGnanaprakasam, JayaSegarra-Marti, JavierGnanasundram, Sivakumar VadivelSegatto, MarcoGnani, DanielaSeger, RonyGniadecki, RobertSegers, VincentGniazdowska, EwaSegura, InmaculadaGobbi, AlbertoSegura, Miguel FGoberdhan, DeborahSehlin, DagGoc, AnnaSeidel, KayGoda, KatalinSeidel, Rüdiger W.Goda, KeisukeSeidl, MaximilianGoddard, AlanSeifert, Georg J.Godena, SaraSeifi, HamedGoderska, KamilaSeixas, AnaGodlewska, MarlenaSekar, ShobanaGodlewski, JanuszSekatski, SergueiGodos, JustynaSeki, EkihiroGoeser, FelixSekine, MasamiGoettig, PeterSekine, ShioriGoetzman, Eric S.Sekino, YoheiGoffinet, ChristineSekiya, SachikoGoggin, Danica E.Sellars, JonathanGoglia, FernandoSelleri, SilviaGojo, SatoshiSellner, JohannGok, OzgulSeluanov, AndreiGolay, JoseeSelva-O’Callaghan, AlbertGolda, AnnaSelvarajah, DineshGoldberg, ErwinSelzer, Michael E.Goldenkova-Pavlova, IrinaSembratowicz, IwonaGolding, Jon P.Semenkovich, Clay F.Goldstein, AllanSemenyuk, PavelGoldstein, Andrew S.Semeraro, PaolaGołembiowska, KrystynaSemeril, DavidGolestaneh, NadySemih, EsinGolgovici, FlorentinaSemlali, AbdelhabibGolias, TerezaSemple, RobertGolinelli, Marie-PierreSempruch, CezaryGolla, UpendarSemsarilar, MonaGolokhvast, KirillSena, ArmandoGolombick, TerrySenapati, ParijatGolovleva, IrinaSenbonmatsu, TakaakiGolubkina, Nadezhda A.Senel, MehmetGolubnitschaja, OlgaSenger, MoritzGolumbeanu, MonicaSengupta, ArjunGomaraschi, MonicaSengupta, SonaliGombart, AdrianSensini, AlbertoGombert, AlexanderSenter, Peter DGomes, Joao RSeo, Cheong HoonGomes, Manuela E.Seo, DaisukeGomes, Paula A. C.Seo, Goo-YoungGomes, PedroSeo, Hak SooGomes, SóniaSeo, HeewonGómez Castellà, CristinaSeo, Je HoonGomez Del Moral, ManuelSeo, JungwonGómez García, Carlos J.Seo, Sang WooGomez Roldan, Maria VictoriaSeo, ShigemiGomez, GuillermoSeoane-Collazo, PatriciaGomez, NidiaSeong, Ki MoonGómez, Pilar SánchezSequeira, Ivana R.Gómez-Barrero, SusanaSerafini Fracassini, DonatellaGomez-Gil, VeronicaSerba, DesalegnGómez-González, BelénSerban, Daniela ElenaGomez-Nino, AngelaSerefko, AnnaGómez-Pastor, RocíoSereno, DenisGómez-Puertas, PaulinoSergeant, KjellGómez-Sagasti, María T.Sergeev, YuriGómez-Zorita, SaioaSergeeva, Marina G.Gomoiu, IoanaSergeeva, OlgaGomzikova, MarinaSerino, GiovannaGonano, LuisSerio, GabriellaGonçalves, Carlos AlbertoSério, SusanaGonçalves, LídiaSerpente, MariaGonçalves, M. Sameiro T.Serpersu, Engin HalitGonçalves, Nádia P.Serra, DianaGonçalves, RenatoSerra, GregorioGonçalves, SóniaSerra, MassimoGonda, SándorSerradji, NawalGondhalekar, Ameya D.Serrador Peiró, Juan M.Gondi, Christopher SSerrano, Antonio L.Gong, LiangweiSerrano, AurelioGong, Siew-gingSerrano, Dolores R.Gong, ZifanSerrano, Javier LeonGONGAL, GyanendraSerrano, Juan Alfonso AyalaGoni, FelixSerrano, MaríaGoñi, Fernando RSerrano, MercedesGonkowski, SławomirSerrano-Aroca, ÁngelGono, TakahisaSerrano-Mislata, AntonioGonsalves, Wilson I.Serrato, AntonioGonzalez Alvarez, IsabelSertic, SarahGonzalez Mariscal, IsabelSertorio, MathieuGonzalez, AntonioSeruggia, DavideGonzalez, DeyarinaServe, KintaGonzalez, Diego LuisServent, DenisGonzález, Florenci V.Servín-Garcidueñas, Luis EduardoGonzalez, GabrielSeshadri, MukundGonzález, J.F.Šestan, NenadGonzález, JerónimoSeth, Punit P.González, MartaSethi, SunjayGonzalez, Martin VillalbaSetiadi, HendraGonzalez, RogelioSetny, PiotrGonzalez, VictorSeto, ToshiyukiGonzalez-Barcala, Francisco-JavierSetzer, William N.Gonzalez-Bulnes, AntonioSeubert, John MGonzàlez-Duarte, RoserSeung-Taek, LeeGonzalez-Escamilla, GabrielŠevčík, JanGonzález-Fernández, LauroSevcikova, SabinaGonzález-García, IsmaelSeverini, CinziaGonzález-Iglesias, HéctorSeverino, BeatriceGonzález-Lafont, ÀngelsSeverino, PaoloGonzalez-Menendez, PedroSeverson, Aaron F.González-Minero, Francisco JoséSevigny, MaryGonzalez-Molina, JordiSevilla, MichaelGonzález-Moro, María BegoñaSevostyanova, VictoriaGonzález-Navajas, José M.Seydel, TiloGonzález-Navarro, HerminiaSeyoum, BerhaneGonzález-Nieto, DanielSferruzzi Perri, AmandaGonzález-Sarmiento, RogelioSgambato, AlessandroGonzález-Sarrías, AntonioSgarbossa, AntonellaGonzález-Vázquez, ArlyngSgodda, MalteGonzalez-Vicente, AgustinSgouros, JosephGoodla, LavanyaSgrignani, JacopoGoodman, Cynthia L.Shabbir, WaheedGoodman, Richard E.Shackelford, JuliaGoodrich, JaclynShafahi, AliGoodwin, ChristopherShafer, William M.Góra, ArturShafiq, SarfrazGóra, Michalina J.Shafique, MichaelGoradia, NishitShah, AjitGora-Sochacka, AnnaShah, DilipGorbacheva, LiubovShah, Furqan A.Gorbatyuk, Oleg S.Shahbaaz, MohdGordeeva, OlgaShahzad, BabarGordon, Darren M.Shaik, Anver BashaGorelik, JuliaShaikh, SibhghatullaGorgieva, SelestinaShakibaei, MehdiGorgoulis, VassilisShakya, AkhileshGorini, FrancescaShakya, AnishaGorio, AlfredoShamay, YosefGoris, TobiasShamieh, SaidGorji, AliShanbhag, VinitGorman, AdrienneShanmugam, GnanendraGornati, RosalbaShanmugam, GobinathGornowicz, AgnieszkaShannon, OonaghGorovits, BorisShao, FangweiGorrell, MarkShao, HaipengGorris, MarkShao, LijianGorshkova, Ekaterina N.Shao, WeiGorza, LuisaShao, Wen HaiGosav, Evelina MariaShapiro, Jason W.Gosciniak, GrazynaShapiro, Joseph IGośliński, TomaszSharaf, KariemGossman, ToniSharan, ShyamGoto, KatsumasaSharbeen, GeorgeGoto, KenichiSharif, JafarGoto, RiekoSharma, AnchalGoto, YusukeSharma, AnketGoto-Inoue, NaokoSharma, AnuragGötte, MartinSharma, ArishyaGottikh, MarinaSharma, Bhesh RajGötz, Klaus-PeterSharma, DileepGotzias, AnastasiosSharma, GeetanjaliGoua, MarieSHARMA, HIMANSHUGoudarzi, MaryamSharma, IshaGouguet, PaulSharma, KrishnaGoukassian, David A.Sharma, NeeruGould, Kathleen L.Sharma, PrasantGould, Robert W.Sharma, PreetiGourdie, RobertSharma, PriyankaGoureau, OlivierSharma, RitinGourmans, Marie-JoséSharma, SanjivGous, PeterSharma, ShwetaGout, IvanSharma, SudhaGouveia, Alexandra M.Sharma, SumanaGovindan, PrasanthSharma, Sunil K. Govindhan, GanesanSharman, Edward H.Gowda, ChandrikaSharova, NataliaGoyal, EtikaSharshov, KirillGoyal, RamanShaulsky, EvyatarGoyal, YogeshShavandi, AminGozzini, AntonellaShavrukov, YuriGrabarek, Beniamin OskarShaw, SubrataGrabarek, Joanna B.Shcharbin, DzmitryGrabauskas, GintautasShcherbakova, Larisa A.Grabiec, UrszulaShcherban, Andrey B.Grabner, Daniel S.She, MaoyunGrabowski, SlawomirSheardy, RichardGrabrucker, Andreas M.Shearer, Aiden EliotGraczyk-Jarzynka, AgnieszkaShearer, JasonGrad, SibylleSHEATS, KatieGradia, DanielaShehadeh, Lina A.Gradoni, LuigiShei, Ren-JayGradov, Oleg V.Shekhova, ElenaGradova, MargaretShekhtman, AlexanderGraether, SteffenSheldrake, HelenGrafi, GideonShelenkov, AndreyGragnano, FeliceShelke, Ganesh VilasGraier, WolfgangShellard, AdamGramatica, PaolaShelomi, MatanGramignoli, RobertoShelton, HollyGramolelli, SilviaShen, Che-Kun JamesGramsbergen, JanShen, HaihongGranado, Jose Maria GonzalezShen, JianqiangGranados-Principal, Sergio M.Shen, LishuangGrande Tovar, Carlos DavidShen, Ming-YiGrande, RaffaeleShen, RongGrangette, CorinneShen, YangGranhag, LenaShen, YiGranito, AlessandroSheng, KuanweiGrant, GeorgeSheng, YueGrant, WilliamSheng, ZhanwuGranzier, Henk L.Shenkman, Boris S.Grãos, MárioShenolikar, ShirishGraphodatsky, AlexanderSheppard, Hilary (Billy)Grasman, JonathanSher, Yuh-PyngGrasshoff, ChristianSherbet, Gajanan V.Grassi, FabrizioSherman, JonathanGrassi, FrancescoSherr, David H.Grasso, GiuseppeSheshadri, NamrathaGratchev, AlexeiShete, VarshaGrattagliano, IgnazioShetty, ShishirGrau, VeronikaSheu, JimGraves, Steven WSheu, Joen-RongGray, Douglas A.Sheu, Shey-ShingGray, Janet M.Sheval, EugeneGray, RachaelShewan, Louise G.Grayson, Dennis R.Shi, ChunlinGraziano, GiuseppeShi, GongjunGrazul-Bilska, AnnaShi, HaitaoGrčević, DankaShi, JianGrebenkov, Denis S.Shi, JunchaoGreb-Markiewicz, BeataShi, JunfengGreco, MartaShi, KuangyuGreco, PantaleoShi, LeiGreco, SimonaShi, WenGreco, ToddShi, XiaoleiGreco, VivianaShiao, Chih-ChungGreen, ErinShiao, Young-JiGreen, MichaelShiba, KiyotakaGreen, Nicola H.Shibaev, AndreyGreenhalgh, Andrew DavidShibata, AtsushiGreenlee, Justin J.Shibata, HiroshiGreenlee-Wacker, MallaryShibuya, AkiraGreenwood, Michael T.Shidoji, YoshihiroGregis, VeronicaShieh, Jeng-jerGrégoire, SébastienShiina, TakashiGregorini, MarilenaShikov, AlexanderGregorio, Gálvez-ValdiviesoShikov, Alexander N.Gregório, MicheleShiku, HitoshiGregory, Christopher DShim, Jae-HyuckGregory, StephenShim, Joon W.Gregory, StevenShima, SeigoGreiner, Jack V.Shimada, IchioGreiner, JohannesShimada, YasuhitoGreisen, Per JrShimada, YohtaGreither, ThomasShimamura, TakeshiGrelet, SimonShimba, ShigekiGrewal, Raji PaulShimbo, TakashiGrewal, ThomasShimek, JoannaGribsholt, Sigrid BjergeShimizu, AnekoGriffis, Eric R.Shimizu, KazuyukiGriffiths, Cara A.Shimizu, ShunichiGriffiths, RianShimizu, TakeyukiGrignani, GiovanniShimizu, TatsuyaGrigorieva, Elvira V.Shimizu, YujiGrigsby, Christopher L.Shimizu-Ibuka, AkikoGrill, MagdalenaShimmi, OsamuGrillo, GiorgioShimo, TsuyoshiGrimaldi, Anna MariaShimoda, HiroshiGrimaldi, AnnalisaShimojo, MasahitoGrimaldi, PaolaShimosawa, TatsuoGrimanelli, DanielShimose, ShigeoGrimaudo, Maria AuroraShimura, TatsuoGrimes, Mark L.Shin, Dong-HyunGrimholt, UnniShin, Meong CheolGrimm, ChristinaShin, SehyunGrimm, MarcusShin, Young JooGrimm, Marcus O.W.Shinde, AparnaGrimm, W. D.Shinde, Arti V.Griñán-Ferré, ChristianShinde, SuhasGrinberg, DanielShingu, TakashiGrippo, Paul JShingu, YasushigeGris, DenisShinohara, AkiraGrisk, OlafShinohara, MitsuruGritli-Linde, AmelShinohara, ShogoGrivas, PetrosShinomiya, NariyoshiGrizzi, FabioShinozaki, JunichiGrob, KoniShin-Yi, MarzanoGrobet, LucShioda, NorifumiGrodzik, MartaShiomi, DaisukeGroen, RichardShippy, TeresaGrogan, GideonShirahata, TatsuyaGroh, Isabel Anna MariaShirai, YasuhitoGrolli, StefanoShirasuna, KoumeiGrones, PeterShiraz, ParveenGroome, JamesShirmanova, Marina V.Grootveld, MartinShirokikh, Nikolay E.Gros, Stephanie J.Shirole, NitinGroschner, KlausShirvani, Arash H Grose, JulianneShishido, Tânia K.Grošelj, UrhShishova, Maria F.Gross, Briana L.Shivapurkar, NarayanGross, GideonShivram, HaridhaGross, Joshua B.Shiwaku, HirokiGross, Stephane R.Shkolnik, DoronGrossbach, AndrewShmarakov, IgorGrosse-Brinkhaus, ChristineShmatov, MikhailGrossi, ValentinaShmidt, EugeniaGrossman, Ashley BarryShnyder, SteveGrosso, FilipaShoaib, MuhammadGroszewicz, Pedro B.Shojaei, ShahlaGrothe, ClaudiaShorter, JamesGrötzinger, CarstenShree, SonalGroux-Degroote, SophieShridhar, SurekhaGroveman, BradleyShtam, TatianaGrubbs, NathanielShu, SherryGrube, SusanneShuh Ichi, NishikawaGruber, AnsgarShui, Hao-aiGrubić Kezele, TanjaShukla, ArvindGrubman, MarvinShukla, Pushp SheelGrudnik, PrzemyslawShukla, SiddharthGrul’ová, DanielaShukla, SurendraGrünert, UlrikeShulman, AdrianGrunewald, RichardShults, Nataliia V.Grunin, MichelleShutang, TanGrunst, MelissaShuvaev, Anton N.Gruntenko, NataliyaSiano, FrancescoGruszczynska-Biegala, JoannaSiatkowski, IdziGruszka, Alicja MSiaugue, Jean-MichelGruszka, DamianSibon, Ody C.M.Gryta, MarekSicari, DariaGrywalska, EwelinaSiciliano, CarloGrzegrzółka, JȩdrzejSiddappa, ByrareddyGrzela, Dawid PSiddharth, SumitGrzeskowiak, BartoszSiddharthan, VenkatramanGrzyb, JoannaSiddiqi, Muhammad ZubairGu, BinSiddiqui, ImranGu, ChunjuSiddiqui, Rafat A.Gu, FengSidelmann, Johannes JakobsenGuadagni, StefanoSidles, Sara J.Guadagno, EliaSidorenko, Viktoriya S.Guagnano, Maria TeresaSidorkiewicz, IwonaGualerzi, Claudio O.Sidoti, AntoninaGualillo, OresteSieber, Alisa-NaomiGuallar, DianaSiebert, Hans-ChristianGuan, QingdongSiegel, AmandaGuangbi, LiSiegers, Gabrielle M.Guardado-Calvo, PabloSiegman, Marion J.Guardia, CarlosSiemer, Ansgar B.Guarnieri, SimoneSiemianowski, OskarGuarnieri, TizianaSieni, ElisabettaGuarraci, FaySieniawska, ElwiraGubanska, IgaSieprawska, ApoloniaGubareva, ElenaSier, CornelisGubin, DenisSierecki, EmmaGucciardo, ErikaSiesto, GabriellaGudbergsson, Johann MarSignore, GiovanniGude, NatalieSignore, MicheleGudermann, TSignorile, AnnaGuéant, Jean LouisSignorini, CinziaGuemez, AliciaSikalidis, AngelosGuérardel, YannSikazwe, DonaldGuerin, CoralieSikora, MariuszGuerlesquin, FrancoiseSil, PayelGuerra, CarmenSil, Susmita RGuerra, FloraSilachev, DenisGuerra-Librero, AnaSilaghi, Ciprian N.Guerreiro, Joana FSilas Fernandes, EtoGuerrero, CarmenŠiler, BranislavGuerrero-Plata, AntonietaSiligardi, GiulianoGuerriero, GiuliaSiliqi, DritanGuerriero, JenniferSiljamäki, ElinaGuerrini, GabriellaSillence, Dan J.Guest, DavidSillerud, Laurel O.Guevara González, RamónSillo, FabianoGugliandolo, AgneseSilva, Adriana RibeiroGugliandolo, EnricoSilva, Alexandre RGugnoni, MilaSilva, CatarinaGuha, PrasunSilva, Nuno A.Guiamet, Juan-JoséSilva, Nuno Helder Da Cruz SimõesGuibert, ChristelleSilva, PaulaGuida, FrancescaSilva, Pedro JorgeGuida, MicheleSilva, SaraGuiderdoni, EmmanuelSilva, Simone S.Guidolin, AlineSilva, Susana N.Guidolin, DiegoSilva, Susana NunesGuignard, GaëtanSilva-Abreu, MarcelleGuillamat-Prats, RaquelSilvagno, FrancescaGuillén, María IsabelSilvas, Jesus A.Guillermet Guibert, JulieSilvestre, Miguel AngelGuillon, ChristopheSilvestri, BrigidaGuillot, XavierSilvestri, CristianGuillou, HerveSima, LiviaGuinovart, Joan JSimal, JesusGuisasola, EduardoSimard, MarcGuix, Francesc XavierSimeoli, RaffaeleGujas, BojanSimeone, PasqualeGulati, SachinSimeonova, IvaGüldiken, NurdanSimes, DinaGulei, DianaSimioni, CarolinaGulic, TamaraSimirgiotis, Mario J.Gulin, Alexander A.Simitzi, CharaGulino, Ferdinando AntonioSimkin, AndrewGulino, RosarioSimko, FedorGullberg, DonaldSimkó, MyrtillGullbrand, Sarah E.Simlat, MagdalenaGuller, AnnaSimmen, Rosalia C.M.Gullì, MariolinaSimmons, Peter A.Gully, DjamelSimo, LadislavGulyaeva, Natalia V.Simões, Marta FilipaGumienny, TinaSimoes, SandraGumulec, JaromirSimon, IstvanGunaje, JayaramaSimon, IstvánGunasekar, Susheel K.Simon, JorgeGundamaraju, RohitSimon, MichelGundersen, Cameron B.Simon, ThomasGünes, CagatayŠimoník, OndřejGuney, EmreSimonini, SaraGunia-Krzyżak, AgnieszkaSimonsen, Anja HviidGunn, Alistair J.Simopoulos, ArtemisGunning III, William TSimopoulou, MaraGunning, PeterSimova-Stoilova, Lyudmila PetrovaGunn-Moore, DanielleSimpson, A. JohnGuns, Pieter-Jan D.Simpson, Julie E.Günzel, DorotheeSimpson, Melanie RaeGuo, BinSims, NatalieGuo, FenghaiSims-Lucas, SunderGuo, QiuquanSinanoglou, VassiliaGuo, TingSince, MarcGuo, TingtingSinclair, DavidGuo, WeiŠindlerová, LenkaGuo, XiufangSing, AndreasGuo, YongSingan, Vasanth R.Guo, ZhefengSingaraju, AdityaGuo, ZhenhengSingh, AbhishekGuo, ZhongmaoSingh, AmitGupta, DevendraSingh, AmritaGupta, DipikaSingh, AnuragGupta, Gagan D.Singh, ArashdeepGupta, MohanSingh, Ashok K.Gupta, MohitSingh, Brijesh KumarGupta, NilakshSingh, ChandraGupta, NishantSingh, DeoGupta, ParulSingh, HarpalGupta, PrachiSingh, KamalendraGupta, RajatSingh, ManjitGupta, Ramesh C.Singh, ManjulGupta, RashmiSingh, NaveenaGupta, RisheinSingh, NishaGupta, SanjaySingh, PradeepGupta, SupritSingh, Puspendra P.Gupta, SwetaSingh, RatnakarGupta, Vijayalaxmi G.Singh, Rupesh KumarGupta, YashSingh, SandeepGurgul, ArturSingh, SarbjitGuriec, NathalieSingh, SeemaGurrapu, SreeharshaSingh, ShaktiGuruli, GeorgiSinha, AmitGurusamy, RamanSinha, DebottamGushchin, Artem L.Sinha, SangitaGust, Kurt A.Sini, Maria CristinaGustav, StalhammarSinico, ChiaraGustin, KurtSinjari, BrunaGutberlet, ThomasSinko, PatrickGutiérrez, LauraSinkovits, GyörgyGutiérrez-Adán, AlfonsoSinner, Debora I.Gutierrez-Aguilar, ManuelSinreih, MašaGutierrez-Camino, AngelaSintim, HermanGutiérrez-Gallego, RicardoSioofy-Khojine, AmirbabakGutiérrez-Juárez, RogerSiotto, MariacristinaGutierrez-Merino, CarlosSipos, KatalinGutowska, IzabelaSips, PatrickGutowska-Owsiak, DanutaSiracusa, RosalbaGutsaeva, DianaSiramshetty, Vishal B.Guy, AlexandreSirangelo, IvanaGuyot, BorisSirbu, OvidiuGuzhova, Irina V.Sireci, GuidoGuzik, AgnieszkaSirenko, OksanaGuzmán Ruiz, RocíoSirko, AgnieszkaGuzman, CarlosSirotkin, AlexanderGuzman, FannySirtori, CesareGuzow-Krzemińska, BeataSirvent, AudreyGwin, KatjaSissung, Tristan M.Gyselaers, WilfriedSisto, FrancescaGyselaers, Wilfried J.A.Sisu, CristinaGyula, PéterSitarek, PrzemysławHa, Bo-KeunSitia, RobertoHa, EunyoungSitzia, ClementinaHa, Jung-HeunSivakumar, ManiHa, Ki-TaeSivakumar, SushamaHa, Yun-SokSivalingam, PeriyasamyHaak, DavidSivanesan, IyyakkannuHaanes, KristianSivarajan, Sajeevan RadhaHaaparanta-Solin, MerjaSivathanu, VivekHaarmann-Stemmann, ThomasSiwek, AgataHaas, OskarSizochenko, NataliaHaase, HajoSjöberg, Bror FolkeHaastert-Talini, KirstenSjoholm, AkeHabas, KhaledSjöqvist, SebastianHack, EthanSjöström, JesperHackauf, BerndSkała, EwaHacker, UlrichSkalioti, ChrysanthiHackfort, Bryan T.Skalska-Kamińska, AgnieszkaHackl, HubertSkalski, PawełHada, MasahikoSkandalis, SpyrosHadano, ShinjiSkarżyńska, EwaHadjiargyrou, MichaelSkarzynski, Piotr HenrykHadjikakou, Sotiris KSkelding, Kathryn A.Ha-Duong, Nguyet-ThanhSkellam, ElizabethHadwiger, Jeffrey A.Skelly, DeanneHaendler, BernardSkerlavaj, BarbaraHaensch, Gertrud MariaSkladany, LubomirHafeez, BilalSklirou, AimiliaHafizi, SassanSkogman, Barbro H.Häfner, NormanSkonieczna, MagdalenaHafner-Bratkovič, IvaSkonieczna-Żydecka, KarolinaHaga, YutakaSkórczewska, KatarzynaHage, RonaldSkorokhod, OleksiiHagedorn, ClaudiaSkotak, MaciejHagerman, Randi J.Skouras, AthanasiosHaghshenas, Sami ShaffieeSkowrońska, AgnieszkaHagidimitriou, MariannaSkowrońska, KatarzynaHagos, Engda G.Skrabana, RostislavHagos, YohannesŠkrabišová, MáriaHaguet, VincentSkretas, GeorgiosHahn, HeidiSkruzny, MichalHahn, Marc BenjaminSkrzypek, EdytaHahn, OliverSkrzypek, KlaudiaHahne, JensSkrzypski, MarekHaigh, AnthonySkuja, SandraHaile, JemaneshSkundric, Dusanka S.Hailes, Helen C.Skuratovskaia, DariaHaines, JefferySkurikhin, Evgenii GermanovichHaitin, YoniSlade, DeaHajdari, AvniSlade, NedaHajduch, EricSlavik, RogerHajime, HiraseSlavokhotova, AnnaHakamada, KenichiSlechta, DeborahHalachmi, SarelŚledziński, TomaszHalász, JúliaSleigh, JamesHalder, Amit KumarSleigh, JamieHalder, AvikSlim, SmaouiHaley, Nicholas J.Śliwa, PawełHalitschke, RaykoŚliwińska-Mossoń, MariolaHalkia, EvgeniaŚliwińska-Wilczewska, SylwiaHall, DuaneŚliwka, JadwigaHall, RebeccaSloan, Erica K.Hallberg, EinarSłoczyńska, KarolinaHalle, Mari K.Słomka, ArturHaller, Steven T.Slota, Jessy A.Hallerman, EricSlotkin, R. KeithHallett, Maurice B.Słowińska, MariolaHallmann, EwelinaSlukvin, IgorHalmos, BalazsSluyter, RonaldHalmos, GáborSmaaland, RuneHálová, IvanaSmaga, IrenaHalsall, David J.Smahel, MichalHam, Won-sikSmalla, Karl-HeinzHam, Young WanSmalley, Joshua L.Hamad, AbdelSmani, TarikHamada, MasakazuSmedler, ErikHamaguchi, MasahideSmertenko, AndreiHamamouch, NoureddineŠmída, MichalHamanishi, JunzoSmietana, MichaelHamann, MartineŚmigiel, RobertHamano, SayuriSminia, PeterHamano, TadanoriSmirnova, E.AHamaoka, TakafumiSmith Callahan, Laura A.Hamar, PeterSmith, AndrewHamazaki, JunSmith, AnnHamel, Patrice P.Smith, Brittany L.Hamel, RodolpheSmith, CarolineHamidi, TewfikSmith, G. JasonHamieh, MohamadSmith, Gaynor AnnHamilton, Duane H.Smith, Jared B.Hamilton, GerhardSmith, JasonHammer, ElkeSmith, JulianneHammer, QuirinSmith, LachlanHammer, Sabine E.Smith, Lloyd M.Hammers, DavidSmith, Loren E.Hamming, JaapSmith, Michael P.Hammond, Billy R.Smith, MicholasHammond, Timothy G.Smith, Micholas DeanHammoudi Halat, DalalSmith, RachelHamon, Jean-RenéSmith, Stephen B.Hampton, Randolph Y.Smith, Wendy S.Han, ChangseokSmolle, Maria AnnaHan, DohyunSmuder, Ashley J.Han, DongSmykal, PetrHan, Dong-WookSmyrnias, IoannisHan, InboSmyth, JamesHan, JinmingSniadecki, MarcinHAN, Kyu-ManSnyder, Orman L.Han, LiangSnytnikova, Olga A.Han, MingyuanSo, JonathanHan, MiraSoares, Ana RaquelHan, PingpingSoares, MarceloHan, Sang-WookSobacchi, CristinaHan, SongSobhani, NavidHan, Soo DeokSobierajska, KatarzynaHan, Sun-YoungSobocanec, CSandraHan, XiaobinSobota, Radoslaw M.Han, YanSobotta, ŁukaszHan, YanhuiSobreira, TiagoHan, YuyanSobrido-Cameán, DanielHanai, Jun-ichiSocol, YehoshuaHanas, JaySocorro, SilviaHanba, Yuko T.Soddu, SilviaHanchate, NareshSöderberg, Magnus P.Hancock, Charles NathanSöderberg-Naucler, CeciliaHancock, JohnSoderhall, IreneHancu, GabrielSoe, KentHanda, Avtar K.Soengas, PilarHanda, OsamuSofue, TadashiHandle, FlorianSofy, Ahmed R.Handler, JohannesSoggiu, AlessioHandral, HarishSohal, SukhwinderHandschin, Martin GrossmannSoica, CodrutaHandzlik, JadwigaSokol, ElizabethHane, MasayaSokolov, AlexeyHanefeld, UlfSokolova, Ekaterina A.Haneke, EckartSokolovskaya, AlisaHanemann, OliverSolanki, SumeetHaney, Conor M.Solano, María EmiliaHanganu, DanielaSolcan, CarmenHangelbroek, Roland W. J.Soldan, Samantha S.Hanisch, Franz-GeorgSoldin, Steven J.Hanke, NinaSolé Cañadas, CarlaHankinson, OliverSolé, MontserratHankir, Mohammed KSolecki, Wojciech BarnabaHanley, ChirstopherSoleimani, ManoocherHannan, Anthony JSoler Valls, Ana JosefaHanne, HoffmannSolesio Torregrosa, Maria De La EncarnacionHansel, ChristianSolimando, Antonio G.Hansen, Keith A.Solimando, Antonio GiovanniHansen, Paul RobertSolingapuram Sai, Kiran KumarHansford, KarlSoll, DieterHansíková, HanaSollazzo, ManuelaHanson, KirstenSoloski, Mark J.Hanson, MaureenSolovieva, AnastasiyaHanson, Nancy D.Solovjeva, Olga N.Hanss, MichelSolovyev, NikolayHantusch, BrigitteSomasundarama, Dinesh BabuHanus-Fajerska, Ewa JoannaSomlyai, GáborHanyuda, AkikoSommariva, ElenaHao, MingangSon, AhjeongHao, YajingSon, YeonghoonHaorah, JamesSonah, HumiraHaq, IramSone, HidekoHaqshenas, GholamrezaSong, ByeongwoonHara, AkiraSong, Dae KyuHara, Emilio SatoshiSong, DanHarada, HisashiSong, HailongHarada, MamoruSong, JaewhanHarada, MasakoSong, JunqiHaraguchi, AkiraSong, KunhuaHaraguchi, RyumaSong, LiujiangHaraguchi, TokukoSong, LushengHaragus, HoriaSong, MinjungHarald, ClausSong, Min-SukHarandi, Vahid M. Song, Myoung-ChongHarashima, NanaeSong, RuiHarazny, Joanna M.Song, WookHarbison, Susan T.Song, XinhuaHarbuzariu, AdrianaSong, YoungsupHardee, Justin PSong, ZiyiHardies, Stephen C.Songsasen, NucharinHardingham, Jennifer E.Soni, ChetnaHardion, LaurentSoni, Kiran KumarHardonnière, KévinSonkin, DmitriyHarduin-Lepers, AnneSonnino, SandroHardwick, James P.Sonoike, KintakeHardwidge, PhilipSonowal, HimangshuHardy, John GeorgeSopper, SieghartHardy, RowanSorbi, FlaviaHare, Joshua M.Sorg, OlivierHarezlak, KatarzynaSorge, MatteoHarford-Wright, ElizabethSoria, Federico N.Hargitai, RitaSoria-Gómez, EdgarHariharan, SeethalakshmiSoriano, Jose MiguelHarijan, Rajesh KSoriano, SalvadorHarijith, AnanthaSoriano-Arroquia, AnaHarkany, TiborSorice, MaurizioHarm, StephanSoriente, AlessandraHarn, Horng-JyhSorokin, AndreyHarnor, SuzannahSorokin, VitalyHaro, DiegoSorrentino, E.Harold, DeniseSorrentino, Francesco SaverioHarpaz, DorinSorrentino, Nicolina CristinaHarper, AlanSorriento, DanielaHarrel, Stephen K.Sortino, Maria AngelaHarrington, CharlesSotgia, SalvatoreHarrington, Sophie A.Sotgiu, StefanoHarris, CarlaSoto, EnriqueHarris, Edward N.Soto-Heras, SandraHarris, William S.Soto-Hermida, AngelHarrison, Paul H. M.Sotomayor, Camilo G.Harrison-Bernard, LisaSoto-Reyes, IleanaHarrowfield, JackSottero, BarbaraHart, DavidSouazé, FrederiqueHart, Peter C.Souček, P.Hartman, MariuszSoufan, OthmanHartman, Matthew C.T.Soufan, WalidHartmann, MatthiasSoukup, AlesHartmann, RolandSoukupova, JanaHartsfield, James K.Soundararajan, MeeraHartsough, EdwardSoundararajan, PrabhakaranHartwig, ThomasSoundharrajan, IlavenilHaruhara, KotaroSoung, Nak-KyunHaruta, MiyoshiSousa Valente, João DeHarvey, AdamSousa, Sérgio F.Harvey, AlexandraSousa, Sérgio FilipeHarzer, KlausSousa, Sílvia A.Has, CristinaSousa, SofiaHasan, RaquibulSouza, PedroHasanuzzaman, MirzaSóvágó, ImreHasche, DanielSoverini, SimonaHase, YoshihiroSowa, IreneuszHasegawa, Paul M.Sowndhararajan, KandhasamyHasenstein, Karl HSoyfoo, MuhammadHashemi, MohtadinSpada, AlbertoHashemolhosseini, SaidSpadaro, FrancescaHashim, AdnanSpadiut, OliverHashim, Ibrahim A.Spagnol, GaëlleHashimoto, MakotoSpagnuolo, RoccoHashimoto, YoshitakaSpallarossa, AndreaHashizume, OsamuSpanakis, MariosHaskó, GyörgySpang, ChristophHaslwanter, DeniseSpanier, BrittaHaspula, DhanushSparagna, Genevieve C.Hassan, MohamedSparkenbaugh, Erica M.Hassell Jr., James E.Sparling, DavidHasterok, RobertSparrer, Konstantin M.J.Hastings, Michelle L.Spartalis, EleftheriosHastings, Philip J.Spartalis, MichaelHata, TamakoSparvoli, FrancescaHatano, EtsuroSpassieva, Stefka D.Hatano, NoriyukiSpassov, SashkoHatano, YuichiroSpecjalski, KrzysztofHatem, StephaneSpencer, Charles T.Hathaway, NathanielSpencer, Gaynor E.Hattori, YuichiSpencer, Peter S.Hatzipetros, TheoSpengler, GabriellaHatzl, StefanSpengler, UlrichHau, PeterSperti, CosimoHaugen, Håvard JSpetz, Carl Jonas JorgeHausauer, Amelia K.Spiegler, JulianeHauser, PeterSpiers, JamesHausmann, LudgerSpinelli, DomenicoHausner, GeorgSpinelli, Francesca RomanaHautz, TheresaSpirina, Liudmila V.Havlik, JaroslavSpizzichino, ValeriaHavunen, RiikkaSpizzirri, U GianfrancoHawi, ZiarihSpletter, MariaHawinkels, LukasSplichal, IgorHawke, Thomas JSplichalova, AllaHawrylak-Nowak, BarbaraSpradley, Frank T.Hawthorne, SusanSpratt, Donald E.Hayar, AbdullahSprenger, Georg A.Hayasaka, HarukoSprio, Andrea ElioHayashi, HisashiSprung, Carl N.Hayashi, KeitaroSquadrito, Mario LeonardoHayashi, MakotoSquassina, AlessioHayashi, Mariko KatoSquillacioti, CaterinaHayashi, MikioSquire, JohnHayashi, NagaoSreedasyam, AvinashHayashi, RyujiSreedharan, SreejeshHayashi, ShinichiroSreerama, SubramanyaHayashi, TomohikoSrinivas, JanaswamyHayashi, YujiroSrinivasan, BharaniHayden, Melvin R.PeteSrinivasan, BhuvaneshHaydon, MikeSrinivasan, RahulHayflick, Susan J.Srinivasan, SowmyaHaynes, JenniferSripathy, SmithaHaynes, KarmellaSrivastava, AkhilHazak, OraSrivastava, AkritiHazane-Puch, FlorenceSrivastava, RiteshHazubska-Przybył, TeresaSrivastava, SarikaHe, AinaSrivastava, SaurabhHe, ChunboSrivastava, VaibhavHe, HongSrivenugopal, KalkunteHe, HongliangSroda-Pomianek, KamilaHe, Jia-QiangSroka, ZbigniewHe, Jin-ShengSroka-Bartnicka, AnnaHe, JunxianSrotin, Igor S.He, MingyuSt Hilaire, Melissa A.He, ShikunSt John, James A.Heakal, YasserSt. Louis, IrinaHealey, AdamStacey, DavidHealey, GarethStacey, Michael C.Heath, P.R.Stachowiak, Michal K.Heberden, ChristineStachowicz, AnetaHecht, Michael H.Stachowicz-Stencel, TeresaHedden, PeterStack, GaryHeddleston, JohnStacpoole, Peter W.Hedhammar, MyStadlbauer-Köllner, VanessaHeemskerk, MirjamStádník, LuděkHeerklotz, HeikoStafford, James L.Heery, David M.Stafforst, ThorstenHeffer, MarijaStafuzza, Nedenia BonvinoHefferon, Kathleen L.Stäheli, PeterHegarty, Shane V.Stakos, DimitriosHegde, ShivanandStaley, ChristopherHegedűs, CsabaStambuk, NikolaHeger, JacquelineStamova, BoryanaHegyi, PéterStana, Anca - DanielaHeidari, BehzadStančiaková, LuciaHeidegger, IsabelStanciu, Gabriela-DumitritaHeikema, AstridStanciu, OanaHeilbronner, UrsStanculeanu, Dana LuciaHeim, FredericStanculescu, IoanaHeinbockel, ThomasStanford, StephanieHeindel, JerryStanga, SerenaHeinisch, JürgenStange, KatjaHeinrich, RalfStaniak, MariolaHeitkam, TonyStanicová, JanaHeitmar, RebekkaStaninska-Pięta, JustynaHeitz, ThierryStanislav, VosolsoběHejatko, JanStanisz, BeataHejnar, JiříStankevičius, VaidotasHejtmancik, J. FieldingStanković, DaliborHelbig, KarlaStankovic, SandaHeleniak, ZbigniewStanley, Jone A.Helfrich, MiepStanojevic, Dragana M.Helge, JørnStarek, MałgorzataHelland, ÅslaugStark, Amy L.Heller, GerwinStarkuviene, VytauteHeller, JanoschSt-Arnaud, ReneHellewell, SarahStaruschenko, AlexanderHelling, Britney A.Starz-Gaiano, MichelleHellinger, RolandStarzyńska, AnnaHellmann, NadjaStarzyński, Rafał R.Hellstrand, KristofferStasi, AlessandraHelms, BerndStasiak, AndrzejHelms, VolkhardStasiak, MagdalenaHemachandra, Reddy. PStasiak-Różańska, LidiaHémadi, MiryanaStasiolek, MariuszHemmersbach, RuthStaško, JánHemming, KarlStaszewski, OriHempel, GünterStathopoulos, ConstantinosHenderson, Robert D.Stathopoulou, KonstantinaHenderson-Jackson, EvitaStathopulos, PeterHendgen-Cotta, Ulrike B.Staudacher, AlexanderHendrik, BargelStawikowski, Maciej J.Henen, Morkos A.Stawski, RobertHeng, XiaoStebelova, KatarinaHenke, AndreasStecca, BarbaraHennerman, JuliaStecco, CarlaHennigs, Jan K.Steel, Laura F.Henningsen, AndersSteele, John WHenrich, DirkSteelman, AndrewHenriksson, Marie ArsenianStefanelli, ClaudioHenrique, RuiStefani, AmbraHenriques, Ana GabrielaStefania Longoni, SilviaHenriques, Bárbara J.Stefania, PucciarelliHenry, MarcŠtefanić, Petra PeharecHenry, Ryan A.Stefanidou, Maria E.Henry, YvesStefanon, BrunoHensel, GötzStefanovic, IvanHenstridge, ChristopherStefanowicz, JoannaHerath, H. M. Ayala S.Štefulj, JasminkaHerath, VenuraSteger, GerhardHerbst, AndreasSteger, GertrudHerczegh, PálStegner, DavidHerdean, AndreiStehle, FelixHéricourt, FrançoisStein, ChristophHering, ThomasStein, ColleenHering-Smith, KathleenStein, MatthiasHermenean, AncaSteinberg, Eliana RuthHermoso-Orzáez, Manuel JesúsSteinberg, SimonHernandez, AgustinSteinle, JenaHernández, CarmenStella, Giulia M.Hernandez, Jean-FrançoisStellavato, AntoniettaHernandez, JoaquimStelzer, FranzHernandez, PilarStender, StefanHernandez, VictorStepanenko, Aleksei A.Hernandez-Alcoceba, RubenStepanov, Gennady V.Hernandez-Alonso, PabloStepanova, EkaterinaHernández-Breijo, BorjaStephen, Michael RajeshHernández-Cifre, José GinésStephens, EdwardHernández-Lucas, I.Stephens, GaryHernández-Martínez, PatriciaStephenson, Mary CHernández-Mesa, MaykelStepien, EwaHernández-Núñez, EmanuelStepien, Karolina M.Hernández-Ochoa, Erick O.Stępień, ŁukaszHernández-Rodríguez, CésarStępnik, MaciejHerndler-Brandstetter, DietmarStepp, Mary AnnHernot, SophieSterk, Geert JanHerold-Mende, ChristelSterner, OlovHerr, Deron RaymondŠtětina, RudolfHerránz, HéctorSteven, AndreHerrera, BlancaStevens, RebeccaHerrera, FedericoStevens, Victoria L.Herrero, EnriqueStevenson, LeoHerrero, María JesúsStevenson-Lerner, HeatherHerrero-Beaumont, GabrielSteverding, DietmarHerrmann, MariettaSteward, RobertHershfinkel, MichalStewart, AdeleHerskin, Mette S.Stewart, DelishaHerskind, CarstenStewart, JamesHertel, RobertStewart, JasonHertwig, StefanStewart, MatthewHertz, Daniel L.Stewart-Ornstein, JacobHervás Pérez, Juan PabloStiburkova, BlankaHervé, TécherStieber, JulianeHervouet, EricStieger, BrunoHerzberg, MartinStiles, AshleeHesemann, PeterStilli, DonatellaHeslegrave, AmandaStillo, FrancescoHessheimer, Amelia J.Stimpfel, MartinHester, JoannaStocco, GabrieleHetényi, CsabaStocker, RetoHetland, GeirStockmann, ChristianHeuer, HolgerStockmeier, Craig A.Hewitt, Sylvia C.Stoian, DanaHey, JeremiasStoiber, DagmarHianik, TiborStojak, MartaHibi, MasahikoStojanović, SanjaHiblot, JulienStojiljkovic, MajaHibrand-St.Oyant, LaurenceStoker, Andrew WilliamHicks, AndrewStolfi, CarmineHiebert, PaulStolp, HelenHieda, MikiStolz, AndreasHigashida, KazuhikoStone, Kari L.Higashijima, Shin-ichiStopka, TomášHiggs, DavidŠtorchová, HelenaHigo, ShimpeiStorchova, ZuzanaHikima, Jun-ichiStorici, FrancescaHildreth, BlakeStorici, PaolaHildreth, EasonStorjord, ElinHildreth, SherryStorlazzi, AuroraHileman, LenaStorling, JoachimHilgemann, Donald WilliamStörmann, SylvèreHilger, ChristianeStornaiuolo, MarianoHill, GrantStossi, FabioHill, MartinStott, KatherineHilliard, Tyvette S.Stotz, Henrik U.Hilliou, FrederiqueStout, Michael J.Hills, RonaldStout, RandyHilty, MarkusStove, Christophe P.Himoto, TakashiStowell, KathrynHindra, HindraStoyanova, AlbenaHinds, Terry D.Stoychev, GeorgiHines, Dustin J.Stradal, TheresiaHines, Justin K.Stragliotto, GiuseppeHingorani, RittuStraiker, AlexHinkula, JormaStrankowska, JustynaHinman, SamuelStrappe, PadraigHinssen, HorstStrasser, RichardHinzpeter, AlexandreStraten, Per ThorHioe, Catarina E.Strati, AretiHippe, BeritStrati, FrancescoHippo, YoshitakaStratmann, BerndHirabayashi, TetsuyaStratton, Jo AnneHiraga, ToruStratton, Margaret M.Hirai, NobumitsuStrauss, JohannesHirano, KatsuyaStrauss, LauraHirano, MinakoStrauss, SarahHirasaka, KatsuyaStravopodis, Dimitrios J.Hirasawa, NoriyasuStreckfuß-Bömeke, KatrinHirata, TetsuyaStreet, Maria ElisabethHirata, YokoStrekalova, TatyanaHirata-Tsuchiya, ShizuStrelnikov, Vladimir V.Hirel, BertrandStreubel, JanaHiroaki, HidekazuStrianese, MariaHirohata, SatoshiStrich, RandyHiroki, ToyodaStrick, ReinerHirono, KeiichiStrickland, JasonHirsch, PierreStrickley, RobertHirst, WarrenStrippoli, RaffaeleHiruma, KeiStrle, DragoHisaoka-Nakashima, KazueStrnad, PavelHitachi, KeisukeStrnadel, JanHjelmen, Carl E.Stroffekova, KatarinaHlaváč, ViktorStrongin, RobertHlozek, TomasStrudwick, XantheHluska, TomášStrugała, PaulinaHnia, KarimStrungaru, Stefan-AdrianHnilicka, FrantisekStruszczyk, Marcin HenrykHo, Cheng-HsunStrzalkowski, NickHo, ChengyingStrzelecka-Kiliszek, AgnieszkaHo, Chia-cheStrzemiecka, BeataHo, Eric S.Stuart, JeffreyHo, Han KiatStuckey, RuthHo, Jar-YiStudzinski, George P.Ho, Shih-ChingStuhlmann, HeidiHo, ThanhStührwohldt, NilsHo, Tsung-ChuanStulberg, MichaelHo, VincentStupka, NicoleHoang, Nam V.Stur, ElaineHoang, Ngoc HaŠturala, JiříHoashi, ToshihikoSturiale, Carmelo LucioHochstrasser, MarkSturm, Brigitte N.Höckner, MartinaSturm, Eva MariaHodges, LynetteSturm, HeinzHodgkinson, JamesStushnoff, CecilHodgkinson, TomStylli, Stanley S.Hodny, ZdenekSu, Chia-HaoHoebeke, JohanSu, Chinh Tran-ToHoefer, ImoSu, DanHoefsloot, E.H.Su, Dong-MingHoell, Jessica I.Su, SiyuanHoffman, Brad G.Su, TaoHoffman, Charles S.Su, XinmingHoffman, Dax AaronSu, YichiHoffman, NolanSu, YongHoffmann, Klaus H.Su, Yu-KaiHoffmann, Michele J.Subbannayya, YashwanthHogan, NatachaSubbian, SelvakumarHogenEsch, HarmSubburaju, SivanHohaus, StefanSubhi, YousifHohenegger, MartinSubramani Paranthaman, BalasubramaniHohenester, Simon D.Subramaniam, NathanHohensinner, PhilippSubramaniam, SrinivasaHohmann, Miriam S. N.Subramanian, ArulHojan, KatarzynaSubramanian, SowmyalakshmiHojjat-Farsangi, MohammadSubramanyam, SubhashreeHojo, HironoriSubrata, SabuiHokugo, AkishigeSuciu, Bogdan AndreiHolak, Tad A.Sud’ina, Galina F.Holalu, Srinidhi VSuda, KenichiHolaska, JamesSudano, Mateus J.Hołda, Mateusz KrystianSudhan, DhivyaHolec, JosefSudheeran, Pradeep KumarHolecki, MichałSuehiro, EiichiHolendova, BlankaSuen, Der-FenHolguin, NilssonSuenaga, HikaruHolien, JessicaSuetsugu, NoriyukiHolland, HeidrunSuga, HirotakaHollenbach, MarcusSugawara, TatsuyaHoller, EggehardSugaya, MakotoHolliday, L. ShannonSugier, PiotrHöllig, AnkeSugii, HidekiHollomon, Mario G.Sugimoto, MasahiroHolloway, JohnSugimura, HaruhikoHolm, Jesper GrønlundSugisaki, KenjiHolme, Jørn AndreasSugishima, MasakazuHolmes, Andrew PSugiyama, MunetakaHolmes, NeilSugiyama, TetsuyaHolmøy, TrygveSuh, Dae-yeonHolnthoner, WolfgangSuh, HyunsukHolsinger, DamainSuhm, Martin A.Hölter, SabineSuhr, FrankHöltke, CarstenSukawa, YasutakaHolynska, MalgorzataSukharev, SergeiHoma, JoannaSukhov, VladimirHomblé, FabriceSukhovershin, RomanHomma, Miwako KatoSukocheva, OlgaHomma, TakujiroSukumaran, SunithaHomma, TetsuyaŠulc, PetrHomman-Ludiye, JihaneSulek, AnnaHomma-Takeda, ShinoSulentic, CourtneyHomocianu, MihaelaSuliman, Hagir BHomolak, JanSullivan, ConHonda, KazuhoSullivan, DavidHonda, YoshitomoSullivan, M. L.Hong, Chang PyoSumara, GrzegorzHong, Chwan-YangSumi, ShoichiroHong, Hyun SookSumida, YoshioHong, Jeong HeeSumiyshi, TomikiHong, Ji HyungSummavielle, TeresaHong, Ka L.Summers, Katie LynnHong, Su-HyungSummers, MatthewHong, XupengSun, BoHong, Yeong HoSun, ChengwenHong, Yi-RenSun, ChongkuiHonke, KoichiSun, DeqiangHonorio-França, AdenildaSun, FengjieHonrao, ChandrashekharSun, GongqinHonvo, GermainSun, GuohuiHooda, JagmohanSun, HaiyanHoogendijk, Mark GSun, JunjiangHoogenkamp, MaartenSun, MujunHook, Lauren M.Sun, QianHook, VivianSun, RamonHooykaas, PaulSun, Tian-HuHopp, RussellSun, WenfeiHorai, YoshiroSun, WenyueHõrak, RitaSun, WujinHorakova, JanaSun, XiaofeiHorchani, HabibSun, XiaomingHorel, ÁgotaSun, XuepengHorgan, FinbarrSun, YeHori, HiroyukiSun, ZhixiongHori, KiyosumiSunami, YoshiakiHorike, Shin-ichiSundar, GangadharaHorký, PavelSundar, Krishna M.Hörmann, GregorSundaram, Sivaraj MohanaHorn, AnselmSundaramoorthy, PasupathiHornig, NadineSundaresan, AlameluHornung, BastianSundvik, MariaHorobin, Richard W.Suñer, Damian HeineHorovistiz, AnaSung, Jong HyukHorovitz, AmnonSung, Pil SooHorowitz, John D.Sung, SibumHorowitz, MiaSung, Tzu-ChengHorsburgh, AnnSung, Wen-WeiHorsky, JiřiSungthong, RungrochHorstkorte, RüdigerSun-Hee, CheongHorstmann, Martin A.Supattapone, SurachaiHortells García, LuisSupuran, ClaudiuHortobagyi, TiborŠuput, DušanHortobágyi, TiborSur, SubhayanHorton, Edward RoySurana, PriyankaHorváth, ÁdámSurapaneni, Krishna MohanHorváti, KataSurapathrudu, KanakalaHosaka, KayokoSurazynski, ArkadiuszHoshino, DaisukeSurber, ChristianHoshino, TamotsuSurendran, KameswaranHosmillo, MyraSurmacz, EvaHosório, HugoSurmenev, RomanHosoya, AkihiroSuryavanshi, SantoshHossain, FokhrulSuryawanshi, VasantikaHossain, Md JakirSusi, PetriHosseinkhani, HosseinŠušor, AndrejHosseinpour, SepantaSussman, Mark A.Hosui, AtsushiSustkova-Fiserova, MagdalenaHosur, VishnuSustr, MarekHotta, YoshihiroSutter, ThomasHou, Chih-YaoSutton, GrangerHou, JinjunSuwan, KeittisakHou, JueSuzuki, AussieHou, ShaopingSuzuki, HideoHouben, RolandSuzuki, HiroetsuHough, KennethSuzuki, KeikoHoujou, HirohikoSuzuki, MaikoHouse, ImranSuzuki, MasaakiHoushmand, MohammadSuzuki, MasakoHouston, FionaSuzuki, Masataka G.Howard, Erin W.Suzuki, MotofumiHowe, Hwee SiewSuzuki, NobuhiroHowell, PhilSuzuki, NorioHowell, StephenSuzuki, ToruHow-Kit, AlexandreSuzuki, ToshikazuHox, ValérieSuzuki, Yuichiro J.Hrabal, RichardŠvandová, Eva BudisovaHrdy, JiriŠvastová, EliškaHrelia, PatriziaSven, GläskerHrenar, TomicaSvensson, Maria K.Hribal, MartaSvetlov, MaksimHrstka, RomanSvingen, TerjeHsaio, MichaelSviridov, DenisHsia, Shih-MinSvirshchevskaya, ElenaHsia, VictorSvoboda, JanHsiao, I-LunSvobodová, JanaHsiao, Yu-ShengSwami, UmangHsieh, Chien-MingSwamy, GaneshHsieh, Ming KunSwanson, Kenneth D.Hsieh, Ming-FaSwartz, Steven ZacharyHsieh, Ming-JuSwartz, Talia H.Hsieh, Pei-LingSwarup, RanjanHsieh, Tusty-JuanSwayne, Leigh-AnneHsieh, Wen-TsongSweadner, Kathleen J.Hsu, Bang-geeSweetman, MartinHsu, Chung-YuanSwelum, Ayman AHsu, Fang RongSwenson, Nathan G.Hsu, FeitingŚwiątek, PiotrHsu, Fei-TingŚwiątkiewicz, IwonaHsu, Fu-YinSwiatkowska, AgataHsu, Kuo-ShengSwida-Barteczka, AleksandraHsu, Li-SungSwiecilo, AgataHsu, Ming-HuaSwiercz, AleksandraHsu, Ping ChihŚwierczek, JanHsu, Ren-JunSwierczynski, JulianHsu, Shang-Te DannySwieringa, FraukeHsu, Sung-PoŚwierniak, AndrzejHsu, ToddŚwiętnicki, WiesławHsu, Tsung-ISyahputra, KhairulHsu, Tuan-TiSybilski, Adam J.Hsu, Yi-ChiungSyed, FarooqHsueh, Yi-HuangSyed, NaeemHu, ChanglongSyed, NeloferHu, Chaur-JongSyeda, AishaHu, Dong GuiSyed-Ab-Rahman, Sharifah FarhanaHu, GuokuSyranidou, EvdokiaHu, GuoliSyrjanen, StinaHu, JianSyrris, PetrosHu, JunhuiSytykiewicz, HubertHu, LiangSzabo, AndrasHu, MinSzabó, AndrásHu, Po-ShengSzabo, AronHu, QiwenSzabó, KornéliaHu, TaoboSzabó, LászlóHu, WenweiSzabo, MilanHu, XiaoSzabó, RenátaHu, XiaoyuSzachniuk, MartaHu, YangSzacon, ElzbietaHu, YuSzade, KrzysztofHu, Yueh-ChiangSzafrańska, KatarzynaHuang, AndrewSzakiel, AnnaHuang, Chih-YangSzalai, GabriellaHuang, Chiu-jungSzaleniec, MaciejHuang, Chiung-ChengSzállási, ÁrpádHuang, FenixSzanto, MagdolnaHuang, Guan-JhongSzaryńska, MagdalenaHuang, Guan-ShiengSzászi, KatalinHuang, Han HsiangSzatkiewicz, Jin P.Huang, HuSzatmari, Erzsebet MariaHuang, HuangSzczegielniak, JadwigaHuang, Huey-ChunSzczepanek, JoannaHuang, Hui-ChunSzczepanowska, KarolinaHuang, Jenq-WenSzczesny, SpencerHuang, JierongSzebeni, Gábor JánosHuang, Kuang-TzuSzéchenyi, AleksandarHuang, Kun-LunSzedlacsek, StefanHuang, LeiSzeiffova Bacova, BarbaraHuang, LiliSzeliga, MonikaHuang, Li-TungSzéll, MártaHuang, Paul Li-HaoSzentesi, PéterHuang, QianshengSzepesi, AgnesHuang, QijinSzepesi, ÁgnesHuang, Shau-KuSzepietowska, BarbaraHuang, Shih-YiSzerdahelyi, EmőkeHuang, ShuanglongSzeto, Savio Yim TongHuang, Shu-HungSzeto, Yim-TongHuang, ShuoSzewczuk, Myron R.Huang, TimothySzewczyk, KatarzynaHuang, Tse-HungSzewczyk-Golec, KarolinaHuang, Tze-SingSzilágyi, AndrásHuang, TzetaSzkaradkiewicz, AndrzejHuang, Tzu-TingSzklarz, GrazynaHuang, Wei-ChunSzklarzewicz, JanuszHuang, WeihuaSzoboszlai, NorbertHuang, Wen-ChinSzöllősi, Attila GáborHuang, Wu-JangSzöllősi, DánielHuang, XiaohuSzondy, ZsuzsaHuang, XinSzoor, ArpadHuang, XuSzopa, AleksandraHuang, XudongSzóstek-Mioduchowska, AnnaHuang, YahuiSztanke, KrzysztofHuang, Yanhua H.Szteyn, KalinaHuang, Yan-JangSztiller-Sikorska, MalgorzataHuang, Ying-HsienSztul, ElizabethHuang, YuSzulc-Dąbrowska, LidiaHuang, Yu-AnSzuts, DavidHuang, Yu-ChuenSzweda, PiotrHuber, MichaelSzwengiel, ArturHuber, ReneSzychlinska, Marta AnnaHuber, VictorSzyfter, KrzysztofHublitz, PhilipSzymańki, PawełHübner, MaxSzymańska, GrażynaHuchzermeyer, BernhardSzymańska, Katarzyna PatrycjaHuck, OlivierSzymborski, TomaszHuda, NazmulTaank, VikasHudlikar, RasikaTaanman, Jan-WillemHudson, Andre O.Tabares, LuciaHudson, NatalieTabary, OlivierHuerta-Angeles, GloriaTabęcka-Łonczyńska, AnnaHuerta-Yépez, SaraTabler, TomHueso, MiguelTabuchi, MasashiHufbauer, MartinTacconelli, StefaniaHughes, ColanTachibana, ShinjiroHughes, WilliamTachibanaki, ShujiHugo, Eric R.Tacke, FrankHugo, LeonTada, RuiHugueney, PhilippeTada, SeiichiHuh, Jin HoeTada, YayoiHuleihel, MahmoudTadepalli, SirimuvvaHull, J. JoeTadié, Jean-MarcHull, J.JoeTadini Buoninsegni, FrancescoHulleman, JohnTadyszak, KrzysztofHulme, JohnTae-Jin, KimHumayun, Mark S.Tafani, MarcoHumbert, MagaliTagad, HarichandraHumbert, Patrick O.Tagang, AluwongHumbolt, PatriceTaghibiglou, ChangizHummel, MaryTagliaferri, PierosandroHunakova, LubaTagliatesta, PietroHund, MartinTagliavia, MarcelloHundley, Heather A.Taglietti, Angelo MariaHung, AndrewTago, KenjiHung, KennethTaguchi, YoshihiroHung, Shao-WenTaha, Muhamed KheirHung, Shih-YaTaha, MuhammadHung, Tai-FengTahara, HidetoshiHung, WeitingTahir, AmmarHung, Yao-ChingTahrioui, AliHung, Yu-JuTaibi, KhaledHunt, TamerahTailhades, JulienHunter, Charles T.Taillandier, DanielHuntington, Nicholas DavidTaishi, NakamuraHuntosova, VeronikaTajeddine, NicolasHupp, SabrinaTaji, TeruakiHuppé-Gourgues, FrédéricTajiri, KazutoHuppert, StaceyTajiri, NaokiHuppertz, BertholdTajuelo, JavierHur, YoonkangTak, EunyoungHura, TomaszTakač, IztokHurwitz, StephanieTakács-Vellai, KrisztinaHusnjak, KoraljkaTakada, AkihikoHussain, Ahmad FawziTakada, TappeiHussain, MubsharTakagi, JunichiHussain, Muhammad SajidTakagi, MasatoshiHussain, Rumana N.Takahashi, AkihisaHussain, SamanTakahashi, AkikoHussaini, HaizalTakahashi, DaisukeHussein, MaythamTakahashi, HidekazuHutchins, Andrew PTakahashi, JunkoHutchinson, John NTakahashi, KenHutmacher, DietmarTakahashi, Melissa KHüttemann, MaikTakahashi, MisaHutter, MichaelTakahashi, ShinyaHutterer, Georg C.Takahashi, TakuHuuskes, Brooke MareeTakahashi, ToshioHuuskonen, Mikko TuomasTakahashi, YutakaHuvenne, WouterTakai, AtsushiHuxford, TomTakai, KazuyukiHwa, JohnTakai, ShinjiHwang, Chang-ilTakai, YasushiHwang, ChangmoTakam Kamga, PaulHwang, Ho SikTakami, AkiyoshiHwang, In KooTakano, HudsonHwang, Jae SungTakano, JunpeiHwang, JeeyeonTakano, KatsuraHwang, JustinTakano, TomomiHwang, Pung PungTakasawa, ShinHwang, WonmukTakashi, IshidaHwang, YongsungTakashi, YuichiHwang-Verslues, Wendy W.Takata, KeiichiHwu, Wuh-LiangTakata, MinoruHyatt, HaydenTakatani-Nakase, TomokaHyeonso, JiTakaya, JunjiHyer, SteveTakaya, TomohideHyink, Deborah PinsonTakayama, KazuoHyun, Young-MinTakayama, KenichiHyun-jung, ParkTakebayashi, ShigeoIaccarino, GuidoTakeda, MakioIaccarino, IngramTakeda, MamoruIaccino, EnricoTakeda, YoshiyuIacoangeli, AlfredoTakemori, HiroshiIacono, DiegoTakemoto, ShinjiIadarola, PaoloTakenaka, MizukiIan, WalshTakenaka, NobuyukiIanchis, RalucaTakeshita, FumitakaIannazzo, DanielaTakeshita, KazutakaIannelli, M. A.Takeuchi, HideyukiIannolo, GioacchinTakeuchi, JunIaria, CarmeloTakeuchi, KohIasevoli, FeliceTakeuchi, TomoharuIatsenko, IgorTakeuchi, YasuhiroIbarra Molero, BeatrizTakhter, Ramandeep S.Iben, SebastianTakino, JunichiIbrahim, AhmedTakito, JiroIbrahim, Mohab M.Takizawa, ShinobuIbrahim, Mohamed AliTakuma, KazuhiroIbrahim, Sulaiman SadiTala, Joana A.Ichihashi, MasamitsuTalaat, Iman M.Ichikawa, TakahiroTalagas, MatthieuIconomidou, VassilikiTalavera, KarelIdda, Maria LauraTalbert, ErinIde, HisamitsuTalesa, Vincenzo NicolaIdili, AndreaTalhada, DanielaIdo, YasuoTallini, Luciana R.Idogawa, MasashiTalora, ClaudioIeni, AntonioTalukdar, Prabhat K.Ientile, RiccardoTalukder, PoulamiIeraci, AlessandroTaly, AntoineIerardi, EnzoTam, Roger Y.Ifergan, IgalTamagawa-Mineoka, RisaIgamberdiev, Abir U.Tamagnone, LucaIgartua, ErnestoTamaki, TetsuroIgbaria, AeidTamasi, GabriellaIgea, AnaTamayo, EstherIglesias Fernández, RaquelTamba, Bogdan IonelIglesias, Maria Del RosarioTambe, MitaliIglesias-Linares, AlejandroTambuwala, MurtazaIglesias-Osma, Maria CarmenTame, JeremyIgnatieva, Elena V.Tamkovich, SvetlanaIgnatov, AlexanderTampa, MirceaIgnatowska-Jankowska, Bogna M.Tamura, KojiIgnjatovic, NenadTamura, YasuhisaIgual, MartaTan, Aaron C.Ihnatko, RobertTan, Bee KIhrie, RebeccaTan, Bee KangIida, KeiTan, DanielIijima, KazutoshiTan, Du-XianIizuka, KatsumiTan, Howe-SiangIkeda, MasahiroTan, Joanne T.M.Ikeda, MasatoTan, KarenIkeda, TohruTan, LongzhiIkeh, MélanieTan, Nguan SoonIkehara, KenjiTan, Shyh HanIkeno, ShinyaTan, Sih MinIkura, TeikichiTan, YuqiIlag, Leodevico L.Tanabe, HideyukiIlagan, Ma. Xenia G.Tanabe, KatsuyukiIlari, AndreaTanabe, KenjiIlchibaeva, TatianaTanabe, ShihoriIldefonso, Cristhian J.Tanaka, AtsushiIlia, GheorgheTanaka, Hiroyoshi Y.Ilic, NebojsaTanaka, HiroyukiIlles, PeterTanaka, HisashiIllien-Jünger, SvenjaTanaka, IchidaiIm, Chang-NimTanaka, KiwamuIm, Seung-SoonTanaka, MasaruIm, SunTanaka, MiyakoImai, HirooTanaka, NaoroImai, JunTanaka, NobuhiroImai, YoichiTanaka, NobuyukiImamura, MasahiroTanaka, SatoshiImamura, SousukeTanaka, ShigeyukiImani, MahdiTanaka, TakujiImbeault, MichaelTanaka, ToshiyaImbert, VéroniqueTanaka, YasukoImbimbo, BrunoTanaka, YukaImbriano, CarolTancini, BrunellaImig, JohnTanda, Enrica TeresaImparato, GiorgiaTanda, NaokoImperatore, ConcettaTang, Chih-HsinImran, Qari MuhammadTang, DamuInagaki, ShinobuTang, FengruInbraj, StephenTang, Ho LamIncarnato, DannyTang, Ho ManInchingolo, FrancescoTang, JingIncitti, TaniaTang, JingjingIncoronato, MariarosariaTang, JonathanIndini, AliceTang, KaiIndovina, PaolaTang, Ming-JerInestrosa, Nibaldo C.Tang, XiaoliInfante, ArantzaTang, XiaoyunInfante, MarcoTang, YaoInfante, PaolaTang, ZhihuaIngendoh-Tsakmakidis, AlexandraTangadanchu, Vijai Kumar ReddyIngham, Aaron B.Tani, AkioInglada-Pérez, LucíaTani, EleniIngram-Smith, CherylTanic, NikolaIniesta Cuerda, MaríaTanida, TakashiInnchi, LeeTaniguchi, HiroakiInnocenti, MetelloTanimizu, NaokiInokuchi, Jin-IchiTanini, DamianoInoue, HaruhikoTanini, MarcoInoue, HaruhisaTanino, KarenInoue, KeiichiTaniuchi, IchiroInoue, TatsuyaTankiewicz-Kwedlo, AnnaInoue, TsuyoshiTanner, NathanInoue, YusukeTannock, Lisa R.Inselman, AmyTanonaka, KouichiInserte, JavierTanphaichitr, NongnujInubushi, ToshihiroTansi, Felista L.Inuzuka, HiroyukiTanti, Goutam KumarInyushin, Mikhail Y.Tantos, ÁgnesInzana, Thomas J.Tao, FengIoan Voicu, StefanTao, JunyanIoannis, GiannakouTao-Cheng, Jung-HwaIolascon, GiovanniTaoka, YousukeIommelli, FrancescaTapia, Jose A.Ionescu, Daniela C.Tapio, SoileIonita, GabrielaTappia, ParamjitIonita, PetreTar, KrisztinaIop, LauraTarakhovskaya, ElenaIordache, FlorinTarallo, AntoniettaIorga, AndreaTarallo, RobertaIorga, B. I.Tarang, ShikhaIorio, EgidioTaranta-Janusz, KatarzynaIorio, Ronald M.Tarantino, GiovanniIosifidis, MichaelTarantino, UmbertoIotti, StefanoTarasova, Ol’Ga A.Iourov, Ivan Y.Tarcea, MonicaIovane, AndreTarn, Woan-YuhIovene, MarinaTarone, AaronIpseiz, NatachaTarozzi, AndreaIqbal, OmerTarsitano, AchilleIrace, CarloTasca, CarlaIram, SurtajTash, Joseph S.Irato, PaolaTashima, KimihitoIrie, TetsumiTashima, ToshihikoIrimiciuc, Stefan-AndreiTashiro, EtsuIrving, TomTashiro, MitsuruIrzyniec, TomaszTasso, BrunoIsabel Veiga, MariaTasso, RobertaIsabey, DanielTate, RothwelleIsacson, OleTate, Shin-IchiIsakova-Sivak, IrinaTateishi, KensukeIsani, GloriaTatischeff, IrèneIsayama, HiroyukiTattikota, Sudhir GopalIsemura, MamoruTattini, MassimilianoISHIAI, MasamichiTatu, Alin LaurenţiuIshibashi, KenichiTaub, MaryIshibashi, NobuyukiTaube, JosephIshibashi, OsamuTauber, EranIshibazawa, AkihiroTauber, JamesIshida, MariTaubert, HelgeIshidate, TakaoTauriello, DanieleIshigami, TomoakiTautermann, Christofer SIshihara, KeiichiTavakoli, JavadIshii, HidekiTavares, AnthonyIshii, MasakazuTavares, LucianaIshii, TetsuroTaves, MatthewIshikane, ShinTavío, María M.Ishikawa, EiichiTavolari, SimonaIshikawa, KiyotakeTayebati, Seyed KhosrowIshikawa, RyujiTayebi, NahidIshimoto, TakahiroTayeh, NadimIshimoto, TakatsuguTaylan, FulyaIshitsuka, YoichiTaylor, AlisonIshitsuka, YosukeTaylor, AllenIshizaki, AzumiTaylor, Erika A.Ishizaki, TakumaTaylor, IsaiahIshizaki, YasukiTaylor, KeithIshizuka, TakumiTaylor, Mark S.Isidoro, CiroTaylor, MatthewIslam, JahidulTazawa, HiroshiIslam, Md MohaimenulTchertanov, LubaIslam, Md SorifulTchetina, Elena V.Islam, Md TohidulTchoghandjian-Auphan, AurélieIslam, Md. TabibulTe Velthuis, AartjanIslam, Mohammad MazharulTeale, WilliamIslam, Mohammad MirazulTedeschi, TulliaIslam, Salim TimoTee, JamesIslam, SalmanTeerds, Katja J.Islam, SorifulTegeder, IrmgardIsmaiel, AdnanTeijeira, ÁlvaroIsmail Ali, HamedTeisseyre, AndrzejIsmail, Mariam N.Teixeira, RitaIsozaki, TakeoTejedor, Rosa M.Itabashi, EtsukoTelegina, Darya V.Itakura, YokoTéletchéa, StéphaneItatani, YoshiroTella, Sri HarshaIto, FumitakeTellez-Isaias, GuillermoIto, KeiichiTellez-Lance, AngelaIto, ShinsakuTellier, MichaelIto, TakamichiTembrock, Luke R.Ito, YasuhiroTemel, AslihanIto, YukakoTempesta, MariaItoh, Jun-IchiTempfer, HerbertItoh, YoshifumiTenbrock, KlausItou, JunjiTenenbaum, LilianeIuga, DinuTeng, Ba-BieIuliano, RodolfoTeng, PengIvančić Šantek, MirelaTeng, Yang D.Ivanda, MileTeng, YongIvanov, AlexanderTenkorang, MavisIvanov, AndreiTenkumo, TaichiIvanov, MilanTennstedt, PierreIvanov, Stanimir S.Tenore, GianIvanov, YuriTentler, DmitriIvanov, Yuri D.Teo, JeremyIvanova, AnelaTeodorescu, MihaelaIvanova, Donika G.Teotia, Arun KumarIvanova, EkaterinaTeotia, SachinIvanova, MargaritaTéoulé, EvelyneIvanova, SvetlanaTer Kuile, BennoIvics, ZoltánTeranishi, KatsunoriIvy, DunbarTeras, RihoIwai, AtsushiTerasaki, MichishigeIwaï, HideoTerayama, HayatoIwaizumi, Masakazu GTerenin, Ilya M.Iwamoto, MasahiroTerent’ev, AlexeiIwasaki, KentaTerentes-Printzios, DimitriosIwasaki, TakashiTerentyev, Vasily V.Iwata, JunichiTerenzi, AlessioIwata, TakanoriTerlikowska, KatarzynaIwata, TomoyukiTerracciano, DanielaIweala, OnyinyeTerracciano, RosaIyer, ChitraTerradas, MarionaIyer, Deepak NarayananTerret, Marie-EmilieIyer, JananiTerrinoni, AlessandroIyer, S.P.Teruel-Montoya, RaúlIzaguirre, GonzaloTeruyama, RyoichiIzasa, HisashiTerzaghi, WilliamIzatulina, Alina R.Terzaghi, William BryanIzawa, HironoriTerzi, ValeriaIzawa, Kazuhiro P.Terzibasi, Tozzini EvaIzawa, TakeshiTerzić, JanošIzawa, TetsuyaTerzuoli, LuciaIzdebska, MagdalenaTesarik, JanIzquierdo-Rico, Maria JoséTeschke, RolfIzquierdo-Useros, NuriaTesfaye, DawitIzumi, YuichiroTeshima, TakanoriIżykowska, KatarzynaTesic, ZivoslavIzzi, ValerioTessarz, PeterIzzo, LuanaTessem, Jeffery S.Izzo, PaolaTessema, Gezahegn GirmaJaaro-Peled, HannaTesta, UgoJabbari, HosnaTestolin, RaffaeleJablonowski, ZbigniewTétard-Jones, CatherineJablonska, EwaTetens, JensJacevic, VesnaTeti, GabriellaJachetti, ElenaTettamanti, GianlucaJackson, SimonTetz, GeorgeJacob, ChristopheTevosian, Sergei G.Jacobs, Elizabeth RTexido Bartes, RobertJacobs, HuguesThackray, AlanaJacobs, RogerThaker, Youg RajJacobsen, MonicaThakur, Basant KumarJacobson, KennethThakur, GarimaJacobs-Sera, DeborahThakur, MathewJacquin, EliseThakur, ShilpaJadeja, RavirajsinhThalamuthu, AnbupalamJadhav, ShyamalagauriThalhammer, AnjaJadidi, AmirThalhammer, TheresiaJadiya, PoojaThalheim, TorstenJae Wook, YangThalmann, MatthiasJaffar, ZeinaThammina, Chandra SekharJaffe, EileenThan, Nandor GaborJagannatha, RaoThan, Tin A.Jaggi, MeenaThangavel, ChellappagounderJaggupilli, AppalarajuThanigaimani, ShivshankarJagiello, KarolinaThankachan, StephenJagiello-Gruszfeld, AgnieszkaThannickal, Thomas C.Jagla, KrzysztofTharkeshwar Raghunath, Arun KumarJagodzinski, FilipThavasyappan, ThambiJagus, RosemaryThe, ErlindaJaguva Vasudevan, Ananda AyyappanTheile, DirkJähn, KatharinaTheiss, CarstenJahn, OlafThen, CorneliaJahng, Wan JinTherese, RiedemannJahnke, GizellaThéron, LaëtitiaJaimes, Edgar A.Theus, Michelle HedrickJain, EraThevenod, FrankJain, MitenThewke, Douglas P.Jain, VaibhavThiel, Cora S.Jaiswal, Jyoti K.Thiel, GeraldJaitovich, ArielThieme, RenéJakaria, Md.Thierry, HauetJakas, AndrejaThies, StephanJakiel, GrzegorzThirunavukkarasu, ShyamalaJakiela, BogdanThoene, JessJakimovski, DejanThomale, JürgenJakobsen, MogensThomas, BobbyJakopovic, MarkoThomas, Carole D.Jakovlevic, DraganaThomas, ClémentJakubík, JanThomas, DebbyJakubowska, MonikaThomas, JulieJakubowski, HieronimThomas, NoelJalali, Beenu MozaThomas, ShaneJalan-Sakrikar, NidhiThomas, ThresiaJalil, Abbe Maleyki MhdThomas, WayneJalkanen, SirpaThompson, AlexanderJambrina, Pablo G.Thompson, JoyceJames, JohnThompson, LaDoraJames, Nicholas G.Thompson, PeterJames, Rob S.Thomson, David M.James, VictoriaThongprayoon, CharatJamesdaniel, SamsonThornton, SophieJameson, DavidThorpe, StephenJamin, NadègeThounaojam, Menaka ChanuJamnik, AndrejThrelkeld, StevenJampilek, JosefThrivikraman, GreeshmaJan Laskowski, ŁukaszThuillier, RaphaëlJana, BimalThulstrup, Peter W.Jana, SayantanThurotte, AdrienJanakiraman, HarinarayananThurston, John H.Janas, AdamThussagunpanit, JutipornJanas, DawidThyele, GeoffreyJancsó, GáborTian, FurongJanczar, SzymonTian, Kun VivianaJanczarek, MonikaTian, LiJandl, KatharinaTian, ShiliangJaneczko, MonikaTian, Ya-ChungJaneczko, TomaszTiao, Mao-MengJanek, TomaszTiapko, OleksandraJang, AeraTiberini, AntonioJang, Byoung KukTicconi, CarloJang, ByungkiTidow, HenningJang, HyoncholTien, Lu-TaiJang, JichanTiera, MarcioJang, JinahTietze, AlesiaJang, SeonghoeTiganescu, AnaJanga, Srikanth ReddyTigyi, GaborJangampalli Adi, PradeepkiranTijing, LeonardJaniak, AgnieszkaTikellis, ChristosJanik, KatrinTikhanovich, IrinaJanik, StefanTikhenko, Natalia D.Janiuk, HIzabelaTikhomirova, IrinaJanjetovic, ZoricaTikhonova, IrinaJankovic, BrankicaTikkanen, RitvaJankowicz-Cieslak, Joanna BeataTilbrook, AlanJanousek, BohuslavTillekeratne, L.m. VirangaJans, Judith J.M.Tilli, Tatiana MJansone, BaibaTilocca, BrunoJansova, DenisaTimashev, PeterJantzie, Lauren L.Timchenko, TatianaJanuchowski, RadosławTimko, MichaelJanusz, GrzegorzTimmerman, KyleJanuszko, AdamTimmins, JoannaJao, Tzu-MingTimofeev, Edward N.Jaquet, KorneliaTimonen, JuriJaradat, NidalTimoshenko, Alexander V.Jaramillo-Flores, MariaTimoshevskiy, Vladimir A.Jara-Palomares, LuisTimpani, CaraJarcuska, PeterTimpano, MassimilianoJardin, IsaacTimperio, Anna MariaJarocka-Cyrta, ElzbietaTimsit, YouriJarome, TimothyTing, Richard C. H.Jarończyk, MałgorzataTinganelli, WalterJaroszewicz, JerzyTinoco, Arthur D.Jaroszuk - Ściseł, JolantaTintera, JaroslavJarret, Robert L.Tintino, SauloJarstfer, Michael BruceTiperciuc, BrindusaJarugula, SridharTirado-corbala, RebeccaJärvinen, Tero A. H. Tiranti, ValeriaJarzebski, MaciejTirino, VirginiaJasińska-Stroschein, MagdalenaTirrò, ElenaJasoliya, MittalTischkau, Shelley A.Jastrzebska, BeataTisi, RenataJászai, JózsefTisoncik-Go, JenniferJat, Parmjit S.Tissier, Paul LeJaumot, JoaquimTiteca-Beauport, DimitriJavadov, SabzaliTittarelli, AndrésJavelle, EmilieTitus, MarkJawhar, MohamadTitz, BjoernJaworek, Andrzej KazimierzTiwari, Rakesh K.Jaworska, DagmaraTiwari, ShashikantJay, StevenTjernberg, IvarJayant, RahulToan, SamJayanthi, SankarTobita, MorikuniJayaraman, ArulTocmo, RestitutoJayawardena, DulariToczylowska-Maminska, RenataJazirehi, Ali R.Toda, TomohisaJB Van Den Hoff, MauriceTodaka, DaisukeJean, ChristineTodd, LaurenJean, LudovicTodd, Nevins W.Jean-François, FrantzTodi, SokolJeanne, MathieuTodorov, Svetoslav DimitrovJean-Quartier, ClaireTogashi, YuichiJedlovszky-Hajdu, AngelaTogawa, AtsushiJedryczka, MalgorzataToghraee, DavoodJee, Hyeon-GunTognini, PaolaJee, ShiouhwaTognolini, MassimilianoJeffree, Rosalind LindyTogo, FumiharuJekabsone, AisteTohidnezad, MersedehJelokhani-Niaraki, MasoudToietta, GabrieleJeltsch, AlbertToillon, Robert-AlainJembrek, Maja JazvinšćakTojima, IchiroJena, Prasant KumarTokar, ErikJendelová, PavlaTokarek, TomaszJenei-Lanzl, ZsuzsaTokars, Valerie L.Jeng, Chung-JiuanTokarz, PaulinaJeng, Jiiang-HueiTokas, TheodorosJenkins, Brendan J.Tokmakov, Alexander A.Jenkins, Jill A.Tokuda, ShinsakuJenkins, Samir V.Tokumaru, YoshihisaJensen, BenjaminTokumoto, ToshinobuJensen, Eric D.Tokunaga, FuminoriJensen, JustTokuraku, KiyotakaJensen, KimmoTolba, René HanyJensen, Lars HenrikTolga, KarsiliJensen, Rebekka VibjergToljan, KarloJensen-Jarolim, ErikaTolomelli, AlessandraJeon, SoheeTolwinski, NicholasJeong, Byung-HoonToma, ClaudioJeong, Dong-HoonToma-Fukai, SachikoJeong, DongtakTomasetti, CarmineJeong, Gil-SaengTomasetti, MarcoJeong, Ho-ChangTomasetto, CatherineJeong, Hyun YoungTomašovičová, NatáliaJeong, Keun-YeongTomassetti, AntonellaJeong, Rae-DongTomas-Zapico, CristinaJeong, SekyooTomaszewska-Zaremba, DorotaJeong, Seon-YongTomaszkiewicz, MartaJeong, SeriTomatsu, ShunjiJerca, Florica AdrianaTomaz, Cândida TeixeiraJerebtsova, MarinaTomei, AliceJeremic, Aleksandar M.Tomescu, Mirela CleopatraJermakowicz, EdytaTominaga, KanaJerman, IgorTominaga-Wada, RumiJernberg-Wiklund, HelenaTomita, KozoJeroen T, BuijsTomita, ShunsukeJeronimo, CarmenTomiyama, ArataJerzak, Malgorzata MariaTomkin, Gerald HJerzemowska, GrażynaTomkinson, NicholasJeseta, MichalTommasino, FrancescoJesionek, MarcinTommasino, MassimoJetten, AntonTommonaro, GiuseppinaJeung, Eui-BaeToms, DerekJewell, JennaTomuleasa, CiprianJežek, PetrTona, FrancesoJeżewska-Frąckowiak, JoannaTonazzini, IlariaJha, MithileshTondi, DonatellaJha, Ramesh K.Tondo, MireiaJhanwar, ShaluTong, MancyJi, HongTong, MingmingJi, PengTong, qiangJia, JunTong, WeiJia, ShangTönges, LarsJia, WilliamTonhajzerova, IngridJia, ZheToniolo, Antonio Q.Jiang, ChengToniolo, LuanaJiang, ChengminTonolo, GiancarloJiang, Jean X.Top, JanettaJiang, JianxiongTopf, UlrikeJiang, QingfeiTopin, JeremieJiang, Shu-YeToplak, IvanJiang, WeiTordai, AttilaJiang, YongToriba, TaiyoJiang, YunToriello, FilippoJiang, ZhenqiTorigoe, KanjiroJiang, ZiwenTörn, CarinaJiantai Timothy, QiuTornillo, GiusyJibran, RubinaToro, Mario DamianoJicsinszky, LászlóToro, RobertaJikuzono, TomooTörök, KatalinJiménez-Barbero, JesusTorra, JoelJiménez-González, GemaTorralba-Cabeza, Miguel-ÁngelJiménez-Jiménez, Félix JavierTorras, JuanJiménez-Sánchez, María CarmenTorras-Ambròs, JoanJimi, EijiroTorre, MarialuisaJin Boo, JeongTorreggiani, MassimoJin, Dong-HoonTorregrosa, RubenJin, HongpingTorrens, FranciscoJin, Hye JinTorres Barcelo, ClaraJin, Jun-OTorres Sangiao, EvaJin, LiTorres, Adrian GabrielJin, TaoTorres, MagdalenaJin, TengchuanTorres-Lagares, DanielJin, XinTorres-Mendoza, DanielJin, YuefeiTorres-Ruiz, RaulJin, ZhaohuiTorres-Sanchez, SoniaJindo, KeijiTorsello, BarbaraJing, RanTorson, AlexJing, WenwenTorti, MauroJinno, YoheiToru, IchisekiJinrui, YangToruner, Gokce AltayJinwal, Umesh K.Torzewska, AgnieszkaJiráček, JiříTósaki, ÁrpádJirkovská, AnnaToscani, AnitaJo, Byung HoonToscani, DeniseJo, Yong HunToseland, Christopher P.Joachim, StephanieTostivint, HervéJoachimiak, EwaTotah, RheemJoão, SilvaToth, Andras JozsefJobin, Marie-LiseTóth, GergőJobling, MalcolmTóth, Melinda ErzsébetJobling, MichaelTóth-Molnár, EditJócsák, IldikóTóthová, ĽubomíraJoe, EunhyeToto, AngeloJohann, SonjaTotsingan, FilbertJohannesen, Katrine M.Totta, PierangelaJohansson, AndersTõugu, VelloJohansson, PegahTouriol, ChristianJohn Jervis, PeterToussaint, ArianeJohn, ŁukaszTovo, Pier-AngeloJohnen, GeorgTovoli, FrancescoJohner, AlbertTower, JohnJohnsen, Kasper BendixTowse, Clare-LouiseJohnson, Boris GabrielToyoda, MasaoJohnson, CarolineToyoda, MasashiJohnson, Colin P.Tozawa, YuzuruJohnson, DanielTozer, GillianJohnson, David G.Tőzsér, JózsefJohnson, ErikTozza, StefanoJohnson, GregoryTozzi, Maria GraziaJohnson, Lance A.Trabocchi, AndreaJohnson, NicholasTraiffort, ÉlisabethJohnson, PhilipTraina, GiovannaJohnson, RajasinghTraina-Dorge, Vicki L.Johnson, Reed F.Traini, ToninoJohnstone, DanielTraka, MariaJohnstone, ElizabethTrammell, SamuelJoles, Jaap A.Tramontano, DonatellaJolkkonen, JukkaTramontano, MarcoJolly, Mohit KumarTran, Anh NhatJones, ChrisTran, PhuJones, Corinne A.Tranbarger, Timothy JohnJones, ElenaTranebjærg, LisbethJones, Jeffrey ATrapani, DarioJones, JohnTrapani, IvanaJones, MattTrapani, ValentinaJones, MerielTrapero, IsabelJones, O.D.Trask, SydneyJones, PeterTravelli, CristinaJong, ChianjuTravlos, IliasJoo, Min WookTrcek, JanjaJoo, YuyoungTreder, JadwigaJordan, ChloeTreeck, MoritzJordan, Cynthia L.Trefely, SophieJordan, HannahTreglia, GiorgioJordan, PeterTrejtnar, FrantisekJordan, V. CraigTremblay, Jean ChristopheJorgačevski, JernejTremblay, MichelJorge, FeitoTremblay, NicolasJorge, J. C. LuisTrendelenburg, MartenJorgensen, AndreaTrentini, AlessandroJorgensen, Jens Otto LundeTrevisan, AndreaJörns, AnneTriaca, VivianaJorrin-Novo, Jesus VTriantafillidis, John KJosef, KapfhammerTribulova, NarcisJoseph, JamesTricarico, DomenicoJoseph, Justin VTrifonov, VladimirJoshi, AdityaTrigatti, BernardoJoshi, Bharat H.Trimarco, BrunoJoshi, KamalTrinchera, MarcoJoshi, Pushpa RajTrinchese, GiovannaJoshi-Navare, KasturiTrindade, AlexandreJost, DanielTrindle, CarlJost, MatthiasTripathi, AmitJosue, DelgadoTripathi, DiwakerJoubert, AnnieTripathi, Jaindra NathJoubert, Lydia-MarieTripathi, Jitendra KumarJoyce, Michael A.Tripathi, ManishJoyce, Susan A.Tripathi, ManishaJóźwicki, WojciechTripi, GabrieleJozwik, MaciejTripodi, PasqualeJu, Sang-YhunTrippel, Stephen B.Juan, Chi-changTripp-Valdez, Miguel A.Juan, Hsueh-FenTrisolino, GiovanniJuengel, Jennifer L.Triunfo, StefaniaJugessur, AjuTrivanovic, DrenkaJuhász, BélaTroedsson, Mats H.T.Juhász, RékaTrofimov, Aleksei V.Juhász, TamásTrögl, JosefJulián, EstherTrohman, RichardJulien, Christian M.Troitsky, AlexeyJulio, Bastos ArrietaTrojanowicz, BoguszJullian, NathalieTrojanowski, DamianJuluri, AbhishekTrombik, TomaszJulve, JosepTromp, Angelino T.Jumbo-Lucioni, PatriciaTrompier, DorianeJun, Chang-DukTronchere, HeleneJun, SawadaTroncoso-Ponce, AdrianJung, Alain C.Troppmair, JakobJung, Chan KwonTrotsko, NazarJung, Chol-HeeTrotta, FrancescoJung, Ho WonTrubiani, OrianaJung, HyungilTrujillo-Rodríguez, María J.Jung, InukTrullas, RamónJung, Jae HwanTruncaitė, LidijaJung, JinseiTrunk, KatharinaJung, Ki-HongTrus, IvanJung, KlausTrusca, Georgeta VioletaJung, Kwang-MookTruskey, George AJung, SaschaTrybus, Kathleen M.Jung, Se YongTryggestad, JeanieJung, Sung ChulTsaballa, AphroditeJung, Woo-JinTsai, Ang-ChenJung, Yuh-SeogTsai, Feng-ChouJungblut, Peter R.Tsai, Jaw-ShiunJunger, WolfgangTsai, Jen-NingJunka, Adam FeliksTsai, Kun-LinJurasz, Paul K.Tsai, Kun-LingJurczyk, B.Tsai, Ming-TzuJurenas, DukasTsai, RongkungJurič, Damijana MojcaTsai, Rong-KungJurk, KerstinTsai, Shiao-WenJurkowska, HalinaTsai, Shih-JenJurkowska, Renata ZofiaTsai, Shu-HuaiJurynec, Michael J.Tsai, Tsung-HuaJust, SteffenTsai, Wan-ChiJuzoń, KatarzynaTsai, Ying-ChiehJyotsna, MishraTsai, Yuan-ChenKabakov, Alexander E.Tsakmakidis, Ioannis A.Kabała-Dzik, AgataTsakovska, IvankaKabir, MorvaridTsampras, NikolaosKaburagi, TomokoTsang, Stephen H.Kaca, WiesławTsaniklidis, GeorgiosKačániová, MiroslavaTsapogas, PanagiotisKachamakova-Trojanowska, NeliTsarouhas, KonstantinosKachroo, PriyadarshiniTsarouhas, VasiliosKącka-Zych, AgnieszkaTsartsalis, StergiosKaczmarek, BeataTsatsakis, AristidisKaczmarek, KrzysztofTsaturyan, AndreyKaczmarek-Ryś, MartaTsay, Gregory J.Kaczor, Agnieszka A.Tscheliessing, RupertKaczorek, EwaTschernig, ThomasKaczorowski, Catherine C.Tse, BrianKaczyńska, KatarzynaTse, William K.F.Kadam, LeenaTseng, Fan-GangKadamoto, SuguroTseng, Jen-ChihKaden, ReneTseng, Wei-ChengKaesler, SusanneTseng, Yu-HsinKaether, ChristophTsergouli, KaterinaKafarski, PawelTsermpini, Evangelia EiriniKafel, AlinaTsiani, EvangeliaKagami, MasayoTsiarli, MariaKagey, Jacob DTsiartas, PanagiotisKageyama, DaisukeTsigkou, OlgaKageyama, HakutoTsika, ElpidaKahaleh, BasharTsim, KarlKähäri, Veli-MattiTsiolaki, Paraskevi L.Kai, KeitaTsirigotis, PanagiotisKai, MihokoTsitsekian, DikranKaida, DaisukeTsitsopoulos, Parmenion P.Kailu, YangTskhovrebov, Alexander G.Kain, VasundharaTsodikov, OlegKainulainen, VeeraTsoi, James Kit-honKaiser, Thomas M.Tsolaki, Anthony G.Kaitsuka, TakuTsolakis, Apostolos V.Kaja, SimonTsouh Fokou, Patrick ValereKaji, HiroshiTsubaki, MasanobuKaji, KosukeTsuboi, HirohitoKaji, ToshiyukiTsubomura, MiyokoKajiura, HiroyukiTsubota, AkihitoKajiya, HiroshiTsubota, TakuyaKajiyama, HiroshiTsuchida, KunihiroKakehashi, AnnaTsuchida, SachioKakeji, YoshihiroTsuchiya, HiroyukiKakinen, AleksandrTsuchiya, MasahikoKakinuma, SeiTsuchiya, YuichiKakinuma, YoshihikoTsuda, TakeshiKakizaki, TomohiroTsugawa, HitoshiKakkar, AshokTsui, JaniceKakkassery, VinodhTsui, Kuan HaoKalafateli, Aimilia LydiaTsuji, GakuKalani, KomalTsuji, HayatoKalaska, BartlomiejTsuji, HiroakiKalathiya, UmeshTsukahara, TamotsuKalatzis, VasilikiTsukahara, ToshifumiKalayda, GannaTsukamoto, OsamuKaldis, PhilippTsukamoto, TetsuoKale, AbhijitTsukaya, HirokazuKalemba, Ewa M.Tsukita, SachikoKalen, AmandaTsuruda, ToshihiroKalíková, KvӗtaTsutsui, N.Kalinec, FedericoTsutsui, ShigeyukiKalinin, Vladimir I. Tsutsumi, HiroshiKalinina, OlgaTsykunova, GalinaKalinka, EwaTsytsarev, VassiliyKalisz, AndrzejTu, MinKalisz, StanisławTu, ThomasKalita-de Croft, PriyakshiTubek, StanislawKallenborn-Gerhardt, WiebkeTucker, MatthewKaller, MarkusTucker, Torry A.Kalló, GergőTudela, JoseKallunki, TuulaTufarelli, VincenzoKalluru, PolirajuTukaj, StefanKalogirou, Andreas S.Tulotta, ClaudiaKalous, JaroslavTůma, ZdeněkKalpachidou, TheodoraTuna, Bilge GuvencKalra, DivyaTunaru, SorinKalska-Szostko, BeataTundo, SilvioKaltsatou, AntoniaTuomanen, ElaineKaltschmidt, BarbaraTuominen, HanneleKalucka, JoannaTupler, RossellaKamada, YoshihiroTURANLI, BesteKamal, Fadia AliTurano, MimmoKamal, Muhammad ShahzadTurano, PaolaKamanová, JanaTurato, CristianKamath, DivyaTurcan, ŞevinKamath, SandipTurck, Christoph W.Kambe, TaihoTurdo, AliceKamei, KaekoTuretta, MatteoKamenarska, Zornitsa GanchevaTurk, DusanKamenski, PiotrTurkmen, SerhatKameshima, SatoshiTürkösi, EdinaKameya, MasafumiTurkowski, KatiKaminaka, HironoriTurnbull, BruceKaminska, BozenaTurnbull, JohnKamińska, JoannaTurner, Arlener D.Kamiński, Tomasz W.Turner, Jason D.Kamitori, ShigehiroTurner, JonathanKamiya, TakehiroTurner, NeillKämmerer, Peer WTurner, Ronald B.Kamnev, AlexanderTuroverov, Konstantin K.Kamoto, ToshiyukiTurowski, Tomasz W.Kamoun, MalekTurrini, EleonoraKamysz, ElżbietaTurula, HollyKamysz, WojciechTurunen, Mikko P.Kanada, MasamitsuTuszynski, Jack A.Kanakis, IoannisTutone, MarcoKanamarlapudi, VenkateswarluTuukkanen, JuhaKanamori, TakaoTuuminen, TamaraKanaoka, MasahiroTuvi-Arad, InbalKanata, EiriniTwarużek, MagdalenaKanazawa, MasatoTwizere, Jean-ClaudeKanczler, Janos M.Tyagi, AshishKanda, AtsuhiroTyagi, MuditKanda, NaokoTyan, Yu-ChangKandhasamy, SathiyarajTylee, KarenKandil, SaharTylki-Szymańska, AnnaKandpal, ManojTyrka, MirosławKaneda, MasahiroTyszka-Czochara, MalgorzataKanekiyo, TakahisaTyunin, Alexey PKanelis, VoulaTyurin-Kuzmin, PyotrKanemura, NobuhiroTzanakakis, GeorgeKanetis, LoukasTzeng, Shu-LingKang, CongBaoTzima, Aliki K.Kang, DavidTzur, Yonatan B.Kang, DongchulUbaldi, MassimoKang, Ellen H.Uchida, ShizukaKang, EunjuUdagawa, NobuyukiKang, HeeminUddin, Golam MezbahKang, Hee-TaikUddin, Sardar M.ZiaKang, Hong-YoUdumula, VenkataKang, HunseungUebanso, TakashiKang, Jae SeungUebe, RenéKang, Jeong-HyunUeda, AkihiroKang, JianmingUeda, MotokiKang, JonghoonUeda-Nakanishi, TakakoKang, Ju-HeeUeffing, MariusKang, Kwon-KyooUeguchi-Tanaka, MiyakoKang, Kyung PyoUehara, YoshinariKang, LiUeno, MasakiKang, MinjeeUeno, TasukuKang, Nam SookUeno, YujiKang, Sang SunUesawa, YoshihiroKang, SeungwooUetake, HiroyukiKang, Sun-WoongUezumi, AkiyoshiKang, Tae-BongUgolini, FrancescaKang, Tae-HongUhde-Stone, ClaudiaKang, Taek JinUhl, EberhardKang, WeiUhm, Jae-SunKang, Yang JaeUhrig, R. GlenKang, Young-HeeUkken, FionaKangas, HeliUlasov, IlyaKani, KianUldrijan, StjepanKania, Stephen A.Ulicna, LiviaKannan, Mathur S.Ulisse, SalvatoreKannappan, RamaswamyUlivieri, CristinaKanno, TakahiroUllah, ImranKano, HideakiUllah, Md AshikKanoi, BernardUllah, MujibKant, ShivaUllmann, EnricoKanto, TakeshiUllmann, ReinhardKanugula, Anantha KoteswararaoUlmer, Candice Z.Kanwar, Yashpal S.Ulrich, BenjaminKanzaki, HiroyukiUlrich, CraigKao, Li-TingUm, Min YoungKao, PeterUm, SoyounKao, Shung-TeUmagiliyage, Arosha LokuKapagianni, PantelitsaUmanah, George K. E.Kapcia, Konrad JerzyUmansky, SamuilKaplan, BarbaraUmansky, ViktorKaplan, Joshua M.Umapathy, GaneshKapłon-Cieślicka, AgnieszkaUmapathy, Nagavedi SiddaramappaKappen, ClaudiaUmehara, MikihisaKapranov, SergeyUmekita, KunihikoKapravelou, GaryfalliaUmemori, JuzohKapsogeorgou, E.K.Umemura, AkiraKapur, NeerajUmemura, TsukuruKar, AdwitiyaUnden, GottfriedKar, AnirbanUnderhill, Suzanne M.Karabina, Sonia-AthinaUnderwood, Mark D.Karakaidos, PanagiotisUngaro, PaolaKaraki, Shin-ichiroUngefroren, HendrikKaraman, SinemUngureanu, CameliaKaramouzis, MichalisUngurianu, AncaKaran, DevUnković, NikolaKarantanos, TheodorosÜnlü, Cüneyt H.Karapanayiotides, TheodoreUnno, TatsuyaKarasawa, KenUnseld, MatthiasKarasik, DavidUntergasser, GeroldKarazniewicz, MartaUosaki, HidekiKarbownik, AgnieszkaUpadhya, Manoj AtmaramjiKarcagi, VeronikaUpadhyay, ChitraKarch, JasonUpadhyay, Rakesh K.Kardassis, DimitrisUppugunduri, Chakradhara RaoKardos, JuliannaUppulury, KarthikKargl, JuliaUr Rehman, Muhammad AtiqKarihtala, PeeterUraki, RyutaKarimi, KhalilUrbach, ValerieKarisola, PiiaUrban, JillKariya, YoshinobuUrban, MilanKariyawasam, HarshaUrban, OtmarKarkanis, AnestisUrban, PhilippeKarki, Hari SharanUrbanc, BrigitaKarkowska-Kuleta, JustynaUrbanek, KonradKarlsson, ErikUrbanek, OlgaKarlsson, Jan-OlofUrbaniak, AlicjaKaroly, HollisUrbaniak, JacekKarras, PanagiotisUrbaniak, MonikaKarsak, MelihaUrbano, Ana MargaridaKartsonaki, ChristianaUrbanska, Ewa M.Karuppiah, VijaykumarUrbański, ArkadiuszKarvinen, SiraUrban-Wojciuk, ZuzannaKarwowski, BoleslawUreshino, HiroshiKasai, FumioUrgel, Manuel EspinosaKaschina, ElenaUribe, Juan EstebanKashaninejad, NavidUrick, Mary EllenKashapov, Ruslan R.Urra, José MiguelKashfi, KhosrowUrtenov, MakhametKashimada, KenichiUrzi, ClaraKashina, AnnaUsama, Syed MuhammadKashiwagi, AkikoUshakova, Nina A.Kashiwakura, IkuoUshimaru, TakashiKashuba, ElenaUsmani, AbulKaski, Juan PabloUsui, NoriyoshiKasparov, SergeyUsui, TatsuyaKasprzak, AldonaUsui, YoshihikoKasprzak, ArturUtkin, Yuri N.Kasprzak, Magdalena PaulinaUusitupa, MattiKass, LauraUversky, VladimirKassai, HidetoshiUyeda, TaroKassassir, HassanUygun, SahraKassayová, MonikaUysal-Onganer, PinarKässmeyer, SabineUzbekov, Rustem E.Kasture, AmeyaUzbekova, SvetlanaKasuga, KensakuUziel, OritKasztan, MalgorzataV Ivanov, AlexanderKatagiri, HiroshiVaamonde, CarlosKatagiri, TakenobuVacca, FabrizioKataoka, HiroakiVaccarezza, MauroKataoka, Tatsuki RVach, KirstinKataoka, YoskyVachtenheim, JiriKatarzyna, MalarzVackova, IrenaKatase, NaokiVaclavikova, RadkaKatayama, ShigeruVáclavíková, RadkaKatayama, TakeshiVafiadaki, ElizabethKate, MaheshVaibhav, KumarKathare, Praveen KumarVaidotas, StankevičiusKathawala, RishilVaiman, DanielKatikou, PanagiotaVainonen, JuliaKatila, NikitaVairetti, MariapiaKatneni, UpendraVaiselbuh, Sarah R.Kato, KeitaValable, SamuelKato, Takamitsu AValadkhan, SabaKato, YasumasaValasi, IreneKato, YoshioValcke, RolandKato, YusukeValdés López, OswaldoKatoh, KazuoValdivielso, PedroKatona, EvaValdivieso, HenarKatona, GáborVale, CarmenKatrinli, SeymaVale, PauloKatsuhara, MakiValensin, DanielaKattner, LarsValente, JoanaKatyal, PriyaValente, SabrinaKatz, Ben Z.Valentina, VillariKauffman, KathrynValentini, FedericaKaufmann, Christopher N.Valentino, MarioKaul, RashmiValentová, KateřinaKauppinen, AnuValentovic, MonicaKaur, AmanpreetValentukeviciene, MarinaKaur, GurvinderValenzuela, Juan LuisKaur, HarleenValenzuela, StellaKaur, SimranjeetValerio, AlessandraKaushik, AjeetValiante, SalvatoreKaushik, AshleshaValicherla, Guru RaghavendraKaushik, Nagendra KumarValihrach, LukasKaushik, NehaValis, MartinKaushik, PrashantValiveti, ChaitanyaKaushik, ShellyValko, MarianKaushik, ShivaniValle, Luisa DallaKavanagh, DeanVallejo-Illarramendi, AinaraKavecan, IvanaVallesi, AdrianaKawabata, KyuichiVallespin, ElenaKawabori, MasahitoVallet, SoniaKawaguchi, NanakoValletti, AlessioKawahara, MasahiroValli, Adrian AlejandroKawakami, HisatoVallone, DanielaKawakami, MasanoriValotassiou, VarvaraKawakami, SusumuValsesia-Wittmann, SandrineKawakatsu, TaijiValtorta, SilviaKawakita, TetsuyaValverde, DianaKawamura, IzuruValverde, Miguel ÁngelKawamura, TeruhisaVan Ballegooijen, HanneKawano, KouichiroVan Bergen, TineKawano, SatoshiVan Beuningen, HenkKawasaki, HiroshiVan Beusechem, VictorKawata, KazumiVan Beusechem, Victor W.Kawauchi, KeikoVan Beusecum, Justin P.Kawiak, AnnaVan Calsteren, Marie-RoseKayser, KlausVan Cruchten, Steven J.Kayser, Reilly R.Van Cutsem, PierreKazakov, SergeyVan Dam, Anne-MarieKazakova, Oxana B.Van Dam, PeterKazama, TomohikoVan De Ven, RienekeKazeto, YukinoriVan De Vijver, KoenKazlauskas, ArunasVan Den Akker, GuusKazuaki, TaguchiVan Den Berg, BernardKazunori, MatsudaVan Den Berg, Marco AlexanderKe, Po-YuanVan den Brekel, Michiel Wilhelmus MariaKeane, Robert WVan Den Ende, WimKearsey, StephenVan Den Hoogen, LucasKeating, ElisaVan Den Meiracker, Anton H.Kędzierska, EwaVan Der Does, Anne M.Keeling, KimVan Der Kant, RikKeereetaweep, JantanaVan Der Merwe, MarieKehrel, BeateVan Der Nest, Magriet AKeightley, CristinaVan Der Pluijm, I.Keiper, Brett D.Van Der Sluis, Renée M.Keita, HadyVan Der Vaart, MichielKejík, ZdeněkVan Der Vorst, EmielKellenberger, StephanVan Der Wel, Myrtle J.Keller, AdrianVan Dyke, MarkKeller, JohannesVan Dyke, MichaelKeller, LanceVan Eijk, MarcoKeller, Nancy P.Van Erp, PietKellermayer, MiklosVan Esch, Betty C. A. M.Kelley, Dior R.Van Etten, Ellis S.Kello, MartinVan Gisbergen, Marike W.Kelly, RobertVan Gool, Stefaan W.Kemler, MartinVan Hook, M.J.Kemp, Michael G.Van Horn, WadeKempisty, BartoszVan Houten, BenKendall, TimVan Krieken, RichardKendal-Wright, Claire E.Van Loon, BarbaraKengelbach-Weigand, AnnikaVan Meurs, Jan C.Kenkel, W. M.Van Muiswinkel, Willem B.Kenmochi, TakashiVan Noorden, Cornelis Johannes ForrindinisKennedy, LindseyVan Nunen, SherylKennedy, Michael A.Van Parys, AlexanderKenney, KimbraVan Rechem, CapucineKennon-McGill, StefanieVan Ryn, JoanneKenny, Hilary A.Van Saen, DorienKenny, Nathan JamesVan Sanford, DavidKeogh, KateVan Schependom, JeroenKepinska, MartaVan Solingen, CoenKepp, OliverVan Straalen, Nico M.Kerckhove, NicolasVan Veluw, Susanne J.Kern, RamonaVan Vliet, SandraKerr, Stephanie C.Van Wijk, RichardKersten, SanderVan Wuytswinkel, OlivierKesari, Kavindra KumarVan Haute, LindseyKesharwani, SiddharthVanBuren, RobertKessel, DavidVancheri, FedericoKesterson, Joshua P.Vandekerckhove, BartKeszler, AgnesVanden Eynde, Jean JacquesKey, JaehongVandenbroeck, KoenKeyel, Peter A.Vandier, ChristopheKeyzers, RobertVandooren, JenniferKhacho, MireilleVangala, Janakiram R.Khadka, Vedbar SinghVangeli, DimitraKhalaf, FatimahVanham, GuidoKhalf, AbdurizzaghVanhie, ArneKhalighi, SanazVankayala, RavirajKhalyfa, AbdelnabyVankova, RadomiraKhambu, BilonVanmierlo, TimKhammanivong, AliVann, WillieKhan, AmjadVanneste, SteffenKhan, Husain YarVannucchi, Maria-GiulianaKhan, IrfanVanoni, SimoneKhan, MahmoodVanSaun, MichaelKhan, Meraj AlamVanscheeuwijck, PatrickKhan, Mohammad AslamVanthuyne, NicolasKhan, Mohd MohsinVaquero, MonicaKhan, MohsinVáradi, CsabaKhan, NababVaradwaj, PradeepKhan, SabbirVarallyay, EvaKhan, SajidVarani, KatiaKhan, SanaullahVarela, MonicaKhan, ShafiqVarela-Nieto, IsabelKhan, ShanjidaVarese, Giovanna CristinaKhan, SonjaVarga, MátéKhan, Wahab A.Varma, Vijaykumar B.Khanal, SameerVarna, MarianaKhananshvili, DanielVaroni, Elena MariaKhanh, Tran DangVarotto, ClaudioKhanna, HemantVarra, MichelaKhanna, RajeshVarrassi, GiustinoKharaeva, Zaira F.Vartiainen, Maria K.Kharche, Sanjay R.Vas, ViragKhattab, MuhammadVasaikar, Suhas V.Khaw, Kooi YeongVascotto, CarloKhella, HebaVaseva, Irina I.Kheradpezhouh, EhsanVasic, VesnaKhisamutdinov, EmilVasilchenko, AlexeyKhivansara, VishalVasile, FrancescaKhobta, AndriyVasilescu, CatalinKhodanovich, MarinaVasiljeva, OlgaKhong, AnthonyVasilyev, AlexanderKhoo, Bee LuanVaškelytė, JolantaKhoo, CheangVasquez, MarlenKhoshmanesh, KhashayarVassallo, JosanneKhozin-Goldberg, InnaVassallo, NevilleKhrustaleva, LiudmilaVassaux, GeorgesKhuchua, ZazaVassilaki, NikiKhung, Yit LungVassilakos, NikonKhurana, NamrataVassiliadis, ThemistoklisKhurshid, ZohaibVassiliou, Aliki GeorgiaKi, Sung HwanVasu, SrividyaKiang, Tony K. L.Vasudevan, SreelakshmiKibenge, FrederickVatter, HartmutKichik, NessimVaury, ChantalKiczorowska, BożenaVauthey, EricKida, SatoshiVaz, Daniela C.Kido, Mizuho A.Vázquez-Domínguez, EvaristoKieć-Kononowicz, KatarzynaVazquez-Hernandez, MariaKieda, ClaudineVázquez-Tato, M. PilarKiefer, ChristianeVázquez-Ucha, Juan C.Kieffer, BrunoVecchio, EleonoraKieliszek, MarekVecchio, MicheleKieran, Troy JVecerek, BranislavKietzmann, ThomasVechetti, IvanKiezun, MartaVecino Cordero, ElenaKijewska, MonikaVecoli, CeciliaKikis, Elise A.Vécsei, LászlóKikuyama, MasatakaVeenstra, RichardKil, Eui-JoonVeerabagu, ManikandanKilár, AnikóVeeramani, SureshKilari, SreenivasuluVeeramani, VediyappanKilb, WernerVeeravalli, VijayabhaskarKilinc, DevrimVega Alvarez, Jose AntonioKilk, KalleVega-Rubín-De-Celis, SilviaKim, A-RuVegeto, ElisabettaKim, BongjuVeglia, GianluigiKim, Boo-YoungVegvari, AkosKim, Byung S.Veit, GuidoKim, Byung SooVeith, PaulKim, Chang-BaeVelagapudi, RavikanthKim, Cheorl-HoVelasco, RobertoKim, Choon YoungVelasco-Hernández, TalíaKim, DokyoungVelayudhan, LathaKim, Dong HyunVelazquez, MiguelKim, Dong SeonVeldhuizen, EdwinKim, DonggeonVeldhuizen, Ruud A. W.Kim, Dong-HwanVelegzhaninov, Ilya O.Kim, Dong-HweeVelena, AstridaKim, Dong-KyuVeletza, StavroulaKim, DongukVélez, MariselaKim, EllaVelez-Cruz, RenierKim, EunheeVelicky, PhilippKim, Haeng-hoonVelikova, TsvetelinaKim, Hae-WonVelikova, VioletaKim, Hahn YoungVelisek, LiborKim, Ha-NeuiVelizarov, Svetlozar G.Kim, Han-JoonVellante, FedericaKim, Hee-JoonVellecco, ValentinaKim, Hee-TaekVemula, HarikaKim, Hong NamVenditti, MassimoKim, Ho-ShikVenerando, AndreaKim, Hye OneVenere, MonicaKim, Hye RyounVenier, PaolaKim, Hyeun SungVenkat, PoornimaKim, Hyun AhVenkatasubramanian, SambasivanKim, Hyun JoonVenkatesan, Jagadeesh KKim, HyungsikVenkateshaiah, Sathisha U.Kim, HyunhoVenkateshwaran, MuthusubramanianKim, Hyun-JungVenkateswaran, SureshKim, Hyun-SoonVennekens, RudiKim, Il-manVenneri, Mary AnnaKim, In JungVenter, HenriettaKim, InKyeomVentura, NatasciaKim, Jae BumVentura-Clapier, Renée E.Kim, Jae GeunVenturin, MarcoKim, Jae KwangVenturini, CarolaKim, Jae YoungVenugopal, DhamodharanKim, JaehanVenuti, ValentinaKim, Jae-HwanVera Rojas, JaimeKim, Jae-SeokVerbanac, DonatellaKim, Ja-EunVerbeek, D.S.Kim, Jae-wooVerbeke, KristinKim, JaeyoonVerde, FedericoKim, Jae-YoungVerdeguer, FranciscoKim, Jee HwanVerdijk, Robert M.Kim, Jee TaekVerdoucq, LionelKim, Jeong-HwanVerdú, EnriqueKim, Jeong-IlVeres, SzilviaKim, JeongtaeVerestiuc, LilianaKim, Jin SuVergadi, EleniKim, JinhoVergani, LauraKim, Jin-HongVergara, AlessandroKim, Jin-KyungVergely, CatherineKim, Jin-WookVerger, StéphaneKim, Jong-EunVerges, MarcelKim, JongkeeVergés, MarcelKim, JongminVerginelli, FabioKim, JoungmokVerhaeghe, JohanKim, Jung SunVerheyen, EstherKim, JungeunVerhoeven, AswinKim, JunghyunVerkhovtsev, AlexeyKim, JunmoVerleih, MariekeKim, Keun IlVerma, AnilKim, Ki HyunVerma, Dinesh KumarKim, Ki-TaekVerma, RatiKim, KyeongjinVerma, SureshKim, Kyoung SooVerma, VikashKim, KyounghyunVermaas, Joshua V.Kim, KyoungtaeVerna, GiulioKim, Kyung BoVernardou, DimitraKim, Kyung EunVernhettes, SamanthaKim, KyunggonVERNÌ, FiammettaKim, Kyung-MinVerona, GuglielmoKim, KyuseokVeronico, PasquaKim, Lenise JiheVerpeut, JessicaKim, Mee KumVerrecchia, FranckKim, Mi-hyunVerri, TizianoKim, Min JungVersacci, PaoloKim, Min-HyunVersino, ElisabettaKim, MinsikVerstockt, BramKim, Min-SooVerstraeten, AlineKim, Nam DeukVerstraeten, IngeKim, NamkwonVerza, Simone GasparinKim, NayoungVerzola, DanielaKim, Ok-KyungVerzoni, ElenaKim, Pyung-HwanVesa, Stefan CristianKim, Raymond HVeselska, OleksandraKim, Ryong NamVeskoukis, Aristidis S.Kim, Sang WanVetere, AmedeoKim, Sang-WeVetere, GisellaKim, SeilVetrano, Ignazio GaspareKim, Seog JuVetsika, Eleni-KyriakiKim, SeokheeVetvicka, VaclavKim, Seok-JunVezzani, BiancaKim, SeongjaeVianello, AndreaKim, SeyunViapiana, AgnieszkaKim, SoochongViappiani, CristianoKim, Soo-HyunViazzi, FrancescaKim, SooyeonVicario, NunzioKim, Soo-YoulVicentini, CaterinaKim, Su YoungVickstrom, Casey RKim, Su-JeongVictoni, TatianaKim, Sun KwangVictor, BrunoKim, Sun TaeViczian, AndrasKIM, Sung JoonVidal, LucianoKim, SungeunVidal, MiguelKim, Sung-Kun (Sean)Vidal, ReneKim, Tae IlVidalakis, GeorgiosKim, Tae MinVidana Mateo, BeatrizKim, Tae-HyungVideau, PatrickKim, Tae-JungVidetta, WalterKim, Tae-SungVidic, JasminaKim, Won HoVidovic, BojanaKim, WootaeVidovic, SinisaKim, YangmeeViehmannová, IvaKim, Yong JinVielba, Jesús MaríaKim, Yong KuViennet, ThibaultKim, Yong-IckVietinghoff, Sibylle VonKim, Yong-SikVieyra, AdalbertoKim, Yoon YoungVieyra, Jorge ParedesKim, Yoon-ChulVig, KomalKim, Young JunViganò, MarcoKim, Young-SangVigh, LaszloKimata, JasonVígh, LászlóKimball, JenniferVigna, ElisaKimber-Trojnar, ŻanetaVignard, JulienKimbrel, JeffreyVigneron, AurelienKimeklis, Anastasiia K.Vignes, MichelKimoto, MichikoVigodner, MargaritaKimura, KazuhiroVigouroux, CorinneKimura, SeisukeViguera, Ana RosaKimura, TomokiViguié, CatherineKimura, TsuyoshiVijay, Geraldine VidhyaKinarivala, NiharVijayakumar, SarathKindermann, HaraldVijayan, MadhuvanthiKing, Chih-YenVijayan, MuraliKing, Thomas F.J.Vijayavenkataraman, SanjairajKino, TabitoVikas, PraveenKinoshita, ChisatoVilasi, SilviaKinoshita, TakayoshiVilgelm, Anna E.Kinthada, LakshmanakumarVilla, AnnaKioussi, ChrissaVilla, ChiaraKirberger, MichaelVilla, FedericaKirby, Elizabeth D.Villafaina, SantosKirchmair, JohannesVillani, RosannaKirchner, SebastianVillanova, ValeriaKireev, Igor I.Villaseñor, AlmaKirimura, KohtaroVimberg, VladimirKirkby, KennethViña, DoloresKirov, RoumenVinall, RuthKirsanov, KirillViñas, MiguelKirsch, RoyVincenzi, ColombinaKirschnek, SusanneVincenzi, FabrizioKirschner, Michaela B.Vincenzo, De LeoKirubakaran, PalaniVinci, CristinaKisand, K.Vinciguerra, ManlioKiselev, AntonVindigni, VincenzoKiselev, Konstantin V.Vingrys, AlgisKishigami, SatoshiVinjamur, DivyaKishimoto, JiroVinogradov, EvgueniiKishimoto, ShinjiVinuesa, TeresaKishimoto, YoViola, GiampietroKishore Sakharkar, MeenaVirag, Jitka A.I.Kiskova, TereziaViranaicken, WildrissKiss, Anna L.Virgilio, AntonellaKiss, AttilaVirsolvy, AnneKiss, CsongorVirto, MailoKiss, ErzsébetVirumbrales-Muñoz, MaríaKiss, ÉvaVisai, LiviaKiss, RitaVisconte, ValeriaKiss, RobertVisentin, MicheleKitaguchi, TetsuyaVishe, MaheshKitahara, RyoVishnoi, KanchanKitajiri, Shin-ichiroVisser, Jenny A.Kitamura, AkiraVisser, LydiaKitano, YoshikazuVistoli, GiulioKitaoka, MotomitsuViswanathan, SowmyaKitase, YukikoViswanathan, VigneshKitaura, JiroVita, FedericoKitazawa, SoheiVita, RobertoKitsiouli, EIRINIVitale, ChiaraKittel, AgnesVitale, FabrizioKittl, SonjaVitale, GiovanniKiya, TaketoshiVitek, LiborKiyan, YuliaVitek, MichaelKjaer, LasseVitiello, Pietro PaoloKjeldsen, EigilVitorica, JavierKlaiman, CherylVitorino, Rui Miguel PinheiroKlampatsa, AsteroVitrac, HeidiKlampfer, LidijaVittorioso, PaolaKlapper, PaulVives-Peris, VicenteKlar, AgnesVivian-Smith, AdamKlassen, RolandVivo, MariaKlatte-Schulz, FrankaVizoso, Francisco J.Klavins, LinardsVizoso-Vázquez, AngelKlebert, SzilviaVlad, Cătălin IoanKleczkowska, PatrycjaVladimirov, VladimirKleeff, JörgVladislavić, NivesKleibl, ZdeněkVlahopoulos, SpirosKlein, BrittaVlahos, Nikos F.Klein, David C.Vlahovic, TraceyKlein, EricVlainić, JosipaKlein, Gordon L.Vlase, GabrielaKlein, GracjanaVlasiou, ManosKlein, Janet DVliagoftis, HarissiosKlein, KerstinVlk, MartinKlein, Mark A.Vo, DatKlein-Szanto, AndrésVo, Tommy VKlek, StanislavVoběrková, StanislavaKlementieva, OxanaVochita, GabrielaKlemme, SonjaVoelkers, MirkoKlener, PavelVoellger, BenjaminKlenov, Mikhail S.Vogel Ciernia, AnnieKlepikova, Anna V.Vogel, ChristophKlimas, JanVogel, MichaelKlimaszewski, LarsVogel, MoniqueKlimczak, AleksandraVögele, MartinKlimek, KatarzynaVogiatzis, GeorgiosKlimek-Chodacka, MagdalenaVogler, GeorgKlimek-Ochab, MagdalenaVogt, AndreasKlimontov, VadimVogt, MeganKlink, MagdalenaVoisin, MaudKlintsova, AnnaVoisset, CecileKlitgaard, JanneVojta, LeaKlobuch, SebastianVokurka, AlešKloc, MalgorzataVolante, AndreaKlocko, Amy L.Volcho, KonstantinKlontzas, MichailVoliani, ValerioKlotz, James LVolicer, LadislavKluczyk, AlicjaVolkov, VadimKlupp, BarbaraVollbrecht, ClaudiaKlussmann, EnnoVolny, MichaelKlyczek, KarenVolpato, MileneKmiec, Eric B.Volpe, ElisabettaKnaap, EstherVolta, MattiaKnap, NarcyzVon Ameln-Mayerhofer, AndreasKnappskog, Per M.Von Der Haar, TobiasKnäuper, VeraVon Der Thüsen, JanKnecht, HansVon Döbeln, UlrikaKnez, JureVon Hänisch, CarstenKnier, BenjaminVon Heideken, JohanKnight, MarcVon Horsten, Hans HenningKnight, Walter E.Von Knethen, AndreasKniss, Douglas A.Von Kopylow, KathreinKnobloch, KarstenVon Kügelgěn, IvarKnoch, EvaVon Morze, CorneliusKnölker, Hans-JoachimVon Schacky, ClemensKnoll, MarkoVondrasek, JiriKnoop, KathrynVoort, Peter Van DerKnowles, HelenVoortman, JensKnüchel, RuthVora, LalitkumarKnudsen, Jonas RVormann, JürgenKo, CheMyongVorotelyak, EkaterinaKo, FrankVoshkin, Andrey A.Ko, Sung-kyunVostinaru, OliviuKo, Swee-SuakVostrikova, Kira E.Kobayashi, HiroshiVourc’h, PatrickKobayashi, JunVrbacký, MarekKobayashi, KazuyaVrecl, MilkaKobayashi, KoichiVreeswijk, MaaikeKobayashi, MasakiVriesekoop, FrankKobayashi, SatoruVrolijk, Misha F.Kobayashi, TatsuyaVrzal, RadimKobayashi, YayoiVu, Lam DaiKobayashi, YoshiroVugrek, OliverKobold, SebastianVujanovic, LazarKobrak, Mark N.Vujosevic, StelaKocábek, TomášVukasinovic, NemanjaKoce, Jasna DolencVukicevic, SlobodanKoch, Clarissa M.Vukojevic, KatarinaKoch, KristineVulturar, RomanaKoch, WojciechVuts, JozsefKochan, Grazyna T.Vutukuri, RajkumarKochmanski, Joseph JohnVychytilova-Faltejskova, PetraKochneva, GalinaVyhnánek, TomášKočí, ZuzanaVykoukal, JodyKocira, AnnaVylkova, SlavenaKocsis, BelaVysotski, Eugene S.Kocsy, GáborWaberski, DagmarKoczyk, GrzegorzWachten, DagmarKodali, MaheedharWackerlig, JudithKodama, TakahiroWade, JohnKodet, OndrejWadzinski, ThomasKodidela, SunithaWagler, JörgKodja, HippolyteWagner, GerdKodziszewska, KatarzynaWagner, Hanna JeannineKoeffel, ReneWagner, Kay DietrichKoenen, RoryWahab, FazalKoga, HiroyukiWakabayashi, SadaoKoga, HisashiWakamatsu, KazumasaKoga, Jun-ichiroWałajtys-Rode, ElżbietaKoga, YoshikatsuWalcheck, BruceKogawa, TakahiroWälchli, SébastienKoh, DavidWalczak-Jędrzejowska, RenataKoh, Gar YeeWaldner, MaximilianKohanbash, GaryWaldum, HelgeKohara, YukihiroWali, JibranKöhl, GudrunWaligorski, PiotrKöhn, MarcelWaligórski, PiotrKohri, MichinariWalker, Anthony L.Kohwi-Shigematsu, TerumiWalker, Chandler L.Koide, TetsuyaWalker, Douglas G.Koike, AtsushiWalker, JessicaKoike, HarukiWalker, Lori A.Koike, KenzoWalker, RobertKoirala, RanjanWalker, Robert P.Koivisto, AriWalker, ValerieKoivumäki, JussiWallace, David R.Kojima, MasamiWallace, Graham R.Kojima, SoichiWallace, Heather M.Kok, WouterWallin, AndersKokabu, ShoichiroWallin, CeciliaKokh, Daria BWalline, HeatherKokkinidis, MichaelWalling, Jason G.Kokkola, TarjaWalne, Amanda J.Kokotou, Maroula G. Walsh, PamelaKoksharova, Olga A.Walter, IngridKolanowski, Jacek L.Walter, Maggie C.Kolar, MitjaWalter, UlrichKolasa-Wołosiuk, AgnieszkaWalters, Eugene HaydnKolawole, AbimbolaWalters, JudithKolcsár, MelindaWan, DehuiKoldehoff, MichaelWan, Timothy Ming HunKolesnikova, TatyanaWang, ​ZhenpingKoli, SwanandWang, AilinKolluru, GopiWang, Bang-AnKolmar, HaraldWang, BinKolobov, Alexandr AlexandrovichWang, ChaoKolodziejski, Pawel AntoniWang, Chi ChiuKolonko, AureliuszWang, Chia-YihKolosnjaj-Tabi, JelenaWang, ChihueiKolosova, NataliyaWang, Chrong-ReenKolukisaoglu, ÜnerWang, ChunKomada, MunekazuWang, ChunxiaKomaki, HisayukiWang, David Q.Komarova, Natalia V.Wang, Feng-ShengKomasa, SatoshiWang, FuyiKomati, RajeshWang, GuanKomatsu, HidetoshiWang, GuixueKomatsu, MasanobuWang, GuliangKomatsu, SatoshiWang, GuorongKomatsu, ToruWang, HaiKomives, TamasWang, HanjunKommireddy, VasuWang, HaoluKomori, ToshihisaWang, HongbingKomuraiah, MyakalaWang, HsiuyingKon, ShunsukeWang, HuaishanKondo, HiromuWang, Hui-minKondo, MotonariWang, JiafuKondo, TatsuyaWang, Jie (Jane)Kondoh, HiroshiWang, Jin’AnKonduri, VanajaWang, JingKondylis, EvangelisWang, Jing-QuanKones, RichardWang, JinkaiKonev, AlexanderWang, JinxiangKong Thoo Lin, PaulWang, JiongweiKong, Il-KeunWang, Jiun-LinKong, Kien VoonWang, Joshua JianxinKong, LishengWang, JunfengKong, QingzhongWang, KaiKonger, Raymond L.Wang, KankanKönig, GerhardWang, LeiKonishi, HiroakiWang, LeyiKono, MichihiroWang, LiKononikhin, Alexey S.Wang, Liang-ChaoKonopacka-Łyskawa, DonataWang, Liang-ChunKonopelski, PiotrWang, LihuaKonopka, AnnaWang, LirongKonrad, HeinoWang, LuKonstantakou, Eumorphia G.Wang, MingboKonstantinidis, Theocharis G.Wang, Nan-kaiKonstantinov, Spiro M.Wang, Pa-ChunKontek, BogdanWang, PeipeiKontogianni, GeorgiaWang, PengKontos, Christos K.Wang, PengchengKontsevaya, IrinaWang, Peng-HuiKonturek, Peter ChristopherWang, QingdeKoo, Bon-NyeoWang, QingjunKoo, Hee-beomWang, QinyiKoos, BjörnWang, RichardKoot, Bart G.P.Wang, Richard R.-CKopaczyńska, MartaWang, ShaobinKopanja, DraganaWang, ShenqiKopecký, DavidWang, ShikeKopecký, JanWang, ShuaishuaiKopel, PavelWang, ShueKopjar, NevenkaWang, SishuoKoppe, Janna G.Wang, SonglinKopra, KariWang, Tsung-JenKoprowski, PiotrWang, WeiKopsky, David J.Wang, Wen-DerKopustinskiene, Dalia M.Wang, WenjingKopyta, IlonaWang, WenxinKorac, AleksandraWang, Xiao QiKorać, PetraWang, XiaodongKorbecki, JanWang, XiaohongKorczeniewska, OlgaWang, XiaolingKordyukova, LarisaWang, XinkunKordyum, Elizabeth L.Wang, XueKorfei, MartinaWang, XuewenKorinek, VladimirWang, YanKorkhov, VolodymyrWang, YangKornhuber, Malte.Wang, Yang-KaoKornke, AlexWang, YingKornmann, MarkoWang, Ying-JanKoromyslova, AnnaWang, YiweiKorsching, EberhardWang, YongqiangKorytář, TomášWang, YoujinKorzhikova-Vlakh, EvgeniaWang, Yu-ChiehKorzhikov-Vlakh, ViktorWang, YumengKosacka, JoannaWang, YunKosan, ChristianWang, ZhanxiangKosecka-Strojek, MajaWang, ZhaoningKoshiba, TakumiWang, ZhigangKoskinas, JohnWang, ZilongKoskinen, PekkaWanger, SamuelKośla, KatarzynaWanigatunga, Amal AsiriKosmala, ArkadiuszWanke, DierkKosman, JoannaWann, AngusKošmerl, TatjanaWanrooij, PaulinaKosova, KlaraWard, Brian M.Kosowska, AgnieszkaWard, MarkKosslick, HendrikWard, Roberta J.Kostakis, IoannisWardowska, AnnaKošťál, VladimírWarnakulasuriya, SamanKostareva, AnnaWarnes, GaryKostecka-Gugala, AnnaWarzecha, ZygmuntKosters, AstridWase, NishikantKostner, Gert M.Waseem, AhmadKostopoulou, OuraniaWashino, SatoshiKostova, DimitrinaWasilewski, TomaszKostrominova, TatianaWasniewski, MarineKostyushev, DmitryWasson, ChristopherKosuge, YasuhiroWasylishen, AmandaKosutova, PetraWaszczak, CezaryKoswara, AndyWaszkiewicz, EwaKosyakov, Dmitry S.Waszkiewicz, NapoleonKoszelewski, DominikWatała, CezaryKota, SatyaWatanabe, EiichiKotani, TomoyaWatanabe, HiroshiKotanidou, AnastasiaWatanabe, MasayukiKotelevets, LarissaWatanabe, MikikoKotra, Lakshmi P.Watanabe, MutsumiKotsiou, Ourania S.Watanabe, NaokiKottakota, ChandrasekharWatanabe, SatoruKotta-Loizou, IolyWatanabe, TakafumiKottaridi, ChristineWatanabe, ToshioKoturbash, IgorWatanabe, YoichiKou, YanjunWatanabe, YuichiroKoukos, Panagiotis I.Wątek, MarzenaKoukoulaki, MariaWaters, Michael J.Koulen, PeterWatnick, Randolph S.Kounatidis, IliasWatson, ZacharyKouril, RomanWatterson, Daniel MartinKouroumalis, Elias A.Watts, JenniferKouroupis, DimitriosWatzky, MurielleKourtidis, AntonisWauters, LucasKoutelou, EvangeliaWautier, Jean-LucKouza, MaksimWawiórka, LeszekKovačević, BorislavWawruch, MartinKovacs, AnitaWawrzycka, DonataKovacs, LajosWazir, UmarKovacs, LaszloWcisło, PiotrKovacs, RenatoWculek, Stefanie K.Kovács-Öller, TamásWeake, Vikki M.Koval, AlexeyWeaver, ConnieKoval, MichaelWeaver, Samantha R.Koval, OlgaWebb, Carol F.Kovalchuk, NinaWebb, Lauren M.Kovalenko, Elena I.Webba Da Silva, MateusKovaleva, ValentinaWebber, CalebKovalská, MáriaWebber, ChristineKoven, WilliamWeber, Bernhard H.F.Kovinich, NikWeber, DanielKowalewska, MagdalenaWeber, JohnKowalska, JoannaWeber, RebekkaKowalska, KarolinaWebster, Jack M.Kowalska-Duplaga, KingaWebster, Nicholas J.G.Kowol, Christian R.Wędzony, MariaKowtharapu, Bhavani S.Weger, StefanKoya, RichardWeghofer, AndreaKoyama, NobuhiroWeglarz, WladyslawKoyama, YasuhitoWegrzyn, AlicjaKoyama, YutakaWęgrzyn, GrzegorzKoyuncu, OrkideWegwitz, FlorianKoyuncu, SedaWehling, PeterKozak, AshotWehrhan, FalkKozak, ChristineWei, David ChangliKozaki, AkikoWei, Gong-HongKozakiewicz, AnnaWei, Kai-CheKozakiewicz, MariuszWei, Wei-ChyouKozhevnikova, OyunaWei, WenyiKoziak, KatarzynaWei, YonglongKozicz, TamasWei, YufengKoziorowska-Gilun, MagdalenaWei, ZhishunKoziróg, AnnaWeickert, Martin O.Kozlov, KonstantinWeidner-Glunde, MagdalenaKozlowski, LukaszWeigand, Julia E.Kozlowski, Michael R.Weigmann, BennoKozlowski, PiotrWeihua, Zhang (John)Kozovska, ZuzanaWeinberg, Annelie-MartinaKrabbenhoft, Trevor J.Weingartner, MagdalenaKracher, DanielWeingärtner, OliverKrägeloh-Mann, IngeborgWeinhold, ArneKrainer, GeorgWeinstein, David A.Krajewska-Włodarczyk, MagdalenaWeinzierl, RobertKrajka-Kuźniak, ViolettaWeisel, John W.Krajnak, Kristine M.Weiser, Brian P.Krakowski, LeszekWeiser, Douglas C.Kramarenko, Elena YuWeiss, JohannaKramer, Boris W.Weiss, JuliaKrammer, Eva-MariaWeiss, MartinKranenburg, Richard VanWeißenbacher, AnnemarieKranendonk, MichelWeissert, RobertKraner, Susan D.Weisz, AlessandroKranz, MathiasWeldon, SineadKrappmann, DanielWellberg, ElizabethKrasheninina, Olga A.Wellinger, RalfKrasniqi, AhmetWellinger, Ralf-ErikKrásný, LiborWellington, CherylKrasuska, UrszulaWellington, MelanieKratochvilova, IrenaWells, TimKratochwill, KlausWelsh, C. JaneKratzke, Robert ArthurWelsh, JaneKrauklis, Andrey E.Welsh, JoshuaKraus, AllisonWelsh, MichaelKraus, DominikWeltens, NathalieKraus, PetraWempe, MichaelKrause, GerdWen, JianjunKrause, GünterWen, WeiKrause, JuliaWen, XiaoyanKravats, AndreaWen, Yu-ChingKravets, VolodymyrWenbin, ZhanKrawczyk, KonradWende, Adam R.Krazinski, Bartlomiej EmilWeng, Ching FengKrchnak, ViktorWeng, XiaoyuKrčma, FrantišekWenger, Roland HKrebs, JoachimWeraduwage, Sarathi M.Kreft, MarkoWerbrouck, Stefaan P.O.Kregiel, Dorota MariaWerlé, ChristopheKreimer, SimionWerneburg, SebastianKreis, Nina-NaomiWerner, Craig T.Krela-Kaźmierczak, IwonaWerstuck, GeoffKrell, TinoWesołowska, OlgaKřen, VladimirWess, TimKrenek, PeterWest, NicholasKresoja-Rakic, JelenaWest, RachelKress, MichaelaWesthaus, SandraKrestinina, OlgaWesthoff, Mike-AndrewKretov, DmitryWestmark, CaraKretz, MarkusWestphal, AdrieKreuger, JohanWetering, Koen Van DeKrieger-Liszkay, AnjaWeyand, NathanKrieghoff-Henning, EvaWhale, AlexandraKrijnse-Ĺocker, Jacomine K.Wheeler, Nicole EKrishna, Somashekar G.Whelan, FionaKrishnakumar, ShyamWhirl Carrillo, MichelleKrishnan, NatrajWhitaker, Brian DKrishnan, VinuWhitcomb, DavidKristensen, MieWhite, ChristineKritchenkov, Andreii S.White, ElizabethKritsiligkou, ParaskeviWhite, James P.Kriz, JanWhite, JohnKrizbai, István A.White, Ryan M.Kroeker, AndreaWhite, Stormi PulverKrogfelt, KarenWhitehead, Debra E.Krojer, TobiasWhiteside, Theresa L.Krokidis, MariosWhitfield, PhilKrokosz, AnitaWhiting, RebeccaKrol, SilkeWhyard, SteveKrólicka, AgnieszkaWhyte, ClaireKrólicka, AleksandraWibowo, DavidKronenberg, DanielWickner, Reed BKroner-Milsch, AntjeWicky Collaud, ChantalKropp, MartinaWidemann, EmilieKroupin, PavelWidera, DariusKrstic, JelenaWidłak, WiesławaKrug, SebastianWie, Myung-BokKrug, Susanne M.Wieckiewicz, MieszkoKrüger, ElkeWieczfińska, JoannaKrüger, MarcusWieczorek, EdytaKruithof De Julio, MariannaWiedlocha, AntoniKrum, Susan A.Wiedman, GregoryKrummenacher, ClaudeWiegmans, Adrian P.Krupa-Małkiewicz, MarcelinaWieland, ThomasKruse, RikkeWielockx, BenKruspig, BjornWierichs, RichardKruszewski, MarcinWierońska, JoannaKruzel, Marian L.Wierzbicka, JustynaKryger, PerWiese, MariaKrylov, Sergey N.Wiesner, RudolfKrystyna, Skalicka - WoźniakWiggs, MichaelKržan, MojcaWight, ThomasKrzeptowski, WojciechWigle, JeffreyKrzyściak, WirginiaWiglusz, KatarzynaKrzystanek, MarekWijaya, RameshKrzystek-Korpacka, MalgorzataWijtmans, MaikelKrzysztof Nowak, JanWikel, StephenKrzyźowska, MaŁgorzataWiktorska, KatarzynaKshirsagar, Prakash G.Wilczek, PiotrKsiążek, KrzysztofWilde, AndrewKsiążkiewicz, MichałWilde, H. DaytonKu, ChuanWildemann, BrittKu, Kang-MoWilding, MatthewKu, Seung-YupWiley, LukeKu, Yee ShanWilhelm, KerstinKuan, Yu-HsiangWilhelm, ThereseKuba, MichaelWilk, AleksandraKubaczka, CarolineWilkins, Charles L.Kubala, LukášWilkins, HeatherKubala, SzymonWilkinson, Adam C.Kubatka, PeterWilkinson, Mark D.Kubatzky, KatharinaWilkinson, Miles F.Kubiak, KatarzynaWille, HolgerKubicova, LenkaWillemsen, ViolaKubilius, RaimondasWillett, JuliaKubinová, ŠárkaWilliams, David L.Kubiński, KonradWilliams, Jessica A.Kubinyi, MiklósWilliams, JohnKubisz, PeterWilliams, Stephen J.Kubo, AsakoWilliams, TrevorKubo, AtsushiWillige, Björn C.Kubo, EriWilling, Alison E.Kubo, YoshinaoWillis, RohanKucera, RadekWillmann, Matthew RKucerova, LucieWillner, PaulKucharíková, SoňaWilm, MatthiasKucharska, KarolinaWilmowicz, EmiliaKuchay, Shafi M.Wilson, Anne M.Kuchler-Bopp, SabineWilson, CallumKuçi, SelimWilson, Debbie L.Kucinski, KrzysztofWilson, George D.Kück, UlrichWilson, IainKućko, AgataWilson, Jamie L.Kucuk, IsrafilWilson, JoyceKucukoglu, MelisWilson, MichaelKuczera, PiotrWilson, PeterKudaibergenov, SarkytWilson, Philippe B.Kudo, YasuseiWilting, JorgKudoyarova, GuzelWilton, SteveKudryasheva, Nadezhda S.Wilusz, JeffKudryashov, Dmitri S.Wimmer, ZdenekKudryavtsev, Denis S.Wimmers, KlausKuehne, ThomasWinchester, LeeKues, Wilfried A.Winckler, ThomasKufer, T. A.Windberger, UrsulaKugler, Matthias ChristianWindhorst, SabineKuhnast, BertrandWindler, EberhardKuhnert, EricWindshügel, BjörnKujan, OmarWinfree, Christopher J.Kujawa, KatarzynaWiniarczyk, KrystynaKujawska, MałgorzataWiniarska-Mieczan, AnnaKukita, YojiWinkler, JohannesKukull, Walter A.Winkler, LarsKulasinghe, AruthaWinn, NathanKulcenty, KatarzynaWinneke, GerhardKülheim, CarstenWinocur, EphraimKuliczkowski, KazimierzWinter, ElizabethKulig, DominikaWinter, LilliKulik, AnnaWinther, OleKulkarni, VarunWinum, Jean-YvesKulma, AnnaWiraja, ChristianKulma, MagdalenaWirsching, Hans-GeorgKulminskaya, Anna A. Wirth, MarkusKulminski, AlexanderWirths, OliverKulus, DariuszWirz, LisaKumai, TakumiWise-Draper, Trisha M.Kumamoto, EiichiWiśniewski, AdamKumar, AkhileshWisniewski, MarekKumar, AlokWisniewski, Michael E.Kumar, AnujWitczak, Carol A.Kumar, ArunWitkin, Jeffrey M.Kumar, AshutoshWitkowska, DanutaKumar, BrajeshWitkowska-Piłaszewicz, OlgaKumar, GauravWitrick, KatherineKumar, HirdeshWitt, StephanKumar, JustinWitte, Claus-PeterKumar, K.J. SenthilWittig, IlkaKumar, NareshWitting, Paul K.Kumar, NitinWittner, LuciaKumar, PijushWitwer, KennethKumar, PrashantWitzany, GuentherKumar, PraveenWitzel, KatjaKumar, RajWlaz, PiotrKumar, RajeshWlaź, PiotrKumar, RameshWłodarczyk, MaciejKumar, SantoshWłodarczyk, MartaKumar, SatishWłoga, DorotaKumar, ShaileshWnuk, AgnieszkaKumar, SujeetWnuk, MaciejKumar, SuneelWoad, Kathryn J.Kumar, UjendraWohlleber, DirkKumarasinghe, Sujith Prasad WanniarachchiWohlmuth, ChristophKumaraswamy, ChitralaWójcik-Jagła, MagdalenaKumari, NeelamWójcikowska, BarbaraKumari, SonamWójcik-Pszczoła, KatarzynaKume, KensukeWojdyło, AnetaKumita, JanetWojtas, BartoszKummer, KaiWojtunik-Kulesza, Karolina AnnaKumpulainen, TatuWojtyczka, RobertKumru, BarisWolach, OfirKun, JozsefWołejko, ElżbietaKundrata, RobinWolf, PetraKunej, TanjaWolff, Anette Susanne BøeKungl, AndreasWolfgang, SchreinerKunikazu, TanjiWolfinger, Michael ThomasKunoh, TatsukiWolfs, Tim G.A.M.Kun-Rodrigues, CéliaWolińska, AgnieszkaKunwar, SanjuWollebo, Hassen S.Kunz, WolframWołowiec, StanisławKuo, Cheng-ChinWolski, KarolKuo, Hann-ChorngWolthers, KirstenKuo, Hsiao-MeiWołyniec, WojciechKuo, Ko-LinWong, Chin-CheanKuo, Ming-TseWong, Ching-OnKuo, Pao-LinWong, ChungKuo, Shu-JuiWong, Clarence T. T.Kuo, Szu-ChengWong, Danny K. Y.Kuo, Yao-HaurWong, Hovy Ho-WaiKupai, KrisztinaWong, Lee LeeKupcova Skalnikova, HelenaWong, Man-kinKüpper, Jan-HeinerWong, MauriceKuppusamy, ManiselvanWong, WingKurabi, ArwaWong, YvetteKurakula, KondababuWoo Lee, GunKurano, MakotoWoo, So-YounKurańska, M.Wood, JasonKurańska, MariaWood, Scott T.Kurczyńska, EwaWoodbury-Smith, MarcKurg, ReetWoodrow, PasqualinaKurganov, Boris I.Woods, Daniel F.Kurien, Biji TheyilamannilWorch, RemigiuszKurihara, ToshihideWorkman, VictoriaKurihara, YukioWormit, AlexandraKurita, HisakaWorrall, MargaretKurmasheva, RaushanWorwa, GabriellaKuroda, DaisukeWoscholski, RudigerKuroda, TeruoWosczyna, MichaelKurowska, MarzenaWöstemeyer, JohannesKurt, Robert A.Wozniak, MichalKurumaddali, AbhinavWoźniak-Budych, MartaKüry, PatrickWozniak-Knopp, GordanaKuryk, LukaszWree, AndreasKusaczuk, MagdalenaWright, CatherineKusano, ShuheiWrobel, MariaKusewitt, Donna F.Wróbel, TomaszKushima, MikiWrona, EwaKushner, Sidney R.Wrzesinski, JanKusko, Rebecca LWrześniok, DorotaKuter, KatarzynaWrzosek, MałgorzataKutwin, MartaWu, CenKuure, SatuWu, Chang-YiKuwabara, TakuWu, Chen-ChiKuwahara, MasayasuWu, Chiao-EnKuwar, Suyog S.Wu, Chin-ChungKuwata, HiroshiWu, Chi-ReiKuzhir, PolinaWu, Chun-ChiKuzmanovic, LjiljanaWu, ChungChunKuźmicki, MariuszWu, Chun-TeKuzmin, ElenaWu, GongweiKuznetsova, Irina M.Wu, Guan-JamesKuźniar, AgnieszkaWu, Hsien-MingKuzuya, AkinoriWu, Hsien-TsaiKvandova, MiroslavaWu, Hsuan-ChenKwa, Faith A.Wu, JenderKwack, KyuBumWu, Jian-YongKwak, ShinWu, Jin-MingKwakowsky, AndreaWu, JohnKweon, Oh-KyeongWu, JunKwiatkowska, EwaWu, KaiKwiecien, JacekWu, KejunKwok, Henry Hang FaiWu, MengxiKwon, Choon-TakWu, Meng-YuKwon, HyokjoonWu, NanKwon, Seong YoungWu, PeixinKwon, So HeeWu, Ping-HsunKwon, Soon HyoWu, QingyuKwon, Soon JaeWu, Sheng-NanKwon, Soon KilWu, Shu-JuKwon, Tae-HwanWu, Tzong-YuanKwon, Woo-SungWu, Wei-LiKwon, Yong-ukWu, Wen JengKwong, Lawrence N.Wu, XianfangKwong, Yok-LamWu, YingKwun, Hyun JinWu, YoujunKyrgios, IoannisWu, Yu-JenKyunghwan, KimWu, ZeguangL’homme, LaurentWuebbles, RyanL’Ollivier, CoralieWullaert, AndyLa Clair, JamesWylie, Benjamin J.La Favor, JustinXanthou, GeorginaLa Forgia, DanieleXaplanteri, PanagiotaLa Penna, GiovanniXavier, Cristina Pinto RibeiroLa Rosa, CarmeloXavier, Nuno M.La Rosa, MassimoXi, GuifaLa Rosa, PiergiorgioXi, XinpingLa Spina, MartinaXi, ZhengxiongLa Teana, AnnaXia, JianguoLa Vieille, SébastienXia, TianLa Vignera, SandroXiao, LiLaakmann, ElenaXiao, XiaLabazi, HichamXiao, XinxinLabbé, JessyXiao, ZhennaLabbozzetta, ManuelaXiaobin, HuangLabeit, SiegfriedXiaohan, WangLabi, VerenaXiaoli, AlusLabo, NazzarenaXie, HangLaboisse, Christian L.Xie, JenniferLabro, AlainXie, LishiLabrou, NikolaosXie, MinLabruna, GiuseppeXie, MouzheLabrune, PhilippeXie, RuiliLabus, KarolinaXie, TingLacadena, JavierXie, Wei-dongLacal, Juan CarlosXie, ZhiqunLăcătușu, Cristina-MihaelaXie, ZhongqiuLach, RalfXie, Zhong-RuLacham-Kaplan, OrlyXilouri, MariaLachance, Marc-AndréXin, YinLachmann, NicoXing, GengmeiLachowicz, DorotaXing, MinliLacis, GunarsXiong, JiaLackie, Peter M.Xiong, XinLackner, NinaXodo, LuigiLacomme, ChristopheXu, ChuanLacza, ZsomborXu, ChuanmingŁączkowski, Krzysztof Z.Xu, GegeLademann, HanneXu, HongyanLadomery, Michael R.Xu, JianLadoux, AnnieXu, JinLadyzynski, PiotrXu, JinbinLaezza, FernandaXu, JunwangLaffey, John G.Xu, KuiLafond, MickaëlXu, LuLafont, ElodieXu, PengLafont, JeromeXu, SuowenLafontaine, DenisXu, WayneLafontant, Pascal J.Xu, WenyanLafrenaye, AudreyXu, XiaojiangLaganà, BrunoXu, XingboLagarkova, MariaXu, YangLaghezza, AntonioXu, Yi-JunLagoumintzis, GeorgeXuan, ShouhuLagrange, JeremyXuan, ZhenyuLaguna, JCXue, MeilangLagunas, BeatrizXue, XiangLaher, IsmailXue, XingjianLahiri, SatadruXue, XufengLahiri, TanayaXynias, Ioannis N.Lahlil, RachidYadav, DhananjayLahner, EdithYadav, Dharmendra K.Lahooti, HooshangYadav, HemrajLahuta, Lesław BernardYadav, MarshleenLai, Chian-HuiYadav, PramodLai, Chien-HanYadav, ShardaLai, Chih-ChiaYadav, Vikash KumarLai, Chih-HoYadav, VinitaLai, Chyong-HueyYadavalli, Nataraja SekharLai, HungchengYager, EricLai, Jui-YangYagi, HiroshiLai, JunyunYagi, NaotoLai, KentYagihashi, SorokuLai, Ming-DergYagishita, YokoLai, Ping-ShanYahiroda, HidekiLai, Thung-SYaish, MahmoudLai, Wen-FuYakavets, IlyaLai, XueleiYakimovich, ArturLai, YanhaoYakovlev, IgorLai, Yen-Chun (Charly)Yakovlev, VasilyLai, Yi-ChenYakymovych, IhorLai, YingJuYamada, HiroyukiLai, Yu-HengYamada, KenjiLai, Zon WengYamada, MasanoriLaine, MikaYamada, MasumiLaing, William AYamada, SohsukeLaizé, VincentYamada, TaketoLajic, SvetlanaYamada, YoheiLakin-Thomas, Patricia L.Yamaguchi, AkiraLakk, MónikaYamaguchi, KiyoshiLakomek, Nils AlexanderYamaguchi, NaohiroLakota, JanYamaguchi, SoichiroLakshmikuttyamma, AshakumaryYamaguchi, YoshihiroLalak-Kańczugowska, JustynaYamakawa, HiroyukiLall, Gurprit S.Yamakoshi, HiroyukiLalmanach, GillesYamamoto, SuguruLalowski, MaciejYamamoto, ToshiharuLam, Chi KeungYamamoto, YusukeLam, Sio-HongYamamura, HisaoLam, YanYamamura, SoichiroLamar, JohnYamanaka, KenyaLaMarca, BabetteYamanaka, RyuyaLamas, J. AntonioYamane, KyokoLamatsch, DunjaYamanishi, KiyofumiLamb, DavidYamaoka, YusukeLamb, LauraYamasaki, MasahiroLamba, DorianoYamashiro, SawakoLambert, KellyYamashita, AkiraLambova, SevdalinaYamashita, AtsushiLambrinidis, GeorgeYamashita, MichiakiLambrou, George IYamato, MasayukiLamers, Marcelo LazzaronYamato, TakehikoLamm, Monica H.Yamauchi, AkiraLamort, Anne-SophieYamauchi, YoshioLampert, AngelikaYamawaki, YosukeLampiasi, NadiaYamazaki, HiroyukiLamport, Derek T. A.Yan, AnLan, Chou-ChinYan, ChunhongLan, LanYan, DayunLan, YuchengYan, HaidongLana, DanieleYan, Hongbin (Tony)Lancel, SteveYan, Liang-junLancella, LauraYan, XiaLandau, DanielYan, Yong-BinLanderos-Weisenberger, AngeliYan, ZiyingLandi, LuciaYanaka, SaekoLandi, SimoneYanda, MuraliLandi, VincenzoYáñez-Mo, MaríaLandon, CelineYang, Ai-LunLandreville, SolangeYang, Chang-HaoLandrieu, IsabelleYang, Chao-HsunLandry, MarcYang, Chao-YieLandsberger, NicolettaYang, Chen-ILane, Hsien-YuanYang, Chih-JenLang, DiYang, Chih-YuLang, FengchaoYang, Chin-YingLang, PatriciaYang, Chun ZhangLange, SigrunYang, ChunhuaLange, TobiasYang, ChunzhangLangelaan, David N.Yang, DaiwenLangendorf, ChristopherYang, Deng-HoLanger, Gitta JuttaYang, DeyingLanger, IngridYang, GuangdongLangsjoen, RoseYang, Hung-ChiLanikova, LucieYang, Jee MyungLankinen, MariaYang, JialeLannigan, JoanneYang, Jiann-JouLanning, NathanYang, JiminLansman, Jeffry B.Yang, JunLanza, GiuseppeYang, JunhuaLanzafame, Raymond JYang, Kai-ChiangLaoudj-Chenivesse, DalilaYang, Kevin Y.Laoui, DamyaYang, Kuang-YaoLappano, RosamariaYang, KunLaPres, John J.Yang, LiliLapteva, MariaYang, LingliLapucci, AndreaYang, MinoYangLarauche, MurielYang, PingfangLarena, InmaculadaYang, QienLargy, EricYang, QiyuanLarionova, IrinaYang, Rong-CaiLark, DanielYang, SejungLarkindale, JaneYang, Seung HwanLaRock, Christopher N.Yang, SeunghwanLaron, ZviYang, ShengmingLarre, IsabelYang, Shun-ChinLarriba, EduardoYang, Shun-FaLarsen, MelindaYang, Soo JinLarson, GöranYang, Sung HoLarussa, TizianaYang, Tzu-SenLasagni, LauraYang, WeibingLascano, JorgeYang, YannanLasik-Kurdyś, MałgorzataYang, Ying-YingLasko, PaulYang, YongkangLasoń, WładysławYang, Young MokLasonder, EdwinYang, YueLassche, SaskiaYang, Yueh-Hsun KevinLászló, CsambalikYang, ZhihongLatajka, ZdzisławYann, AudicLateef, ZabeenYanniccari, MarcosLatek, DorotaYano, HajimeLatella, GiovanniYano, MasatoLatendresse, MarioYano, ShozoLatini, AndreaYano, ShuyaLatinovic, OlgaYano, YoshiakiLatorre, EvaYano, YoshihikoLatour, SylvainYao, Humphrey H.Latowski, DariuszYao, JiayiLattanzi, AnnalisaYao, Qizhi CathyLatunde-Dada, Gladys OluyemisiYao, WeiLau, AdelineYao, ZhenqiangLau, Allison N.Yap, DesmondLau, BenjaminYap, Kiryu K.Lau, ChunghoYapa, Asanka SajiniLau, JosephYaron, Jordan R.Lau, Kin H.Yarus, Michael J.Lau, Wei LingYarushkina, Natalia ILauf, PeterYasoda, AkihiroLaufs, PatrickYasuda, NinaLäuger, JörgYasuhara, TakaoLaughton, Charles A.Yasuhiko, KogaLaura, RossoYasuko, ManabeLaurent, PatrickYates, Nathanael J.Lauretani, FulvioYatoh, ShigeruLauretta, RosaYatsunyk, LiliyaLauricella, MariannaYavin, EylonLaurin, JérômeYazawa, KenjiroLauritano, DorinaYazawa, TakashiLauro, ClotildeYazdi, ImanLauro, DavideYazdimamaghani, MostafaLauronen, JouniYazlovitskaya, Eugenia M.Laursen, Kristian BruunYe, HaobinLauterbach, John H.Ye, JiangchuanLauwers, ElsaYe, LinLavender, AndrewYe, WenleiLavista Llanos, SofíaYe, YuLavogina, DarjaYe, ZhiweiLavrik, InnaYee, Callista StephanieLavrova, Anastasia I.Yee, DouglasLawas, Lovely Mae F.Yeger, HermanLawson, CharlotteYeh, Chih-KoLawson, KimYeh, Ching-HuiLazar, CatalinYeh, Chun-NanLazarev, VladimirYeh, Elizabeth S.Lazarević, VladimirYeh, Jun-JunLazaridis, GeorgiosYeh, Kuang-ShengLazaris, AnthoulaYeh, Shu-LanLazaro-Dieguez, FranciscoYeh, Te-HueiLazarowski, AlbertoYehya, NadirLazartigues, EricYélamos, JoséLazennec, GwendalYeliseev, AlexeiLazutka, JuozasYellapu, Nanda KumarLazzara, GiuseppeYen, B. LinjuLazzarato, LorettaYen, Frances T.Le Clec’h, WinkaYen, KelvinLe Dévédec, SylviaYen, Meng-ChiLe Guillou, DouniaYen, Yi-TingLe Huërou-Luron, IsabelleYengo-Kahn, Aaron M.Le Joncour, VadimYeo, SeonjuLe Magueresse-Battistoni, BrigitteYeo, SynLe Romancer, MurielYepuri, Nageshwar R.Le Saux, OlivierYeremenko, NataliyaLe, Hoang-ThanhYerushalmi, RinatLe, MinhYeste, MarcLeabu, MirceaYetnikoff, LeoraLeach, Jennie B.Yevtushenko, DmytroLeach, KatieYi, Ae-KyungLeão, Pedro N.Yi, GibumLeavesley, DavidYi, Sang AhLebar, MatthewYi, TaoLebaron, RichardYi, Young-SuLebaron, SimonYilmaz, MehmetLebedev, IgorYim, Soon-HoLebedev, VadimYimlamai, DeanLebenthal, YaelYin, ChuntaoLebert, MichaelYin, GuoweiLebreton, StéphanieYin, KingsleyLebrón, José AntonioYin, WenqingLEBRUN, Marc-HenriYin, YinLebrun, VincentYing, HaoLebwohl, Mark GYing, LeLecca, SalvatoreYing, MingyaoLechanteur, AnnaYinnon, Tamar A.Lechel, AndreYip, Bon HamLechner, Bob-DanYip, Ping K.Leclercq, GuyYlostalo, Joni H.Leclercq, LoicYochum, Gregory S.Lecona, EmilioYoder, KristineLedda, CaterinaYohda, MasafumiLedda, MarioYohe, Marielle E.Ledesma, Ana EstelaYokawa, KenLedesma, Maria DoloresYokoi, FumiakiLedo, AnaYokosyo, KengoLee, An-RongYokota, TakafumiLee, Bong HoYoneda, ToshiyukiLee, Brenda Y.Yong, JeongsikLee, Byong-taekYoo, Hah YoungLee, Byoung DaeYoo, Jung SunLee, Chang-HoonYoo, So YoungLee, ChangshingYoo, Yeong-MinLee, Chang-SooYoo, Yung JoonLee, ChangWooYoon, Do-YoungLee, ChanhuiYoon, HyeonseokLee, Che-HsinYoon, HyonokLee, Cheng-IYoon, IlLee, Chia-HwaYoon, Joon-KeeLee, Chien-TeYoon, MinjoongLee, Chih-HungYoon, Sung-SooLee, Ching-ChiYoong, MichaelLee, Ching-KuoYorgan, TimurLee, Choong-GuYoshida, AkihiroLee, ChoonghoYoshida, HiroshiLee, Chung-HaoYoshida, KaoruLee, Dae HoYoshida, KatsunoriLee, Dae-HeeYoshida, KentaroLee, DarrenYoshida, ManabuLee, Dong HunYoshida, NaofumiLee, Dong RyulYoshida, NaoyasuLee, Eugene S.Yoshida, ShinichiroLee, Eun JungYoshida, TakuyaLee, Eun YoungYoshida, ToshikoLee, Eun-WooYoshida, YukariLee, HeedooYoshie, MikihiroLee, HeonsooYoshigaki, JunkoLee, Hiang KweeYoshikuni, YasuoLee, Hsin-ChenYoshimatsu, MitsuhiroLee, HsinyuYoshimoto, Francis K.Lee, Hye YoungYoshimura, MasamiLee, Ik JaeYoshinaga, ArataLee, I-TaYoshinari, KouichiLee, Jae WookYoshino, HironoriLee, JaecheolYoshino, MiyaLee, Jeong WookYoshioka, Ken-ichiLee, Jeong-DongYoshioka, TaiyoLee, Jetty Chung-YungYoshioka, WataruLee, Ji MinYosten, GinaLee, Jin-YongYotova, Iveta Y.Lee, Joo HyoungYou, Frank M.Lee, JoohyungYou, HongLee, Jue-YeonYou, Hye JinLee, Jun HeeYou, Hyun JuLee, Jung RoYounès, DELLEROLee, Jung SeokYoung, ArabellaLee, Jung-KulYoung, Eric M.Lee, JungwooYoung, Howard A.Lee, Keun-HyeungYoung, Jason C.Lee, KwanukYoung, JessicaLee, Kyung JunYoung, John S.Lee, KyungminYoung, MichaelLee, Li-AngYoung-Jin, SonLee, Li-JenYoungstrom, Daniel W.Lee, Man RyulYounis, ShadyLee, Meng-shiouYousefi, MortezaLee, Min YoungYoussef, ZahraaLee, Myung-ShikYoussefian, LeilaLee, Peter AYruela, InmaculadaLee, Sang BongYu, Cheng-ChiaLee, Sang JinYu, Chia-LiLee, SanghoYu, ChunsongLee, SangyeopYu, FengqunLee, Seung HeeYu, GangLee, Seung HwanYu, Hsu-ShengLee, Seung-HongYu, Il-JeoungLee, Seung-HwanYu, Jian Q.Lee, SewonYu, JingyouLee, Shyi-LongYu, JiujiuLee, Soo InYu, KitaokaLee, Soo YoungYu, LuLee, Soo-younYu, MeifangLee, StellaYu, MinzhongLee, SujinYu, TianzhengLee, Sung KiYu, WeilingLee, Sung-JaeYu, XiangLee, Sun-YoungYu, YanboLee, Tet WooYu, YanleiLee, Tse-MinYu, YanpingLee, Tzong-ShyuanYu, YongLee, Wei-HwaYu, Yung-LuenLee, Wing-KeeYuan, Chiun-JyeLee, Won HeeYuan, HaoLee, Won-HaYuan, JianLee, YanYuan, Kuo-ChingLee, Yeon SunYuan, PeiguoLee, Yi-JangYuan, Shyng Shiou F.Lee, YongbokYuan, XinxuLee, YoonJungYuan, XuegangLee, Young HanYuchun, DuLee, YoungkyunYudina, LyubovLee, Yun BinYudoh, KazuoLee, YunjongYue, John K.Lees, Jennifer S.Yuemeng, JiaLeese, Henry J.Yuh, Chiou-HwaLefebvre, PhlippeYui, KunioLefebvre, VeroniqueYuk, Heung JooLefevre, Sophie D.Yukl, Erik T.Lefèvre, ThierryYumoto, HiromichiLeff, ToddYumoto, TetsuyaLeffler, JonatanYun, HongseokLefrancois, StephaneYun, Hyung-MunLegal, LucYun, Jun-WonLegembre, PatrickYung, Hong-WaLeggatt, GrahamYung, Ken Kin LamLegname, GiuseppeYura, KeiLegrand, SylvainYura, YoshiakiLegras, AntoineYurchenko, EkaterinaLehmann, ChristianYushina, Irina D.Lehner, MałgorzataYuzhalin, ArseniyLehocký, MariánYvergnaux, FlorentLehotsky, JanYves, CombarnousLehtonen, ŠárkaYvonne, BörgelingLei, FangZaballos, MiguelLei, KechengZabetakis, IoannisLei, WeiZabuchi, GiulianoLei, ZhentianZaccagnini, GermanaLeibovitch, Ilan J.Zaccaroni, AnnalisaLeibowitz, JulianZacchia, MiriamLeichtle, Stefan W.Zaccolo, ManuelaLeifels, MatsZacharias, Helena U.Leiphrakpam, PremilaZacharias, MartinLeisz, SandraZachariou, ZachariasLeitão, Jorge H.Zachos, GeorgeLeitão, José M.Zachut, MayaLeitch, Heather A.Żaczek, Anna J.Leitinger, BirgitZádor, ErnőLeitner, AlexanderZádori, ZoltánLeiva-Brondo, MiguelZaenker, KurtLeivonen, S.k.Zafrakas, MenelaosLejeune, FabriceZagidullin, NaufalLek, AngelaZagórska, AgnieszkaLek, MonkolZagrebelsky, MartaLel Khattabi, LailaZaharia, CatalinLelli, MorenoZaheer, RahatLemay, GuyZahid, MalihaLemcke, HeickoZaia, JosephLemieux, HélèneZaini, PauloLemli, BeátaZaiou, MohamedLemmermann, Niels A WZaiss, DietmarLeMoine, AllainZaitlin, DavidLemon, ChristopherZaitsev, AlekseyLemon, Jennifer A.Zając, MirosławLempiäinen, JuhaZakany, RozaLenaz, GiorgioZakaraya, RaziaLenci, ElenaZakharova, LuciaLenfant, FrançoiseZakhartchouk, AlexanderLeng, RogerZaki, Md. HasanLentini, LauraZakrocka, IzabelaLenzo, NatZakrzewska, MalgorzataLeo, Alfredo DiZalewska, AnnaLeo, Chen HueiZaltariov, Mirela-FernandaLeo, Jack ChristopherZaltzman, BorisLéon, CatherineZalubovskis, RavisLeonard, StephenZalzman, MichalLeonardi, AntonioZaman, FarasatLeonardi, RobertaZamaratskaia, GaliaLeonardi, RosaliaZamboni, AnitaLeoncini, GiulianaZambrano, Byron A.Leoncini, LorenzoZamfir, Carmen LăcrămioaraLeone, MarilisaZamocky, MarcelLeone, PhilippeŻamojć, KrzysztofLeonetti, FrancescoZamora-León, S. PilarLeonhardt, RalfZamorano-Leon, José JavierLeoni, AlbertoZamostny, PetrLeon-Juarez, MoisesZampieri, StefaniaLeonzino, MariannaZamponi, Gerald W.Lepage, CecileZamyatina, AllaLepeduš, HrvojeZamyatnin, AndreyLepenies, BerndZander, MarkLeppik, LiudmilaZanetti, MaurizioLequin, OlivierZanetti, StefaniaLerat, EmmanuelleZangari, MaurizioLermyte, FrederikZangemeister-Wittke, UweLerner, AaronZanghellini, JürgenLerner, UlfZani, Bianca MariaLerner, Ulf H.Zanlungo, SilvanaLeroy, DidierZannella, CarlaLes, FranciscoZanón-Moreno, Vicente CalixtoLeshchynska, IrynaZanotti, IlariaLesina, MarinaZanut, AlessandraLesniak, WieslawaZapata, AgustinLesov, IvanZapata, CarlosLessmann, VolkmarZapata, FabiolaLestavel, SophieZapico, Sara C.Lesuisse, DominiqueZapico, Sara CasadoLesyk, Roman B.Zapolski, TomaszLeszczynska, KatarzynaZappavigna, SilviaLetizia, TrovatoZarà, MartaLetts, JamesZaraca, FrancescoLeu, SteveZaravinos, ApostolosLeuci, ValeriaZaremba-Czogalla, MagdalenaLeung, Nicki Y.H.Zaric, SvetislavLeung, Tin-ChungZarnowska, Iwona MariaLeung, Yu minZarnowski, TomaszLevantini, ElenaZarrelli, ArmandoLevaot, NoamZarza, XavierLeVasseur, NathalieZastrow, Melissa L.Levavi-Sivan, BertaZatsepin, TimofeiLevenson, CathyZatterale, FedericaLever, TeresaZaunders, JohnLevi Sandri, Giovanni BattistaZavattari, PatriziaLevi, GiovanniZavattaro, ElisaLevick, ScottZavodnik, IlyaLevitskii, SergeyZavyalova, Elena G.Levitsky, Victor G.Zawacka-Pankau, JoannaLevy, Arkene S.A.Zawilska, Jolanta B.Levy, DanZawrotniak, MarcinLevy, StevenZbigniew, BrzozkaLewandowska, MałgorzataZbroch, EdytaLewandowska-Polak, AnnaZdanowicz, MagdalenaLewandowska-Szumieł, MałgorzataŽdralević, MašaLewczuk, BogdanZdrowowicz, MagdalenaLewicki, SławomirZdzisińska, BarbaraLewin, AlfredŻebrowska, AleksandraLewin, EwaZedník, JiříLewinska, AnnaZeeman, Samuel C.Lewis, James H.Zefferino, RobertoLewis, JoZegzouti, HichamLewis, L. KevinZehentmayr, FranzLewsey, Mathew G.Zehner, RichardLey, BenediktZeidi, MahdiLeybaert, LucZeier, ZaneLezot, FredericŻelaszczyk, DorotaLezza, Angela Maria SerenaZelepuga, ElenaLi Volti, GiovanniZelles, TiborLi, Bing-YanZelli, RiccardoLi, ChaoZeltner, NadjaLi, ChenshuangZeman, MichalLi, Chia-JungZemanova, ZuzanaLi, Chien-FengZemkova, H.Li, ChinZemmour, DavidLi, DequanZenaro, ElenaLi, DongmeiZenarruzabeitia, OlatzLi, DongqingZenclussen, Ana ClaudiaLi, DongyingZeng, HuaweiLi, FengzhiZeng, JiaLi, GangZeng, MelodyLi, GuannanZeni, OlgaLi, GuohuiZenkevich, Irog G.LI, Hai-changZentner, GabrielLi, HaoboZerbini, GianpaoloLi, HeZerdes, IoannisLi, HongchunZernii, Evgeni Yu.Li, HongdaZerze, GülLi, HongjieZettler, Lawrence W.Li, HuaZgarian, Roxana GabrielaLi, HuinanZhai, KuiLi, Hung-WenZhai, TianruiLi, JiaZhan, WenboLi, Jiao JiaoZhang, AipingLi, JiashenZhang, BaohongLi, Ji-liangZhang, CankuiLi, JinpingZhang, ChangyiLi, JunhaoZhang, ChiLi, JunxuZhang, DanLi, LeiZhang, Daniel XinLi, LianboZhang, DaweiLi, LinZhang, DeliangLi, LingZhang, DianbaoLi, LingxiaoZhang, DimingLi, LipingZhang, DongLi, Mei-LingZhang, DuoLi, MinZhang, ErshuaiLi, MingZhang, FeiLi, Paul C.H.Zhang, GeLi, Pei FengZhang, GuannanLi, PengZhang, Gui-RongLi, QuanxiZhang, GuofangLi, RenfengZhang, HongqiaoLi, RonghuiZhang, JianhuiLi, Shengwen CalvinZhang, JianminLi, ShizhaoZhang, JianyingLi, ShurenZhang, JileiLi, SunanZhang, JinweiLi, TianhuZhang, JixiangLi, TieshiZhang, KeLi, WAN-CHUNZhang, KeqiangLi, WeiZhang, LipingLi, WeihanZhang, LiqiangLi, Wei-MingZhang, Lu-PeiLi, WenboZhang, QiangLi, WenyiZhang, QiangzheLi, XiangnanZhang, QiongLi, XiaojunZhang, QiuyangLi, XiaopengZhang, QuanLi, XinZhang, RuiLi, XundeZhang, ShixiongLi, XupingZhang, ShuangLi, YinZhang, ShulingLi, YingZhang, SicongLi, YingluZhang, TingLi, Yongle (Leo)Zhang, WeijieLi, Zhi-yongZhang, WencaiLi, ZhongweiZhang, WenhengLi, ZiruZhang, WenjunLi, ZongliZhang, WenliLia Do Amaral, SandraZhang, WujieLiakina, ValentinaZhang, XiangLian, QizhouZhang, XiaohuiLiang, JunZhang, XiaotaoLiang, PeiZhang, XiaoyuLiang, Pi-HuiZhang, XinLiang, Winnie S.Zhang, XingminLiang, ZhikaiZhang, XinxingLiao, ChenZhang, XuexinLiao, DaiqingZhang, YiLiao, Jyh-feiZhang, YifanLiao, WupengZhang, Yin HuaLiao, XudongZhang, YixiangLiao, Yi-JenZhang, YixuanLiao, Yu-ChiehZhang, YonghongLiapi, CharisZhang, YongliangLiarte, SergioZhang, YumiaoLiatikus, ŽilvinasZhang, ZhanhuiLiau, Nicholas P. D.Zhang, ZhenLiaw, Chya-YanZhao, BaoyinLiberi, GiordanoZhao, ChaoLiberles, DavidZhao, ChaoyangLibich, DavidZhao, ChenLibra, MassimoZhao, GexinLiby, KarenZhao, HongboLicata, PatriziaZhao, Jie JaneLicciardello, ConcettaZhao, JinpingLicciulli, FlavioZhao, LinboLichner, ZsuzsannaZhao, Peishen (Elva)Licini, CaterinaZhao, QingnanLiebau, StefanZhao, XiaoaiLiebel, MatzZhao, YangLiebman, MichaelZhao, YingxinLiebman, Sue W.Zhao, YuguangLiebscher, JürgenZhao, YunfengLiechti, George W.Zhao, YuqiLiechti, Matthias EmanuelZhao, ZhenwenLiehr, ThomasZhdanova, Irina V.Liesveld, JaneZhelankin, Andrey V. Lietha, DanielZheleva-Dimitrova, DimitrinaLigaba-Osena, AyalewZheng, WenweiLigat, GaetanZheng, YiLihi, NorbertZheng, Yun-WenLikhatskaya, GalinaZheng, ZhongLi-Kroeger, DavidZhong, Haizhen AndrewLikus, WirginiaZhong, JinshunLillo, CathrineZhong, Wei-ZhuLim, Andy K.H.Zhong, XiaoboLim, Chin YanZhong, ZhaohuaLim, Chinten JamesZhou, BangjunLim, ChunghunZhou, BeiyanLim, DaewoonZhou, Carol L. EcaleLim, EdwinZhou, ChengjiLim, HyungyuZhou, ChiLim, Jae HyangZhou, DingyingLim, JunxianZhou, DongLim, Kee SiangZhou, Hai-bingLim, KenjiZhou, HaoLim, Ki-TaekZhou, JiaLim, Kyung-MinZhou, JiajingLim, Lee WeiZhou, JianLim, Megan S.Zhou, JichunLim, Rayne R.Zhou, Jing-JiangLim, Soon SungZhou, JingyingLim, SoyeonZhou, LeiLim, Tae-GyuZhou, LinliLima, DênisZhou, ManLima, SofiaZhou, MeiLima, Wanessa C.Zhou, MinLimbach, PatrickZhou, PhoebeLimon, AgenorZhou, QiminLimozin, LaurentZhou, RongLin, Been-RenZhou, ShanLin, Chang-ChiZhou, ShuanhuLin, Chang-ShenZhou, ShuntaiLin, Chia-HuaZhou, TianhaoLin, Chieh-HsinZhou, WeijieLin, Chien-hongZhou, WenchaoLin, Chih-ChienZhou, YiqunLin, Chih-LiZhou, ZhidongLin, Chi-JanZhou, ZhijunLin, Dar-ShongZhouravleva, Galina A.Lin, Hai-ShuZhu, BingLin, HoZhu, ChengLin, Hui-HsuanZhu, ChrisLin, Hung-YinZhu, DandanLin, I-ChaZhu, HuaLin, Jian-MingZhu, JieLin, Kuan HungZhu, JingenLin, Kwang-HueiZhu, JinshengLin, Long-LiuZhu, JunjieLin, Ming-HongZhu, Li (Licky)Lin, Muh-ShiZhu, LinLin, Pei-HuiZhu, LuchangLin, Peter P.Zhu, Michael X.Lin, RuweiZhu, Qian-HaoLin, Shian-RenZhu, QingLin, Shih-YiZhu, Qiuyu MartinLin, Shuan-PeiZhu, ShiweiLin, TaoZhu, TianyuLin, Tzu-EnZhu, WeiLin, Wei ChiehZhu, WufuLin, WeifengZhu, YuLin, Wei-YiZhu, ZhouLin, WenZhu, ZihuaLin, Wey-jinqZhu, ZiwenLin, YanghsiangZhuang, XiaoxuanLin, Ya-wenZhuGe, RonghuaLin, YibinZhukov, IgorLin, Ying-HungZhuo, ChunliuLin, Yi-TingZhuo, ZhuLin, Yu-HsuanZia, KashifLin, Yu-LingZiaee, AhmadLin, ZhiweiŽiarovská, JanaLinares-Pastén, JavierZidar, NinaLinciano, PasqualeŽídek, LukášLindahl, LasseZidovec Lepej, SnjezanaLindberg, David R.Ziegler-Graff, VéroniqueLindberg, GerrickZieleniak, AndrzejLindeman, JanZielenkiewicz, PiotrLindemann, StephanZielonka, StefanLinden, Anni-MaijaZiemann, MarkLinden, RafaelZiemnicka, KatarzynaLindenmann, JoergZieniuk, BartłomiejLinder, StigZienkiewicz, AgnieszkaLindkvist, KarinZienkiewicz, KrzysztofLindner, SteffenZientara-Rytter, KatarzynaLindsay, Andrew J.Zieseniss, AnkeLindseth, IngeZiganshina, AyratLindström, Mikael S.Zigo, MichalLinhares, PauloZilli, ThomasLinial, MichalZilliox, Michael J.Linke, DirkZimmer, JacquesLins, LaurenceZimmer, Klaus PeterLinseman, DanielZimmer, MarcLiobikas, JuliusZimmer, SebastianLionetto, Maria GiuliaZimmer-Bensch, GeraldineLionne, CorinneZimmerlin, LudovicLiopis, JuanZimmermann, Sabine DagmarLiou, Chian-JiunZimmermannová, OlgaLiou, Gunn-GuangZimowska, MałgorzataLiou, Jr-JiunZimta, Alina-AndreeaLiou, Ying-mingZingg, Jean-MarcLiovic, IvicaZironi, IsabellaLiovic, MirjanaZisapel, NavaLioy, SimoneZitman-Gal, TaliLipert, AnnaZitta, SabineLipina, RadimZivcak, MarekLipinski, Daniel M.Zloh, MireLipiński, Piotr F. J.Zmora, PawelLipke, PeterZoete, VincentLipparini, FilippoZofall, MartinLippert, ElisabethZoghbi, María ElenaLipphaus, AndreasZoidis, EvangelosLis Arias, Manuel J.Żok, TomaszLis, Grzegorz J.Żołądek, TeresaLisacek, FrederiqueZoladz, Jerzy A.Lisi, LuciaŽoldák, GabrielLisowska, KatarzynaŻołek, TeresaLissa, DelphineZoli, AngeloListos, JoannaZollino, MarcellaLitak, JakubZollner, GernotLitschauer, BrigitteŻołnowska, BeataLittle, JulianZolotukhin, Igor A.Little, MarcZondler, LisaLitvinova, Ekaterina A.Zoratti, MarioLitvinova, LarisaZorec, RobertLitwin, Ireneusz BernardZorena, KatarzynaLiu, AiminZorov, Dmitry BLiu, Chao-LinZorzan, SimoneLiu, ChaoxingZosky, GrahamLiu, Cheuk LunZottini, MichelaLiu, Chi-MingZou, ChunbinLiu, Ching-Ping LiuZou, JunjieLiu, Chung-JungZou, WeiLiu, ChunyuZou, XueqingLiu, DavidZou, ZhongweiLiu, Der-zenZovkic, IvaLIU, FANGZsurka, GaborLiu, GangZubarev, AndreyLiu, HengruiZubareva, Ekaterina VLiu, HongyuZuba-Surma, EwaLiu, Hu-chenZubiaga, AnaLiu, J. JayZubieta, ChloeLiu, JiangZucconi, LauraLiu, Jui-MingZuffo, MichelaLiu, JunZugaza, José LuisLiu, LingZujovic, ZoranLiu, LiyunZuna, JanLiu, LuŽunar, BojanLiu, MoZuñiga, SoniaLiu, NingZúñiga-Hernández, JessicaLiu, PengdaZunke, FriederikeLiu, QiZuo, LiLiu, QinghangŽupanič, AnžeLiu, QunZurawa-Janicka, DorotaLiu, RongŻurawek, MagdalenaLiu, ShenkuiŻurawska-Płaksej, EwaLiu, Shih-PingŻurek, GrzegorzLiu, Shing-HwaZurita, MarioLiu, ShirongZuryn, StevenLiu, TaoZwacka, RalfLiu, Ting-YuZweigerdt, RobertLiu, WeiZyromska, AngieszkaLiu, Wei-HsiuŻyżelewicz, Dorota

